# Red Wine May Mitigate the Risk of Intracerebral Hemorrhage by Preventing Hypertension—A Mendelian Randomization Study Combining CHARLS

**DOI:** 10.1002/fsn3.71329

**Published:** 2025-12-12

**Authors:** Fuan Zhang, Ning Ding, Lanying Zhang, Ping Zhang, Rui Liu, Ziqiang Chen, Jiao Chen, Huanhuan Li, Shengtao Yao

**Affiliations:** ^1^ Department of Neurosurgery Affiliated Hospital of Zunyi Medical University Zunyi Guizhou China; ^2^ Department of Neurosurgery and Key Laboratory of Neurotrauma Southwest Hospital, Third Military Medical University (Army Medical University) Chongqing China; ^3^ Department of Respiratory and Critical Care Medicine, Affiliated Hospital of Zunyi Medical University Zunyi Guizhou China

**Keywords:** alcohol intake, hypertension, intracerebral hemorrhage, Mendelian randomization, predictive, preventive, personalized medicine, red wine intake

## Abstract

Although alcohol is viewed as a risk factor for intracerebral hemorrhage, the causal relationship between different types of alcoholic beverages and intracerebral hemorrhage remains unclear, representing a significant gap in the field of intracerebral hemorrhage prevention. This study aims to fill this gap by investigating how daily drinking habits can help prevent intracerebral hemorrhage. Alcohol, red wine, and white wine were selected to conduct two‐sample Mendelian randomization analyses to investigate their associations with intracerebral hemorrhage. Positive results (alcohol, red wine) were further analyzed using multivariable Mendelian randomization to distinguish independent effects. Additionally, hypertension, a risk factor for intracerebral hemorrhage, was utilized for mediation Mendelian randomization analysis to preliminarily explore the mechanisms by which exposure factors influence intracerebral hemorrhage outcomes. Finally, the conclusions were validated in the Chinese population using the CHARLS database and extended the findings. Alcohol was found to be a risk factor (OR = 1.21, *p*‐value = 0.0351). Red wine was found to reduce the risk of ICH (OR = 0.61, *p*‐value = 0.0400). The protective effect of red wine was still observed in the multivariable Mendelian randomization analysis (OR = 0.55, *p*‐value = 0.0442). In the mediation analysis, red wine was found to prevent intracerebral hemorrhage by reducing hypertension (mediation = 13.45%, *p* = 0.0004). In the CHARLS‐based analysis, wine consumption was associated with a lower incidence of hypertension in comparison with teetotalers (OR = 0.54, *p*‐value < 0.0001). Our study revealed that although alcohol is generally considered a risk factor for intracerebral hemorrhage, red wine may have a protective effect against it. This protective effect is partly due to red wine's ability to reduce the incidence of hypertension, and this conclusion can be generalized to different populations.

## Graphical abstract

This study shows alcohol increases intracerebral hemorrhage risk (OR = 1.21), while red wine reduces it (two‐sample MR OR = 0.61; multivariable MR OR = 0.55), partly via lowering hypertension (mediation = 13.45%). CHARLS data find wine drinkers (especially elderly males) have lower hypertension (OR = 0.54), and this holds after multivariable logistic regression adjustment.

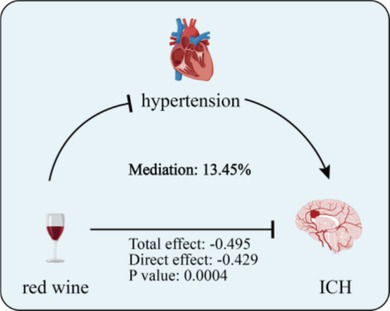

**Abbreviations**
BMI: body mass index
CAA: cerebral amyloid angiopathy
CHARLS: China Health and Retirement Longitudinal Study
EAF: effect allele frequency
GWAS: Genome Wide Association Study
HBP: high blood pressure
ICH: intracerebral hemorrhage
IVW: inverse‐variance weighted
LD: linkage disequilibrium
MR: Mendelian randomization
PPPM: predictive, preventive, personalized medicine
SNP: single nucleotide polymorphism

## 1 Introduction

Intracerebral hemorrhage (ICH), defined as bleeding into the cerebral hemisphere due to the rupture of a blood vessel within the brain, has inflicted substantial losses to human health. ICH is featured by significant annual incidence and mortality rates, with up to 3.5 million new cases annually and a 1‐year fatality rate of 50% (Puy et al. 2023). The current treatment for ICH faces major challenges. Hemostatic and antihypertensive therapies have not yielded significant positive outcomes (Qureshi et al. 2016; Sprigg et al. 2018; Steiner et al. 2016). Additionally, the therapeutic effects of both traditional and minimally invasive surgeries remain unclear (Hanley et al. 2019; Mendelow et al. 2013). Robot‐assisted surgery also faces difficulties in its promotion due to economic and technological obstacles. Therefore, thoroughly investigating the risk and protective factors associated with ICH and enhancing the level of ICH prevention are critical approaches to effectively reducing the losses caused by ICH.

Globally, more than 30% of the population is current drinkers, and the annual per capita alcohol consumption remains high, increasing from 5.5 to 6.4 L in 2005 to 2010, with projections reaching 7.6 L by 2030 (Park and Kim 2020). The health issues arising from this trend cannot be overlooked. Excessive drinking leads to chronic alcoholism, alcoholic liver disease, alcoholic cardiomyopathy, and many other illnesses, significantly elevating the risk of death. In 2020, alcohol consumption was responsible for approximately 1.78 million deaths, making it the leading risk factor for mortality among males aged 15–49 (GBD 2020 Alcohol Collaborators 2020).

Binge drinking is often identified as a significant precipitating factor for ICH (Puy et al. 2023). Research by Andrew Smyth et al. has demonstrated that current alcohol consumption can increase the risk of ICH by approximately 0.5‐fold, while heavy episodic drinking (defined as consuming more than five drinks on one or more days per month) can elevate the risk of ICH by about 0.76‐fold (Smyth et al. 2023). However, there is currently no consensus on whether different types of alcoholic beverages vary in their correlation with ICH.

From a theoretical perspective, polyphenols in wine can effectively prevent the aging of vascular endothelial cells (Botden et al. 2012). A meta‐analysis by Shao‐Hua Li et al. also demonstrated that daily grape polyphenol intake can reduce systolic blood pressure to a certain extent (Li et al. 2015). These findings suggest a protective effect against ICH. From the perspective of clinical trials, Andrew Smyth et al. found no significant correlation between wine consumption and the risk of developing ICH compared to beer and spirits (Smyth et al. 2023). Research by A G Thrift et al. suggested that consuming wine has certain benefits in preventing ICH (Thrift et al. 1999). Conversely, a study by Arthur L Klatsky et al. indicated that wine consumption might also increase the risk of ICH (Klatsky et al. 2002).

However, the studies by Andrew Smyth and A. G. Thrift are limited by the inherent shortcomings of retrospective research, which cannot completely eliminate selection bias and information bias. The prospective study by Arthur L. Klatsky spans an extended period, during which participants' drinking habits may have changed, potentially affecting the study's outcomes.

In summary, there is still a lack of high‐quality evidence‐based medical evidence regarding whether consuming wine increases or mitigates the risk of ICH. Therefore, it was innovative for this study to employ the Mendelian randomization (MR) method to explore the causal relationship between wine consumption and ICH. The MR approach utilizes specific single nucleotide polymorphisms (SNPs) as instrumental variables to simulate the effect of exposure. This method also prevents bias caused by participants underreporting or misreporting their alcohol intake, thereby allowing for more accurate and reliable conclusions (Grover et al. 2017; Teumer 2018).

## 2 Methods

### 2.1 Study Design

**FIGURE 1:**
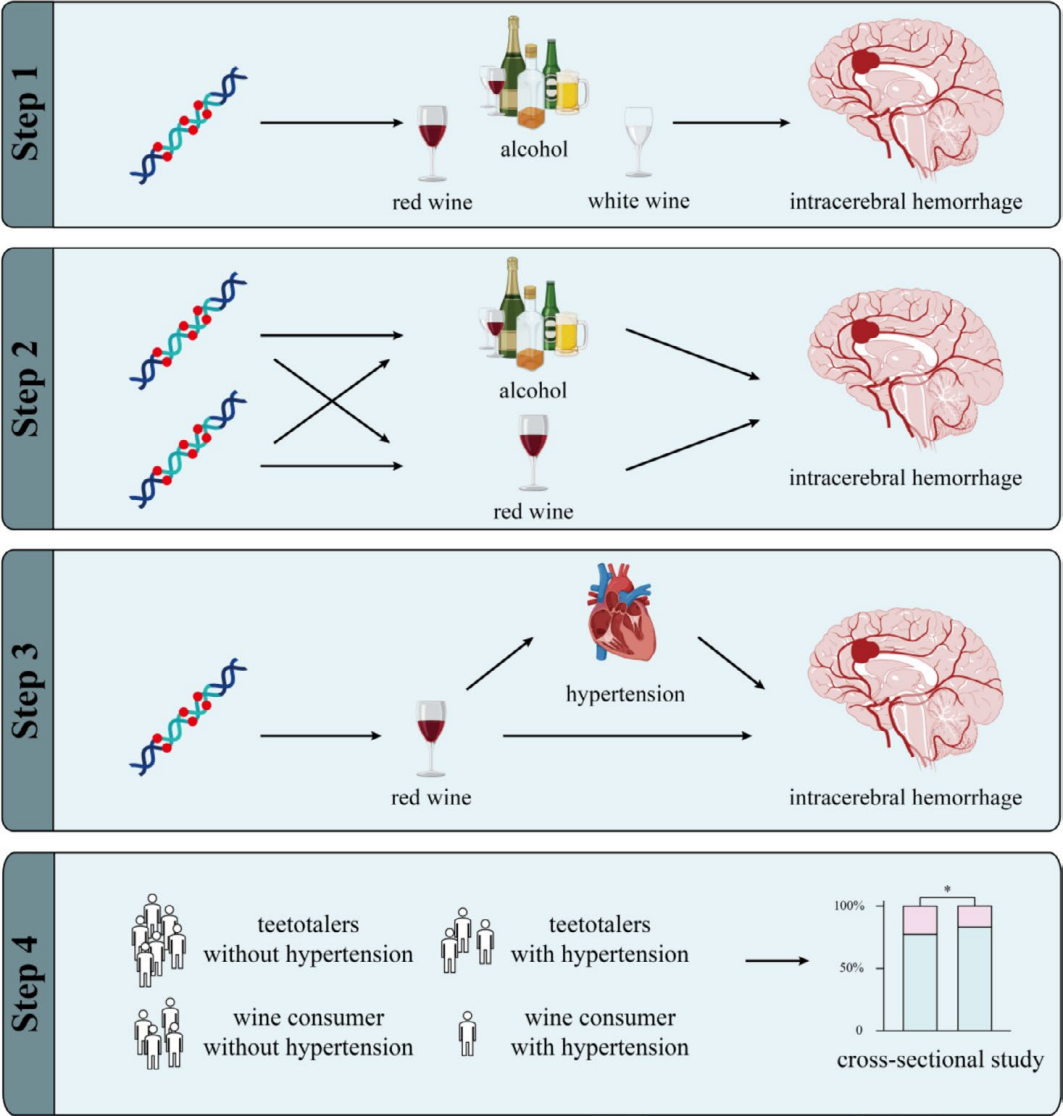
Schematic diagram of the entire study design. This article analyzes the relationship between different alcoholic beverages and ICH through two‐sample and multi‐variable Mendelian randomization and finds red wine is seemingly to lower ICH incidence rate. Mediating Mendelian randomization and CHARLS database analysis further indicates that this protective effect is partially achieved by reducing the risk of hypertension.

The entire study design is illustrated in Figure 1. Initially, Genome Wide Association Study (GWAS) data were extracted from the UK Biobank database via the IEU official website for exposure factors, while GWAS data downloaded from the FinnGen database were used for outcomes. Two‐sample MR analyses were conducted on alcohol, red wine, and white wine as three exposure factors in relation to ICH. Subsequently, positive exposures (alcohol, red wine) were selected to perform multivariable MR analysis with ICH to distinguish independent effects and eliminate confounding factors. Furthermore, hypertension, a risk factor for ICH, was selected from the FinnGen database for mediation analysis to explore the potential mechanisms through which exposure factors affect outcomes. Finally, data from 10,364 participants were extracted from the China Health and Retirement Longitudinal Study (CHARLS) database. Their drinking habits and hypertension status were analyzed using chi‐square tests to further verify the conclusions drawn.

### 2.2 Data Sources

The GWAS data utilized in this study was derived from the UK Biobank and FinnGen. GWAS data of alcohol, red wine, white wine, and BMI was online extracted from the IEU open GWAS project website (GWAS ID: ukb‐b‐5779, ukb‐b‐5239, ukb‐b‐5716, ukb‐b‐19953), which is collected and pretreated by UK Biobank (http://www.nealelab.is/uk‐biobank). The MR‐Base platform supports systematic causal inference across the human phenome (Hemani, Bowden, and Davey Smith 2018; Hemani, Zheng, et al. 2018). The UK Biobank is a large biomedical database and research resource. As a prospective epidemiological scientific research program, it has collected data from 500,000 volunteers aged between 40 and 69 years old from across the UK. The collected information includes genetic data, multimodal imaging data, and health‐related data of the participants. This research program has been ongoing since 2006, and officially, it is stated that there will be a long‐term follow‐up on the health and medical conditions of this population for the next 30 years (Sudlow et al. 2015). GWAS data of ICH and hypertension was downloaded from the FinnGen database. FinnGen represents a comprehensive research initiative in the domains of genomics and personalized medicine. This substantial public‐private partnership has amassed and scrutinized genomic and health‐related data from a cohort of 500,000 Finnish biobank contributors, with the objective of elucidating the genetic underpinnings of various diseases. The project is currently broadening its scope to encompass the study of disease progression and the underlying biological mechanisms. FinnGen serves as a premier resource, facilitating further advancements in the realms of disease prevention, diagnosis, and therapeutic intervention, while also offering insights into the intricacies of our genetic constitution (Kurki et al. 2023). This article also utilizes questionnaire data from the official website of CHARLS, extracting participants' drinking habits and hypertension conditions for cross‐sectional research. CHARLS aims to collect a set of high‐quality micro‐data representing families and individuals aged 45 years and above in China. CHARLS is a commonly used and widely recognized data source for researching issues related to population aging and promoting interdisciplinary research on aging (Zhao et al. 2014). All databases utilized in this study (UK Biobank, FinnGen, CHARLS) are public databases that have obtained prior ethical approval, and they are widely recognized and extensively used in academic research.

### 2.3 Instrument Selection and MR Analyses

Firstly, three two‐sample MR analyses were conducted. Different types of alcoholic beverages, extracted online from the IEU website, were used as exposure factors. SNPs with *p*‐value < 5E‐6 and an effect allele frequency (EAF) between 0.001 and 0.999 were selected (Yu et al. 2023). Next, the *F*‐statistic for each SNP was calculated using the formula *R* ^2^ = 2 × *β* ^2^ × EAF × (1 − EAF) and *F* = (samplesize−2) × *R* ^2^/(1 − *R* ^2^), excluding SNPs with *F* < 10 to obtain strongly associated SNPs (Weng et al. 2023). Linkage disequilibrium (LD)‐based clumping was then performed with parameters clump_kb = 10,000 and clump_r2 = 0.001 to ensure SNP independence (R package TwoSampleMR version 0.6.3). The candidate instrumental SNPs were merged with outcome GWAS data from the FinnGen database, excluding SNPs not present in the outcome GWAS data and those with a *p*‐value < 5E‐6 for their association with the outcome. During the harmonization process, palindromic and incompatible SNPs were removed, and the alleles of the exposure and outcome SNPs were aligned to obtain the final instrumental SNPs. Subsequently, two‐sample MR analyses were conducted using inverse‐variance weighted (IVW) as the primary method, with MR Egger, Weighted Median, Simple Mode, and Weighted Mode as supplementary methods. The MR analysis results were considered positive only if the IVW analysis yielded a significant result (*p* < 0.05) and the β values from the other four methods were consistent with the IVW direction. To further validate the reliability of the two‐sample MR analysis results, heterogeneity and pleiotropy analyses were performed. Heterogeneity analysis helped assess the robustness and reliability of the MR results by identifying significant differences in instrumental variables, while pleiotropy analysis was used to test for potential confounding factors, thereby avoiding misinterpretation of causal relationships and enhancing result stability (Hemani, Bowden, and Davey Smith 2018; Zou et al. 2022).

Next, the positive exposure factors (alcohol and red wine) from the two‐sample MR analysis were selected for a further multivariable MR analysis. Four methods were employed for the multivariable MR analysis: Multivariable inverse‐variance weighted method, Multivariable MR‐Egger method, Multivariable MR‐Lasso method, and Multivariable median method (R package Mendelian Randomization, version 0.10.0). To ensure the reliability of the results, the Q‐Statistic was calculated to assess pleiotropy (R package MVMR, version 0.4). The multivariable analysis results were considered reliable only if the *p*‐value was greater than 0.05, indicating no pleiotropy.

Sequentially, a mediation MR analysis was performed. Hypertension, a recognized risk factor for ICH, was chosen as the mediator. The GWAS data for hypertension was downloaded from the FinnGen database. Based on the previously validated causal relationship between red wine and ICH, a reverse two‐sample MR from ICH to red wine was conducted, demonstrating no reverse causality. Subsequently, two two‐sample MRs were conducted: from red wine to hypertension and from hypertension to ICH. Although no pleiotropy was detected, heterogeneity tests were positive, so the method of MR‐PRESSO was utilized to analyze and exclude outlier SNPs to enhance result robustness (Ruan et al. 2023). Thus, the beta1 of red wine to hypertension and beta2 of hypertension to ICH were calculated. The instrumental SNPs used in the hypertension to ICH analysis were not directly related to red wine and were not used in the red wine to hypertension MR analysis. The mediation effect and direct effect of red wine to ICH were calculated based on beta1, beta2, and previously calculated total effect beta. Additionally, the *p*‐value was calculated based on the *Z*‐value to confirm the statistical significance of the mediation effect.

Finally, due to the severe missing of the BMI data in the CHARLS database, a multivariable Mendelian randomization study with red wine and BMI as exposure factors and hypertension as the outcome factor was further conducted, aiming to exclude the influence of BMI—a key risk factor for hypertension. The operation of this step is roughly similar to the aforementioned multivariable Mendelian randomization process, so it will not be repeated here.

### 2.4 Cross‐Sectional Study

The questionnaire data from the CHARLS for the years 2011, 2013, 2015, and 2018 was downloaded. First, the questionnaire data for each year was screened separately. The participants who reported “drinking alcohol in the past year” but did not specify the type of alcohol consumed or reported consuming multiple types of alcoholic beverages simultaneously were excluded. The participants who did not report hypertension status were also excluded. After preprocessing the questionnaire data for each year, the data from these 4 years was merged. For participants included in the study multiple times, only the data from their first inclusion was preserved. This resulted in a total of 17,759 lines of data. The data was then retained for individuals aged over 55, resulting in 10,364 lines of data. These 10,364 lines of data were analyzed, separately counting the number of individuals who never drank alcohol and did not have hypertension, those who never drank alcohol but had hypertension, those who drank wine but did not have hypertension, and those who drank wine and had hypertension. Pearson's chi‐squared test was performed to evaluate the association between exposure and outcome (Wang et al. 2020). Multivariate logistic regression analysis was used to adjust for confounding factors such as “age”, “income”, “gender”, “marital status”, and “smoking”, and based on this, to examine the association between “wine consumption” and hypertension.

## 3 Results

### 3.1 GWAS Data Results

In the two‐sample MR analysis, the GWAS data for alcohol was filtered using a threshold of *p* = 5E‐6, excluding weak candidate instrumental variables with an F‐statistic less than 10. After LD‐based clumping, 254 candidate instrumental SNPs were obtained. Upon merging with the outcome GWAS data for ICH, 247 SNPs remained. Following harmonization, 10 SNPs (rs10274474, rs10779523, rs10903720, rs1104608, rs117799466, rs1894544, rs2159935, rs4419791, rs62097995, rs803663) were excluded, and the remaining SNPs were not associated with the ICH outcome. Consequently, 237 instrumental SNPs were finally retained to analyze the causal relationship between alcohol and ICH. The GWAS data for red wine were filtered using a threshold of *p* = 5E‐6, excluding weak candidate instrumental variables with an F‐statistic less than 10. After LD‐based clumping, 100 SNPs were preserved. Upon merging with the outcome GWAS data for ICH, 97 candidate instrumental variables remained. Following harmonization, 2 SNPs (rs2270494, rs9388171) were eliminated, and the remaining SNPs were not associated with the ICH outcome. Consequently, 95 instrumental SNPs were selected to analyze the causal relationship between red wine and ICH. The GWAS data for white wine were filtered using a threshold of *p* = 5E‐6, excluding weak candidate instrumental variables with an F‐statistic less than 10. After clumping, 49 candidate instrumental SNPs were attained. Upon merging with the outcome GWAS data for ICH, 47 SNPs remained. Following harmonization, 1 SNP (rs78268708) was excluded, and the remaining SNPs were not associated with the ICH outcome. Consequently, 46 instrumental SNPs were utilized to analyze the causal relationship between white wine and ICH.

In the multivariable MR analysis, the instrumental SNPs for alcohol and red wine were first combined (237 SNPs for alcohol and 95 SNPs for red wine), resulting in 329 SNPs. After performing clumping again, 266 SNPs were obtained. Upon verification, these 266 SNPs could be mapped to the GWAS data for alcohol, red wine, and ICH, thus serving as the final instrumental SNPs for the multivariable MR analysis (Fazia et al. 2020).

In the mediation MR process, three additional two‐sample MR analyses were conducted. In the MR analysis of ICH to red wine, the GWAS data for ICH were filtered using a threshold of *p* = 5E‐6, excluding weak candidate instrumental variables with an *F*‐statistic less than 10. After LD‐based clumping, 12 candidate instrumental SNPs were obtained. Upon merging with the outcome GWAS data for red wine, 11 SNPs remained. Following harmonization, 11 SNPs were retained, all of which were not associated with the red wine outcome. Consequently, 11 instrumental SNPs were obtained to analyze the causal relationship between ICH and red wine. In the two‐sample MR analysis of red wine to hypertension, the 95 SNPs harmonized in the previous red wine to ICH analysis were used as the basis. Two SNPs associated with the hypertension outcome (rs17817497, rs303753) and seven outlier SNPs (rs1229984, rs2383361, rs300918, rs35488630, rs4900965, rs568030, rs7433378) were excluded. Consequently, 86 instrumental SNPs were obtained to analyze the causal relationship between red wine and hypertension. The GWAS data for hypertension were filtered using a threshold of *p* = 5E‐6, excluding weak candidate instrumental variables with an *F*‐statistic less than 10. After LD‐based clumping, 383 candidate SNPs were obtained. Upon merging with the outcome GWAS data for ICH, all 383 SNPs remained. Following harmonization, 11 SNPs (rs10832778, rs12534129, rs1558901, rs1745414, rs2302661, rs4977575, rs564065, rs6565175, rs7088877, rs9812885, rs9851392) were excluded. Five outlier SNPs (rs1879053, rs2246363, rs2704368, rs604723, rs915894) were also excluded to avoid heterogeneity, resulting in 367 SNPs. These 367 SNPs were not associated with the ICH outcome. Consequently, 367 instrumental SNPs were obtained to analyze the causal relationship between hypertension and ICH.

### 3.2 MR Results

In this study, all pleiotropy analyses were negative, indicating a low likelihood that the instrumental SNPs influenced the outcome through pathways other than the exposure factors, thereby supporting the assumptions of independence and exclusivity. Except for one step in the mediation analysis where heterogeneity was present, all other analyses passed the heterogeneity tests, suggesting that the MR results were robust and highly reproducible.

**FIGURE 2:**
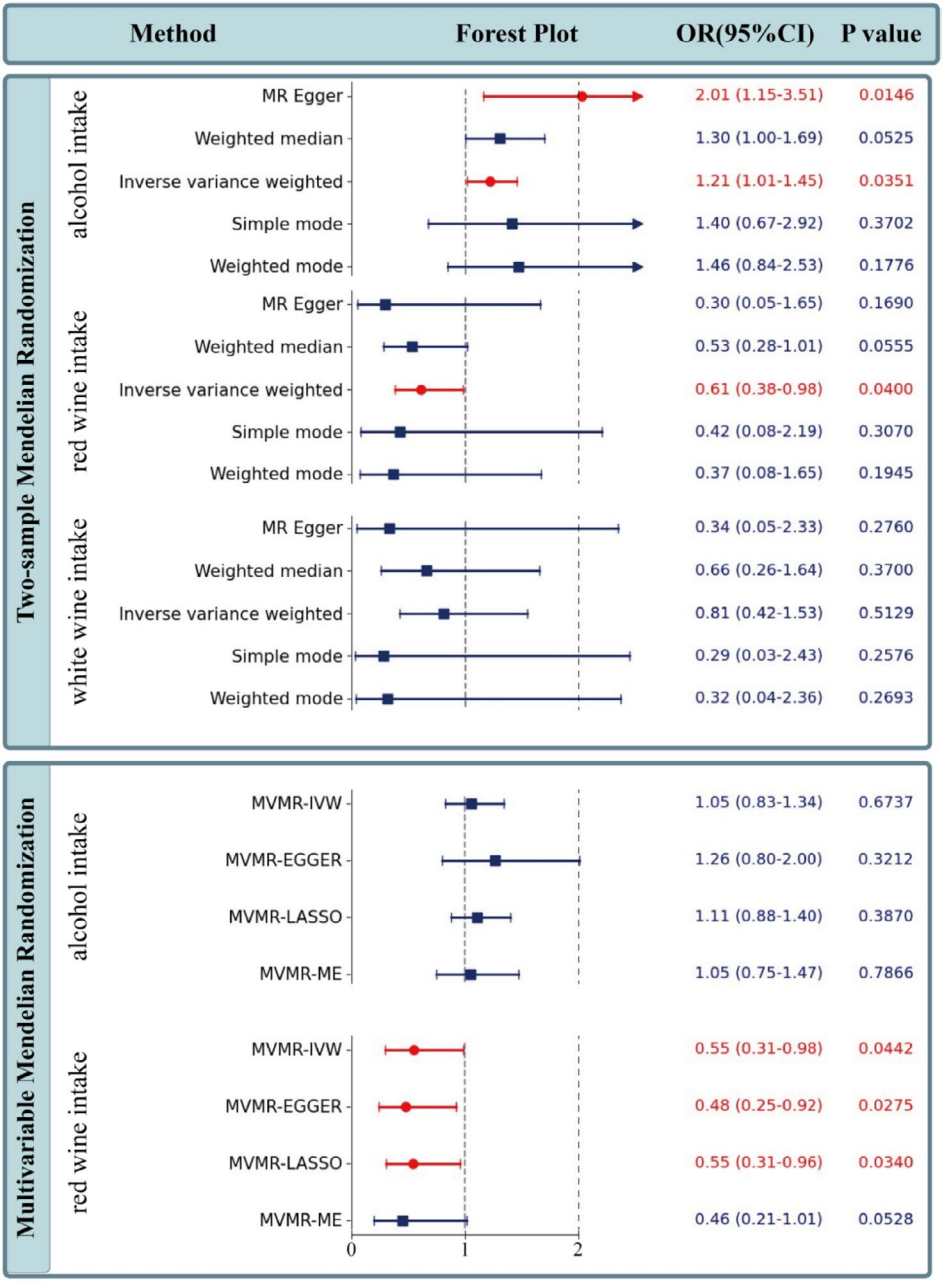
Forest plot of two‐sample and multivariable MR results. In the forest plot, red lines with circular dots indicate statistical significance, while blue lines with square dots indicate statistical non‐significance. If the endpoints of the lines are short vertical lines, they represent the actual end of the line; if they are arrows, it indicates that the interval's maximum value exceeds 2.5. OR (95% CI) and PI are shown in blue if statistically non‐significant and in red if statistically significant.

Through two‐sample MR analyses, alcohol and red wine were found to have causal relationships with ICH. Alcohol, as a risk factor for ICH, was found to increase the risk of ICH by approximately 20% (*p*‐value = 0.0351). Conversely, red wine, as a protective factor, was found to reduce the risk of ICH by approximately 39% (*p*‐value = 0.04). No significant causal relationship was found between white wine and ICH (Figure 2). The differing effects of red wine and white wine may explain the varying conclusions of previous studies, as past research has not distinguished between the different types of wine.

Multivariable MR analysis demonstrated that, after adjusting for the factor of alcohol, red wine remained a protective factor for ICH, with an even more pronounced protective effect (OR = 0.55, *p*‐value = 0.04). This suggests that active components in red wine, independent of alcohol, may be key factors in preventing ICH. The causal relationship between alcohol and ICH was not significant in this part of the analysis (Figure 2).

**FIGURE 3:**
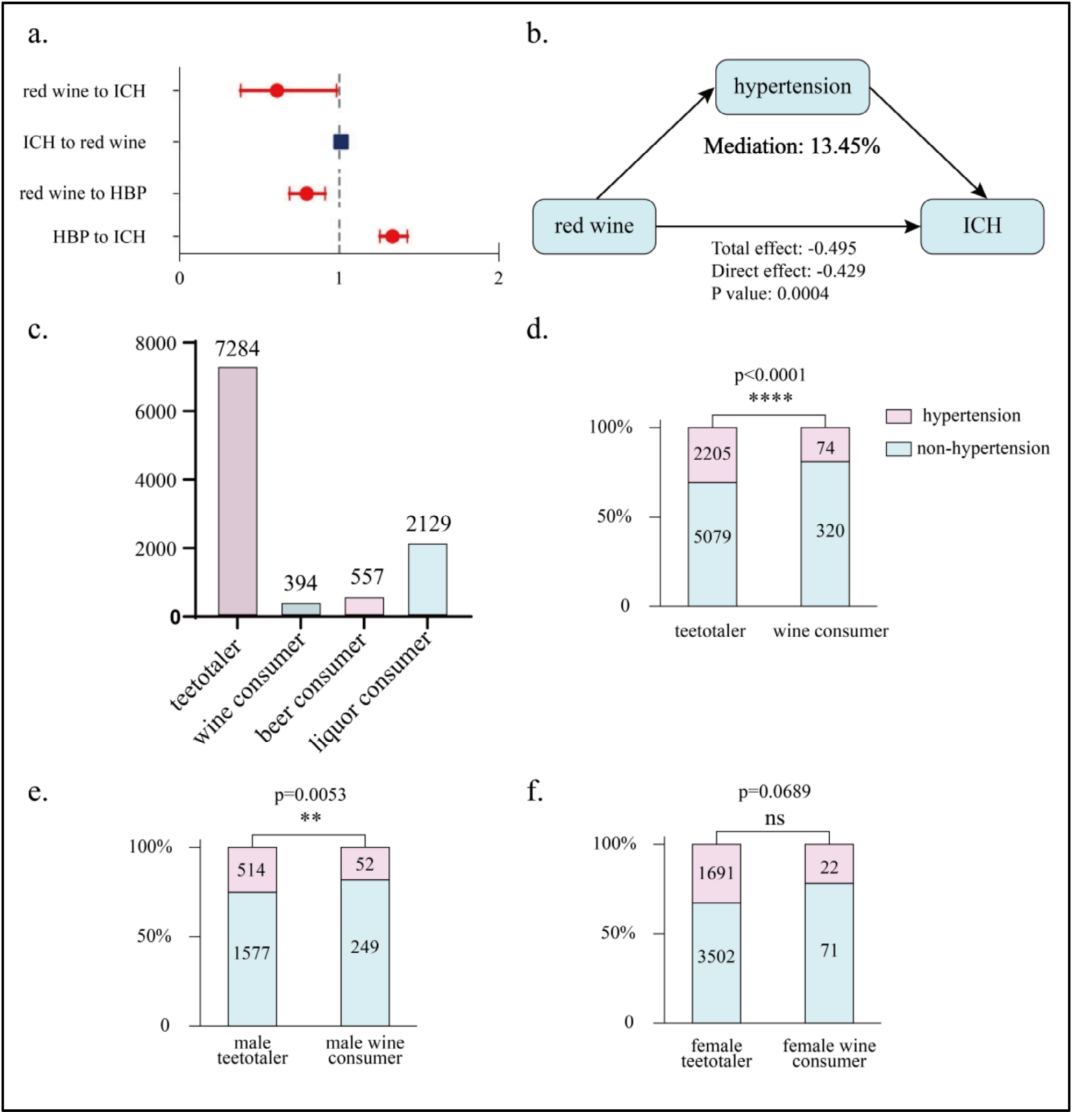
Forest plot of mediation MR results and CHARLS data analysis. (a) The forest plot of mediation MR. Red lines with circular dots indicates statistical significance, and blue lines with square dots indicates statistical non‐significance. (b) The schematic diagram of mediation MR. (c) the distribution of alcohol choices among the population extracted from the CHARLS. (d, e, f). The chi‐square statistical charts displaying the number of groups extracted from the CHARLS database. ICH stands for intracerebral hemorrhage. HBP stands for high blood pressure.

In the mediation MR analysis, the mediation effect of red wine in preventing ICH through the prevention of hypertension was found to have a *β* value of approximately −0.067, with a mediation effect proportion of about 13.45% (*p*‐value = 0.0004; Figure 3). This indicates that red wine may help prevent the occurrence of ICH partially by preventing hypertension.

### 3.3 Cross‐Sectional Results

Data for 10,364 participants aged over 55 was extracted from the CHARLS database. These 10,364 lines of data were analyzed to separately count the number of individuals who never drank alcohol and did not have hypertension, those who never drank alcohol but had hypertension, those who drank wine but did not have hypertension, and those who drank wine and had hypertension. Initially, a chi‐squared test without gender differentiation was conducted, indicating that the prevalence of hypertension was indeed lower in the wine‐drinking group compared to the non‐drinking group (*p*‐value < 0.0001). Subsequently, chi‐squared tests based on gender subgroups were performed, revealing that the aforementioned conclusion held true for males (*p*‐value = 0.0053) while not statistically significant in the females (*p*‐value = 0.0689; Figure 3). The statistical power was calculated (R package pwr, version 1.3.0) and the value is 0.9993927, which exceeds the conventional threshold of 0.8. This indicates a very high probability (over 99.9%) of detecting a true effect of the hypothesized magnitude in the study, minimizing the risk of Type II error. Therefore, the consumption of red wine was associated with a lower prevalence of hypertension in elderly males and females, consistent with the findings from the MR analysis presented earlier.

### 3.4 Correlation Analysis Adjusted Based on Baseline Levels

**FIGURE 4:**
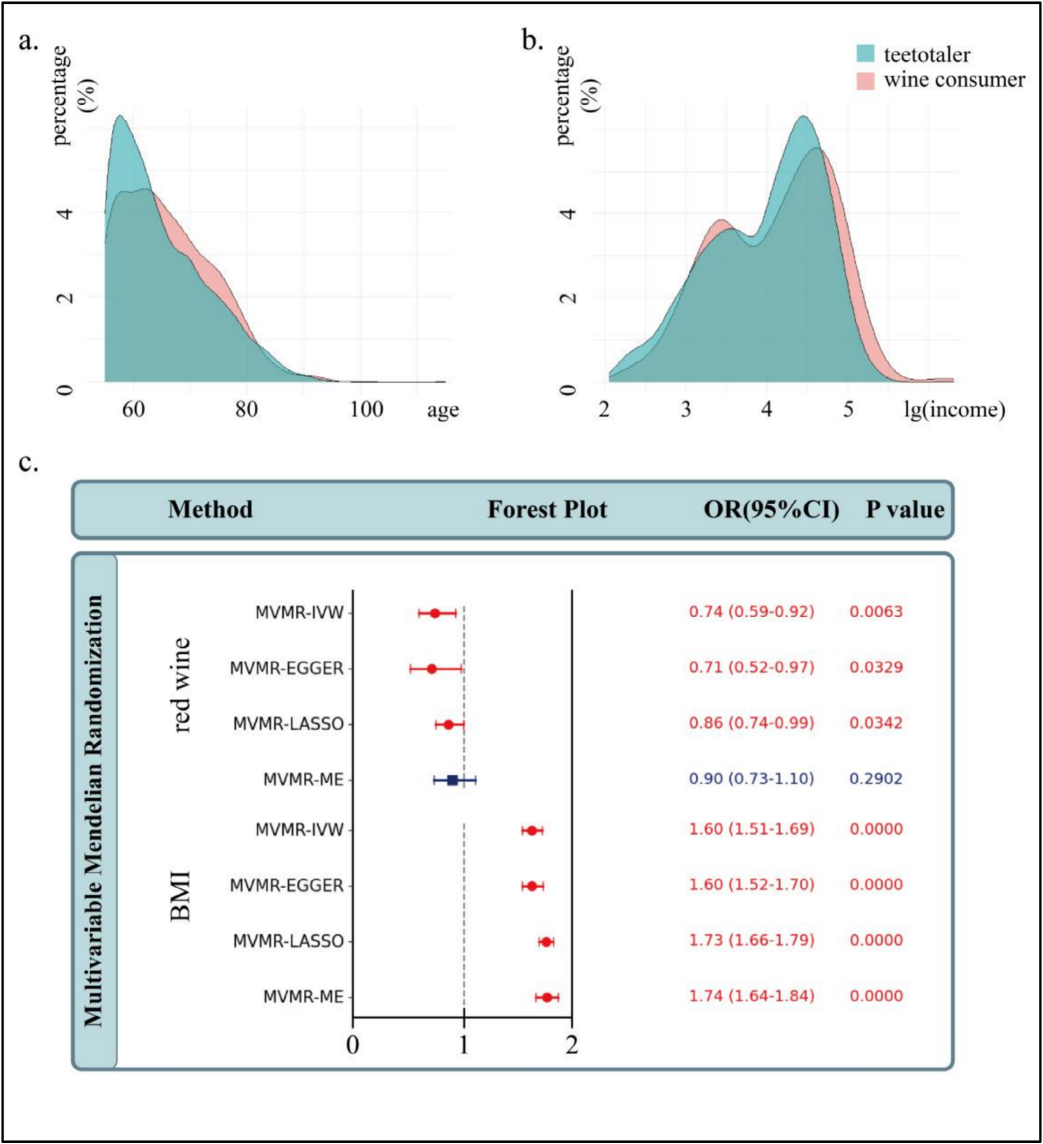
The age and income distributions of teetotalers and wine consumers, the results of MVMR analyses using red wine and BMI as exposures, and hypertension as outcome. In the forest plot, red lines with circular dots indicate statistical significance, while blue lines with square dots indicate statistical non‐significance. OR (95% CI) and *p* value are shown in blue if statistically non‐significant and in red if statistically significant.

Table 1 and Figure 4 together present the characteristics of the main baseline indicators for two groups of people: wine consumers and teetotalers. It should be noted that the baseline characteristic of BMI was not presented, which is strongly correlated with hypertension. This is because BMI in the CHARLS database is severely missing. Taking wine consumers with hypertension as an example, the missing value of BMI is as high as 39.06%. Therefore, it seems inappropriate to conduct analysis and correction based on this BMI. So only high‐quality baseline data comparisons are shown in the table. It can be seen from the table that there are differences between these two groups at multiple important baseline levels. The logistic regression analysis corrected according to these different baseline indicators respectively suggests that wine consumers still have a significant correlation with a lower prevalence of hypertension, further supporting our scientific hypothesis.

**TABLE 1:** Baseline subject characteristics, the analysis of baseline level differences, and adjusted forest plot based on differential baseline indicators. Numbers are % (*N*) unless otherwise noted. IQR indicates interquartile range from P25 to P75. The *p*‐value column presents the results of baseline comparisons accounting for different confounding factors. WRS Test stands for Wilcoxon rank sum test. *χ* ^2^ test stands for Chi‐squared test. FET stands for Fisher's exact test. The adjusted forest plot column presents the results of correlation analysis adjusted by multivariate logistic regression analysis, based on the baseline data with discrepancies.

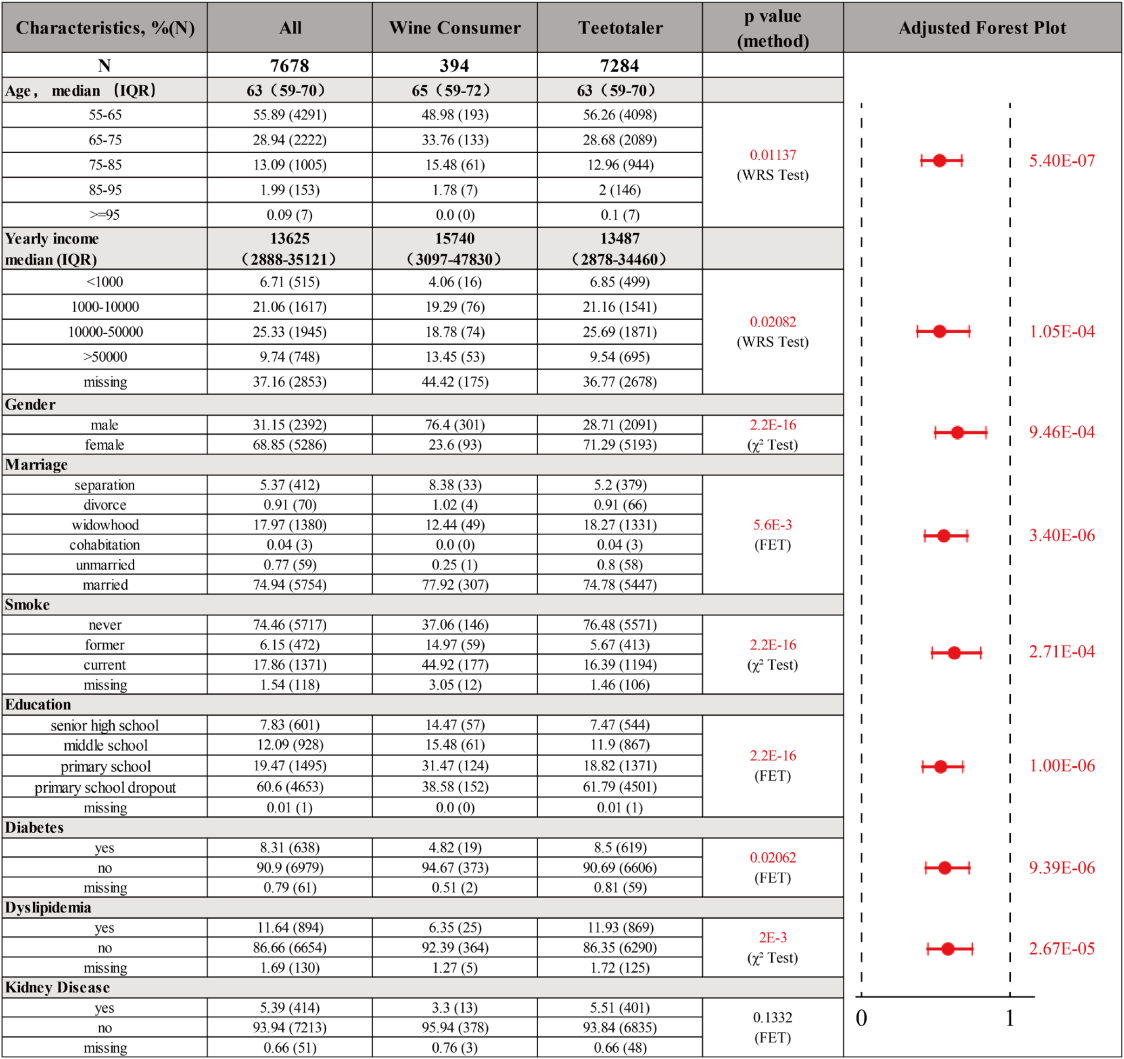

In addition, the missing BMI was also included in the analysis by means of multivariable Mendelian randomization to exclude independent effects (Figure 4c). After adjusting for BMI, red wine is still a protective factor for hypertension (OR = 0.74, *p*‐value = 0.0063).

## 4 Discussion

Various biases have always been inevitable and significant influencing factors in clinical research (Gluud 2006). Particularly, drinking habits, which are often in a state of unstable dynamic change, pose a considerable challenge to clinical studies focusing on alcohol consumption due to the potential for subjective underreporting by participants (Zhao et al. 2023). Therefore, this study leverages the advantages of MR to effectively avoid the interference of other confounding factors and the impact of participant misreporting, thereby providing high‐quality evidence‐based medical evidence (Davies et al. 2018). Moreover, MR studies can save time and financial costs associated with long‐term large‐scale clinical research, avoid biases caused by sample loss over long trial periods, and circumvent ethical controversies. Additionally, the use of multivariable MR and mediation MR methods in this study not only effectively distinguishes independent effects but also explores the mechanisms of causal effects to some extent. This study also incorporates data from the CHARLS database to further validate the findings in the Chinese population, enhancing the reliability of the research. Therefore, from a methodological perspective, this MR‐based study possesses advantages that distinguish it from other similar studies.

In this study, two‐sample MR was used to ascertain that while alcohol consumption elevates the risk of ICH, this risk is not uniform across all types of alcoholic beverages. Specifically, white wine consumption was not significantly associated with an increased risk of ICH, whereas red wine consumption may confer a protective effect against ICH. These findings warrant further investigation into the compositional differences among various alcoholic beverages. Notably, white wine did not demonstrate the same level of protection against ICH as red wine in our MR analysis, which may be attributed to the substantially higher polyphenol content in red wine compared to white wine (Campanella et al. 2004). The failure to distinguish the different effects of red wine and white wine may also be the reason why previous studies have reached different conclusions. Therefore, from the perspective of reviewing past research and improving the design of subsequent clinical investigations, this article has significant promotional and enlightening effects.

In this study, two‐sample MR was initially employed to identify potential risk and protective factors associated with ICH. Subsequently, multivariable MR was conducted to further confirm the protective effect of red wine in reducing the risk of ICH, while eliminating potential confounding factors. Next, hypertension, a risk factor for ICH, was selected for mediation analysis to explore the potential mechanisms by which the exposure factor affects the outcome. Finally, the aforementioned conclusions were further validated in the Chinese population, demonstrating the generalizability of the findings. Therefore, from a research design perspective, this study is comprehensive, rigorous, and convincing. Despite the significant implications of this study, some limitations remain.

Firstly, although no pleiotropy was detected in any of the analytical steps in this study, heterogeneity was positive in the mediation analysis. In the two‐sample MR analysis of red wine to hypertension, outlier SNPs (rs1229984, rs2383361, rs300918, rs35488630, rs4900965, rs568030, rs7433378) were excluded, raising the heterogeneity test *p*‐value from 1.71E‐09 to 3.99E‐03. In the two‐sample MR analysis of hypertension to ICH, outlier SNPs (rs1879053, rs2246363, rs2704368, rs604723, rs915894) were excluded, raising the heterogeneity test *p*‐value from 2.64E‐02 to 4.08E‐01. This heterogeneity may stem from different analytical platforms, experiments, and populations. Although it does not directly invalidate the conclusions of our MR analysis, it somewhat reduces the robustness and reproducibility of the results, warranting attention.

Secondly, in the fourth step involving the CHARLS database analysis, logically, the focus should have been on red wine rather than wine, and on the outcome of ICH rather than the intermediary factor of hypertension. However, due to the limitations of the CHARLS database, neither red wine as an indicator nor ICH as an outcome could be identified. Consequently, only the relationship between wine and the intermediary factor of hypertension was validated, which somewhat reduced the completeness of the study.

Thirdly, although this study revealed that red wine could reduce the risk of ICH and partially attributed this to the reduction in hypertension incidence, the mediation effect proportion was only about 13.45%, indicating the presence of other mechanisms. Potential mechanisms such as reducing oxidative stress damage to vascular endothelium, improving endothelial cell function, and increasing vascular elasticity were not fully discussed in this study and warrant further basic research exploration.

This study lacked a quantitative investigation into the relationship between red wine consumption and ICH, failing to explore the correlation between varying levels of red wine intake and the incidence of ICH, and consequently, did not provide an optimal recommended value. During the research process, an attempt was made to use the wine consumption data from the CHARLS database for quantification. However, after extracting the relevant indicators, it was found that the completeness of responses to this question was severely lacking, potentially introducing significant bias, insufficient to support a quantitative analysis based on red wine consumption.

## 5 Interpretation

In this study, the use of SNP instruments validated that red wine reduces the risk of hypertension and prevents the occurrence of ICH in European populations, while alcohol consumption potentially increases the risk of ICH. Additionally, a cross‐sectional study in the Chinese population confirmed the correlation between wine consumption and a reduced incidence of hypertension. These findings can guide us in adopting a balanced diet and healthy lifestyle habits to reasonably avoid the occurrence of ICH.

## Author Contributions

**Fuan Zhang:** formal analysis (equal), writing – original draft (equal). **Ning Ding:** formal analysis (equal), writing – original draft (equal). **Lanying Zhang:** visualization (supporting). **Ping Zhang:** visualization (supporting). **Rui Liu:** validation (supporting). **Ziqiang Chen:** data curation (supporting). **Jiao Chen:** resources (supporting). **Huanhuan Li:** supervision (equal), writing – review and editing (equal). **Shengtao Yao:** supervision (equal), writing – review and editing (equal).

## Funding

This work was supported by the National Natural Science Foundation of China (Grant number 82260247, granted to Shengtao Yao) and the Master Scientific Research Start‐up Fund of the Affiliated Hospital of Zunyi Medical College (No. 36 [2018], YZ, granted to Fuan Zhang).

## Ethics Statement

Data used in this article was from databases that obtained ethical approval a priori. All information was obtained from publicly available databases without sensitive or restrained data involving intelligence, education, social outcomes such as income.

## Consent

All authors approved the final manuscript and the submission to this journal.

## Conflicts of Interest

The authors declare no conflicts of interest.

## Supporting information


**Table S1:** Detailed information for the GWAS datasets used in the study.
**Table S2:** Instrumental variables used in two‐sample MR analysis of alcohol to ICH.
**Table S3:** Instrumental variables used in two‐sample MR analysis of red wine to ICH.
**Table S4:** Instrumental variables used in two‐sample MR analysis of white wine to ICH.
**Table S5:** Instrumental variables used in multivariable MR analysis of alcohol to ICH.
**Table S6:** Instrumental variables used in multivariable MR analysis of red wine to ICH.
**Table S7:** Instrumental variables used in two‐sample MR analysis of ICH to red wine.
**Table S8:** Instrumental variables used in two‐sample MR analysis of red wine to hypertension.
**Table S9:** Instrumental variables used in two‐sample MR analysis of hypertension to ICH.
**Table S10:** Instrumental variables used in MVMR analysis of red wine to hypertension.
**Table S11:** Instrumental variables used in MVMR analysis of BMI to hypertension.
**Table S12:** Two‐sample MR analysis result of alcohol to ICH.
**Table S13:** Two‐sample MR analysis result of red wine to ICH.
**Table S14:** Two‐sample MR analysis result of white wine to ICH.
**Table S15:** Multivariable MR analysis result of alcohol and red wine to ICH. Multivariable inverse‐variance weighted method.
**Table S16:** Two‐sample MR analysis result of ICH to red wine.
**Table S17:** Two‐sample MR analysis result of red wine to hypertension.
**Table S18:** Two‐sample MR analysis result of hypertension to ICH.
**Table S19:** Multivariable MR analysis result of BMI and red wine to hypertension.
**Table S20:** STROBE‐MR checklist of recommended items to address in reports of Mendelian randomization studies.

## Data Availability

Data from MR‐base (https://gwas.mrcieu.ac.uk/) and the FinnGen consortium (https://www.finngen.fi/en) is publicly available. Other details like GWAS_ID can be found in Supporting Information. Any researcher can access the data used in this article from MR‐base and FinnGen according to the GWAS_ID provided. The CHARLS data used in this work is publicly available. The data from CHARLS is unrestricted, and any researcher can apply for it with the aim of scientific research from the CHARLS website for free. The URL is https://charls.charlsdata.com/. After obtaining the raw data in DTA format from CHARLS, it can be read using Stata software in conjunction with the corresponding year's code_book, thereby extracting the relevant data employed in this research. Further inquiries can be directed to the corresponding authors. Besides, in this study, these data are utilized solely for scientific purposes, without any commercial interests, and do not involve sensitive topics such as intelligence, ethnicity, or religion.
