# Supplementary material for: Red Wine May Mitigate the Risk of Intracerebral Hemorrhage by Preventing Hypertension—A Mendelian Randomization Study Combining CHARLS
Source: Food Sci Nutr. 2025 Dec 12;13(12):e71329. doi: 10.1002/fsn3.71329 (PMC12701324; doi:10.1002/fsn3.71329)
Supplement: Supplementary file 1 — Table S1: Detailed information for the GWAS datasets used in the study. Table S2: Instrumental variables used in two‐sample MR analysis of alcohol to ICH. Table S3: Instrumental variables used in two‐sample MR analysis of red wine to ICH. Table S4: Instrumental variables used in two‐sample MR analysis of white wine to ICH. Table S5: Instrumental variables used in multivariable MR analysis of alcohol to ICH. Table S6: Instrumental variables used in multivariable MR analysis of red wine to ICH. Table S7: Instrumental variables used in two‐sample MR analysis of ICH to red wine. Table S8: Instrumental variables used in two‐sample MR analysis of red wine to hypertension. Table S9: Instrumental variables used in two‐sample MR analysis of hypertension to ICH. Table S10: Instrumental variables used in MVMR analysis of red wine to hypertension. Table S11: Instrumental variables used in MVMR analysis of BMI to hypertension. Table S12: Two‐sample MR analysis result of alcohol to ICH. Table S13: Two‐sample MR analysis result of red wine to ICH. Table S14: Two‐sample MR analysis result of white wine to ICH. Table S15: Multivariable MR analysis result of alcohol and red wine to ICH. Multivariable inverse‐variance weighted method. Table S16: Two‐sample MR analysis result of ICH to red wine. Table S17: Two‐sample MR analysis result of red wine to hypertension. Table S18: Two‐sample MR analysis result of hypertension to ICH. Table S19: Multivariable MR analysis result of BMI and red wine to hypertension. Table S20: STROBE‐MR checklist of recommended items to address in reports of Mendelian randomization studies. [file FSN3-13-e71329-s001.zip › fsn371329-sup-0001-TableS1@supplementary_data1_GWAS_database.docx]

**Table S1.** Detailed information for the GWAS datasets used in the study

| **Phenotypes** | **GWAS ID** | **Data Source** | **Population** | **Sex** | **Sample Size** |
| --- | --- | --- | --- | --- | --- |
| alcohol intake frequency | ukb-b-5779 | UKBiobank | European | Both | 462346 |
| average weekly red wine intake | ukb-b-5239 | UKBiobank | European | Both | 327026 |
| average weekly champagne plus white wine intake | ukb-b-5716 | UKBiobank | European | Both | 326801 |
| Body mass index (BMI) | ukb-b-19953 | UKBiobank | European | Both | 461460 |
| intracerebral haemmorrhage | finngen_R10_I9_ICH | FinnGen | Finnish | Both | 375773 |
| hypertension | finngen_R10_I9_HYPTENS | FinnGen | Finnish | Both | 412113 |

Notes: First three lines of GWAS data were obtained through the IEU database with the listed GWAS ID. The last two lines of GWAS data were obtained through the FinnGen database.
