# Supplementary material for: Red Wine May Mitigate the Risk of Intracerebral Hemorrhage by Preventing Hypertension—A Mendelian Randomization Study Combining CHARLS
Source: Food Sci Nutr. 2025 Dec 12;13(12):e71329. doi: 10.1002/fsn3.71329 (PMC12701324; doi:10.1002/fsn3.71329)
Supplement: Supplementary file 1 — Table S1: Detailed information for the GWAS datasets used in the study. Table S2: Instrumental variables used in two‐sample MR analysis of alcohol to ICH. Table S3: Instrumental variables used in two‐sample MR analysis of red wine to ICH. Table S4: Instrumental variables used in two‐sample MR analysis of white wine to ICH. Table S5: Instrumental variables used in multivariable MR analysis of alcohol to ICH. Table S6: Instrumental variables used in multivariable MR analysis of red wine to ICH. Table S7: Instrumental variables used in two‐sample MR analysis of ICH to red wine. Table S8: Instrumental variables used in two‐sample MR analysis of red wine to hypertension. Table S9: Instrumental variables used in two‐sample MR analysis of hypertension to ICH. Table S10: Instrumental variables used in MVMR analysis of red wine to hypertension. Table S11: Instrumental variables used in MVMR analysis of BMI to hypertension. Table S12: Two‐sample MR analysis result of alcohol to ICH. Table S13: Two‐sample MR analysis result of red wine to ICH. Table S14: Two‐sample MR analysis result of white wine to ICH. Table S15: Multivariable MR analysis result of alcohol and red wine to ICH. Multivariable inverse‐variance weighted method. Table S16: Two‐sample MR analysis result of ICH to red wine. Table S17: Two‐sample MR analysis result of red wine to hypertension. Table S18: Two‐sample MR analysis result of hypertension to ICH. Table S19: Multivariable MR analysis result of BMI and red wine to hypertension. Table S20: STROBE‐MR checklist of recommended items to address in reports of Mendelian randomization studies. [file FSN3-13-e71329-s001.zip › fsn371329-sup-0002-TableS1-S20@supplementary_data4_STROBE_MR_checklist.docx]

| **Tab S20. STROBE-MR checklist of recommended items to address in reports of Mendelian randomization studies^1,2^** | | | | |
| --- | --- | --- | --- | --- |
| **Item No.** | **Section** | **Checklist Item** | **Page No.** | **Relevant text from manuscript** |
| 1 | **TITLE and ABSTRACT** | Indicate Mendelian randomization (MR) as the study's design in the title and/or the abstract if that is a main purpose of the study. | 1 | Red wine may mitigate the risk of intracerebral  hemorrhage by preventing hypertension—a Mendelian randomization study combining CHARLS |
|  | **INTRODUCTION** |  |  |  |
| 2 | **Background** | Explain the scientific background and rationale for the reported study. What is the exposure? Is a potential causal relationship between exposure and outcome plausible? Justify why MR is a helpful method to address the study question. | 2-3 | In summary, there is still a lack of high-quality evidence-based medical evidence regarding whether consuming wine increases or mitigates the risk of ICH. Therefore, it was innovative for this study to employ the Mendelian randomization (MR) method to explore the causal relationship between wine consumption and ICH. |
| 3 | **Objectives** | State specific objectives clearly, including pre-  specified causal hypotheses (if any). State that MR is a method that, under specific assumptions, intends to estimate causal effects. | 3 | The MR approach utilizes specific single nucleotide polymorphisms (SNPs) as instrumental variables to simulate the effect of exposure. |
|  | **METHODS** |  |  |  |
| 4 | **Study design and data sources** | Present key elements of the study design early in the  article. Consider including a table listing sources of data for all phases of the study. For each data source contributing to the analysis, describe the following: |  |  |
|  | a) | Setting: Describe the study design and the underlying  population, if possible. Describe the setting, locations, and relevant dates, including periods of recruitment, exposure, follow-up, and data collection, when available. | 3-4 | Fig 1, Table S1 |
|  | b) | Participants: Give the eligibility criteria, and the  sources and methods of selection of participants. Report the sample size, and whether any power or sample size calculations were carried out prior to the main analysis. | NA | Table S1 |
|  | c) | Describe measurement, quality control and selection of genetic variants. | 5 | SNPs with p-value < 5E-6 and an effect allele  frequency (EAF) between 0.001 and 0.999 were selected (Yu, Li, Li, & Ge, 2023). Next, the F- statistic for each SNP was calculated using the formula R2=2×β2×EAF×(1−EAF) and F=(samplesize−2)×R2/(1−R2), excluding SNPs with F < 10 to obtain strongly associated SNPs (Weng et al., 2023). Linkage disequilibrium (LD)- based clumping was then performed with parameters clump_kb = 10000 and clump_r2 =  0.001 to ensure SNP independence (R package TwoSampleMR version 0.6.3). The candidate instrumental SNPs were merged with outcome GWAS data from the FinnGen database, excluding SNPs not present in the outcome GWAS data and those with a p-value < 5E-6 for their association with the outcome. During the harmonization process, palindromic and incompatible SNPs were removed, and the alleles of the exposure and  outcome SNPs were aligned to obtain the final |
|  | d) | For each exposure, outcome, and other relevant  variables, describe methods of assessment and diagnostic criteria for diseases. | NA |  |
|  | e) | Provide details of ethics committee approval and participant informed consent, if relevant. | 14 | Data used in this article was from databases that  obtained ethical approval a priori. All information was obtained from publicly available databases without sensitive or restrained data involving intelligence, education, social outcomes such as income. |

| 5 | **Assumptions** | Explicitly state the three core IV assumptions for the main analysis (relevance, independence and exclusion restriction) as well assumptions for any additional or sensitivity analysis. | 5 | excluding SNPs with F < 10 to obtain strongly  associated SNPs;  Linkage disequilibrium (LD)-based clumping was then performed with parameters clump_kb = 10000 and clump_r2 = 0.001 to ensure SNP independence;  To further validate the reliability of the two-sample MR analysis results, heterogeneity and pleiotropy  analyses were performed. |
| --- | --- | --- | --- | --- |
| 6 | **Statistical methods: main**  **analysis** | Describe statistical methods and statistics used. |  |  |
|  | a) | Describe how quantitative variables were handled in  the analyses (i.e., scale, units, model). | NA | Table S12-S19 |
|  | b) | Describe how genetic variants were handled in the  analyses and, if applicable, how their weights were selected. | NA | Table S12-S19 |
|  | c) | Describe the MR estimator (e.g. two-stage least  squares, Wald ratio) and related statistics. Detail the included covariates and, in case of two-sample MR, whether the same covariate set was used for adjustment in the two samples. | NA | Table S12-S19 |
|  | d) | Explain how missing data were addressed. | 5 | The candidate instrumental SNPs were merged  with outcome GWAS data from the FinnGen database, excluding SNPs not present in the outcome GWAS data and those with a p-value < 5E-6 for their association with the outcome. |
|  | e) | If applicable, indicate how multiple testing was  addressed. | NA |  |
| 7 | **Assessment of assumptions** | Describe any methods or prior knowledge used to assess the assumptions or justify their validity. | 5-6 | Assumption assessment: Pleiotropy analysis (Q-  Statistic); heterogeneity analysis (I2, Q statistic); MR-PRESSO for outlier exclusion; no reverse causality (reverse two-sample MR). |
| 8 | **Sensitivity analyses and additional analyses** | Describe any sensitivity analyses or additional analyses  performed (e.g. comparison of effect estimates from different approaches, independent replication, bias analytic techniques, validation of instruments, simulations). | 5-6 | Sensitivity analyses: Multiple MR methods (IVW, MR Egger, etc.); outlier exclusion (MR-PRESSO); multivariable MR for confounding control.  Additional analyses: Mediation MR |
| 9 | **Software and preregistration** |  |  |  |
|  | a) | Name statistical software and package(s), including version and settings used. | 5-6 | Software/packages: R; TwoSampleMR (v0.6.3), MendelianRandomization (v0.10.0), MVMR (v0.4) |
|  | b) | State whether the study protocol and details were pre-  registered (as well as when and where). | NA |  |
|  | **RESULTS** |  |  |  |
| 10 | **Descriptive data** |  |  |  |
|  | **a)** | Report the numbers of individuals at each stage of  included studies and reasons for exclusion. Consider use of a flow diagram. | Not relevant |  |
|  | **b)** | Report summary statistics for phenotypic exposure(s),  outcome(s), and other relevant variables (e.g. means, SDs, proportions). | NA |  |
|  | **c)** | If the data sources include meta-analyses of previous  studies, provide the assessments of heterogeneity across these studies. | Not relevant |  |
|  | **d)** | For two-sample MR: |  |  |
|  |  | i. Provide justification of the similarity of the  genetic variant-exposure associations between the exposure and outcome samples. | 2 | From a theoretical perspective, polyphenols in wine  can effectively prevent the aging of vascular endothelial cells. |
|  |  | ii. Provide information on the number of individuals who overlap between the exposure and outcome studies. | Not  relevant (GWAS  data of eposure and outcome from different databases  ) |  |
| 11 | **Main results** |  |  |  |
|  | **a)** | Report the associations between genetic variant and  exposure, and between genetic variant and outcome, preferably on an interpretable scale. | NA | Tab S2-S11 |

|  | **b)** | Report MR estimates of the relationship between  exposure and outcome, and the measures of uncertainty from the MR analysis, on an interpretable scale, such as odds ratio or relative risk per SD difference. | 9, 10, 12 | Fig2-4 |
| --- | --- | --- | --- | --- |
|  | **c)** | If relevant, consider translating estimates of relative  risk into absolute risk for a meaningful time period. | Not  relevant |  |
|  | **d)** | Consider plots to visualize results (e.g. forest plot,  scatterplot of associations between genetic variants and outcome versus between genetic variants and exposure). | 9, 10, 12 | Fig2-4 |
| 12 | **Assessment of assumptions** |  |  |  |
|  | **a)** | Report the assessment of the validity of the assumptions. | 7-8 | Except for one step in the mediation analysis where heterogeneity was present, all other analyses passed the heterogeneity tests, suggesting that the MR results were robust and highly reproducible. |
|  | **b)** | Report any additional statistics (e.g., assessments of  heterogeneity across genetic variants, such as I2, Q statistic or E-value). | NA | Table S12-S19 |
| 13 | **Sensitivity analyses and**  **additional analyses** |  |  |  |
|  | **a)** | Report any sensitivity analyses to assess the robustness of the main results to violations of the assumptions. | 7-8 | Except for one step in the mediation analysis where heterogeneity was present, all other analyses passed the heterogeneity tests, suggesting that the MR results were robust and highly reproducible. |
|  | **b)** | Report results from other sensitivity analyses or  additional analyses. | NA |  |
|  | **c)** | Report any assessment of direction of causal  relationship (e.g., bidirectional MR). | NA |  |
|  | **d)** | When relevant, report and compare with estimates from non-MR analyses. | 1 | Finally, the conclusions were validated in Chinese  population using the CHARLS database and extended the findings. |
|  | **e)** | Consider additional plots to visualize results (e.g.,  leave-one-out analyses). | NA |  |
|  | **DISCUSSION** |  |  |  |
| 14 | **Key results** | Summarize key results with reference to study objectives. | 1 | Alcohol was found to be a risk factor (OR = 1.21,  p-value = 0.0351). Red wine was found to reduce the risk of ICH (OR = 0.61, p-value = 0.0400). The protective effect of red wine was still observed in the multivariable Mendelian randomization analysis (OR = 0.55, p-value = 0.0442). In the mediation analysis, red wine was found to prevent intracerebral hemorrhage by reducing hypertension (mediation = 13.45%, p = 0.0004). In the CHARLS-based analysis, wine consumption was associated with a lower incidence of hypertension  in compared without teetotalers (OR= 0.54, p-value  < 0.0001). |
| 15 | **Limitations** | Discuss limitations of the study, taking into account  the validity of the IV assumptions, other sources of potential bias, and imprecision. Discuss both direction and magnitude of any potential bias and any efforts to address them. | 13-14 |  |
| 16 | **Interpretation** |  |  |  |
|  | **a)** | Meaning: Give a cautious overall interpretation of  results in the context of their limitations and in comparison with other studies. | 2, 13-14 |  |
|  | **b)** | Mechanism: Discuss underlying biological mechanisms that could drive a potential causal relationship between the investigated exposure and the outcome, and whether the gene-environment equivalence assumption is reasonable. Use causal language carefully, clarifying that IV estimates may provide causal effects only under certain assumptions. | 2, 7 |  |
|  | **c)** | Clinical relevance: Discuss whether the results have clinical or public policy relevance, and to what extent they inform effect sizes of possible interventions. | NA |  |

| 17 | **Generalizability** | Discuss the generalizability of the study results (a) to other populations, (b) across other exposure periods/timings, and (c) across other levels of exposure. | (a) 1  (b) 3  (c) NA | Finally, the conclusions were validated in Chinese  population using the CHARLS database and extended the findings.  Two-sample MR analyses were conducted on alcohol, red wine, and white wine as three exposure  factors in relation to ICH. |
| --- | --- | --- | --- | --- |
|  | **OTHER INFORMATION** |  |  |  |
| 18 | **Funding** | Describe sources of funding and the role of funders in the present study and, if applicable, sources of funding for the databases and original study or studies on which the present study is based. | 15 | This work was supported by the National Natural  Science Foundation of China (Grant number 82260247, granted to Shengtao Yao) and the Master Scientific Research Start-up Fund of the Affiliated Hospital of Zunyi Medical College (No. 36 [2018], YZ, granted to Fuan Zhang). |
| 19 | **Data and data sharing** | Provide the data used to perform all analyses or report  where and how the data can be accessed, and reference these sources in the article. Provide the statistical code needed to reproduce the results in the article, or report whether the code is publicly accessible and if so, where. | Table S1 | Table S1 |
| 20 | **Conflicts of Interest** | All authors should declare all potential conflicts of  interest. | 15 | All authors declared that they had no competing  financial interests exist. |

This checklist is copyrighted by the Equator Network under the Creative Commons Attribution 3.0 Unported (CC BY 3.0) license.

Skrivankova VW, Richmond RC, Woolf BAR, Yarmolinsky J, Davies NM, Swanson SA, et al. Strengthening the Reporting of Observational Studies

1. in Epidemiology using Mendelian Randomization (STROBE-MR) Statement. JAMA. 2021;under review.

Skrivankova VW, Richmond RC, Woolf BAR, Davies NM, Swanson SA, VanderWeele TJ, et al. Strengthening the Reporting of Observational

1. Studies in Epidemiology using Mendelian Randomisation (STROBE-MR): Explanation and Elaboration. BMJ. 2021;375:n2233.
