# Supplementary material for: Red Wine May Mitigate the Risk of Intracerebral Hemorrhage by Preventing Hypertension—A Mendelian Randomization Study Combining CHARLS
Source: Food Sci Nutr. 2025 Dec 12;13(12):e71329. doi: 10.1002/fsn3.71329 (PMC12701324; doi:10.1002/fsn3.71329)
Supplement: Supplementary file 1 — Table S1: Detailed information for the GWAS datasets used in the study. Table S2: Instrumental variables used in two‐sample MR analysis of alcohol to ICH. Table S3: Instrumental variables used in two‐sample MR analysis of red wine to ICH. Table S4: Instrumental variables used in two‐sample MR analysis of white wine to ICH. Table S5: Instrumental variables used in multivariable MR analysis of alcohol to ICH. Table S6: Instrumental variables used in multivariable MR analysis of red wine to ICH. Table S7: Instrumental variables used in two‐sample MR analysis of ICH to red wine. Table S8: Instrumental variables used in two‐sample MR analysis of red wine to hypertension. Table S9: Instrumental variables used in two‐sample MR analysis of hypertension to ICH. Table S10: Instrumental variables used in MVMR analysis of red wine to hypertension. Table S11: Instrumental variables used in MVMR analysis of BMI to hypertension. Table S12: Two‐sample MR analysis result of alcohol to ICH. Table S13: Two‐sample MR analysis result of red wine to ICH. Table S14: Two‐sample MR analysis result of white wine to ICH. Table S15: Multivariable MR analysis result of alcohol and red wine to ICH. Multivariable inverse‐variance weighted method. Table S16: Two‐sample MR analysis result of ICH to red wine. Table S17: Two‐sample MR analysis result of red wine to hypertension. Table S18: Two‐sample MR analysis result of hypertension to ICH. Table S19: Multivariable MR analysis result of BMI and red wine to hypertension. Table S20: STROBE‐MR checklist of recommended items to address in reports of Mendelian randomization studies. [file FSN3-13-e71329-s001.zip › fsn371329-sup-0003-TableS2-S11@supplementary_data2_instrumental_variables.docx]

**Table S2 Instrumental variables used in two-sample MR analysis of alcohol to ICH.**

|  | **Exposure** | **SNP** | **Effect**  **Allele** | **Other**  **Allele** | **EAF** | **beta.alcohol** | **se.alcohol** | **pval.alcohol** | **beta.ICH** | **se.ICH** | **pval.ICH** |
| --- | --- | --- | --- | --- | --- | --- | --- | --- | --- | --- | --- |
| 1 | alcohol intake frequency | rs1004787 | A | G | 0.532943 | -0.0222262 | 0.00304518 | 2.90E-13 | 0.0336329 | 0.0233432 | 0.14964 |
| 2 | alcohol intake frequency | rs1006231 | T | C | 0.835327 | -0.0195375 | 0.00409397 | 1.80E-06 | 0.00619598 | 0.0340613 | 0.855656 |
| 3 | alcohol intake frequency | rs10188314 | T | C | 0.470852 | -0.0197869 | 0.00303605 | 7.20E-11 | -0.0283108 | 0.0226898 | 0.212129 |
| 4 | alcohol intake frequency | rs1022666 | T | G | 0.862602 | -0.0220879 | 0.00440779 | 5.40E-07 | -0.0072937 | 0.0384716 | 0.849633 |
| 5 | alcohol intake frequency | rs10262713 | C | G | 0.115368 | 0.0237421 | 0.00473231 | 5.20E-07 | 0.00696302 | 0.0304942 | 0.819383 |
| 6 | alcohol intake frequency | rs10777337 | G | A | 0.658336 | 0.01537 | 0.00319724 | 1.50E-06 | -0.0026185 | 0.0250958 | 0.9169 |
| 7 | alcohol intake frequency | rs10792669 | G | A | 0.505254 | 0.0174322 | 0.00304065 | 9.90E-09 | -0.0268228 | 0.0226428 | 0.236173 |
| 8 | alcohol intake frequency | rs10819272 | C | T | 0.42404 | 0.0146013 | 0.00306127 | 1.80E-06 | 0.00139175 | 0.0227794 | 0.951282 |
| 9 | alcohol intake frequency | rs1083347 | A | T | 0.201204 | -0.0174133 | 0.00380627 | 4.80E-06 | 0.0164541 | 0.0269102 | 0.540905 |
| 10 | alcohol intake frequency | rs10835498 | A | G | 0.43093 | 0.0151421 | 0.00306277 | 7.70E-07 | 0.028455 | 0.0226465 | 0.20894 |
| 11 | alcohol intake frequency | rs10847019 | T | G | 0.019069 | 0.0506259 | 0.0110614 | 4.70E-06 | 0.00891364 | 0.0724126 | 0.902032 |
| 12 | alcohol intake frequency | rs10860862 | T | G | 0.180927 | -0.0181444 | 0.00394408 | 4.20E-06 | -0.045759 | 0.026806 | 0.087813 |
| 13 | alcohol intake frequency | rs10893956 | A | T | 0.176945 | -0.0197734 | 0.00396978 | 6.30E-07 | 0.00734126 | 0.0269805 | 0.785548 |
| 14 | alcohol intake frequency | rs11039429 | T | C | 0.454624 | -0.0235595 | 0.00303737 | 8.70E-15 | 0.00394853 | 0.0234399 | 0.866227 |
| 15 | alcohol intake frequency | rs11109118 | C | A | 0.153841 | 0.0198826 | 0.00421084 | 2.30E-06 | 0.0162593 | 0.0288195 | 0.572634 |
| 16 | alcohol intake frequency | rs11176488 | C | T | 0.319532 | -0.0165697 | 0.00325981 | 3.70E-07 | -0.0267466 | 0.0233211 | 0.251429 |
| 17 | alcohol intake frequency | rs11223617 | A | G | 0.206155 | 0.0250908 | 0.00375381 | 2.30E-11 | 0.0462812 | 0.0248575 | 0.062622 |
| 18 | alcohol intake frequency | rs113893318 | T | C | 0.040326 | -0.0371354 | 0.00773019 | 1.60E-06 | 0.00972762 | 0.0534033 | 0.855462 |
| 19 | alcohol intake frequency | rs11575902 | G | T | 0.081202 | 0.0287653 | 0.00557216 | 2.40E-07 | 0.0500089 | 0.0418215 | 0.231785 |
| 20 | alcohol intake frequency | rs11646721 | T | C | 0.075372 | 0.0303207 | 0.00578692 | 1.60E-07 | 0.0559423 | 0.0521918 | 0.283783 |
| 21 | alcohol intake frequency | rs11700855 | G | A | 0.093465 | -0.029795 | 0.00523292 | 1.20E-08 | -0.0557617 | 0.0348577 | 0.109666 |
| 22 | alcohol intake frequency | rs117317480 | A | G | 0.052021 | -0.0326644 | 0.00705173 | 3.60E-06 | -0.0855837 | 0.0601672 | 0.154901 |
| 23 | alcohol intake frequency | rs117386364 | A | G | 0.045412 | -0.0355023 | 0.00756659 | 2.70E-06 | 0.0985777 | 0.0519023 | 0.057525 |
| 24 | alcohol intake frequency | rs11750012 | G | A | 0.859552 | -0.0205374 | 0.00435096 | 2.40E-06 | -0.0248223 | 0.0347106 | 0.474535 |
| 25 | alcohol intake frequency | rs11750777 | A | G | 0.209454 | -0.020493 | 0.00372613 | 3.80E-08 | -0.0128457 | 0.031552 | 0.683913 |
| 26 | alcohol intake frequency | rs11787216 | T | C | 0.369127 | 0.0244162 | 0.00320078 | 2.40E-14 | 0.00411378 | 0.0235864 | 0.861541 |
| 27 | alcohol intake frequency | rs11790082 | G | A | 0.159211 | -0.0201535 | 0.00415476 | 1.20E-06 | 0.0156731 | 0.0353152 | 0.657184 |
| 28 | alcohol intake frequency | rs118185251 | C | T | 0.01891 | 0.0532958 | 0.0115751 | 4.10E-06 | 0.0969463 | 0.214315 | 0.651014 |
| 29 | alcohol intake frequency | rs11940694 | G | A | 0.604193 | -0.0437138 | 0.00311609 | 1.00E-44 | -0.0106154 | 0.0236487 | 0.653519 |
| 30 | alcohol intake frequency | rs12139282 | A | G | 0.085619 | 0.0256751 | 0.00554203 | 3.60E-06 | -0.0154752 | 0.0413037 | 0.707907 |
| 31 | alcohol intake frequency | rs12153855 | C | T | 0.10497 | 0.0294435 | 0.00493484 | 2.40E-09 | -0.0823981 | 0.0485493 | 0.089658 |
| 32 | alcohol intake frequency | rs12203592 | T | C | 0.219623 | 0.0167948 | 0.00360075 | 3.10E-06 | 0.043368 | 0.067592 | 0.521124 |
| 33 | alcohol intake frequency | rs12240850 | A | G | 0.036602 | 0.0376664 | 0.00816511 | 4.00E-06 | 0.115094 | 0.0639983 | 0.072116 |
| 34 | alcohol intake frequency | rs1228589 | A | G | 0.246133 | 0.0210699 | 0.00352806 | 2.30E-09 | -0.004075 | 0.0261873 | 0.87634 |
| 35 | alcohol intake frequency | rs1229984 | C | T | 0.97277 | -0.261708 | 0.00918496 | 1.40E-178 | -0.279883 | 0.162525 | 0.085053 |

| 36 | alcohol intake frequency | rs12436192 | A | G | 0.175635 | 0.0182212 | 0.00397745 | 4.60E-06 | 0.0151422 | 0.0307856 | 0.622818 |
| --- | --- | --- | --- | --- | --- | --- | --- | --- | --- | --- | --- |
| 37 | alcohol intake frequency | rs12467407 | T | C | 0.469336 | 0.0140988 | 0.00304233 | 3.60E-06 | 0.0264565 | 0.0227741 | 0.245362 |
| 38 | alcohol intake frequency | rs12598321 | T | C | 0.560562 | 0.0157984 | 0.00305053 | 2.20E-07 | 0.0148834 | 0.0231011 | 0.519399 |
| 39 | alcohol intake frequency | rs12692604 | C | G | 0.235079 | 0.0192871 | 0.00356168 | 6.10E-08 | 0.00601195 | 0.0275213 | 0.827081 |
| 40 | alcohol intake frequency | rs12740811 | G | A | 0.094206 | 0.0275161 | 0.0051856 | 1.10E-07 | 0.0963496 | 0.0411282 | 0.019147 |
| 41 | alcohol intake frequency | rs12756299 | C | T | 0.491466 | 0.0155155 | 0.00303175 | 3.10E-07 | 0.00061592 | 0.0226334 | 0.97829 |
| 42 | alcohol intake frequency | rs12784183 | T | G | 0.142543 | 0.0226344 | 0.00435682 | 2.00E-07 | -0.0012182 | 0.0350172 | 0.972249 |
| 43 | alcohol intake frequency | rs12814101 | G | C | 0.583768 | -0.0147415 | 0.00307588 | 1.60E-06 | 0.0070312 | 0.022956 | 0.759383 |
| 44 | alcohol intake frequency | rs13024996 | A | C | 0.36949 | 0.0161091 | 0.00313217 | 2.70E-07 | -0.0081871 | 0.0241246 | 0.734333 |
| 45 | alcohol intake frequency | rs13102973 | C | T | 0.61881 | -0.0194072 | 0.00311875 | 4.90E-10 | 0.0482853 | 0.0227723 | 0.033977 |
| 46 | alcohol intake frequency | rs13120366 | C | T | 0.505676 | 0.0138531 | 0.00302513 | 4.70E-06 | 0.00916856 | 0.0225904 | 0.684845 |
| 47 | alcohol intake frequency | rs13135092 | G | A | 0.083483 | 0.0438341 | 0.00549884 | 1.60E-15 | -0.0946123 | 0.0868482 | 0.275978 |
| 48 | alcohol intake frequency | rs13153216 | G | A | 0.024769 | -0.0471618 | 0.00972292 | 1.20E-06 | -0.0782473 | 0.0690689 | 0.257262 |
| 49 | alcohol intake frequency | rs13178443 | T | C | 0.276349 | -0.0186516 | 0.00338983 | 3.80E-08 | 0.0561715 | 0.0245841 | 0.02232 |
| 50 | alcohol intake frequency | rs13248791 | C | T | 0.445499 | 0.0146128 | 0.0031032 | 2.50E-06 | -0.0026636 | 0.0228002 | 0.907002 |
| 51 | alcohol intake frequency | rs13378810 | G | C | 0.121302 | 0.023357 | 0.00465569 | 5.30E-07 | 0.0121715 | 0.0379795 | 0.748609 |
| 52 | alcohol intake frequency | rs13390019 | C | T | 0.134041 | 0.0296116 | 0.00449182 | 4.30E-11 | 0.0270619 | 0.0469905 | 0.564682 |
| 53 | alcohol intake frequency | rs13413257 | T | C | 0.012809 | 0.063348 | 0.0135284 | 2.80E-06 | -0.0132284 | 0.084261 | 0.87525 |
| 54 | alcohol intake frequency | rs1377491 | T | A | 0.793633 | -0.020206 | 0.00373905 | 6.50E-08 | -0.0006078 | 0.0305398 | 0.984123 |
| 55 | alcohol intake frequency | rs1421085 | C | T | 0.403447 | 0.0199392 | 0.00308481 | 1.00E-10 | 0.00688791 | 0.022883 | 0.76341 |
| 56 | alcohol intake frequency | rs1453027 | G | T | 0.537406 | -0.0158329 | 0.00304412 | 2.00E-07 | -0.0498967 | 0.0227254 | 0.028118 |
| 57 | alcohol intake frequency | rs145898511 | G | T | 0.025639 | 0.049037 | 0.00970964 | 4.40E-07 | -0.0104895 | 0.0672007 | 0.87596 |
| 58 | alcohol intake frequency | rs1468967 | A | G | 0.664658 | 0.0174031 | 0.00321851 | 6.40E-08 | -0.0249441 | 0.0230291 | 0.278739 |
| 59 | alcohol intake frequency | rs1488389 | G | C | 0.880848 | -0.0222125 | 0.00483721 | 4.40E-06 | -0.026962 | 0.0389952 | 0.489301 |
| 60 | alcohol intake frequency | rs1490492 | T | C | 0.663774 | 0.0159267 | 0.00321397 | 7.20E-07 | 0.022619 | 0.0252647 | 0.370636 |
| 61 | alcohol intake frequency | rs1515108 | T | C | 0.636644 | 0.0163891 | 0.0031438 | 1.90E-07 | 0.00709603 | 0.0232465 | 0.760174 |
| 62 | alcohol intake frequency | rs1515590 | T | C | 0.383245 | 0.0182446 | 0.00311623 | 4.80E-09 | -0.0188095 | 0.0227675 | 0.408715 |
| 63 | alcohol intake frequency | rs1558426 | C | T | 0.416222 | -0.0153937 | 0.00307925 | 5.80E-07 | 0.0172712 | 0.0236964 | 0.466092 |
| 64 | alcohol intake frequency | rs1569777 | T | C | 0.103005 | -0.0263391 | 0.00499045 | 1.30E-07 | -0.0443269 | 0.0307774 | 0.149799 |
| 65 | alcohol intake frequency | rs1666658 | C | T | 0.392206 | 0.0179674 | 0.00309866 | 6.70E-09 | -0.0240455 | 0.0232223 | 0.30046 |
| 66 | alcohol intake frequency | rs17127898 | A | T | 0.087459 | 0.0253574 | 0.00536282 | 2.30E-06 | 0.0814264 | 0.036957 | 0.027575 |
| 67 | alcohol intake frequency | rs17185470 | C | G | 0.166071 | -0.0208495 | 0.00410508 | 3.80E-07 | 0.0480938 | 0.0313182 | 0.124624 |
| 68 | alcohol intake frequency | rs1762777 | A | G | 0.756193 | -0.0163176 | 0.00352252 | 3.60E-06 | -0.0058701 | 0.0297907 | 0.843792 |
| 69 | alcohol intake frequency | rs17648701 | C | T | 0.179368 | 0.0196103 | 0.00398105 | 8.40E-07 | -0.0263218 | 0.0285038 | 0.355774 |
| 70 | alcohol intake frequency | rs17662759 | C | T | 0.089115 | 0.0301348 | 0.00546031 | 3.40E-08 | 0.0235139 | 0.0366156 | 0.520754 |
| 71 | alcohol intake frequency | rs17690703 | T | C | 0.262687 | 0.0250342 | 0.00343021 | 2.90E-13 | 0.0224609 | 0.0350704 | 0.521879 |
| 72 | alcohol intake frequency | rs185799410 | T | G | 0.026145 | 0.0505343 | 0.00963585 | 1.60E-07 | -0.218351 | 0.0815943 | 0.00745 |
| 73 | alcohol intake frequency | rs186347 | T | G | 0.463343 | 0.0179489 | 0.00305071 | 4.00E-09 | 0.001509 | 0.0227736 | 0.94717 |

| 74 | alcohol intake frequency | rs1937522 | G | A | 0.528054 | 0.0168979 | 0.00303207 | 2.50E-08 | -0.0288024 | 0.022633 | 0.203166 |
| --- | --- | --- | --- | --- | --- | --- | --- | --- | --- | --- | --- |
| 75 | alcohol intake frequency | rs1984584 | A | G | 0.435215 | -0.0165658 | 0.00305586 | 5.90E-08 | 0.0171506 | 0.0226298 | 0.448524 |
| 76 | alcohol intake frequency | rs1991083 | T | C | 0.679886 | -0.0223925 | 0.00325813 | 6.30E-12 | 0.00897747 | 0.0261004 | 0.730877 |
| 77 | alcohol intake frequency | rs2007761 | A | G | 0.268571 | -0.0171998 | 0.00346513 | 6.90E-07 | -0.0044497 | 0.0274718 | 0.871326 |
| 78 | alcohol intake frequency | rs2009367 | G | T | 0.302999 | 0.0172975 | 0.00330596 | 1.70E-07 | -0.0173323 | 0.0237619 | 0.465747 |
| 79 | alcohol intake frequency | rs2055581 | A | G | 0.299198 | 0.0166853 | 0.00333229 | 5.50E-07 | 0.0178656 | 0.0278748 | 0.521573 |
| 80 | alcohol intake frequency | rs2160935 | T | C | 0.604293 | -0.018718 | 0.00309117 | 1.40E-09 | -0.0366499 | 0.0236739 | 0.121594 |
| 81 | alcohol intake frequency | rs2224873 | A | T | 0.222857 | 0.017496 | 0.00364695 | 1.60E-06 | -0.0106269 | 0.0302063 | 0.72498 |
| 82 | alcohol intake frequency | rs2238660 | G | A | 0.140155 | 0.0211236 | 0.00438489 | 1.50E-06 | -0.0649638 | 0.0427603 | 0.128698 |
| 83 | alcohol intake frequency | rs2244598 | C | T | 0.605114 | -0.018378 | 0.00311907 | 3.80E-09 | -0.0114401 | 0.0232281 | 0.622358 |
| 84 | alcohol intake frequency | rs2411453 | G | T | 0.597353 | -0.0350793 | 0.00309039 | 7.30E-30 | -0.0235346 | 0.0227039 | 0.299927 |
| 85 | alcohol intake frequency | rs2535911 | T | C | 0.354749 | -0.0188476 | 0.00316849 | 2.70E-09 | -0.0070529 | 0.0228122 | 0.757192 |
| 86 | alcohol intake frequency | rs2586462 | G | A | 0.849235 | -0.021178 | 0.00423879 | 5.80E-07 | 0.00789003 | 0.0335379 | 0.814009 |
| 87 | alcohol intake frequency | rs2622167 | A | G | 0.428653 | -0.0191155 | 0.0030674 | 4.60E-10 | -0.0456116 | 0.0241745 | 0.059193 |
| 88 | alcohol intake frequency | rs262240 | T | C | 0.468553 | -0.017207 | 0.00303483 | 1.40E-08 | -0.0095481 | 0.0232593 | 0.681436 |
| 89 | alcohol intake frequency | rs2717063 | A | C | 0.585731 | -0.0203704 | 0.00308457 | 4.00E-11 | 0.0391215 | 0.0231351 | 0.090839 |
| 90 | alcohol intake frequency | rs28412070 | T | C | 0.348216 | -0.0147198 | 0.00319177 | 4.00E-06 | -0.0060026 | 0.0255791 | 0.814466 |
| 91 | alcohol intake frequency | rs28525613 | A | C | 0.153651 | 0.020456 | 0.00424037 | 1.40E-06 | -0.0188753 | 0.0386921 | 0.625668 |
| 92 | alcohol intake frequency | rs28768122 | C | T | 0.759525 | 0.0207 | 0.00355204 | 5.60E-09 | 0.00488522 | 0.0265923 | 0.854243 |
| 93 | alcohol intake frequency | rs28787109 | A | G | 0.40423 | 0.0178107 | 0.00308461 | 7.70E-09 | -0.0363619 | 0.0264298 | 0.168887 |
| 94 | alcohol intake frequency | rs2914860 | T | C | 0.331287 | 0.0150435 | 0.00322706 | 3.10E-06 | -0.0344913 | 0.0242203 | 0.154427 |
| 95 | alcohol intake frequency | rs2924321 | A | G | 0.539592 | -0.0195131 | 0.00305022 | 1.60E-10 | -0.0614922 | 0.0233684 | 0.008503 |
| 96 | alcohol intake frequency | rs2944776 | T | C | 0.060938 | -0.0301991 | 0.00635738 | 2.00E-06 | -0.0504678 | 0.0414167 | 0.22302 |
| 97 | alcohol intake frequency | rs2962193 | G | A | 0.764081 | -0.0164188 | 0.00356748 | 4.20E-06 | -0.0136382 | 0.0307538 | 0.657431 |
| 98 | alcohol intake frequency | rs322776 | G | T | 0.579807 | -0.0149026 | 0.00307764 | 1.30E-06 | -0.008278 | 0.0226587 | 0.714862 |
| 99 | alcohol intake frequency | rs324012 | T | C | 0.448569 | -0.0177932 | 0.00304357 | 5.00E-09 | -0.0090025 | 0.0227222 | 0.691958 |
| 100 | alcohol intake frequency | rs34440851 | T | C | 0.157151 | -0.0226831 | 0.00415058 | 4.60E-08 | -0.0078469 | 0.0268722 | 0.770279 |
| 101 | alcohol intake frequency | rs34473884 | A | G | 0.24819 | -0.0203615 | 0.00350346 | 6.20E-09 | 0.00505552 | 0.026915 | 0.851008 |
| 102 | alcohol intake frequency | rs34631026 | T | C | 0.446061 | -0.0169128 | 0.00304832 | 2.90E-08 | -0.0105175 | 0.0239486 | 0.660538 |
| 103 | alcohol intake frequency | rs34805485 | A | G | 0.013825 | -0.0656773 | 0.0130683 | 5.00E-07 | -0.125836 | 0.12033 | 0.295671 |
| 104 | alcohol intake frequency | rs34811474 | A | G | 0.230728 | -0.0201809 | 0.00359305 | 1.90E-08 | -0.0353556 | 0.0269238 | 0.189124 |
| 105 | alcohol intake frequency | rs35005436 | C | T | 0.158469 | 0.022003 | 0.00416019 | 1.20E-07 | 0.011989 | 0.0355517 | 0.735946 |
| 106 | alcohol intake frequency | rs35073053 | C | G | 0.202212 | -0.0188746 | 0.00380064 | 6.80E-07 | 0.0567026 | 0.0307698 | 0.065358 |
| 107 | alcohol intake frequency | rs35105141 | T | C | 0.401541 | 0.026345 | 0.00308788 | 1.40E-17 | -0.002401 | 0.0234003 | 0.918278 |
| 108 | alcohol intake frequency | rs35589108 | G | A | 0.017435 | -0.0548536 | 0.011569 | 2.10E-06 | -0.0537296 | 0.0896386 | 0.548905 |
| 109 | alcohol intake frequency | rs362307 | T | C | 0.074582 | 0.0433047 | 0.00580219 | 8.40E-14 | 0.0649275 | 0.0450681 | 0.149683 |
| 110 | alcohol intake frequency | rs366684 | C | G | 0.740985 | 0.016095 | 0.00346215 | 3.30E-06 | -0.0211181 | 0.025222 | 0.402431 |
| 111 | alcohol intake frequency | rs3857984 | G | A | 0.415095 | 0.0167169 | 0.00309803 | 6.80E-08 | -0.0101057 | 0.0227033 | 0.656231 |

| 112 | alcohol intake frequency | rs3898475 | C | T | 0.179239 | 0.018608 | 0.00398431 | 3.00E-06 | 0.00242294 | 0.0313197 | 0.938336 |
| --- | --- | --- | --- | --- | --- | --- | --- | --- | --- | --- | --- |
| 113 | alcohol intake frequency | rs3914188 | C | G | 0.733732 | -0.0161592 | 0.00342422 | 2.40E-06 | 0.0431365 | 0.0241513 | 0.074083 |
| 114 | alcohol intake frequency | rs4057919 | T | C | 0.286047 | 0.0169869 | 0.0033557 | 4.10E-07 | -0.0196006 | 0.0262794 | 0.455756 |
| 115 | alcohol intake frequency | rs4135294 | A | G | 0.148588 | -0.022654 | 0.00430689 | 1.40E-07 | -0.0019856 | 0.0335212 | 0.952767 |
| 116 | alcohol intake frequency | rs4241258 | T | C | 0.13763 | 0.0250636 | 0.00440325 | 1.30E-08 | 0.0741324 | 0.0376403 | 0.048897 |
| 117 | alcohol intake frequency | rs4242715 | A | G | 0.680585 | -0.0186543 | 0.00324831 | 9.30E-09 | 0.0322088 | 0.0229918 | 0.161249 |
| 118 | alcohol intake frequency | rs427534 | G | A | 0.412197 | -0.0156854 | 0.00309025 | 3.90E-07 | -0.0078273 | 0.0226146 | 0.729253 |
| 119 | alcohol intake frequency | rs4417025 | A | G | 0.361153 | -0.0188379 | 0.00316516 | 2.70E-09 | -0.0593371 | 0.0269354 | 0.027599 |
| 120 | alcohol intake frequency | rs4500930 | T | C | 0.34327 | 0.0158278 | 0.00318187 | 6.50E-07 | 0.0544387 | 0.0243421 | 0.025326 |
| 121 | alcohol intake frequency | rs4503294 | T | C | 0.565333 | 0.0181476 | 0.00307049 | 3.40E-09 | -0.0235146 | 0.0228413 | 0.303254 |
| 122 | alcohol intake frequency | rs4532588 | C | T | 0.465379 | -0.0147811 | 0.00303752 | 1.10E-06 | 0.00295666 | 0.0230742 | 0.89804 |
| 123 | alcohol intake frequency | rs461599 | C | A | 0.462259 | -0.0191888 | 0.00303977 | 2.70E-10 | -0.0059138 | 0.022591 | 0.793493 |
| 124 | alcohol intake frequency | rs4726481 | T | G | 0.400576 | 0.0217614 | 0.00310188 | 2.30E-12 | 0.044554 | 0.0238323 | 0.061556 |
| 125 | alcohol intake frequency | rs4728701 | G | A | 0.953011 | 0.0328505 | 0.00715273 | 4.40E-06 | -0.0459787 | 0.0488621 | 0.346711 |
| 126 | alcohol intake frequency | rs4739105 | C | T | 0.787783 | -0.019804 | 0.00374496 | 1.20E-07 | 0.0257942 | 0.0281095 | 0.35881 |
| 127 | alcohol intake frequency | rs4742659 | G | A | 0.246749 | 0.0166303 | 0.00350794 | 2.10E-06 | 0.00912658 | 0.0261555 | 0.727138 |
| 128 | alcohol intake frequency | rs4757589 | C | T | 0.466442 | -0.0160551 | 0.00303756 | 1.30E-07 | -0.0121235 | 0.0227389 | 0.593921 |
| 129 | alcohol intake frequency | rs4800487 | G | A | 0.456873 | -0.0289372 | 0.00304694 | 2.20E-21 | -0.0306371 | 0.0226353 | 0.175893 |
| 130 | alcohol intake frequency | rs4811031 | G | A | 0.348533 | 0.0146 | 0.00317873 | 4.40E-06 | 0.0547366 | 0.023324 | 0.018936 |
| 131 | alcohol intake frequency | rs4815366 | T | G | 0.639069 | -0.0159884 | 0.00315907 | 4.20E-07 | -0.0143018 | 0.0245498 | 0.560188 |
| 132 | alcohol intake frequency | rs4865166 | T | C | 0.276936 | 0.017361 | 0.00338473 | 2.90E-07 | -0.0473867 | 0.0274447 | 0.084235 |
| 133 | alcohol intake frequency | rs489062 | A | G | 0.437454 | 0.0166498 | 0.00305291 | 4.90E-08 | 0.0245083 | 0.0227978 | 0.282362 |
| 134 | alcohol intake frequency | rs4916723 | C | A | 0.420617 | 0.0239479 | 0.00309951 | 1.10E-14 | -0.0029753 | 0.0226358 | 0.895426 |
| 135 | alcohol intake frequency | rs4940926 | C | T | 0.735045 | -0.0191003 | 0.00344068 | 2.80E-08 | 0.0199488 | 0.0285486 | 0.484698 |
| 136 | alcohol intake frequency | rs4968391 | T | G | 0.674892 | -0.0192695 | 0.0032265 | 2.30E-09 | -0.0183514 | 0.0231812 | 0.428563 |
| 137 | alcohol intake frequency | rs4982052 | G | A | 0.34022 | -0.0162733 | 0.00320134 | 3.70E-07 | 0.0352896 | 0.0236763 | 0.136093 |
| 138 | alcohol intake frequency | rs5022348 | T | C | 0.40703 | 0.0202641 | 0.00357005 | 1.40E-08 | 0.0314827 | 0.0226888 | 0.165262 |
| 139 | alcohol intake frequency | rs533143 | C | T | 0.288593 | -0.0180506 | 0.00338932 | 1.00E-07 | 0.00748736 | 0.0306351 | 0.806918 |
| 140 | alcohol intake frequency | rs550942 | T | C | 0.823865 | 0.022401 | 0.00398884 | 2.00E-08 | -0.0258354 | 0.0378202 | 0.494536 |
| 141 | alcohol intake frequency | rs551998 | C | T | 0.366895 | -0.0156552 | 0.00314936 | 6.70E-07 | 0.00537583 | 0.02293 | 0.814639 |
| 142 | alcohol intake frequency | rs56149652 | G | A | 0.24756 | -0.0186206 | 0.00351342 | 1.20E-07 | -0.021902 | 0.0251478 | 0.383793 |
| 143 | alcohol intake frequency | rs56194430 | T | C | 0.16931 | 0.0225403 | 0.00407148 | 3.10E-08 | -0.0087806 | 0.0343307 | 0.798133 |
| 144 | alcohol intake frequency | rs56228311 | T | G | 0.342458 | -0.0151059 | 0.0031988 | 2.30E-06 | -0.0130673 | 0.0270064 | 0.628485 |
| 145 | alcohol intake frequency | rs56328909 | A | T | 0.306248 | 0.0152981 | 0.00328855 | 3.30E-06 | -0.0341554 | 0.0251306 | 0.174109 |
| 146 | alcohol intake frequency | rs565078 | T | C | 0.485317 | 0.0143649 | 0.00303964 | 2.30E-06 | -0.0252861 | 0.0226985 | 0.265278 |
| 147 | alcohol intake frequency | rs565522 | C | T | 0.429988 | 0.0141086 | 0.00306229 | 4.10E-06 | 0.0198018 | 0.0225791 | 0.380488 |
| 148 | alcohol intake frequency | rs58905411 | A | G | 0.410052 | -0.0266343 | 0.00307848 | 5.10E-18 | 0.00849927 | 0.0227709 | 0.708962 |
| 149 | alcohol intake frequency | rs59188188 | T | G | 0.290363 | 0.0170449 | 0.00334179 | 3.40E-07 | 0.0293951 | 0.024518 | 0.230559 |

| 150 | alcohol intake frequency | rs6030200 | A | G | 0.31415 | -0.019529 | 0.00327075 | 2.40E-09 | -0.0003301 | 0.0229988 | 0.988547 |
| --- | --- | --- | --- | --- | --- | --- | --- | --- | --- | --- | --- |
| 151 | alcohol intake frequency | rs6079439 | G | A | 0.527488 | -0.0151677 | 0.00303993 | 6.10E-07 | -0.0044978 | 0.0227286 | 0.843131 |
| 152 | alcohol intake frequency | rs61825452 | T | A | 0.192752 | 0.0180098 | 0.00388612 | 3.60E-06 | -0.014201 | 0.0291549 | 0.626196 |
| 153 | alcohol intake frequency | rs61873510 | T | G | 0.32785 | 0.0203737 | 0.00330311 | 6.90E-10 | 0.00044737 | 0.0248361 | 0.985629 |
| 154 | alcohol intake frequency | rs62135521 | T | G | 0.048482 | 0.0344792 | 0.00703689 | 9.60E-07 | 0.0817264 | 0.054859 | 0.136289 |
| 155 | alcohol intake frequency | rs62182135 | A | C | 0.330638 | -0.0150592 | 0.00321408 | 2.80E-06 | 0.01944 | 0.0247126 | 0.43149 |
| 156 | alcohol intake frequency | rs62271373 | A | T | 0.060006 | 0.0334009 | 0.006494 | 2.70E-07 | 0.0683639 | 0.0555359 | 0.218328 |
| 157 | alcohol intake frequency | rs62304163 | T | C | 0.042477 | 0.0383203 | 0.00756497 | 4.10E-07 | -0.121934 | 0.0617203 | 0.048201 |
| 158 | alcohol intake frequency | rs62305780 | G | C | 0.102253 | -0.0485216 | 0.00506585 | 9.90E-22 | -0.0163284 | 0.0327775 | 0.618371 |
| 159 | alcohol intake frequency | rs62339673 | A | C | 0.626705 | 0.0182943 | 0.00315406 | 6.60E-09 | -0.0058175 | 0.0238199 | 0.807054 |
| 160 | alcohol intake frequency | rs62439319 | T | C | 0.140966 | 0.0213766 | 0.0043477 | 8.80E-07 | 0.0116606 | 0.0338513 | 0.730496 |
| 161 | alcohol intake frequency | rs62466318 | T | C | 0.202827 | -0.0254919 | 0.0037742 | 1.40E-11 | 0.0169523 | 0.0287966 | 0.556068 |
| 162 | alcohol intake frequency | rs6427160 | C | T | 0.421605 | 0.0143061 | 0.00306334 | 3.00E-06 | 0.00324104 | 0.0227547 | 0.886737 |
| 163 | alcohol intake frequency | rs650558 | T | C | 0.247918 | 0.0207362 | 0.0035079 | 3.40E-09 | 0.0142324 | 0.0276215 | 0.606366 |
| 164 | alcohol intake frequency | rs6531648 | C | T | 0.517258 | 0.0153231 | 0.00302708 | 4.10E-07 | -0.0329961 | 0.023154 | 0.154136 |
| 165 | alcohol intake frequency | rs6534293 | G | A | 0.556934 | 0.0140304 | 0.00306044 | 4.60E-06 | -0.0116389 | 0.0227104 | 0.608307 |
| 166 | alcohol intake frequency | rs6699175 | T | C | 0.109501 | 0.0254827 | 0.00491174 | 2.10E-07 | -0.0447667 | 0.0366993 | 0.222532 |
| 167 | alcohol intake frequency | rs6700839 | A | G | 0.567975 | 0.0147389 | 0.00306802 | 1.60E-06 | 0.0463758 | 0.0229 | 0.042852 |
| 168 | alcohol intake frequency | rs6727281 | T | C | 0.184023 | -0.024322 | 0.00391964 | 5.50E-10 | 0.0404027 | 0.0258477 | 0.118028 |
| 169 | alcohol intake frequency | rs6744640 | G | A | 0.436123 | 0.015141 | 0.00305915 | 7.40E-07 | -0.0296169 | 0.0247124 | 0.230736 |
| 170 | alcohol intake frequency | rs67822265 | T | C | 0.380951 | 0.0147902 | 0.00312904 | 2.30E-06 | -0.0592622 | 0.0237159 | 0.01246 |
| 171 | alcohol intake frequency | rs68024891 | C | T | 0.232972 | -0.0179697 | 0.00360018 | 6.00E-07 | 0.017053 | 0.0269845 | 0.527416 |
| 172 | alcohol intake frequency | rs6810396 | G | C | 0.195181 | -0.0202611 | 0.00382656 | 1.20E-07 | -0.0037863 | 0.02503 | 0.879761 |
| 173 | alcohol intake frequency | rs6819372 | G | A | 0.533037 | -0.0163435 | 0.00303392 | 7.20E-08 | -0.0495593 | 0.0227944 | 0.029691 |
| 174 | alcohol intake frequency | rs693458 | C | A | 0.265691 | 0.018623 | 0.00342728 | 5.50E-08 | 0.0381868 | 0.0293511 | 0.193247 |
| 175 | alcohol intake frequency | rs6992604 | C | T | 0.290386 | 0.0165551 | 0.00335128 | 7.80E-07 | -0.00358 | 0.0244465 | 0.883572 |
| 176 | alcohol intake frequency | rs6993770 | T | A | 0.287365 | -0.0165469 | 0.00334122 | 7.30E-07 | 0.0255879 | 0.0272525 | 0.347772 |
| 177 | alcohol intake frequency | rs7029768 | T | G | 0.128925 | -0.0233267 | 0.00452161 | 2.50E-07 | 0.0159432 | 0.0349405 | 0.648176 |
| 178 | alcohol intake frequency | rs7046881 | G | T | 0.423226 | -0.014238 | 0.00307135 | 3.60E-06 | 0.00255594 | 0.025573 | 0.920387 |
| 179 | alcohol intake frequency | rs71651683 | T | C | 0.0142 | -0.0704589 | 0.0127906 | 3.60E-08 | -0.252724 | 0.135061 | 0.06132 |
| 180 | alcohol intake frequency | rs72641050 | C | A | 0.247115 | 0.0170168 | 0.00352184 | 1.40E-06 | -0.0071489 | 0.0240957 | 0.766706 |
| 181 | alcohol intake frequency | rs72642323 | G | T | 0.008282 | 0.0885015 | 0.016743 | 1.30E-07 | 0.15774 | 0.26794 | 0.556054 |
| 182 | alcohol intake frequency | rs72769229 | T | A | 0.154942 | -0.0231352 | 0.00419154 | 3.40E-08 | 0.0185521 | 0.0349601 | 0.595651 |
| 183 | alcohol intake frequency | rs72787062 | A | G | 0.162767 | -0.0281947 | 0.00410324 | 6.40E-12 | -0.0258147 | 0.0319101 | 0.418527 |
| 184 | alcohol intake frequency | rs728538 | G | T | 0.168868 | 0.0228752 | 0.00406259 | 1.80E-08 | 0.0395208 | 0.028947 | 0.172165 |
| 185 | alcohol intake frequency | rs7298932 | G | A | 0.147849 | -0.0237214 | 0.00431166 | 3.80E-08 | 0.0206018 | 0.0347792 | 0.55361 |
| 186 | alcohol intake frequency | rs7302200 | A | G | 0.339998 | -0.0184222 | 0.00319841 | 8.40E-09 | -0.018898 | 0.0247451 | 0.445044 |
| 187 | alcohol intake frequency | rs73050128 | A | C | 0.164488 | -0.0260048 | 0.00409088 | 2.10E-10 | 0.0230786 | 0.026384 | 0.381727 |

| 188 | alcohol intake frequency | rs73168402 | C | G | 0.268552 | 0.0176647 | 0.00342815 | 2.60E-07 | -0.0442774 | 0.0279782 | 0.11352 |
| --- | --- | --- | --- | --- | --- | --- | --- | --- | --- | --- | --- |
| 189 | alcohol intake frequency | rs7330939 | T | C | 0.720352 | -0.0213301 | 0.0034046 | 3.70E-10 | 0.0176287 | 0.0239395 | 0.461497 |
| 190 | alcohol intake frequency | rs73543290 | A | G | 0.044089 | -0.0356162 | 0.00744089 | 1.70E-06 | -0.102248 | 0.0754584 | 0.175411 |
| 191 | alcohol intake frequency | rs7357754 | G | A | 0.500148 | 0.0149913 | 0.00303636 | 7.90E-07 | 0.013318 | 0.0226272 | 0.55614 |
| 192 | alcohol intake frequency | rs74424378 | G | T | 0.23761 | 0.0176039 | 0.00356049 | 7.60E-07 | 0.00700843 | 0.0256422 | 0.78461 |
| 193 | alcohol intake frequency | rs74439139 | G | T | 0.091282 | 0.0260691 | 0.00540153 | 1.40E-06 | -0.052246 | 0.0510335 | 0.30595 |
| 194 | alcohol intake frequency | rs7460106 | C | T | 0.23562 | -0.0217112 | 0.00361927 | 2.00E-09 | 0.0240849 | 0.0258825 | 0.352087 |
| 195 | alcohol intake frequency | rs74679146 | C | T | 0.074515 | -0.0320735 | 0.00575757 | 2.50E-08 | -0.0222233 | 0.043923 | 0.612884 |
| 196 | alcohol intake frequency | rs74808805 | T | G | 0.043603 | -0.0350455 | 0.0075006 | 3.00E-06 | -0.0870283 | 0.070264 | 0.215497 |
| 197 | alcohol intake frequency | rs7514579 | C | A | 0.232457 | 0.0196672 | 0.00359788 | 4.60E-08 | -0.0516689 | 0.0277706 | 0.062806 |
| 198 | alcohol intake frequency | rs7587791 | C | A | 0.51317 | -0.015383 | 0.00302725 | 3.70E-07 | -0.0148308 | 0.0227979 | 0.515347 |
| 199 | alcohol intake frequency | rs76082653 | T | C | 0.054327 | 0.0464269 | 0.00668673 | 3.80E-12 | 0.0764467 | 0.0645011 | 0.235938 |
| 200 | alcohol intake frequency | rs7610856 | A | C | 0.429053 | -0.023864 | 0.00307022 | 7.70E-15 | 0.00013704 | 0.0232062 | 0.995288 |
| 201 | alcohol intake frequency | rs7616201 | G | A | 0.348507 | -0.0146143 | 0.00318942 | 4.60E-06 | 0.0116921 | 0.0237306 | 0.622224 |
| 202 | alcohol intake frequency | rs767870 | A | G | 0.849798 | -0.0196656 | 0.00423795 | 3.50E-06 | -0.026274 | 0.0340551 | 0.440401 |
| 203 | alcohol intake frequency | rs77371894 | G | C | 0.090781 | 0.0254952 | 0.00528899 | 1.40E-06 | 0.0269152 | 0.0528736 | 0.610718 |
| 204 | alcohol intake frequency | rs780094 | C | T | 0.615206 | -0.0509938 | 0.00310506 | 1.30E-60 | -0.0279857 | 0.0236645 | 0.236967 |
| 205 | alcohol intake frequency | rs780569 | A | T | 0.70882 | 0.0198033 | 0.00336454 | 4.00E-09 | -0.0358402 | 0.0267686 | 0.180607 |
| 206 | alcohol intake frequency | rs7932314 | G | A | 0.71681 | -0.0169114 | 0.00336071 | 4.90E-07 | -0.0485051 | 0.0256522 | 0.058641 |
| 207 | alcohol intake frequency | rs7973103 | T | C | 0.193762 | -0.0202521 | 0.00383338 | 1.30E-07 | -0.0318631 | 0.0379111 | 0.400646 |
| 208 | alcohol intake frequency | rs79942197 | G | C | 0.064046 | -0.0301693 | 0.00621192 | 1.20E-06 | 0.0226945 | 0.064315 | 0.724189 |
| 209 | alcohol intake frequency | rs80101850 | C | T | 0.038332 | 0.0398214 | 0.00796143 | 5.70E-07 | -0.0977829 | 0.0553632 | 0.077361 |
| 210 | alcohol intake frequency | rs8015726 | C | T | 0.362509 | -0.0157641 | 0.00316883 | 6.50E-07 | -0.0012184 | 0.0254575 | 0.961827 |
| 211 | alcohol intake frequency | rs80214940 | G | A | 0.17751 | -0.0184159 | 0.00398593 | 3.80E-06 | 0.0242296 | 0.0297997 | 0.416172 |
| 212 | alcohol intake frequency | rs80255756 | A | G | 0.063277 | 0.0316484 | 0.00623365 | 3.80E-07 | 0.0637283 | 0.0853467 | 0.455245 |
| 213 | alcohol intake frequency | rs80292319 | C | T | 0.057704 | -0.0393728 | 0.00649583 | 1.40E-09 | -0.0026793 | 0.0413441 | 0.94833 |
| 214 | alcohol intake frequency | rs8030809 | A | G | 0.553608 | 0.0164409 | 0.00306751 | 8.30E-08 | 0.0451347 | 0.0229857 | 0.049577 |
| 215 | alcohol intake frequency | rs803223 | A | G | 0.74506 | -0.0172308 | 0.00347132 | 6.90E-07 | -0.0148328 | 0.0228983 | 0.517134 |
| 216 | alcohol intake frequency | rs8043563 | C | G | 0.737192 | 0.0233654 | 0.00347125 | 1.70E-11 | 0.0167797 | 0.0254415 | 0.509549 |
| 217 | alcohol intake frequency | rs8050545 | G | A | 0.112293 | 0.0238016 | 0.00479516 | 6.90E-07 | 0.0241717 | 0.0281981 | 0.39133 |
| 218 | alcohol intake frequency | rs8083110 | T | C | 0.418707 | -0.0168099 | 0.00307792 | 4.70E-08 | 0.0324019 | 0.0226579 | 0.152703 |
| 219 | alcohol intake frequency | rs838145 | A | G | 0.542982 | 0.0219548 | 0.00305549 | 6.70E-13 | -0.0042813 | 0.0237587 | 0.856997 |
| 220 | alcohol intake frequency | rs8614 | A | C | 0.182509 | 0.0247806 | 0.00392537 | 2.70E-10 | -0.0158393 | 0.0314669 | 0.614708 |
| 221 | alcohol intake frequency | rs874296 | C | T | 0.386036 | 0.0159123 | 0.00312504 | 3.50E-07 | 0.00297073 | 0.0230431 | 0.897421 |
| 222 | alcohol intake frequency | rs900802 | T | C | 0.694659 | -0.0169459 | 0.00330306 | 2.90E-07 | -0.0341132 | 0.0251307 | 0.174645 |
| 223 | alcohol intake frequency | rs911475 | T | C | 0.166508 | 0.019878 | 0.0041038 | 1.30E-06 | -0.0148029 | 0.0363634 | 0.683946 |
| 224 | alcohol intake frequency | rs9349379 | G | A | 0.405493 | -0.0193455 | 0.00308215 | 3.50E-10 | -0.0176599 | 0.0226143 | 0.434852 |
| 225 | alcohol intake frequency | rs9350781 | T | A | 0.351505 | 0.0150037 | 0.00318403 | 2.50E-06 | 0.0221849 | 0.023922 | 0.353726 |

| 226 | alcohol intake frequency | rs9372625 | A | G | 0.381706 | -0.0255579 | 0.00312467 | 2.90E-16 | -0.0218925 | 0.0242832 | 0.367297 |
| --- | --- | --- | --- | --- | --- | --- | --- | --- | --- | --- | --- |
| 227 | alcohol intake frequency | rs9403297 | A | G | 0.372967 | 0.0188234 | 0.00313037 | 1.80E-09 | 0.0232451 | 0.0246367 | 0.345419 |
| 228 | alcohol intake frequency | rs9537938 | A | G | 0.682279 | -0.0171193 | 0.00326026 | 1.50E-07 | 0.00080202 | 0.0240428 | 0.973389 |
| 229 | alcohol intake frequency | rs956752 | G | A | 0.100624 | 0.0255759 | 0.00502689 | 3.60E-07 | 0.00621876 | 0.0412838 | 0.880264 |
| 230 | alcohol intake frequency | rs9611953 | G | A | 0.491257 | 0.0158205 | 0.00303095 | 1.80E-07 | -0.0242536 | 0.0233914 | 0.299801 |
| 231 | alcohol intake frequency | rs9648478 | A | G | 0.510245 | 0.0168603 | 0.00302891 | 2.60E-08 | 0.0137287 | 0.0225884 | 0.543337 |
| 232 | alcohol intake frequency | rs9690021 | T | G | 0.491326 | 0.0150599 | 0.00302732 | 6.50E-07 | 0.0325362 | 0.0226643 | 0.151125 |
| 233 | alcohol intake frequency | rs9814516 | T | G | 0.237423 | -0.0251117 | 0.00355589 | 1.60E-12 | 0.0260283 | 0.0274699 | 0.343373 |
| 234 | alcohol intake frequency | rs9829192 | T | G | 0.435133 | 0.0169324 | 0.00305032 | 2.80E-08 | -0.022395 | 0.0227918 | 0.32581 |
| 235 | alcohol intake frequency | rs9891943 | C | T | 0.232109 | 0.0198671 | 0.00364147 | 4.90E-08 | -0.0049735 | 0.02944 | 0.865847 |
| 236 | alcohol intake frequency | rs9902512 | G | C | 0.356406 | -0.0144847 | 0.00316271 | 4.70E-06 | -0.014281 | 0.0241189 | 0.553778 |
| 237 | alcohol intake frequency | rs9906502 | A | G | 0.176998 | 0.0237883 | 0.00396178 | 1.90E-09 | 0.0209678 | 0.028654 | 0.464314 |

Table S3 Instrumental variables used in two-sample MR analysis of red wine to ICH.

|  | **Exposure** | **SNP** | **Effect**  **Allele** | **Other**  **Allele** | **EAF** | **beta.red_wine** | **se.red_wine** | **pval.red_wine** | **beta.ICH** | **se.ICH** | **pval.ICH** |
| --- | --- | --- | --- | --- | --- | --- | --- | --- | --- | --- | --- |
| 1 | average weekly  red wine intake | rs10006551 | T | C | 0.539013 | 0.0105641 | 0.0020678 | 3.20E-07 | 0.0188685 | 0.022871 | 0.409373 |
| 2 | average weekly  red wine intake | rs10156047 | T | G | 0.138048 | -0.0140078 | 0.00300956 | 3.20E-06 | -0.0274901 | 0.0395625 | 0.487148 |
| 3 | average weekly  red wine intake | rs10210652 | A | G | 0.350016 | -0.010268 | 0.00216574 | 2.10E-06 | 0.0658306 | 0.0233259 | 0.00476947 |
| 4 | average weekly  red wine intake | rs10280836 | G | T | 0.771368 | -0.0123121 | 0.00245691 | 5.40E-07 | 0.0280258 | 0.0282984 | 0.321993 |
| 5 | average weekly  red wine intake | rs10822129 | T | C | 0.406521 | 0.01344 | 0.0021284 | 2.70E-10 | -0.0459135 | 0.023004 | 0.0459462 |
| 6 | average weekly  red wine intake | rs10865093 | C | T | 0.447573 | -0.00959194 | 0.00207867 | 3.90E-06 | 0.00579356 | 0.0227902 | 0.799331 |
| 7 | average weekly  red wine intake | rs10873070 | G | A | 0.624045 | -0.00999457 | 0.00215359 | 3.50E-06 | 0.013115 | 0.0242925 | 0.589282 |
| 8 | average weekly  red wine intake | rs10925183 | A | G | 0.607433 | -0.011922 | 0.00211168 | 1.60E-08 | 0.0239068 | 0.0233742 | 0.306408 |
| 9 | average weekly  red wine intake | rs11021354 | G | A | 0.393325 | 0.0112243 | 0.0021204 | 1.20E-07 | 0.0146214 | 0.0256031 | 0.567946 |
| 10 | average weekly  red wine intake | rs11024405 | G | A | 0.185291 | -0.0137073 | 0.00266707 | 2.80E-07 | 0.0346548 | 0.0269934 | 0.199204 |
| 11 | average weekly  red wine intake | rs11114498 | T | C | 0.673867 | -0.0102206 | 0.00220386 | 3.50E-06 | 0.00662552 | 0.0250259 | 0.791205 |
| 12 | average weekly  red wine intake | rs11126576 | T | C | 0.440017 | 0.010927 | 0.00207899 | 1.50E-07 | -0.0135969 | 0.0226232 | 0.547828 |
| 13 | average weekly  red wine intake | rs11221890 | G | A | 0.148265 | 0.0158665 | 0.00291925 | 5.50E-08 | 0.0190092 | 0.0328449 | 0.562753 |
| 14 | average weekly  red wine intake | rs114313565 | A | G | 0.027269 | 0.0320306 | 0.00653599 | 9.60E-07 | -0.106774 | 0.107851 | 0.32217 |
| 15 | average weekly  red wine intake | rs114795493 | A | G | 0.016059 | 0.0393968 | 0.00819179 | 1.50E-06 | -0.0203441 | 0.141144 | 0.885392 |
| 16 | average weekly  red wine intake | rs115189110 | T | C | 0.041798 | 0.0246877 | 0.00532761 | 3.60E-06 | 0.121683 | 0.0440619 | 0.00575135 |
| 17 | average weekly  red wine intake | rs11662234 | G | T | 0.524066 | -0.00945495 | 0.00206748 | 4.80E-06 | 0.0101229 | 0.022876 | 0.658118 |

| 18 | average weekly  red wine intake | rs11705856 | A | G | 0.642632 | 0.01074 | 0.00215133 | 6.00E-07 | 0.0375897 | 0.0231147 | 0.103901 |
| --- | --- | --- | --- | --- | --- | --- | --- | --- | --- | --- | --- |
| 19 | average weekly  red wine intake | rs11714337 | A | G | 0.43305 | 0.0123918 | 0.00208811 | 2.90E-09 | 0.000160106 | 0.023207 | 0.994495 |
| 20 | average weekly  red wine intake | rs11715683 | A | T | 0.344906 | 0.013978 | 0.00217352 | 1.30E-10 | -0.0224253 | 0.023785 | 0.345764 |
| 21 | average weekly  red wine intake | rs117331323 | T | C | 0.025354 | 0.0300717 | 0.00656731 | 4.70E-06 | -0.104592 | 0.130721 | 0.423641 |
| 22 | average weekly  red wine intake | rs11877758 | G | T | 0.310973 | -0.0107631 | 0.00223808 | 1.50E-06 | -0.0391458 | 0.0259396 | 0.13127 |
| 23 | average weekly  red wine intake | rs12030413 | C | G | 0.178642 | 0.012622 | 0.00272179 | 3.50E-06 | 0.0568491 | 0.0307562 | 0.0645476 |
| 24 | average weekly  red wine intake | rs12043569 | G | C | 0.220931 | 0.0129125 | 0.00248759 | 2.10E-07 | -0.0106308 | 0.0264849 | 0.688132 |
| 25 | average weekly  red wine intake | rs1229984 | C | T | 0.976512 | 0.0609318 | 0.00673472 | 1.50E-19 | -0.279883 | 0.162525 | 0.0850531 |
| 26 | average weekly  red wine intake | rs12373827 | A | G | 0.515869 | 0.00957816 | 0.0020731 | 3.80E-06 | 0.0181103 | 0.0226672 | 0.42431 |
| 27 | average weekly  red wine intake | rs12692596 | T | C | 0.370567 | -0.0116782 | 0.0021345 | 4.50E-08 | 0.00561581 | 0.0238362 | 0.813743 |
| 28 | average weekly  red wine intake | rs13034936 | C | T | 0.111522 | -0.0172726 | 0.00327463 | 1.30E-07 | -0.0451505 | 0.0560858 | 0.420804 |
| 29 | average weekly  red wine intake | rs1364603 | G | A | 0.624999 | -0.0105902 | 0.00214104 | 7.60E-07 | -0.00937923 | 0.0239675 | 0.695553 |
| 30 | average weekly  red wine intake | rs1441165 | G | A | 0.534631 | -0.010974 | 0.00207298 | 1.20E-07 | -0.0177434 | 0.0227249 | 0.434926 |
| 31 | average weekly  red wine intake | rs1446577 | G | C | 0.256445 | 0.0151328 | 0.00236106 | 1.50E-10 | -0.0246988 | 0.024681 | 0.316962 |
| 32 | average weekly  red wine intake | rs146821330 | C | T | 0.014265 | -0.0413513 | 0.00873602 | 2.20E-06 | -0.0109905 | 0.148946 | 0.941179 |
| 33 | average weekly  red wine intake | rs164328 | T | G | 0.912282 | 0.0184492 | 0.00367585 | 5.20E-07 | -0.00305474 | 0.049223 | 0.950516 |
| 34 | average weekly  red wine intake | rs16989140 | T | C | 0.088544 | -0.0190561 | 0.00364992 | 1.80E-07 | 0.0523782 | 0.0391935 | 0.181419 |
| 35 | average weekly  red wine intake | rs17817497 | C | T | 0.392483 | 0.014091 | 0.00211408 | 2.60E-11 | 0.00626791 | 0.0230144 | 0.785355 |
| 36 | average weekly  red wine intake | rs1896297 | T | C | 0.655541 | -0.0118387 | 0.00218034 | 5.60E-08 | 0.0183387 | 0.0239394 | 0.443648 |

| 37 | average weekly  red wine intake | rs1916414 | G | A | 0.535336 | 0.0100546 | 0.00207381 | 1.20E-06 | -0.0133746 | 0.0225526 | 0.553153 |
| --- | --- | --- | --- | --- | --- | --- | --- | --- | --- | --- | --- |
| 38 | average weekly  red wine intake | rs1966836 | G | A | 0.710792 | 0.0123253 | 0.00227216 | 5.80E-08 | 0.0281182 | 0.024707 | 0.255093 |
| 39 | average weekly  red wine intake | rs2117137 | G | A | 0.405583 | 0.00983136 | 0.0020973 | 2.80E-06 | 0.0153229 | 0.0226933 | 0.499536 |
| 40 | average weekly  red wine intake | rs211799 | G | A | 0.949547 | -0.0220773 | 0.00476767 | 3.60E-06 | -0.0602499 | 0.0511131 | 0.238495 |
| 41 | average weekly  red wine intake | rs2162277 | T | C | 0.875179 | 0.0146972 | 0.00311722 | 2.40E-06 | 0.0144341 | 0.0373875 | 0.699447 |
| 42 | average weekly  red wine intake | rs2291007 | C | T | 0.570657 | 0.0098601 | 0.00208449 | 2.20E-06 | -0.00416591 | 0.0236268 | 0.860042 |
| 43 | average weekly  red wine intake | rs2383361 | A | G | 0.576022 | -0.00993591 | 0.002093 | 2.10E-06 | 0.045239 | 0.0229183 | 0.0483905 |
| 44 | average weekly  red wine intake | rs2511225 | C | A | 0.353774 | -0.0116801 | 0.00215811 | 6.20E-08 | 0.00957901 | 0.025447 | 0.706597 |
| 45 | average weekly  red wine intake | rs261046 | A | G | 0.245007 | 0.0111114 | 0.00241422 | 4.20E-06 | 0.0384532 | 0.0244477 | 0.115748 |
| 46 | average weekly  red wine intake | rs2789517 | A | G | 0.868394 | 0.0146578 | 0.00307273 | 1.80E-06 | 0.100126 | 0.0505432 | 0.0475905 |
| 47 | average weekly  red wine intake | rs28768122 | C | T | 0.75844 | -0.0125919 | 0.00241565 | 1.90E-07 | 0.00488522 | 0.0265923 | 0.854243 |
| 48 | average weekly  red wine intake | rs300918 | T | C | 0.546776 | -0.00975724 | 0.00208335 | 2.80E-06 | 0.00730537 | 0.0229119 | 0.749844 |
| 49 | average weekly  red wine intake | rs303753 | A | G | 0.344672 | -0.0183206 | 0.0021899 | 6.00E-17 | 0.0244412 | 0.023737 | 0.303167 |
| 50 | average weekly  red wine intake | rs34198201 | A | C | 0.230866 | 0.0116136 | 0.00244813 | 2.10E-06 | -0.0150028 | 0.0290956 | 0.606108 |
| 51 | average weekly  red wine intake | rs34863300 | G | T | 0.30968 | -0.0106628 | 0.00223637 | 1.90E-06 | -2.22E-05 | 0.0240447 | 0.999263 |
| 52 | average weekly  red wine intake | rs35488630 | A | G | 0.177635 | 0.0131037 | 0.00269415 | 1.20E-06 | 0.0196915 | 0.0297045 | 0.507387 |
| 53 | average weekly  red wine intake | rs35698271 | C | A | 0.180339 | -0.0153487 | 0.00269709 | 1.30E-08 | -0.0411077 | 0.0308052 | 0.182059 |
| 54 | average weekly  red wine intake | rs3783297 | C | T | 0.369444 | -0.0109951 | 0.00214288 | 2.90E-07 | 0.0107087 | 0.0236001 | 0.650005 |
| 55 | average weekly  red wine intake | rs3913960 | C | T | 0.62562 | 0.00979277 | 0.00213039 | 4.30E-06 | -0.0168172 | 0.0227891 | 0.460546 |

| 56 | average weekly  red wine intake | rs4316974 | G | C | 0.834771 | -0.0128804 | 0.00278984 | 3.90E-06 | -0.0149036 | 0.0351742 | 0.671778 |
| --- | --- | --- | --- | --- | --- | --- | --- | --- | --- | --- | --- |
| 57 | average weekly  red wine intake | rs4643716 | A | C | 0.583739 | 0.0115308 | 0.00210517 | 4.30E-08 | 0.018002 | 0.0226426 | 0.426584 |
| 58 | average weekly  red wine intake | rs4757370 | A | G | 0.264074 | -0.0109805 | 0.00234018 | 2.70E-06 | 0.0104686 | 0.0244729 | 0.668824 |
| 59 | average weekly  red wine intake | rs4900965 | C | T | 0.30677 | -0.0103348 | 0.00225977 | 4.80E-06 | 0.0354922 | 0.0236904 | 0.13409 |
| 60 | average weekly  red wine intake | rs4953122 | A | G | 0.665297 | -0.0113859 | 0.00218371 | 1.80E-07 | -0.0102754 | 0.0232862 | 0.659021 |
| 61 | average weekly  red wine intake | rs55968191 | A | G | 0.24686 | 0.0132033 | 0.00241641 | 4.70E-08 | 0.0141771 | 0.0278232 | 0.610371 |
| 62 | average weekly  red wine intake | rs56006101 | T | C | 0.019424 | 0.0401881 | 0.00769735 | 1.80E-07 | -0.143078 | 0.071927 | 0.0466778 |
| 63 | average weekly  red wine intake | rs568030 | T | G | 0.742928 | 0.0114655 | 0.0023705 | 1.30E-06 | -0.0416929 | 0.0230582 | 0.0705814 |
| 64 | average weekly  red wine intake | rs60170726 | A | G | 0.142939 | 0.0139683 | 0.00295929 | 2.40E-06 | 0.0667267 | 0.0440508 | 0.129831 |
| 65 | average weekly  red wine intake | rs61958175 | G | A | 0.047829 | 0.024198 | 0.00482569 | 5.30E-07 | -0.0237888 | 0.0609845 | 0.696477 |
| 66 | average weekly  red wine intake | rs62335889 | T | A | 0.267511 | 0.0109489 | 0.00234838 | 3.10E-06 | 0.000446457 | 0.0251097 | 0.985814 |
| 67 | average weekly  red wine intake | rs62371224 | A | T | 0.065498 | 0.0198083 | 0.00427573 | 3.60E-06 | 0.0361688 | 0.0462311 | 0.43401 |
| 68 | average weekly  red wine intake | rs62573521 | T | C | 0.040468 | -0.0323105 | 0.00524921 | 7.50E-10 | 0.13346 | 0.095121 | 0.160599 |
| 69 | average weekly  red wine intake | rs627685 | C | T | 0.302864 | -0.0125352 | 0.00226097 | 3.00E-08 | 0.0268714 | 0.0257046 | 0.295842 |
| 70 | average weekly  red wine intake | rs6442994 | T | C | 0.774678 | -0.012162 | 0.0024891 | 1.00E-06 | 0.0202617 | 0.0259564 | 0.435034 |
| 71 | average weekly  red wine intake | rs6552079 | T | G | 0.557983 | 0.0112661 | 0.00207789 | 5.90E-08 | -0.0549792 | 0.0228156 | 0.0159647 |
| 72 | average weekly  red wine intake | rs6882046 | G | A | 0.268866 | 0.0162857 | 0.00234886 | 4.10E-12 | 0.0091417 | 0.029512 | 0.756742 |
| 73 | average weekly  red wine intake | rs6908328 | A | C | 0.514021 | 0.0127108 | 0.00206267 | 7.20E-10 | 0.0118016 | 0.0236151 | 0.617253 |
| 74 | average weekly  red wine intake | rs6978944 | G | A | 0.202502 | -0.0122005 | 0.00257142 | 2.10E-06 | 0.0239569 | 0.0263869 | 0.363928 |

| 75 | average weekly  red wine intake | rs713598 | G | C | 0.4001 | -0.0173682 | 0.00210277 | 1.50E-16 | 0.0423926 | 0.0237246 | 0.0739605 |
| --- | --- | --- | --- | --- | --- | --- | --- | --- | --- | --- | --- |
| 76 | average weekly  red wine intake | rs7192601 | T | C | 0.343078 | -0.0107415 | 0.00220792 | 1.10E-06 | 0.021081 | 0.0230118 | 0.359616 |
| 77 | average weekly  red wine intake | rs7277942 | T | C | 0.172968 | 0.0126232 | 0.00273123 | 3.80E-06 | -0.0245781 | 0.0303094 | 0.417419 |
| 78 | average weekly  red wine intake | rs73373942 | T | C | 0.148402 | 0.0139803 | 0.00292021 | 1.70E-06 | -0.0642125 | 0.0271621 | 0.0180767 |
| 79 | average weekly  red wine intake | rs7431028 | G | T | 0.525645 | -0.0099514 | 0.00207493 | 1.60E-06 | -0.0112367 | 0.0227373 | 0.621166 |
| 80 | average weekly  red wine intake | rs7433378 | T | C | 0.232435 | 0.0117215 | 0.00248979 | 2.50E-06 | -0.00413364 | 0.0270996 | 0.878765 |
| 81 | average weekly  red wine intake | rs74567946 | G | T | 0.028667 | 0.0312151 | 0.00624833 | 5.90E-07 | -0.0807796 | 0.0713049 | 0.257266 |
| 82 | average weekly  red wine intake | rs7460106 | C | T | 0.236839 | 0.0113766 | 0.00245957 | 3.70E-06 | 0.0240849 | 0.0258825 | 0.352087 |
| 83 | average weekly  red wine intake | rs7546040 | G | C | 0.732216 | 0.0117688 | 0.00232646 | 4.20E-07 | 0.041383 | 0.0259256 | 0.110439 |
| 84 | average weekly  red wine intake | rs76448834 | G | C | 0.054343 | 0.0213995 | 0.00462355 | 3.70E-06 | 0.00750429 | 0.0443424 | 0.865612 |
| 85 | average weekly  red wine intake | rs77061979 | A | G | 0.029752 | 0.0281732 | 0.0061489 | 4.60E-06 | -0.0744748 | 0.0978027 | 0.44637 |
| 86 | average weekly  red wine intake | rs77169096 | A | T | 0.011358 | -0.0509671 | 0.00998914 | 3.40E-07 | 0.0295034 | 0.0790844 | 0.709102 |
| 87 | average weekly  red wine intake | rs77719387 | A | T | 0.017634 | -0.0389537 | 0.00832371 | 2.90E-06 | 0.183402 | 0.106576 | 0.0852786 |
| 88 | average weekly  red wine intake | rs7977455 | G | C | 0.760856 | -0.0116503 | 0.0024228 | 1.50E-06 | -0.0280175 | 0.0258675 | 0.278757 |
| 89 | average weekly  red wine intake | rs898751 | T | C | 0.49202 | -0.012363 | 0.00206424 | 2.10E-09 | 0.0109225 | 0.022663 | 0.62984 |
| 90 | average weekly  red wine intake | rs906580 | T | A | 0.684429 | 0.0102247 | 0.00223068 | 4.60E-06 | 0.0114961 | 0.0238715 | 0.630104 |
| 91 | average weekly  red wine intake | rs927769 | A | C | 0.627128 | 0.0101978 | 0.00213328 | 1.80E-06 | -0.00310665 | 0.0230752 | 0.892903 |
| 92 | average weekly  red wine intake | rs9329343 | G | A | 0.45062 | -0.00952777 | 0.00207725 | 4.50E-06 | 0.0190199 | 0.0227782 | 0.403717 |
| 93 | average weekly  red wine intake | rs9585326 | G | A | 0.53352 | 0.0109987 | 0.00207001 | 1.10E-07 | 0.00350666 | 0.0227588 | 0.877547 |

| 94 | average weekly  red wine intake | rs9728289 | G | A | 0.410572 | -0.00992252 | 0.00209624 | 2.20E-06 | 0.00906541 | 0.0228161 | 0.691129 |
| --- | --- | --- | --- | --- | --- | --- | --- | --- | --- | --- | --- |
| 95 | average weekly  red wine intake | rs9899357 | C | T | 0.381526 | -0.00982063 | 0.0021334 | 4.20E-06 | 0.0529102 | 0.0237016 | 0.0255918 |

Table S4 Instrumental variables used in two-sample MR analysis of white wine to ICH.

|  | **Exposure** | **SNP** | **Effect**  **Allele** | **Other**  **Allele** | **EAF** | **beta.white**  **_wine** | **se.white_**  **wine** | **pval.white_**  **wine** | **beta.ICH** | **se.ICH** | **pval.ICH** |
| --- | --- | --- | --- | --- | --- | --- | --- | --- | --- | --- | --- |
| 1 | average weekly  white wine intake | rs11144705 | G | A | 0.065557 | -0.020739 | 0.004298 | 1.40E-06 | 0.0322511 | 0.0703941 | 0.646845 |
| 2 | average weekly  white wine intake | rs11167136 | A | G | 0.439043 | 0.0110913 | 0.002111 | 1.50E-07 | -0.00817249 | 0.0225844 | 0.717454 |
| 3 | average weekly  white wine intake | rs11206134 | T | C | 0.385913 | -0.011162 | 0.002152 | 2.10E-07 | 0.0340608 | 0.0229645 | 0.138023 |
| 4 | average weekly  white wine intake | rs114071442 | T | C | 0.055475 | 0.0213376 | 0.00459 | 3.30E-06 | 0.0869248 | 0.0526257 | 0.0985848 |
| 5 | average weekly  white wine intake | rs11619768 | A | G | 0.21165 | -0.012085 | 0.002573 | 2.70E-06 | -0.0250408 | 0.0292993 | 0.392741 |
| 6 | average weekly  white wine intake | rs116998060 | T | A | 0.048467 | -0.02287 | 0.004975 | 4.30E-06 | -0.0135947 | 0.0427609 | 0.750544 |
| 7 | average weekly  white wine intake | rs117230177 | A | G | 0.013534 | -0.044554 | 0.009047 | 8.40E-07 | 0.0100359 | 0.162914 | 0.95088 |
| 8 | average weekly  white wine intake | rs11790517 | T | C | 0.263843 | -0.011346 | 0.002383 | 1.90E-06 | 0.021363 | 0.0259904 | 0.4111 |
| 9 | average weekly  white wine intake | rs12131807 | C | G | 0.340031 | -0.011491 | 0.002213 | 2.10E-07 | -0.0162485 | 0.0269139 | 0.546028 |
| 10 | average weekly  white wine intake | rs13017871 | T | C | 0.458352 | 0.0098904 | 0.002097 | 2.40E-06 | 0.029675 | 0.0233853 | 0.204455 |
| 11 | average weekly  white wine intake | rs13029509 | A | G | 0.478256 | -0.012379 | 0.002097 | 3.50E-09 | 0.0414895 | 0.0229174 | 0.070236 |
| 12 | average weekly  white wine intake | rs1391438 | C | T | 0.687344 | -0.01102 | 0.002258 | 1.10E-06 | 0.0120253 | 0.0238433 | 0.614018 |
| 13 | average weekly  white wine intake | rs142700856 | G | C | 0.010393 | 0.049537 | 0.010607 | 3.00E-06 | -0.0668521 | 0.066603 | 0.315504 |
| 14 | average weekly  white wine intake | rs149466208 | T | C | 0.019475 | -0.04073 | 0.007915 | 2.70E-07 | 0.00705073 | 0.0900067 | 0.937561 |
| 15 | average weekly  white wine intake | rs16937623 | C | T | 0.029753 | -0.034461 | 0.006606 | 1.80E-07 | 0.0146778 | 0.0468524 | 0.754071 |
| 16 | average weekly  white wine intake | rs17014308 | A | C | 0.347889 | 0.0104721 | 0.00221 | 2.10E-06 | -0.0349347 | 0.0241721 | 0.148389 |
| 17 | average weekly  white wine intake | rs17231483 | G | A | 0.37612 | 0.0100954 | 0.002177 | 3.50E-06 | -0.0151888 | 0.0234317 | 0.516847 |

| 18 | average weekly  white wine intake | rs174567 | G | A | 0.350388 | 0.0120192 | 0.002193 | 4.20E-08 | 0.0370729 | 0.0228802 | 0.105168 |
| --- | --- | --- | --- | --- | --- | --- | --- | --- | --- | --- | --- |
| 19 | average weekly  white wine intake | rs1870405 | G | A | 0.337067 | 0.0101355 | 0.002211 | 4.60E-06 | -0.00192858 | 0.0236757 | 0.935078 |
| 20 | average weekly  white wine intake | rs2076587 | A | G | 0.342751 | 0.0106838 | 0.002206 | 1.30E-06 | 0.0109987 | 0.0236285 | 0.641585 |
| 21 | average weekly  white wine intake | rs242093 | A | G | 0.569046 | -0.010514 | 0.002129 | 7.90E-07 | 0.0169391 | 0.0230622 | 0.462645 |
| 22 | average weekly  white wine intake | rs2819330 | A | G | 0.288681 | -0.011007 | 0.002311 | 1.90E-06 | 0.0181041 | 0.0266121 | 0.496316 |
| 23 | average weekly  white wine intake | rs2969158 | T | G | 0.648823 | 0.0117197 | 0.002209 | 1.10E-07 | -0.0536805 | 0.023927 | 0.0248639 |
| 24 | average weekly  white wine intake | rs35002974 | A | T | 0.057557 | -0.022423 | 0.004507 | 6.50E-07 | 0.0807881 | 0.0628631 | 0.198742 |
| 25 | average weekly  white wine intake | rs35121390 | G | C | 0.40011 | 0.012443 | 0.002144 | 6.50E-09 | -0.0294464 | 0.0241924 | 0.223538 |
| 26 | average weekly  white wine intake | rs35367661 | A | G | 0.055292 | -0.021946 | 0.004608 | 1.90E-06 | 0.073188 | 0.0578375 | 0.205725 |
| 27 | average weekly  white wine intake | rs35690196 | A | G | 0.457813 | -0.009702 | 0.002101 | 3.90E-06 | 0.0185207 | 0.0228181 | 0.416982 |
| 28 | average weekly  white wine intake | rs35918430 | T | G | 0.08082 | 0.0182801 | 0.003894 | 2.70E-06 | 0.0524605 | 0.042403 | 0.216018 |
| 29 | average weekly  white wine intake | rs3795310 | T | C | 0.449867 | 0.0113839 | 0.0021 | 5.90E-08 | 0.0349386 | 0.0236369 | 0.139371 |
| 30 | average weekly  white wine intake | rs4410947 | A | G | 0.56521 | -0.010015 | 0.002111 | 2.10E-06 | 0.00357778 | 0.0229808 | 0.876281 |
| 31 | average weekly  white wine intake | rs4697060 | G | A | 0.473104 | -0.010733 | 0.002092 | 2.90E-07 | 0.00396394 | 0.0227198 | 0.861495 |
| 32 | average weekly  white wine intake | rs55925421 | A | G | 0.323927 | -0.01139 | 0.002262 | 4.70E-07 | -0.039944 | 0.024406 | 0.101704 |
| 33 | average weekly  white wine intake | rs58969859 | T | C | 0.287755 | -0.011056 | 0.002316 | 1.80E-06 | -0.029513 | 0.0238123 | 0.215197 |
| 34 | average weekly  white wine intake | rs6035621 | C | G | 0.604748 | -0.010648 | 0.002164 | 8.70E-07 | -0.0156165 | 0.0235349 | 0.506981 |
| 35 | average weekly  white wine intake | rs6425280 | G | A | 0.461913 | 0.0099265 | 0.002099 | 2.30E-06 | 0.0374522 | 0.0227186 | 0.0992453 |
| 36 | average weekly  white wine intake | rs6831872 | A | G | 0.090081 | -0.017341 | 0.003672 | 2.30E-06 | 0.0363416 | 0.0387045 | 0.347756 |

| 37 | average weekly  white wine intake | rs6833962 | T | C | 0.087169 | -0.017102 | 0.003707 | 4.00E-06 | -0.0292607 | 0.0496425 | 0.555574 |
| --- | --- | --- | --- | --- | --- | --- | --- | --- | --- | --- | --- |
| 38 | average weekly  white wine intake | rs72801825 | G | A | 0.300703 | 0.0105005 | 0.002283 | 4.20E-06 | 0.0266441 | 0.0258008 | 0.301751 |
| 39 | average weekly  white wine intake | rs73036519 | C | G | 0.299356 | -0.01077 | 0.002297 | 2.80E-06 | -0.0276381 | 0.0240752 | 0.250972 |
| 40 | average weekly  white wine intake | rs7648610 | C | T | 0.305532 | -0.011136 | 0.002278 | 1.00E-06 | 0.0126933 | 0.0247483 | 0.608024 |
| 41 | average weekly  white wine intake | rs7661904 | A | G | 0.205294 | -0.012074 | 0.002612 | 3.80E-06 | 0.0234982 | 0.0286376 | 0.41191 |
| 42 | average weekly  white wine intake | rs76747111 | C | G | 0.037526 | 0.0262964 | 0.005716 | 4.20E-06 | -0.0406847 | 0.0545654 | 0.4559 |
| 43 | average weekly  white wine intake | rs7695773 | C | A | 0.278411 | -0.010776 | 0.00234 | 4.10E-06 | -0.0157168 | 0.0264004 | 0.551628 |
| 44 | average weekly  white wine intake | rs77639683 | G | A | 0.160523 | -0.013539 | 0.002957 | 4.70E-06 | 0.000158499 | 0.0295673 | 0.995723 |
| 45 | average weekly  white wine intake | rs9576190 | A | G | 0.377493 | -0.010146 | 0.002158 | 2.60E-06 | 0.000454217 | 0.0231785 | 0.984365 |
| 46 | average weekly  white wine intake | rs9912530 | C | T | 0.726616 | 0.0133684 | 0.00235 | 1.30E-08 | -0.025612 | 0.0290647 | 0.378207 |

Table S5 Instrumental variables used in multivariable MR analysis of alcohol to ICH.

|  | **Exposure** | **SNP** | **Effect**  **Allele** | **Other**  **Allele** | **EAF** | **beta.alcohol** | **se.alcohol** | **pval.alcohol** | **beta.ICH** | **se.ICH** | **pval.ICH** |
| --- | --- | --- | --- | --- | --- | --- | --- | --- | --- | --- | --- |
| 1 | alcohol intake  frequency | rs1004787 | A | G | 0.532943 | -0.0222262 | 0.00304518 | 2.90E-13 | 0.0336329 | 0.0233432 | 0.14964 |
| 2 | alcohol intake  frequency | rs1006231 | T | C | 0.835327 | -0.0195375 | 0.00409397 | 1.80E-06 | 0.00619598 | 0.0340613 | 0.855656 |
| 3 | alcohol intake  frequency | rs10188314 | T | C | 0.470852 | -0.0197869 | 0.00303605 | 7.20E-11 | -0.0283108 | 0.0226898 | 0.212129 |
| 4 | alcohol intake  frequency | rs10210652 | A | G | 0.349462 | 0.00118568 | 0.00317618 | 0.709999 | 0.0658306 | 0.0233259 | 0.00476947 |
| 5 | alcohol intake  frequency | rs1022666 | T | G | 0.862602 | -0.0220879 | 0.00440779 | 5.40E-07 | -0.00729371 | 0.0384716 | 0.849633 |
| 6 | alcohol intake  frequency | rs10262713 | C | G | 0.115368 | 0.0237421 | 0.00473231 | 5.20E-07 | 0.00696302 | 0.0304942 | 0.819383 |
| 7 | alcohol intake  frequency | rs10280836 | G | T | 0.771416 | 0.00281732 | 0.00360932 | 0.44 | 0.0280258 | 0.0282984 | 0.321993 |
| 8 | alcohol intake  frequency | rs10777337 | G | A | 0.658336 | 0.01537 | 0.00319724 | 1.50E-06 | -0.0026185 | 0.0250958 | 0.9169 |
| 9 | alcohol intake  frequency | rs10792669 | G | A | 0.505254 | 0.0174322 | 0.00304065 | 9.90E-09 | -0.0268228 | 0.0226428 | 0.236173 |
| 10 | alcohol intake  frequency | rs10822129 | T | C | 0.406225 | -0.00672247 | 0.00312256 | 0.0309999 | -0.0459135 | 0.023004 | 0.0459462 |
| 11 | alcohol intake  frequency | rs1083347 | A | T | 0.201204 | -0.0174133 | 0.00380627 | 4.80E-06 | 0.0164541 | 0.0269102 | 0.540905 |
| 12 | alcohol intake  frequency | rs10835498 | A | G | 0.43093 | 0.0151421 | 0.00306277 | 7.70E-07 | 0.028455 | 0.0226465 | 0.20894 |
| 13 | alcohol intake  frequency | rs10847019 | T | G | 0.019069 | 0.0506259 | 0.0110614 | 4.70E-06 | 0.00891364 | 0.0724126 | 0.902032 |
| 14 | alcohol intake  frequency | rs10860862 | T | G | 0.180927 | -0.0181444 | 0.00394408 | 4.20E-06 | -0.045759 | 0.026806 | 0.0878132 |
| 15 | alcohol intake  frequency | rs10865093 | C | T | 0.449058 | 0.0157315 | 0.00304802 | 2.50E-07 | 0.00579356 | 0.0227902 | 0.799331 |
| 16 | alcohol intake  frequency | rs10873070 | G | A | 0.624546 | 0.0051465 | 0.003165 | 0.1 | 0.013115 | 0.0242925 | 0.589282 |
| 17 | alcohol intake  frequency | rs10893956 | A | T | 0.176945 | -0.0197734 | 0.00396978 | 6.30E-07 | 0.00734126 | 0.0269805 | 0.785548 |

| 18 | alcohol intake  frequency | rs10925183 | A | G | 0.607113 | 0.0035952 | 0.00309695 | 0.25 | 0.0239068 | 0.0233742 | 0.306408 |
| --- | --- | --- | --- | --- | --- | --- | --- | --- | --- | --- | --- |
| 19 | alcohol intake  frequency | rs11024405 | G | A | 0.185433 | 0.00715672 | 0.0039144 | 0.0680002 | 0.0346548 | 0.0269934 | 0.199204 |
| 20 | alcohol intake  frequency | rs11039429 | T | C | 0.454624 | -0.0235595 | 0.00303737 | 8.70E-15 | 0.00394853 | 0.0234399 | 0.866227 |
| 21 | alcohol intake  frequency | rs11109118 | C | A | 0.153841 | 0.0198826 | 0.00421084 | 2.30E-06 | 0.0162593 | 0.0288195 | 0.572634 |
| 22 | alcohol intake  frequency | rs11114498 | T | C | 0.674038 | 0.0085743 | 0.00323347 | 0.008 | 0.00662552 | 0.0250259 | 0.791205 |
| 23 | alcohol intake  frequency | rs11176488 | C | T | 0.319532 | -0.0165697 | 0.00325981 | 3.70E-07 | -0.0267466 | 0.0233211 | 0.251429 |
| 24 | alcohol intake  frequency | rs11221890 | G | A | 0.14819 | -0.00727136 | 0.00428998 | 0.0899995 | 0.0190092 | 0.0328449 | 0.562753 |
| 25 | alcohol intake  frequency | rs11223617 | A | G | 0.206155 | 0.0250908 | 0.00375381 | 2.30E-11 | 0.0462812 | 0.0248575 | 0.0626224 |
| 26 | alcohol intake  frequency | rs113893318 | T | C | 0.040326 | -0.0371354 | 0.00773019 | 1.60E-06 | 0.00972762 | 0.0534033 | 0.855462 |
| 27 | alcohol intake  frequency | rs114313565 | A | G | 0.027044 | -0.0267574 | 0.00962599 | 0.00539995 | -0.106774 | 0.107851 | 0.32217 |
| 28 | alcohol intake  frequency | rs114795493 | A | G | 0.016176 | -0.00786735 | 0.0119865 | 0.51 | -0.0203441 | 0.141144 | 0.885392 |
| 29 | alcohol intake  frequency | rs11646721 | T | C | 0.075372 | 0.0303207 | 0.00578692 | 1.60E-07 | 0.0559423 | 0.0521918 | 0.283783 |
| 30 | alcohol intake  frequency | rs11662234 | G | T | 0.525138 | 0.0117595 | 0.00303719 | 0.000109999 | 0.0101229 | 0.022876 | 0.658118 |
| 31 | alcohol intake  frequency | rs11700855 | G | A | 0.093465 | -0.029795 | 0.00523292 | 1.20E-08 | -0.0557617 | 0.0348577 | 0.109666 |
| 32 | alcohol intake  frequency | rs11705856 | A | G | 0.641945 | -0.00846216 | 0.00315794 | 0.00739997 | 0.0375897 | 0.0231147 | 0.103901 |
| 33 | alcohol intake  frequency | rs11715683 | A | T | 0.34352 | -0.0173 | 0.00319423 | 6.10E-08 | -0.0224253 | 0.023785 | 0.345764 |
| 34 | alcohol intake  frequency | rs117317480 | A | G | 0.052021 | -0.0326644 | 0.00705173 | 3.60E-06 | -0.0855837 | 0.0601672 | 0.154901 |
| 35 | alcohol intake  frequency | rs117386364 | A | G | 0.045412 | -0.0355023 | 0.00756659 | 2.70E-06 | 0.0985777 | 0.0519023 | 0.0575254 |
| 36 | alcohol intake  frequency | rs11750012 | G | A | 0.859552 | -0.0205374 | 0.00435096 | 2.40E-06 | -0.0248223 | 0.0347106 | 0.474535 |

| 37 | alcohol intake  frequency | rs11750777 | A | G | 0.209454 | -0.020493 | 0.00372613 | 3.80E-08 | -0.0128457 | 0.031552 | 0.683913 |
| --- | --- | --- | --- | --- | --- | --- | --- | --- | --- | --- | --- |
| 38 | alcohol intake  frequency | rs11787216 | T | C | 0.369127 | 0.0244162 | 0.00320078 | 2.40E-14 | 0.00411378 | 0.0235864 | 0.861541 |
| 39 | alcohol intake  frequency | rs118185251 | C | T | 0.01891 | 0.0532958 | 0.0115751 | 4.10E-06 | 0.0969463 | 0.214315 | 0.651014 |
| 40 | alcohol intake  frequency | rs11877758 | G | T | 0.312274 | 0.00993563 | 0.0032815 | 0.0025 | -0.0391458 | 0.0259396 | 0.13127 |
| 41 | alcohol intake  frequency | rs11940694 | G | A | 0.604193 | -0.0437138 | 0.00311609 | 1.00E-44 | -0.0106154 | 0.0236487 | 0.653519 |
| 42 | alcohol intake  frequency | rs12030413 | C | G | 0.177375 | -0.020387 | 0.00400239 | 3.50E-07 | 0.0568491 | 0.0307562 | 0.0645476 |
| 43 | alcohol intake  frequency | rs12139282 | A | G | 0.085619 | 0.0256751 | 0.00554203 | 3.60E-06 | -0.0154752 | 0.0413037 | 0.707907 |
| 44 | alcohol intake  frequency | rs12203592 | T | C | 0.219623 | 0.0167948 | 0.00360075 | 3.10E-06 | 0.043368 | 0.067592 | 0.521124 |
| 45 | alcohol intake  frequency | rs12240850 | A | G | 0.036602 | 0.0376664 | 0.00816511 | 4.00E-06 | 0.115094 | 0.0639983 | 0.0721157 |
| 46 | alcohol intake  frequency | rs1228589 | A | G | 0.246133 | 0.0210699 | 0.00352806 | 2.30E-09 | -0.00407501 | 0.0261873 | 0.87634 |
| 47 | alcohol intake  frequency | rs1229984 | C | T | 0.97277 | -0.261708 | 0.00918496 | 1.40E-178 | -0.279883 | 0.162525 | 0.0850531 |
| 48 | alcohol intake  frequency | rs12436192 | A | G | 0.175635 | 0.0182212 | 0.00397745 | 4.60E-06 | 0.0151422 | 0.0307856 | 0.622818 |
| 49 | alcohol intake  frequency | rs12467407 | T | C | 0.469336 | 0.0140988 | 0.00304233 | 3.60E-06 | 0.0264565 | 0.0227741 | 0.245362 |
| 50 | alcohol intake  frequency | rs12598321 | T | C | 0.560562 | 0.0157984 | 0.00305053 | 2.20E-07 | 0.0148834 | 0.0231011 | 0.519399 |
| 51 | alcohol intake  frequency | rs12692596 | T | C | 0.371817 | 0.0160275 | 0.00312894 | 3.00E-07 | 0.00561581 | 0.0238362 | 0.813743 |
| 52 | alcohol intake  frequency | rs12740811 | G | A | 0.094206 | 0.0275161 | 0.0051856 | 1.10E-07 | 0.0963496 | 0.0411282 | 0.0191465 |
| 53 | alcohol intake  frequency | rs12756299 | C | T | 0.491466 | 0.0155155 | 0.00303175 | 3.10E-07 | 0.000615922 | 0.0226334 | 0.97829 |
| 54 | alcohol intake  frequency | rs12784183 | T | G | 0.142543 | 0.0226344 | 0.00435682 | 2.00E-07 | -0.00121816 | 0.0350172 | 0.972249 |
| 55 | alcohol intake  frequency | rs12814101 | G | C | 0.583768 | -0.0147415 | 0.00307588 | 1.60E-06 | 0.0070312 | 0.022956 | 0.759383 |

| 56 | alcohol intake  frequency | rs13024996 | A | C | 0.36949 | 0.0161091 | 0.00313217 | 2.70E-07 | -0.00818713 | 0.0241246 | 0.734333 |
| --- | --- | --- | --- | --- | --- | --- | --- | --- | --- | --- | --- |
| 57 | alcohol intake  frequency | rs13034936 | C | T | 0.111781 | 0.00775191 | 0.00480395 | 0.11 | -0.0451505 | 0.0560858 | 0.420804 |
| 58 | alcohol intake  frequency | rs13102973 | C | T | 0.61881 | -0.0194072 | 0.00311875 | 4.90E-10 | 0.0482853 | 0.0227723 | 0.0339766 |
| 59 | alcohol intake  frequency | rs13120366 | C | T | 0.505676 | 0.0138531 | 0.00302513 | 4.70E-06 | 0.00916856 | 0.0225904 | 0.684845 |
| 60 | alcohol intake  frequency | rs13135092 | G | A | 0.083483 | 0.0438341 | 0.00549884 | 1.60E-15 | -0.0946123 | 0.0868482 | 0.275978 |
| 61 | alcohol intake  frequency | rs13153216 | G | A | 0.024769 | -0.0471618 | 0.00972292 | 1.20E-06 | -0.0782473 | 0.0690689 | 0.257262 |
| 62 | alcohol intake  frequency | rs13178443 | T | C | 0.276349 | -0.0186516 | 0.00338983 | 3.80E-08 | 0.0561715 | 0.0245841 | 0.0223203 |
| 63 | alcohol intake  frequency | rs13248791 | C | T | 0.445499 | 0.0146128 | 0.0031032 | 2.50E-06 | -0.00266355 | 0.0228002 | 0.907002 |
| 64 | alcohol intake  frequency | rs13378810 | G | C | 0.121302 | 0.023357 | 0.00465569 | 5.30E-07 | 0.0121715 | 0.0379795 | 0.748609 |
| 65 | alcohol intake  frequency | rs13390019 | C | T | 0.134041 | 0.0296116 | 0.00449182 | 4.30E-11 | 0.0270619 | 0.0469905 | 0.564682 |
| 66 | alcohol intake  frequency | rs13413257 | T | C | 0.012809 | 0.063348 | 0.0135284 | 2.80E-06 | -0.0132284 | 0.084261 | 0.87525 |
| 67 | alcohol intake  frequency | rs1377491 | T | A | 0.793633 | -0.020206 | 0.00373905 | 6.50E-08 | -0.000607758 | 0.0305398 | 0.984123 |
| 68 | alcohol intake  frequency | rs1421085 | C | T | 0.403447 | 0.0199392 | 0.00308481 | 1.00E-10 | 0.00688791 | 0.022883 | 0.76341 |
| 69 | alcohol intake  frequency | rs1446577 | G | C | 0.255629 | -0.00601748 | 0.00346548 | 0.0819993 | -0.0246988 | 0.024681 | 0.316962 |
| 70 | alcohol intake  frequency | rs1453027 | G | T | 0.537406 | -0.0158329 | 0.00304412 | 2.00E-07 | -0.0498967 | 0.0227254 | 0.0281177 |
| 71 | alcohol intake  frequency | rs145898511 | G | T | 0.025639 | 0.049037 | 0.00970964 | 4.40E-07 | -0.0104895 | 0.0672007 | 0.87596 |
| 72 | alcohol intake  frequency | rs146821330 | C | T | 0.014228 | -0.0122258 | 0.0128495 | 0.34 | -0.0109905 | 0.148946 | 0.941179 |
| 73 | alcohol intake  frequency | rs1468967 | A | G | 0.664658 | 0.0174031 | 0.00321851 | 6.40E-08 | -0.0249441 | 0.0230291 | 0.278739 |
| 74 | alcohol intake  frequency | rs1488389 | G | C | 0.880848 | -0.0222125 | 0.00483721 | 4.40E-06 | -0.026962 | 0.0389952 | 0.489301 |

| 75 | alcohol intake  frequency | rs1490492 | T | C | 0.663774 | 0.0159267 | 0.00321397 | 7.20E-07 | 0.022619 | 0.0252647 | 0.370636 |
| --- | --- | --- | --- | --- | --- | --- | --- | --- | --- | --- | --- |
| 76 | alcohol intake  frequency | rs1515108 | T | C | 0.636644 | 0.0163891 | 0.0031438 | 1.90E-07 | 0.00709603 | 0.0232465 | 0.760174 |
| 77 | alcohol intake  frequency | rs1515590 | T | C | 0.383245 | 0.0182446 | 0.00311623 | 4.80E-09 | -0.0188095 | 0.0227675 | 0.408715 |
| 78 | alcohol intake  frequency | rs1558426 | C | T | 0.416222 | -0.0153937 | 0.00307925 | 5.80E-07 | 0.0172712 | 0.0236964 | 0.466092 |
| 79 | alcohol intake  frequency | rs1569777 | T | C | 0.103005 | -0.0263391 | 0.00499045 | 1.30E-07 | -0.0443269 | 0.0307774 | 0.149799 |
| 80 | alcohol intake  frequency | rs164328 | T | G | 0.912543 | -0.00248636 | 0.00541181 | 0.649999 | -0.00305474 | 0.049223 | 0.950516 |
| 81 | alcohol intake  frequency | rs1666658 | C | T | 0.392206 | 0.0179674 | 0.00309866 | 6.70E-09 | -0.0240455 | 0.0232223 | 0.30046 |
| 82 | alcohol intake  frequency | rs16989140 | T | C | 0.089217 | 0.0183773 | 0.00534156 | 0.000580003 | 0.0523782 | 0.0391935 | 0.181419 |
| 83 | alcohol intake  frequency | rs17127898 | A | T | 0.087459 | 0.0253574 | 0.00536282 | 2.30E-06 | 0.0814264 | 0.036957 | 0.0275753 |
| 84 | alcohol intake  frequency | rs17185470 | C | G | 0.166071 | -0.0208495 | 0.00410508 | 3.80E-07 | 0.0480938 | 0.0313182 | 0.124624 |
| 85 | alcohol intake  frequency | rs1762777 | A | G | 0.756193 | -0.0163176 | 0.00352252 | 3.60E-06 | -0.00587012 | 0.0297907 | 0.843792 |
| 86 | alcohol intake  frequency | rs17648701 | C | T | 0.179368 | 0.0196103 | 0.00398105 | 8.40E-07 | -0.0263218 | 0.0285038 | 0.355774 |
| 87 | alcohol intake  frequency | rs17662759 | C | T | 0.089115 | 0.0301348 | 0.00546031 | 3.40E-08 | 0.0235139 | 0.0366156 | 0.520754 |
| 88 | alcohol intake  frequency | rs17690703 | T | C | 0.262687 | 0.0250342 | 0.00343021 | 2.90E-13 | 0.0224609 | 0.0350704 | 0.521879 |
| 89 | alcohol intake  frequency | rs185799410 | T | G | 0.026145 | 0.0505343 | 0.00963585 | 1.60E-07 | -0.218351 | 0.0815943 | 0.00744955 |
| 90 | alcohol intake  frequency | rs186347 | T | G | 0.463343 | 0.0179489 | 0.00305071 | 4.00E-09 | 0.001509 | 0.0227736 | 0.94717 |
| 91 | alcohol intake  frequency | rs1916414 | G | A | 0.535477 | -0.0047532 | 0.00304384 | 0.12 | -0.0133746 | 0.0225526 | 0.553153 |
| 92 | alcohol intake  frequency | rs1937522 | G | A | 0.528054 | 0.0168979 | 0.00303207 | 2.50E-08 | -0.0288024 | 0.022633 | 0.203166 |
| 93 | alcohol intake  frequency | rs1966836 | G | A | 0.709084 | -0.0164217 | 0.00332849 | 8.10E-07 | 0.0281182 | 0.024707 | 0.255093 |

| 94 | alcohol intake  frequency | rs1984584 | A | G | 0.435215 | -0.0165658 | 0.00305586 | 5.90E-08 | 0.0171506 | 0.0226298 | 0.448524 |
| --- | --- | --- | --- | --- | --- | --- | --- | --- | --- | --- | --- |
| 95 | alcohol intake  frequency | rs2007761 | A | G | 0.268571 | -0.0171998 | 0.00346513 | 6.90E-07 | -0.00444972 | 0.0274718 | 0.871326 |
| 96 | alcohol intake  frequency | rs2009367 | G | T | 0.302999 | 0.0172975 | 0.00330596 | 1.70E-07 | -0.0173323 | 0.0237619 | 0.465747 |
| 97 | alcohol intake  frequency | rs2055581 | A | G | 0.299198 | 0.0166853 | 0.00333229 | 5.50E-07 | 0.0178656 | 0.0278748 | 0.521573 |
| 98 | alcohol intake  frequency | rs2117137 | G | A | 0.404997 | -0.00907939 | 0.00308083 | 0.0032 | 0.0153229 | 0.0226933 | 0.499536 |
| 99 | alcohol intake  frequency | rs2160935 | T | C | 0.604293 | -0.018718 | 0.00309117 | 1.40E-09 | -0.0366499 | 0.0236739 | 0.121594 |
| 100 | alcohol intake  frequency | rs2224873 | A | T | 0.222857 | 0.017496 | 0.00364695 | 1.60E-06 | -0.0106269 | 0.0302063 | 0.72498 |
| 101 | alcohol intake  frequency | rs2238660 | G | A | 0.140155 | 0.0211236 | 0.00438489 | 1.50E-06 | -0.0649638 | 0.0427603 | 0.128698 |
| 102 | alcohol intake  frequency | rs2291007 | C | T | 0.56971 | -0.00899351 | 0.00305873 | 0.00329997 | -0.00416591 | 0.0236268 | 0.860042 |
| 103 | alcohol intake  frequency | rs2411453 | G | T | 0.597353 | -0.0350793 | 0.00309039 | 7.30E-30 | -0.0235346 | 0.0227039 | 0.299927 |
| 104 | alcohol intake  frequency | rs2511225 | C | A | 0.35442 | 0.00835922 | 0.00316714 | 0.00830004 | 0.00957901 | 0.025447 | 0.706597 |
| 105 | alcohol intake  frequency | rs2535911 | T | C | 0.354749 | -0.0188476 | 0.00316849 | 2.70E-09 | -0.00705287 | 0.0228122 | 0.757192 |
| 106 | alcohol intake  frequency | rs2586462 | G | A | 0.849235 | -0.021178 | 0.00423879 | 5.80E-07 | 0.00789003 | 0.0335379 | 0.814009 |
| 107 | alcohol intake  frequency | rs261046 | A | G | 0.244483 | -0.00799386 | 0.00354897 | 0.0239999 | 0.0384532 | 0.0244477 | 0.115748 |
| 108 | alcohol intake  frequency | rs2622167 | A | G | 0.428653 | -0.0191155 | 0.0030674 | 4.60E-10 | -0.0456116 | 0.0241745 | 0.059193 |
| 109 | alcohol intake  frequency | rs262240 | T | C | 0.468553 | -0.017207 | 0.00303483 | 1.40E-08 | -0.00954806 | 0.0232593 | 0.681436 |
| 110 | alcohol intake  frequency | rs2717063 | A | C | 0.585731 | -0.0203704 | 0.00308457 | 4.00E-11 | 0.0391215 | 0.0231351 | 0.0908385 |
| 111 | alcohol intake  frequency | rs2789517 | A | G | 0.868002 | -0.0170657 | 0.00450679 | 0.00015 | 0.100126 | 0.0505432 | 0.0475905 |
| 112 | alcohol intake  frequency | rs28412070 | T | C | 0.348216 | -0.0147198 | 0.00319177 | 4.00E-06 | -0.0060026 | 0.0255791 | 0.814466 |

| 113 | alcohol intake  frequency | rs28768122 | C | T | 0.759525 | 0.0207 | 0.00355204 | 5.60E-09 | 0.00488522 | 0.0265923 | 0.854243 |
| --- | --- | --- | --- | --- | --- | --- | --- | --- | --- | --- | --- |
| 114 | alcohol intake  frequency | rs28787109 | A | G | 0.40423 | 0.0178107 | 0.00308461 | 7.70E-09 | -0.0363619 | 0.0264298 | 0.168887 |
| 115 | alcohol intake  frequency | rs2914860 | T | C | 0.331287 | 0.0150435 | 0.00322706 | 3.10E-06 | -0.0344913 | 0.0242203 | 0.154427 |
| 116 | alcohol intake  frequency | rs2944776 | T | C | 0.060938 | -0.0301991 | 0.00635738 | 2.00E-06 | -0.0504678 | 0.0414167 | 0.22302 |
| 117 | alcohol intake  frequency | rs2962193 | G | A | 0.764081 | -0.0164188 | 0.00356748 | 4.20E-06 | -0.0136382 | 0.0307538 | 0.657431 |
| 118 | alcohol intake  frequency | rs303753 | A | G | 0.346398 | 0.0286212 | 0.00320887 | 4.70E-19 | 0.0244412 | 0.023737 | 0.303167 |
| 119 | alcohol intake  frequency | rs322776 | G | T | 0.579807 | -0.0149026 | 0.00307764 | 1.30E-06 | -0.00827798 | 0.0226587 | 0.714862 |
| 120 | alcohol intake  frequency | rs324012 | T | C | 0.448569 | -0.0177932 | 0.00304357 | 5.00E-09 | -0.00900247 | 0.0227222 | 0.691958 |
| 121 | alcohol intake  frequency | rs34440851 | T | C | 0.157151 | -0.0226831 | 0.00415058 | 4.60E-08 | -0.00784692 | 0.0268722 | 0.770279 |
| 122 | alcohol intake  frequency | rs34473884 | A | G | 0.24819 | -0.0203615 | 0.00350346 | 6.20E-09 | 0.00505552 | 0.026915 | 0.851008 |
| 123 | alcohol intake  frequency | rs34631026 | T | C | 0.446061 | -0.0169128 | 0.00304832 | 2.90E-08 | -0.0105175 | 0.0239486 | 0.660538 |
| 124 | alcohol intake  frequency | rs34805485 | A | G | 0.013825 | -0.0656773 | 0.0130683 | 5.00E-07 | -0.125836 | 0.12033 | 0.295671 |
| 125 | alcohol intake  frequency | rs34811474 | A | G | 0.230728 | -0.0201809 | 0.00359305 | 1.90E-08 | -0.0353556 | 0.0269238 | 0.189124 |
| 126 | alcohol intake  frequency | rs35005436 | C | T | 0.158469 | 0.022003 | 0.00416019 | 1.20E-07 | -0.011989 | 0.0355517 | 0.735946 |
| 127 | alcohol intake  frequency | rs35073053 | C | G | 0.202212 | -0.0188746 | 0.00380064 | 6.80E-07 | 0.0567026 | 0.0307698 | 0.0653582 |
| 128 | alcohol intake  frequency | rs35105141 | T | C | 0.401541 | 0.026345 | 0.00308788 | 1.40E-17 | -0.00240095 | 0.0234003 | 0.918278 |
| 129 | alcohol intake  frequency | rs35488630 | A | G | 0.177422 | -0.00674411 | 0.00395878 | 0.0879995 | 0.0196915 | 0.0297045 | 0.507387 |
| 130 | alcohol intake  frequency | rs35589108 | G | A | 0.017435 | -0.0548536 | 0.011569 | 2.10E-06 | -0.0537296 | 0.0896386 | 0.548905 |
| 131 | alcohol intake  frequency | rs362307 | T | C | 0.074582 | 0.0433047 | 0.00580219 | 8.40E-14 | 0.0649275 | 0.0450681 | 0.149683 |

| 132 | alcohol intake  frequency | rs3783297 | C | T | 0.369982 | 0.00874243 | 0.00314711 | 0.00549997 | 0.0107087 | 0.0236001 | 0.650005 |
| --- | --- | --- | --- | --- | --- | --- | --- | --- | --- | --- | --- |
| 133 | alcohol intake  frequency | rs3898475 | C | T | 0.179239 | 0.018608 | 0.00398431 | 3.00E-06 | 0.00242294 | 0.0313197 | 0.938336 |
| 134 | alcohol intake  frequency | rs3913960 | C | T | 0.6261 | 0.00199202 | 0.00312808 | 0.52 | -0.0168172 | 0.0227891 | 0.460546 |
| 135 | alcohol intake  frequency | rs3914188 | C | G | 0.733732 | -0.0161592 | 0.00342422 | 2.40E-06 | 0.0431365 | 0.0241513 | 0.0740832 |
| 136 | alcohol intake  frequency | rs4057919 | T | C | 0.286047 | 0.0169869 | 0.0033557 | 4.10E-07 | -0.0196006 | 0.0262794 | 0.455756 |
| 137 | alcohol intake  frequency | rs4135294 | A | G | 0.148588 | -0.022654 | 0.00430689 | 1.40E-07 | -0.00198555 | 0.0335212 | 0.952767 |
| 138 | alcohol intake  frequency | rs4241258 | T | C | 0.13763 | 0.0250636 | 0.00440325 | 1.30E-08 | 0.0741324 | 0.0376403 | 0.0488968 |
| 139 | alcohol intake  frequency | rs4242715 | A | G | 0.680585 | -0.0186543 | 0.00324831 | 9.30E-09 | 0.0322088 | 0.0229918 | 0.161249 |
| 140 | alcohol intake  frequency | rs427534 | G | A | 0.412197 | -0.0156854 | 0.00309025 | 3.90E-07 | -0.00782732 | 0.0226146 | 0.729253 |
| 141 | alcohol intake  frequency | rs4417025 | A | G | 0.361153 | -0.0188379 | 0.00316516 | 2.70E-09 | -0.0593371 | 0.0269354 | 0.0275988 |
| 142 | alcohol intake  frequency | rs4500930 | T | C | 0.34327 | 0.0158278 | 0.00318187 | 6.50E-07 | 0.0544387 | 0.0243421 | 0.0253256 |
| 143 | alcohol intake  frequency | rs4503294 | T | C | 0.565333 | 0.0181476 | 0.00307049 | 3.40E-09 | -0.0235146 | 0.0228413 | 0.303254 |
| 144 | alcohol intake  frequency | rs4532588 | C | T | 0.465379 | -0.0147811 | 0.00303752 | 1.10E-06 | 0.00295666 | 0.0230742 | 0.89804 |
| 145 | alcohol intake  frequency | rs461599 | C | A | 0.462259 | -0.0191888 | 0.00303977 | 2.70E-10 | -0.00591379 | 0.022591 | 0.793493 |
| 146 | alcohol intake  frequency | rs4643716 | A | C | 0.583181 | -0.00530039 | 0.003091 | 0.0860003 | 0.018002 | 0.0226426 | 0.426584 |
| 147 | alcohol intake  frequency | rs4726481 | T | G | 0.400576 | 0.0217614 | 0.00310188 | 2.30E-12 | 0.044554 | 0.0238323 | 0.0615559 |
| 148 | alcohol intake  frequency | rs4728701 | G | A | 0.953011 | 0.0328505 | 0.00715273 | 4.40E-06 | -0.0459787 | 0.0488621 | 0.346711 |
| 149 | alcohol intake  frequency | rs4739105 | C | T | 0.787783 | -0.019804 | 0.00374496 | 1.20E-07 | 0.0257942 | 0.0281095 | 0.35881 |
| 150 | alcohol intake  frequency | rs4742659 | G | A | 0.246749 | 0.0166303 | 0.00350794 | 2.10E-06 | 0.00912658 | 0.0261555 | 0.727138 |

| 151 | alcohol intake  frequency | rs4757370 | A | G | 0.263616 | 0.00536604 | 0.00343567 | 0.12 | 0.0104686 | 0.0244729 | 0.668824 |
| --- | --- | --- | --- | --- | --- | --- | --- | --- | --- | --- | --- |
| 152 | alcohol intake  frequency | rs4811031 | G | A | 0.348533 | 0.0146 | 0.00317873 | 4.40E-06 | 0.0547366 | 0.023324 | 0.0189361 |
| 153 | alcohol intake  frequency | rs4815366 | T | G | 0.639069 | -0.0159884 | 0.00315907 | 4.20E-07 | -0.0143018 | 0.0245498 | 0.560188 |
| 154 | alcohol intake  frequency | rs4865166 | T | C | 0.276936 | 0.017361 | 0.00338473 | 2.90E-07 | -0.0473867 | 0.0274447 | 0.0842345 |
| 155 | alcohol intake  frequency | rs489062 | A | G | 0.437454 | 0.0166498 | 0.00305291 | 4.90E-08 | 0.0245083 | 0.0227978 | 0.282362 |
| 156 | alcohol intake  frequency | rs4900965 | C | T | 0.306907 | 0.00216846 | 0.00331669 | 0.51 | 0.0354922 | 0.0236904 | 0.13409 |
| 157 | alcohol intake  frequency | rs4916723 | C | A | 0.420617 | 0.0239479 | 0.00309951 | 1.10E-14 | -0.00297528 | 0.0226358 | 0.895426 |
| 158 | alcohol intake  frequency | rs4968391 | T | G | 0.674892 | -0.0192695 | 0.0032265 | 2.30E-09 | -0.0183514 | 0.0231812 | 0.428563 |
| 159 | alcohol intake  frequency | rs4982052 | G | A | 0.34022 | -0.0162733 | 0.00320134 | 3.70E-07 | 0.0352896 | 0.0236763 | 0.136093 |
| 160 | alcohol intake  frequency | rs533143 | C | T | 0.288593 | -0.0180506 | 0.00338932 | 1.00E-07 | 0.00748736 | 0.0306351 | 0.806918 |
| 161 | alcohol intake  frequency | rs551998 | C | T | 0.366895 | -0.0156552 | 0.00314936 | 6.70E-07 | 0.00537583 | 0.02293 | 0.814639 |
| 162 | alcohol intake  frequency | rs56006101 | T | C | 0.019362 | -0.00750301 | 0.0113143 | 0.51 | -0.143078 | 0.071927 | 0.0466778 |
| 163 | alcohol intake  frequency | rs56149652 | G | A | 0.24756 | -0.0186206 | 0.00351342 | 1.20E-07 | -0.021902 | 0.0251478 | 0.383793 |
| 164 | alcohol intake  frequency | rs56194430 | T | C | 0.16931 | 0.0225403 | 0.00407148 | 3.10E-08 | -0.00878059 | 0.0343307 | 0.798133 |
| 165 | alcohol intake  frequency | rs56228311 | T | G | 0.342458 | -0.0151059 | 0.0031988 | 2.30E-06 | -0.0130673 | 0.0270064 | 0.628485 |
| 166 | alcohol intake  frequency | rs565078 | T | C | 0.485317 | 0.0143649 | 0.00303964 | 2.30E-06 | -0.0252861 | 0.0226985 | 0.265278 |
| 167 | alcohol intake  frequency | rs565522 | C | T | 0.429988 | 0.0141086 | 0.00306229 | 4.10E-06 | 0.0198018 | 0.0225791 | 0.380488 |
| 168 | alcohol intake  frequency | rs568030 | T | G | 0.741994 | -0.013889 | 0.00347731 | 6.50E-05 | -0.0416929 | 0.0230582 | 0.0705814 |
| 169 | alcohol intake  frequency | rs58905411 | A | G | 0.410052 | -0.0266343 | 0.00307848 | 5.10E-18 | 0.00849927 | 0.0227709 | 0.708962 |

| 170 | alcohol intake  frequency | rs59188188 | T | G | 0.290363 | 0.0170449 | 0.00334179 | 3.40E-07 | 0.0293951 | 0.024518 | 0.230559 |
| --- | --- | --- | --- | --- | --- | --- | --- | --- | --- | --- | --- |
| 171 | alcohol intake  frequency | rs6030200 | A | G | 0.31415 | -0.019529 | 0.00327075 | 2.40E-09 | -0.000330149 | 0.0229988 | 0.988547 |
| 172 | alcohol intake  frequency | rs6079439 | G | A | 0.527488 | -0.0151677 | 0.00303993 | 6.10E-07 | -0.00449776 | 0.0227286 | 0.843131 |
| 173 | alcohol intake  frequency | rs61825452 | T | A | 0.192752 | 0.0180098 | 0.00388612 | 3.60E-06 | -0.014201 | 0.0291549 | 0.626196 |
| 174 | alcohol intake  frequency | rs61873510 | T | G | 0.32785 | 0.0203737 | 0.00330311 | 6.90E-10 | 0.000447365 | 0.0248361 | 0.985629 |
| 175 | alcohol intake  frequency | rs62135521 | T | G | 0.048482 | 0.0344792 | 0.00703689 | 9.60E-07 | 0.0817264 | 0.054859 | 0.136289 |
| 176 | alcohol intake  frequency | rs62182135 | A | C | 0.330638 | -0.0150592 | 0.00321408 | 2.80E-06 | 0.01944 | 0.0247126 | 0.43149 |
| 177 | alcohol intake  frequency | rs62271373 | A | T | 0.060006 | 0.0334009 | 0.006494 | 2.70E-07 | 0.0683639 | 0.0555359 | 0.218328 |
| 178 | alcohol intake  frequency | rs62304163 | T | C | 0.042477 | 0.0383203 | 0.00756497 | 4.10E-07 | -0.121934 | 0.0617203 | 0.0482014 |
| 179 | alcohol intake  frequency | rs62305780 | G | C | 0.102253 | -0.0485216 | 0.00506585 | 9.90E-22 | -0.0163284 | 0.0327775 | 0.618371 |
| 180 | alcohol intake  frequency | rs62335889 | T | A | 0.267131 | -0.0085307 | 0.00344961 | 0.0129999 | 0.000446457 | 0.0251097 | 0.985814 |
| 181 | alcohol intake  frequency | rs62339673 | A | C | 0.626705 | 0.0182943 | 0.00315406 | 6.60E-09 | -0.00581749 | 0.0238199 | 0.807054 |
| 182 | alcohol intake  frequency | rs62439319 | T | C | 0.140966 | 0.0213766 | 0.0043477 | 8.80E-07 | 0.0116606 | 0.0338513 | 0.730496 |
| 183 | alcohol intake  frequency | rs62466318 | T | C | 0.202827 | -0.0254919 | 0.0037742 | 1.40E-11 | 0.0169523 | 0.0287966 | 0.556068 |
| 184 | alcohol intake  frequency | rs62573521 | T | C | 0.040759 | 0.0244337 | 0.00768485 | 0.0015 | 0.13346 | 0.095121 | 0.160599 |
| 185 | alcohol intake  frequency | rs627685 | C | T | 0.303538 | 0.0146274 | 0.00331772 | 1.00E-05 | 0.0268714 | 0.0257046 | 0.295842 |
| 186 | alcohol intake  frequency | rs6427160 | C | T | 0.421605 | 0.0143061 | 0.00306334 | 3.00E-06 | 0.00324104 | 0.0227547 | 0.886737 |
| 187 | alcohol intake  frequency | rs6442994 | T | C | 0.774721 | 0.0021084 | 0.00365463 | 0.56 | 0.0202617 | 0.0259564 | 0.435034 |
| 188 | alcohol intake  frequency | rs650558 | T | C | 0.247918 | 0.0207362 | 0.0035079 | 3.40E-09 | 0.0142324 | 0.0276215 | 0.606366 |

| 189 | alcohol intake  frequency | rs6531648 | C | T | 0.517258 | 0.0153231 | 0.00302708 | 4.10E-07 | -0.0329961 | 0.023154 | 0.154136 |
| --- | --- | --- | --- | --- | --- | --- | --- | --- | --- | --- | --- |
| 190 | alcohol intake  frequency | rs6534293 | G | A | 0.556934 | 0.0140304 | 0.00306044 | 4.60E-06 | -0.0116389 | 0.0227104 | 0.608307 |
| 191 | alcohol intake  frequency | rs6700839 | A | G | 0.567975 | 0.0147389 | 0.00306802 | 1.60E-06 | 0.0463758 | 0.0229 | 0.0428519 |
| 192 | alcohol intake  frequency | rs6727281 | T | C | 0.184023 | -0.024322 | 0.00391964 | 5.50E-10 | 0.0404027 | 0.0258477 | 0.118028 |
| 193 | alcohol intake  frequency | rs6744640 | G | A | 0.436123 | 0.015141 | 0.00305915 | 7.40E-07 | -0.0296169 | 0.0247124 | 0.230736 |
| 194 | alcohol intake  frequency | rs67822265 | T | C | 0.380951 | 0.0147902 | 0.00312904 | 2.30E-06 | -0.0592622 | 0.0237159 | 0.0124603 |
| 195 | alcohol intake  frequency | rs68024891 | C | T | 0.232972 | -0.0179697 | 0.00360018 | 6.00E-07 | 0.017053 | 0.0269845 | 0.527416 |
| 196 | alcohol intake  frequency | rs6810396 | G | C | 0.195181 | -0.0202611 | 0.00382656 | 1.20E-07 | -0.00378634 | 0.02503 | 0.879761 |
| 197 | alcohol intake  frequency | rs6819372 | G | A | 0.533037 | -0.0163435 | 0.00303392 | 7.20E-08 | -0.0495593 | 0.0227944 | 0.0296907 |
| 198 | alcohol intake  frequency | rs6908328 | A | C | 0.51424 | -0.00593795 | 0.00302821 | 0.05 | 0.0118016 | 0.0236151 | 0.617253 |
| 199 | alcohol intake  frequency | rs693458 | C | A | 0.265691 | 0.018623 | 0.00342728 | 5.50E-08 | 0.0381868 | 0.0293511 | 0.193247 |
| 200 | alcohol intake  frequency | rs6978944 | G | A | 0.203026 | 0.00569388 | 0.00377244 | 0.13 | 0.0239569 | 0.0263869 | 0.363928 |
| 201 | alcohol intake  frequency | rs6992604 | C | T | 0.290386 | 0.0165551 | 0.00335128 | 7.80E-07 | -0.00357999 | 0.0244465 | 0.883572 |
| 202 | alcohol intake  frequency | rs6993770 | T | A | 0.287365 | -0.0165469 | 0.00334122 | 7.30E-07 | 0.0255879 | 0.0272525 | 0.347772 |
| 203 | alcohol intake  frequency | rs7029768 | T | G | 0.128925 | -0.0233267 | 0.00452161 | 2.50E-07 | 0.0159432 | 0.0349405 | 0.648176 |
| 204 | alcohol intake  frequency | rs7046881 | G | T | 0.423226 | -0.014238 | 0.00307135 | 3.60E-06 | 0.00255594 | 0.025573 | 0.920387 |
| 205 | alcohol intake  frequency | rs71651683 | T | C | 0.0142 | -0.0704589 | 0.0127906 | 3.60E-08 | -0.252724 | 0.135061 | 0.0613197 |
| 206 | alcohol intake  frequency | rs72641050 | C | A | 0.247115 | 0.0170168 | 0.00352184 | 1.40E-06 | -0.00714887 | 0.0240957 | 0.766706 |
| 207 | alcohol intake  frequency | rs72642323 | G | T | 0.008282 | 0.0885015 | 0.016743 | 1.30E-07 | 0.15774 | 0.26794 | 0.556054 |

| 208 | alcohol intake  frequency | rs7277942 | T | C | 0.17297 | -0.00536302 | 0.00401259 | 0.18 | -0.0245781 | 0.0303094 | 0.417419 |
| --- | --- | --- | --- | --- | --- | --- | --- | --- | --- | --- | --- |
| 209 | alcohol intake  frequency | rs72787062 | A | G | 0.162767 | -0.0281947 | 0.00410324 | 6.40E-12 | -0.0258147 | 0.0319101 | 0.418527 |
| 210 | alcohol intake  frequency | rs728538 | G | T | 0.168868 | 0.0228752 | 0.00406259 | 1.80E-08 | 0.0395208 | 0.028947 | 0.172165 |
| 211 | alcohol intake  frequency | rs7302200 | A | G | 0.339998 | -0.0184222 | 0.00319841 | 8.40E-09 | -0.018898 | 0.0247451 | 0.445044 |
| 212 | alcohol intake  frequency | rs73050128 | A | C | 0.164488 | -0.0260048 | 0.00409088 | 2.10E-10 | 0.0230786 | 0.026384 | 0.381727 |
| 213 | alcohol intake  frequency | rs7330939 | T | C | 0.720352 | -0.0213301 | 0.0034046 | 3.70E-10 | 0.0176287 | 0.0239395 | 0.461497 |
| 214 | alcohol intake  frequency | rs73543290 | A | G | 0.044089 | -0.0356162 | 0.00744089 | 1.70E-06 | -0.102248 | 0.0754584 | 0.175411 |
| 215 | alcohol intake  frequency | rs7357754 | G | A | 0.500148 | 0.0149913 | 0.00303636 | 7.90E-07 | 0.013318 | 0.0226272 | 0.55614 |
| 216 | alcohol intake  frequency | rs7431028 | G | T | 0.526692 | 0.00995347 | 0.00304643 | 0.00109999 | -0.0112367 | 0.0227373 | 0.621166 |
| 217 | alcohol intake  frequency | rs7433378 | T | C | 0.231818 | -0.0115286 | 0.003656 | 0.0016 | -0.00413364 | 0.0270996 | 0.878765 |
| 218 | alcohol intake  frequency | rs74424378 | G | T | 0.23761 | 0.0176039 | 0.00356049 | 7.60E-07 | 0.00700843 | 0.0256422 | 0.78461 |
| 219 | alcohol intake  frequency | rs74439139 | G | T | 0.091282 | 0.0260691 | 0.00540153 | 1.40E-06 | -0.052246 | 0.0510335 | 0.30595 |
| 220 | alcohol intake  frequency | rs74567946 | G | T | 0.028721 | -0.012396 | 0.00916224 | 0.18 | -0.0807796 | 0.0713049 | 0.257266 |
| 221 | alcohol intake  frequency | rs7460106 | C | T | 0.23562 | -0.0217112 | 0.00361927 | 2.00E-09 | 0.0240849 | 0.0258825 | 0.352087 |
| 222 | alcohol intake  frequency | rs74679146 | C | T | 0.074515 | -0.0320735 | 0.00575757 | 2.50E-08 | -0.0222233 | 0.043923 | 0.612884 |
| 223 | alcohol intake  frequency | rs74808805 | T | G | 0.043603 | -0.0350455 | 0.0075006 | 3.00E-06 | -0.0870283 | 0.070264 | 0.215497 |
| 224 | alcohol intake  frequency | rs7514579 | C | A | 0.232457 | 0.0196672 | 0.00359788 | 4.60E-08 | -0.0516689 | 0.0277706 | 0.0628058 |
| 225 | alcohol intake  frequency | rs7546040 | G | C | 0.731004 | -0.0146415 | 0.00341036 | 1.80E-05 | 0.041383 | 0.0259256 | 0.110439 |
| 226 | alcohol intake  frequency | rs7587791 | C | A | 0.51317 | -0.015383 | 0.00302725 | 3.70E-07 | -0.0148308 | 0.0227979 | 0.515347 |

| 227 | alcohol intake  frequency | rs76082653 | T | C | 0.054327 | 0.0464269 | 0.00668673 | 3.80E-12 | 0.0764467 | 0.0645011 | 0.235938 |
| --- | --- | --- | --- | --- | --- | --- | --- | --- | --- | --- | --- |
| 228 | alcohol intake  frequency | rs7610856 | A | C | 0.429053 | -0.023864 | 0.00307022 | 7.70E-15 | 0.000137035 | 0.0232062 | 0.995288 |
| 229 | alcohol intake  frequency | rs7616201 | G | A | 0.348507 | -0.0146143 | 0.00318942 | 4.60E-06 | 0.0116921 | 0.0237306 | 0.622224 |
| 230 | alcohol intake  frequency | rs767870 | A | G | 0.849798 | -0.0196656 | 0.00423795 | 3.50E-06 | -0.026274 | 0.0340551 | 0.440401 |
| 231 | alcohol intake  frequency | rs77169096 | A | T | 0.011361 | 0.0322807 | 0.014686 | 0.0280001 | 0.0295034 | 0.0790844 | 0.709102 |
| 232 | alcohol intake  frequency | rs77371894 | G | C | 0.090781 | 0.0254952 | 0.00528899 | 1.40E-06 | 0.0269152 | 0.0528736 | 0.610718 |
| 233 | alcohol intake  frequency | rs780569 | A | T | 0.70882 | 0.0198033 | 0.00336454 | 4.00E-09 | -0.0358402 | 0.0267686 | 0.180607 |
| 234 | alcohol intake  frequency | rs7973103 | T | C | 0.193762 | -0.0202521 | 0.00383338 | 1.30E-07 | -0.0318631 | 0.0379111 | 0.400646 |
| 235 | alcohol intake  frequency | rs7977455 | G | C | 0.762009 | 0.0174495 | 0.00356102 | 9.60E-07 | -0.0280175 | 0.0258675 | 0.278757 |
| 236 | alcohol intake  frequency | rs79942197 | G | C | 0.064046 | -0.0301693 | 0.00621192 | 1.20E-06 | 0.0226945 | 0.064315 | 0.724189 |
| 237 | alcohol intake  frequency | rs80101850 | C | T | 0.038332 | 0.0398214 | 0.00796143 | 5.70E-07 | -0.0977829 | 0.0553632 | 0.0773606 |
| 238 | alcohol intake  frequency | rs8015726 | C | T | 0.362509 | -0.0157641 | 0.00316883 | 6.50E-07 | -0.00121843 | 0.0254575 | 0.961827 |
| 239 | alcohol intake  frequency | rs80214940 | G | A | 0.17751 | -0.0184159 | 0.00398593 | 3.80E-06 | 0.0242296 | 0.0297997 | 0.416172 |
| 240 | alcohol intake  frequency | rs80255756 | A | G | 0.063277 | 0.0316484 | 0.00623365 | 3.80E-07 | 0.0637283 | 0.0853467 | 0.455245 |
| 241 | alcohol intake  frequency | rs80292319 | C | T | 0.057704 | -0.0393728 | 0.00649583 | 1.40E-09 | -0.00267925 | 0.0413441 | 0.94833 |
| 242 | alcohol intake  frequency | rs8030809 | A | G | 0.553608 | 0.0164409 | 0.00306751 | 8.30E-08 | 0.0451347 | 0.0229857 | 0.049577 |
| 243 | alcohol intake  frequency | rs803223 | A | G | 0.74506 | -0.0172308 | 0.00347132 | 6.90E-07 | -0.0148328 | 0.0228983 | 0.517134 |
| 244 | alcohol intake  frequency | rs8043563 | C | G | 0.737192 | 0.0233654 | 0.00347125 | 1.70E-11 | 0.0167797 | 0.0254415 | 0.509549 |
| 245 | alcohol intake  frequency | rs8050545 | G | A | 0.112293 | 0.0238016 | 0.00479516 | 6.90E-07 | 0.0241717 | 0.0281981 | 0.39133 |

| 246 | alcohol intake  frequency | rs8083110 | T | C | 0.418707 | -0.0168099 | 0.00307792 | 4.70E-08 | 0.0324019 | 0.0226579 | 0.152703 |
| --- | --- | --- | --- | --- | --- | --- | --- | --- | --- | --- | --- |
| 247 | alcohol intake  frequency | rs838145 | A | G | 0.542982 | 0.0219548 | 0.00305549 | 6.70E-13 | -0.00428127 | 0.0237587 | 0.856997 |
| 248 | alcohol intake  frequency | rs874296 | C | T | 0.386036 | 0.0159123 | 0.00312504 | 3.50E-07 | 0.00297073 | 0.0230431 | 0.897421 |
| 249 | alcohol intake  frequency | rs898751 | T | C | 0.492948 | 0.00806466 | 0.00302968 | 0.00779992 | 0.0109225 | 0.022663 | 0.62984 |
| 250 | alcohol intake  frequency | rs900802 | T | C | 0.694659 | -0.0169459 | 0.00330306 | 2.90E-07 | -0.0341132 | 0.0251307 | 0.174645 |
| 251 | alcohol intake  frequency | rs906580 | T | A | 0.683388 | -0.0110784 | 0.00327268 | 0.000710003 | 0.0114961 | 0.0238715 | 0.630104 |
| 252 | alcohol intake  frequency | rs911475 | T | C | 0.166508 | 0.019878 | 0.0041038 | 1.30E-06 | -0.0148029 | 0.0363634 | 0.683946 |
| 253 | alcohol intake  frequency | rs927769 | A | C | 0.627148 | -0.00342248 | 0.00313394 | 0.27 | -0.00310665 | 0.0230752 | 0.892903 |
| 254 | alcohol intake  frequency | rs9349379 | G | A | 0.405493 | -0.0193455 | 0.00308215 | 3.50E-10 | -0.0176599 | 0.0226143 | 0.434852 |
| 255 | alcohol intake  frequency | rs9350781 | T | A | 0.351505 | 0.0150037 | 0.00318403 | 2.50E-06 | 0.0221849 | 0.023922 | 0.353726 |
| 256 | alcohol intake  frequency | rs9372625 | A | G | 0.381706 | -0.0255579 | 0.00312467 | 2.90E-16 | -0.0218925 | 0.0242832 | 0.367297 |
| 257 | alcohol intake  frequency | rs9403297 | A | G | 0.372967 | 0.0188234 | 0.00313037 | 1.80E-09 | 0.0232451 | 0.0246367 | 0.345419 |
| 258 | alcohol intake  frequency | rs9537938 | A | G | 0.682279 | -0.0171193 | 0.00326026 | 1.50E-07 | 0.000802024 | 0.0240428 | 0.973389 |
| 259 | alcohol intake  frequency | rs956752 | G | A | 0.100624 | 0.0255759 | 0.00502689 | 3.60E-07 | 0.00621876 | 0.0412838 | 0.880264 |
| 260 | alcohol intake  frequency | rs9611953 | G | A | 0.491257 | 0.0158205 | 0.00303095 | 1.80E-07 | -0.0242536 | 0.0233914 | 0.299801 |
| 261 | alcohol intake  frequency | rs9648478 | A | G | 0.510245 | 0.0168603 | 0.00302891 | 2.60E-08 | 0.0137287 | 0.0225884 | 0.543337 |
| 262 | alcohol intake  frequency | rs9690021 | T | G | 0.491326 | 0.0150599 | 0.00302732 | 6.50E-07 | 0.0325362 | 0.0226643 | 0.151125 |
| 263 | alcohol intake  frequency | rs9728289 | G | A | 0.410815 | 0.000211766 | 0.00307654 | 0.95 | 0.00906541 | 0.0228161 | 0.691129 |
| 264 | alcohol intake  frequency | rs9899357 | C | T | 0.381903 | 0.00819105 | 0.00313084 | 0.0089 | 0.0529102 | 0.0237016 | 0.0255918 |

| 265 | alcohol intake  frequency | rs9902512 | G | C | 0.356406 | -0.0144847 | 0.00316271 | 4.70E-06 | -0.014281 | 0.0241189 | 0.553778 |
| --- | --- | --- | --- | --- | --- | --- | --- | --- | --- | --- | --- |
| 266 | alcohol intake  frequency | rs9906502 | A | G | 0.176998 | 0.0237883 | 0.00396178 | 1.90E-09 | 0.0209678 | 0.028654 | 0.464314 |

Table S6 Instrumental variables used in multivariable MR analysis of red wine to ICH.

|  | **Exposure** | **SNP** | **Effect**  **Allele** | **Other**  **Allele** | **EAF** | **beta.red_wine** | **se.red_wine** | **pval.red_wine** | **beta.ICH** | **se.ICH** | **pval.ICH** |
| --- | --- | --- | --- | --- | --- | --- | --- | --- | --- | --- | --- |
| 1 | average weekly  red wine intake | rs1004787 | A | G | 0.534112 | 0.00435672 | 0.00207619 | 0.0359998 | 0.0336329 | 0.0233432 | 0.14964 |
| 2 | average weekly  red wine intake | rs1006231 | T | C | 0.83677 | -2.18E-05 | 0.00280027 | 0.99 | 0.00619598 | 0.0340613 | 0.855656 |
| 3 | average weekly  red wine intake | rs10188314 | T | C | 0.472849 | 0.00330922 | 0.00206911 | 0.11 | -0.0283108 | 0.0226898 | 0.212129 |
| 4 | average weekly  red wine intake | rs10210652 | A | G | 0.350016 | -0.010268 | 0.00216574 | 2.10E-06 | 0.0658306 | 0.0233259 | 0.00476947 |
| 5 | average weekly  red wine intake | rs1022666 | T | G | 0.864186 | 0.00627806 | 0.00301518 | 0.0369999 | -0.00729371 | 0.0384716 | 0.849633 |
| 6 | average weekly  red wine intake | rs10262713 | C | G | 0.114538 | 0.00233066 | 0.00323622 | 0.47 | 0.00696302 | 0.0304942 | 0.819383 |
| 7 | average weekly  red wine intake | rs10280836 | G | T | 0.771368 | -0.0123121 | 0.00245691 | 5.40E-07 | 0.0280258 | 0.0282984 | 0.321993 |
| 8 | average weekly  red wine intake | rs10777337 | G | A | 0.657247 | -0.00433353 | 0.00217835 | 0.0470002 | -0.0026185 | 0.0250958 | 0.9169 |
| 9 | average weekly  red wine intake | rs10792669 | G | A | 0.503663 | -0.00374151 | 0.00207164 | 0.0710003 | -0.0268228 | 0.0226428 | 0.236173 |
| 10 | average weekly  red wine intake | rs10822129 | T | C | 0.406521 | 0.01344 | 0.0021284 | 2.70E-10 | -0.0459135 | 0.023004 | 0.0459462 |
| 11 | average weekly  red wine intake | rs1083347 | A | T | 0.20246 | -0.000168441 | 0.00258669 | 0.95 | 0.0164541 | 0.0269102 | 0.540905 |
| 12 | average weekly  red wine intake | rs10835498 | A | G | 0.429806 | -0.000297624 | 0.00208654 | 0.89 | 0.028455 | 0.0226465 | 0.20894 |
| 13 | average weekly  red wine intake | rs10847019 | T | G | 0.018763 | -0.00832999 | 0.00759179 | 0.27 | 0.00891364 | 0.0724126 | 0.902032 |
| 14 | average weekly  red wine intake | rs10860862 | T | G | 0.181686 | 0.00495964 | 0.00268418 | 0.0649995 | -0.045759 | 0.026806 | 0.0878132 |
| 15 | average weekly  red wine intake | rs10865093 | C | T | 0.447573 | -0.00959194 | 0.00207867 | 3.90E-06 | 0.00579356 | 0.0227902 | 0.799331 |
| 16 | average weekly  red wine intake | rs10873070 | G | A | 0.624045 | -0.00999457 | 0.00215359 | 3.50E-06 | 0.013115 | 0.0242925 | 0.589282 |
| 17 | average weekly  red wine intake | rs10893956 | A | T | 0.177989 | 0.00327197 | 0.00269674 | 0.23 | 0.00734126 | 0.0269805 | 0.785548 |

| 18 | average weekly  red wine intake | rs10925183 | A | G | 0.607433 | -0.011922 | 0.00211168 | 1.60E-08 | 0.0239068 | 0.0233742 | 0.306408 |
| --- | --- | --- | --- | --- | --- | --- | --- | --- | --- | --- | --- |
| 19 | average weekly  red wine intake | rs11024405 | G | A | 0.185291 | -0.0137073 | 0.00266707 | 2.80E-07 | 0.0346548 | 0.0269934 | 0.199204 |
| 20 | average weekly  red wine intake | rs11039429 | T | C | 0.456485 | 0.00505115 | 0.00206924 | 0.015 | 0.00394853 | 0.0234399 | 0.866227 |
| 21 | average weekly  red wine intake | rs11109118 | C | A | 0.152811 | -0.00555027 | 0.00287696 | 0.0539995 | 0.0162593 | 0.0288195 | 0.572634 |
| 22 | average weekly  red wine intake | rs11114498 | T | C | 0.673867 | -0.0102206 | 0.00220386 | 3.50E-06 | 0.00662552 | 0.0250259 | 0.791205 |
| 23 | average weekly  red wine intake | rs11176488 | C | T | 0.321129 | 6.16E-05 | 0.00221603 | 0.98 | -0.0267466 | 0.0233211 | 0.251429 |
| 24 | average weekly  red wine intake | rs11221890 | G | A | 0.148265 | 0.0158665 | 0.00291925 | 5.50E-08 | 0.0190092 | 0.0328449 | 0.562753 |
| 25 | average weekly  red wine intake | rs11223617 | A | G | 0.20459 | -0.00743584 | 0.00256382 | 0.00369999 | 0.0462812 | 0.0248575 | 0.0626224 |
| 26 | average weekly  red wine intake | rs113893318 | T | C | 0.040912 | 0.0106655 | 0.00522973 | 0.0409996 | 0.00972762 | 0.0534033 | 0.855462 |
| 27 | average weekly  red wine intake | rs114313565 | A | G | 0.027269 | 0.0320306 | 0.00653599 | 9.60E-07 | -0.106774 | 0.107851 | 0.32217 |
| 28 | average weekly  red wine intake | rs114795493 | A | G | 0.016059 | 0.0393968 | 0.00819179 | 1.50E-06 | -0.0203441 | 0.141144 | 0.885392 |
| 29 | average weekly  red wine intake | rs11646721 | T | C | 0.074717 | -0.0134904 | 0.00395317 | 0.00064 | 0.0559423 | 0.0521918 | 0.283783 |
| 30 | average weekly  red wine intake | rs11662234 | G | T | 0.524066 | -0.00945495 | 0.00206748 | 4.80E-06 | 0.0101229 | 0.022876 | 0.658118 |
| 31 | average weekly  red wine intake | rs11700855 | G | A | 0.094601 | 0.00547217 | 0.00354584 | 0.12 | -0.0557617 | 0.0348577 | 0.109666 |
| 32 | average weekly  red wine intake | rs11705856 | A | G | 0.642632 | 0.01074 | 0.00215133 | 6.00E-07 | 0.0375897 | 0.0231147 | 0.103901 |
| 33 | average weekly  red wine intake | rs11715683 | A | T | 0.344906 | 0.013978 | 0.00217352 | 1.30E-10 | -0.0224253 | 0.023785 | 0.345764 |
| 34 | average weekly  red wine intake | rs117317480 | A | G | 0.052854 | -0.000959466 | 0.00476569 | 0.84 | -0.0855837 | 0.0601672 | 0.154901 |
| 35 | average weekly  red wine intake | rs117386364 | A | G | 0.045884 | 0.00725636 | 0.00512236 | 0.16 | 0.0985777 | 0.0519023 | 0.0575254 |
| 36 | average weekly  red wine intake | rs11750012 | G | A | 0.860815 | -0.000201605 | 0.00297487 | 0.95 | -0.0248223 | 0.0347106 | 0.474535 |

| 37 | average weekly  red wine intake | rs11750777 | A | G | 0.210766 | 0.00220553 | 0.00253161 | 0.38 | -0.0128457 | 0.031552 | 0.683913 |
| --- | --- | --- | --- | --- | --- | --- | --- | --- | --- | --- | --- |
| 38 | average weekly  red wine intake | rs11787216 | T | C | 0.36809 | -0.0101948 | 0.00218122 | 3.00E-06 | 0.00411378 | 0.0235864 | 0.861541 |
| 39 | average weekly  red wine intake | rs118185251 | C | T | 0.018395 | -0.00245319 | 0.00798241 | 0.760001 | 0.0969463 | 0.214315 | 0.651014 |
| 40 | average weekly  red wine intake | rs11877758 | G | T | 0.310973 | -0.0107631 | 0.00223808 | 1.50E-06 | -0.0391458 | 0.0259396 | 0.13127 |
| 41 | average weekly  red wine intake | rs11940694 | G | A | 0.60862 | 0.00643083 | 0.00212684 | 0.0025 | -0.0106154 | 0.0236487 | 0.653519 |
| 42 | average weekly  red wine intake | rs12030413 | C | G | 0.178642 | 0.012622 | 0.00272179 | 3.50E-06 | 0.0568491 | 0.0307562 | 0.0645476 |
| 43 | average weekly  red wine intake | rs12139282 | A | G | 0.08488 | -0.000116603 | 0.00378882 | 0.98 | -0.0154752 | 0.0413037 | 0.707907 |
| 44 | average weekly  red wine intake | rs12203592 | T | C | 0.219379 | -0.00651443 | 0.00245508 | 0.008 | 0.043368 | 0.067592 | 0.521124 |
| 45 | average weekly  red wine intake | rs12240850 | A | G | 0.036039 | -0.00954301 | 0.00560252 | 0.089 | 0.115094 | 0.0639983 | 0.0721157 |
| 46 | average weekly  red wine intake | rs1228589 | A | G | 0.244782 | -0.00564421 | 0.00240832 | 0.0189998 | -0.00407501 | 0.0261873 | 0.87634 |
| 47 | average weekly  red wine intake | rs1229984 | C | T | 0.976512 | 0.0609318 | 0.00673472 | 1.50E-19 | -0.279883 | 0.162525 | 0.0850531 |
| 48 | average weekly  red wine intake | rs12436192 | A | G | 0.17475 | -0.00472819 | 0.00271394 | 0.0810009 | 0.0151422 | 0.0307856 | 0.622818 |
| 49 | average weekly  red wine intake | rs12467407 | T | C | 0.468308 | -0.00206101 | 0.0020743 | 0.32 | 0.0264565 | 0.0227741 | 0.245362 |
| 50 | average weekly  red wine intake | rs12598321 | T | C | 0.558979 | -0.00577303 | 0.00207879 | 0.00549997 | 0.0148834 | 0.0231011 | 0.519399 |
| 51 | average weekly  red wine intake | rs12692596 | T | C | 0.370567 | -0.0116782 | 0.0021345 | 4.50E-08 | 0.00561581 | 0.0238362 | 0.813743 |
| 52 | average weekly  red wine intake | rs12740811 | G | A | 0.093779 | -0.0105622 | 0.00353683 | 0.00280001 | 0.0963496 | 0.0411282 | 0.0191465 |
| 53 | average weekly  red wine intake | rs12756299 | C | T | 0.490398 | 0.00231837 | 0.00206499 | 0.26 | 0.000615922 | 0.0226334 | 0.97829 |
| 54 | average weekly  red wine intake | rs12784183 | T | G | 0.141633 | 0.00034065 | 0.00297444 | 0.91 | -0.00121816 | 0.0350172 | 0.972249 |
| 55 | average weekly  red wine intake | rs12814101 | G | C | 0.585403 | 0.0015212 | 0.00209616 | 0.47 | 0.0070312 | 0.022956 | 0.759383 |

| 56 | average weekly  red wine intake | rs13024996 | A | C | 0.368265 | -0.00151405 | 0.00213601 | 0.48 | -0.00818713 | 0.0241246 | 0.734333 |
| --- | --- | --- | --- | --- | --- | --- | --- | --- | --- | --- | --- |
| 57 | average weekly  red wine intake | rs13034936 | C | T | 0.111522 | -0.0172726 | 0.00327463 | 1.30E-07 | -0.0451505 | 0.0560858 | 0.420804 |
| 58 | average weekly  red wine intake | rs13102973 | C | T | 0.621381 | 0.00168558 | 0.00212837 | 0.43 | 0.0482853 | 0.0227723 | 0.0339766 |
| 59 | average weekly  red wine intake | rs13120366 | C | T | 0.504645 | -0.00491764 | 0.00205945 | 0.017 | 0.00916856 | 0.0225904 | 0.684845 |
| 60 | average weekly  red wine intake | rs13135092 | G | A | 0.082455 | -0.014127 | 0.0037689 | 0.000179999 | -0.0946123 | 0.0868482 | 0.275978 |
| 61 | average weekly  red wine intake | rs13153216 | G | A | 0.025258 | 0.00144916 | 0.00656128 | 0.83 | -0.0782473 | 0.0690689 | 0.257262 |
| 62 | average weekly  red wine intake | rs13178443 | T | C | 0.278441 | 0.00418309 | 0.0023043 | 0.0690001 | 0.0561715 | 0.0245841 | 0.0223203 |
| 63 | average weekly  red wine intake | rs13248791 | C | T | 0.444037 | 0.00100708 | 0.0021136 | 0.630001 | -0.00266355 | 0.0228002 | 0.907002 |
| 64 | average weekly  red wine intake | rs13378810 | G | C | 0.12002 | 0.00235445 | 0.00318314 | 0.46 | 0.0121715 | 0.0379795 | 0.748609 |
| 65 | average weekly  red wine intake | rs13390019 | C | T | 0.13228 | -0.0129871 | 0.0030777 | 2.40E-05 | 0.0270619 | 0.0469905 | 0.564682 |
| 66 | average weekly  red wine intake | rs13413257 | T | C | 0.012329 | -0.038984 | 0.00938755 | 3.30E-05 | -0.0132284 | 0.084261 | 0.87525 |
| 67 | average weekly  red wine intake | rs1377491 | T | A | 0.794814 | 0.00573707 | 0.00255269 | 0.025 | -0.000607758 | 0.0305398 | 0.984123 |
| 68 | average weekly  red wine intake | rs1421085 | C | T | 0.401598 | 0.01372 | 0.00210549 | 7.20E-11 | 0.00688791 | 0.022883 | 0.76341 |
| 69 | average weekly  red wine intake | rs1446577 | G | C | 0.256445 | 0.0151328 | 0.00236106 | 1.50E-10 | -0.0246988 | 0.024681 | 0.316962 |
| 70 | average weekly  red wine intake | rs1453027 | G | T | 0.538521 | -0.000109902 | 0.0020735 | 0.96 | -0.0498967 | 0.0227254 | 0.0281177 |
| 71 | average weekly  red wine intake | rs145898511 | G | T | 0.025231 | -0.0110268 | 0.00665941 | 0.0980009 | -0.0104895 | 0.0672007 | 0.87596 |
| 72 | average weekly  red wine intake | rs146821330 | C | T | 0.014265 | -0.0413513 | 0.00873602 | 2.20E-06 | -0.0109905 | 0.148946 | 0.941179 |
| 73 | average weekly  red wine intake | rs1468967 | A | G | 0.662984 | 0.00010023 | 0.00218842 | 0.96 | -0.0249441 | 0.0230291 | 0.278739 |
| 74 | average weekly  red wine intake | rs1488389 | G | C | 0.881732 | 0.00598023 | 0.00330427 | 0.0700003 | -0.026962 | 0.0389952 | 0.489301 |

| 75 | average weekly  red wine intake | rs1490492 | T | C | 0.662366 | -0.00343839 | 0.00218713 | 0.12 | 0.022619 | 0.0252647 | 0.370636 |
| --- | --- | --- | --- | --- | --- | --- | --- | --- | --- | --- | --- |
| 76 | average weekly  red wine intake | rs1515108 | T | C | 0.635242 | -0.00446884 | 0.00214182 | 0.0369999 | 0.00709603 | 0.0232465 | 0.760174 |
| 77 | average weekly  red wine intake | rs1515590 | T | C | 0.381406 | -0.00567281 | 0.00212492 | 0.00759994 | -0.0188095 | 0.0227675 | 0.408715 |
| 78 | average weekly  red wine intake | rs1558426 | C | T | 0.417292 | 0.00034943 | 0.00209667 | 0.87 | 0.0172712 | 0.0236964 | 0.466092 |
| 79 | average weekly  red wine intake | rs1569777 | T | C | 0.103684 | 0.00730154 | 0.00338929 | 0.0309999 | -0.0443269 | 0.0307774 | 0.149799 |
| 80 | average weekly  red wine intake | rs164328 | T | G | 0.912282 | 0.0184492 | 0.00367585 | 5.20E-07 | -0.00305474 | 0.049223 | 0.950516 |
| 81 | average weekly  red wine intake | rs1666658 | C | T | 0.390576 | -0.00484986 | 0.00211318 | 0.0219999 | -0.0240455 | 0.0232223 | 0.30046 |
| 82 | average weekly  red wine intake | rs16989140 | T | C | 0.088544 | -0.0190561 | 0.00364992 | 1.80E-07 | 0.0523782 | 0.0391935 | 0.181419 |
| 83 | average weekly  red wine intake | rs17127898 | A | T | 0.086971 | -0.00629368 | 0.003663 | 0.0860003 | 0.0814264 | 0.036957 | 0.0275753 |
| 84 | average weekly  red wine intake | rs17185470 | C | G | 0.167013 | 0.00357315 | 0.00278975 | 0.2 | 0.0480938 | 0.0313182 | 0.124624 |
| 85 | average weekly  red wine intake | rs1762777 | A | G | 0.757232 | 0.0064372 | 0.00240346 | 0.00739997 | -0.00587012 | 0.0297907 | 0.843792 |
| 86 | average weekly  red wine intake | rs17648701 | C | T | 0.178136 | 0.0103212 | 0.00271612 | 0.000140001 | -0.0263218 | 0.0285038 | 0.355774 |
| 87 | average weekly  red wine intake | rs17662759 | C | T | 0.088553 | -0.0093034 | 0.00373022 | 0.0129999 | 0.0235139 | 0.0366156 | 0.520754 |
| 88 | average weekly  red wine intake | rs17690703 | T | C | 0.261462 | -0.00752543 | 0.00233935 | 0.00129999 | 0.0224609 | 0.0350704 | 0.521879 |
| 89 | average weekly  red wine intake | rs185799410 | T | G | 0.025497 | -0.0228637 | 0.00664612 | 0.000580003 | -0.218351 | 0.0815943 | 0.00744955 |
| 90 | average weekly  red wine intake | rs186347 | T | G | 0.461085 | -0.00207518 | 0.00207782 | 0.32 | 0.001509 | 0.0227736 | 0.94717 |
| 91 | average weekly  red wine intake | rs1916414 | G | A | 0.535336 | 0.0100546 | 0.00207381 | 1.20E-06 | -0.0133746 | 0.0225526 | 0.553153 |
| 92 | average weekly  red wine intake | rs1937522 | G | A | 0.526406 | -0.00364737 | 0.00206392 | 0.0769999 | -0.0288024 | 0.022633 | 0.203166 |
| 93 | average weekly  red wine intake | rs1966836 | G | A | 0.710792 | 0.0123253 | 0.00227216 | 5.80E-08 | 0.0281182 | 0.024707 | 0.255093 |

| 94 | average weekly  red wine intake | rs1984584 | A | G | 0.436391 | 0.00500337 | 0.00207989 | 0.016 | 0.0171506 | 0.0226298 | 0.448524 |
| --- | --- | --- | --- | --- | --- | --- | --- | --- | --- | --- | --- |
| 95 | average weekly  red wine intake | rs2007761 | A | G | 0.270115 | -0.000711281 | 0.00235228 | 0.760001 | -0.00444972 | 0.0274718 | 0.871326 |
| 96 | average weekly  red wine intake | rs2009367 | G | T | 0.302149 | -0.00462449 | 0.00225298 | 0.04 | -0.0173323 | 0.0237619 | 0.465747 |
| 97 | average weekly  red wine intake | rs2055581 | A | G | 0.297836 | 6.85E-05 | 0.00227332 | 0.98 | 0.0178656 | 0.0278748 | 0.521573 |
| 98 | average weekly  red wine intake | rs2117137 | G | A | 0.405583 | 0.00983136 | 0.0020973 | 2.80E-06 | 0.0153229 | 0.0226933 | 0.499536 |
| 99 | average weekly  red wine intake | rs2160935 | T | C | 0.606114 | 0.00696552 | 0.00210679 | 0.000949992 | -0.0366499 | 0.0236739 | 0.121594 |
| 100 | average weekly  red wine intake | rs2224873 | A | T | 0.221582 | -0.00844898 | 0.0024887 | 0.000690001 | -0.0106269 | 0.0302063 | 0.72498 |
| 101 | average weekly  red wine intake | rs2238660 | G | A | 0.139357 | -0.00718164 | 0.00299732 | 0.017 | -0.0649638 | 0.0427603 | 0.128698 |
| 102 | average weekly  red wine intake | rs2291007 | C | T | 0.570657 | 0.0098601 | 0.00208449 | 2.20E-06 | -0.00416591 | 0.0236268 | 0.860042 |
| 103 | average weekly  red wine intake | rs2411453 | G | T | 0.599817 | 0.00958925 | 0.00210875 | 5.40E-06 | -0.0235346 | 0.0227039 | 0.299927 |
| 104 | average weekly  red wine intake | rs2511225 | C | A | 0.353774 | -0.0116801 | 0.00215811 | 6.20E-08 | 0.00957901 | 0.025447 | 0.706597 |
| 105 | average weekly  red wine intake | rs2535911 | T | C | 0.356734 | 0.00331062 | 0.00215456 | 0.12 | -0.00705287 | 0.0228122 | 0.757192 |
| 106 | average weekly  red wine intake | rs2586462 | G | A | 0.850143 | 0.00040839 | 0.00289338 | 0.89 | 0.00789003 | 0.0335379 | 0.814009 |
| 107 | average weekly  red wine intake | rs261046 | A | G | 0.245007 | 0.0111114 | 0.00241422 | 4.20E-06 | 0.0384532 | 0.0244477 | 0.115748 |
| 108 | average weekly  red wine intake | rs2622167 | A | G | 0.430093 | -0.00371515 | 0.00208776 | 0.0749998 | -0.0456116 | 0.0241745 | 0.059193 |
| 109 | average weekly  red wine intake | rs262240 | T | C | 0.469937 | 0.0053726 | 0.00206642 | 0.00929994 | -0.00954806 | 0.0232593 | 0.681436 |
| 110 | average weekly  red wine intake | rs2717063 | A | C | 0.587147 | 0.000180595 | 0.00210233 | 0.93 | 0.0391215 | 0.0231351 | 0.0908385 |
| 111 | average weekly  red wine intake | rs2789517 | A | G | 0.868394 | 0.0146578 | 0.00307273 | 1.80E-06 | 0.100126 | 0.0505432 | 0.0475905 |
| 112 | average weekly  red wine intake | rs28412070 | T | C | 0.350162 | 0.00282312 | 0.00217086 | 0.19 | -0.0060026 | 0.0255791 | 0.814466 |

| 113 | average weekly  red wine intake | rs28768122 | C | T | 0.75844 | -0.0125919 | 0.00241565 | 1.90E-07 | 0.00488522 | 0.0265923 | 0.854243 |
| --- | --- | --- | --- | --- | --- | --- | --- | --- | --- | --- | --- |
| 114 | average weekly  red wine intake | rs28787109 | A | G | 0.401433 | 0.00621353 | 0.00210542 | 0.0032 | -0.0363619 | 0.0264298 | 0.168887 |
| 115 | average weekly  red wine intake | rs2914860 | T | C | 0.329882 | 0.00149859 | 0.00220031 | 0.5 | -0.0344913 | 0.0242203 | 0.154427 |
| 116 | average weekly  red wine intake | rs2944776 | T | C | 0.061817 | 0.00627335 | 0.00429845 | 0.14 | -0.0504678 | 0.0414167 | 0.22302 |
| 117 | average weekly  red wine intake | rs2962193 | G | A | 0.765358 | 0.00181724 | 0.00243548 | 0.46 | -0.0136382 | 0.0307538 | 0.657431 |
| 118 | average weekly  red wine intake | rs303753 | A | G | 0.344672 | -0.0183206 | 0.0021899 | 6.00E-17 | 0.0244412 | 0.023737 | 0.303167 |
| 119 | average weekly  red wine intake | rs322776 | G | T | 0.581178 | -0.00127624 | 0.00209772 | 0.54 | -0.00827798 | 0.0226587 | 0.714862 |
| 120 | average weekly  red wine intake | rs324012 | T | C | 0.451014 | 0.0015847 | 0.00207321 | 0.44 | -0.00900247 | 0.0227222 | 0.691958 |
| 121 | average weekly  red wine intake | rs34440851 | T | C | 0.15838 | -0.00147927 | 0.00281793 | 0.6 | -0.00784692 | 0.0268722 | 0.770279 |
| 122 | average weekly  red wine intake | rs34473884 | A | G | 0.249941 | 0.00762809 | 0.00238295 | 0.00140001 | 0.00505552 | 0.026915 | 0.851008 |
| 123 | average weekly  red wine intake | rs34631026 | T | C | 0.448374 | 0.0050273 | 0.00207482 | 0.015 | -0.0105175 | 0.0239486 | 0.660538 |
| 124 | average weekly  red wine intake | rs34805485 | A | G | 0.014164 | 0.00418061 | 0.00878972 | 0.630001 | -0.125836 | 0.12033 | 0.295671 |
| 125 | average weekly  red wine intake | rs34811474 | A | G | 0.232314 | 0.00762082 | 0.00244149 | 0.00179999 | -0.0353556 | 0.0269238 | 0.189124 |
| 126 | average weekly  red wine intake | rs35005436 | C | T | 0.157145 | -0.00424767 | 0.00284186 | 0.13 | -0.011989 | 0.0355517 | 0.735946 |
| 127 | average weekly  red wine intake | rs35073053 | C | G | 0.202919 | 0.00158989 | 0.00258517 | 0.54 | 0.0567026 | 0.0307698 | 0.0653582 |
| 128 | average weekly  red wine intake | rs35105141 | T | C | 0.399929 | -0.00324759 | 0.00210492 | 0.12 | -0.00240095 | 0.0234003 | 0.918278 |
| 129 | average weekly  red wine intake | rs35488630 | A | G | 0.177635 | 0.0131037 | 0.00269415 | 1.20E-06 | 0.0196915 | 0.0297045 | 0.507387 |
| 130 | average weekly  red wine intake | rs35589108 | G | A | 0.017687 | 0.0300209 | 0.00782491 | 0.00012 | -0.0537296 | 0.0896386 | 0.548905 |
| 131 | average weekly  red wine intake | rs362307 | T | C | 0.073523 | -0.00832126 | 0.00397769 | 0.0359998 | 0.0649275 | 0.0450681 | 0.149683 |

| 132 | average weekly  red wine intake | rs3783297 | C | T | 0.369444 | -0.0109951 | 0.00214288 | 2.90E-07 | 0.0107087 | 0.0236001 | 0.650005 |
| --- | --- | --- | --- | --- | --- | --- | --- | --- | --- | --- | --- |
| 133 | average weekly  red wine intake | rs3898475 | C | T | 0.178254 | -0.00768281 | 0.00271999 | 0.00470002 | 0.00242294 | 0.0313197 | 0.938336 |
| 134 | average weekly  red wine intake | rs3913960 | C | T | 0.62562 | 0.00979277 | 0.00213039 | 4.30E-06 | -0.0168172 | 0.0227891 | 0.460546 |
| 135 | average weekly  red wine intake | rs3914188 | C | G | 0.734392 | 9.13E-05 | 0.00233449 | 0.97 | 0.0431365 | 0.0241513 | 0.0740832 |
| 136 | average weekly  red wine intake | rs4057919 | T | C | 0.28545 | -0.0085752 | 0.00228908 | 0.000179999 | -0.0196006 | 0.0262794 | 0.455756 |
| 137 | average weekly  red wine intake | rs4135294 | A | G | 0.149535 | 0.0060735 | 0.00292414 | 0.0379997 | -0.00198555 | 0.0335212 | 0.952767 |
| 138 | average weekly  red wine intake | rs4241258 | T | C | 0.136562 | -0.00484998 | 0.00301122 | 0.11 | 0.0741324 | 0.0376403 | 0.0488968 |
| 139 | average weekly  red wine intake | rs4242715 | A | G | 0.682607 | 0.00461535 | 0.00221584 | 0.0369999 | 0.0322088 | 0.0229918 | 0.161249 |
| 140 | average weekly  red wine intake | rs427534 | G | A | 0.413589 | 0.00741442 | 0.00210404 | 0.000430002 | -0.00782732 | 0.0226146 | 0.729253 |
| 141 | average weekly  red wine intake | rs4417025 | A | G | 0.36318 | 0.00215914 | 0.00215456 | 0.32 | -0.0593371 | 0.0269354 | 0.0275988 |
| 142 | average weekly  red wine intake | rs4500930 | T | C | 0.341614 | -0.00407986 | 0.00217233 | 0.0599998 | 0.0544387 | 0.0243421 | 0.0253256 |
| 143 | average weekly  red wine intake | rs4503294 | T | C | 0.563416 | -0.0022181 | 0.00209068 | 0.29 | -0.0235146 | 0.0228413 | 0.303254 |
| 144 | average weekly  red wine intake | rs4532588 | C | T | 0.467043 | 0.00358729 | 0.00206848 | 0.0830004 | 0.00295666 | 0.0230742 | 0.89804 |
| 145 | average weekly  red wine intake | rs461599 | C | A | 0.463873 | 0.00228879 | 0.00206975 | 0.27 | -0.00591379 | 0.022591 | 0.793493 |
| 146 | average weekly  red wine intake | rs4643716 | A | C | 0.583739 | 0.0115308 | 0.00210517 | 4.30E-08 | 0.018002 | 0.0226426 | 0.426584 |
| 147 | average weekly  red wine intake | rs4726481 | T | G | 0.398624 | -0.0172463 | 0.00211506 | 3.50E-16 | 0.044554 | 0.0238323 | 0.0615559 |
| 148 | average weekly  red wine intake | rs4728701 | G | A | 0.952445 | -0.0055906 | 0.00484283 | 0.25 | -0.0459787 | 0.0488621 | 0.346711 |
| 149 | average weekly  red wine intake | rs4739105 | C | T | 0.789004 | -0.00376133 | 0.00255519 | 0.14 | 0.0257942 | 0.0281095 | 0.35881 |
| 150 | average weekly  red wine intake | rs4742659 | G | A | 0.244928 | -0.00180326 | 0.00239498 | 0.450001 | 0.00912658 | 0.0261555 | 0.727138 |

| 151 | average weekly  red wine intake | rs4757370 | A | G | 0.264074 | -0.0109805 | 0.00234018 | 2.70E-06 | 0.0104686 | 0.0244729 | 0.668824 |
| --- | --- | --- | --- | --- | --- | --- | --- | --- | --- | --- | --- |
| 152 | average weekly  red wine intake | rs4811031 | G | A | 0.34729 | -0.00102718 | 0.00216664 | 0.64 | 0.0547366 | 0.023324 | 0.0189361 |
| 153 | average weekly  red wine intake | rs4815366 | T | G | 0.640265 | 0.00190321 | 0.00215157 | 0.38 | -0.0143018 | 0.0245498 | 0.560188 |
| 154 | average weekly  red wine intake | rs4865166 | T | C | 0.275441 | -0.0078153 | 0.00231066 | 0.000719996 | -0.0473867 | 0.0274447 | 0.0842345 |
| 155 | average weekly  red wine intake | rs489062 | A | G | 0.436158 | -0.00336517 | 0.00207912 | 0.11 | 0.0245083 | 0.0227978 | 0.282362 |
| 156 | average weekly  red wine intake | rs4900965 | C | T | 0.30677 | -0.0103348 | 0.00225977 | 4.80E-06 | 0.0354922 | 0.0236904 | 0.13409 |
| 157 | average weekly  red wine intake | rs4916723 | C | A | 0.418804 | -0.0120207 | 0.002113 | 1.30E-08 | -0.00297528 | 0.0226358 | 0.895426 |
| 158 | average weekly  red wine intake | rs4968391 | T | G | 0.677372 | 0.00252132 | 0.00220313 | 0.25 | -0.0183514 | 0.0231812 | 0.428563 |
| 159 | average weekly  red wine intake | rs4982052 | G | A | 0.341433 | 0.00642818 | 0.00218021 | 0.0032 | 0.0352896 | 0.0236763 | 0.136093 |
| 160 | average weekly  red wine intake | rs533143 | C | T | 0.289143 | 0.00174571 | 0.00230549 | 0.450001 | 0.00748736 | 0.0306351 | 0.806918 |
| 161 | average weekly  red wine intake | rs551998 | C | T | 0.367973 | 0.00531076 | 0.00214462 | 0.0129999 | 0.00537583 | 0.02293 | 0.814639 |
| 162 | average weekly  red wine intake | rs56006101 | T | C | 0.019424 | 0.0401881 | 0.00769735 | 1.80E-07 | -0.143078 | 0.071927 | 0.0466778 |
| 163 | average weekly  red wine intake | rs56149652 | G | A | 0.24913 | -0.000561619 | 0.00238532 | 0.81 | -0.021902 | 0.0251478 | 0.383793 |
| 164 | average weekly  red wine intake | rs56194430 | T | C | 0.16815 | -0.00942519 | 0.00278145 | 0.000700003 | -0.00878059 | 0.0343307 | 0.798133 |
| 165 | average weekly  red wine intake | rs56228311 | T | G | 0.343699 | 0.0049838 | 0.00217728 | 0.0219999 | -0.0130673 | 0.0270064 | 0.628485 |
| 166 | average weekly  red wine intake | rs565078 | T | C | 0.484127 | -0.00163397 | 0.00207107 | 0.43 | -0.0252861 | 0.0226985 | 0.265278 |
| 167 | average weekly  red wine intake | rs565522 | C | T | 0.428749 | -0.00480349 | 0.00208699 | 0.021 | 0.0198018 | 0.0225791 | 0.380488 |
| 168 | average weekly  red wine intake | rs568030 | T | G | 0.742928 | 0.0114655 | 0.0023705 | 1.30E-06 | -0.0416929 | 0.0230582 | 0.0705814 |
| 169 | average weekly  red wine intake | rs58905411 | A | G | 0.412645 | 0.00600759 | 0.00209257 | 0.00409996 | 0.00849927 | 0.0227709 | 0.708962 |

| 170 | average weekly  red wine intake | rs59188188 | T | G | 0.288728 | -0.00418154 | 0.00228058 | 0.0669993 | 0.0293951 | 0.024518 | 0.230559 |
| --- | --- | --- | --- | --- | --- | --- | --- | --- | --- | --- | --- |
| 171 | average weekly  red wine intake | rs6030200 | A | G | 0.315626 | 0.00431417 | 0.00222376 | 0.0519996 | -0.000330149 | 0.0229988 | 0.988547 |
| 172 | average weekly  red wine intake | rs6079439 | G | A | 0.529213 | 0.00644574 | 0.00207117 | 0.00189998 | -0.00449776 | 0.0227286 | 0.843131 |
| 173 | average weekly  red wine intake | rs61825452 | T | A | 0.191472 | -0.00391468 | 0.00265499 | 0.14 | -0.014201 | 0.0291549 | 0.626196 |
| 174 | average weekly  red wine intake | rs61873510 | T | G | 0.326022 | -0.000769473 | 0.0022519 | 0.73 | 0.000447365 | 0.0248361 | 0.985629 |
| 175 | average weekly  red wine intake | rs62135521 | T | G | 0.048211 | -0.00509586 | 0.00481156 | 0.29 | 0.0817264 | 0.054859 | 0.136289 |
| 176 | average weekly  red wine intake | rs62182135 | A | C | 0.332384 | 0.00590663 | 0.00218746 | 0.00690001 | 0.01944 | 0.0247126 | 0.43149 |
| 177 | average weekly  red wine intake | rs62271373 | A | T | 0.059409 | -0.00969525 | 0.00444419 | 0.0290001 | 0.0683639 | 0.0555359 | 0.218328 |
| 178 | average weekly  red wine intake | rs62304163 | T | C | 0.042005 | -0.0109396 | 0.00517308 | 0.0340001 | -0.121934 | 0.0617203 | 0.0482014 |
| 179 | average weekly  red wine intake | rs62305780 | G | C | 0.103858 | 0.000305551 | 0.00342788 | 0.93 | -0.0163284 | 0.0327775 | 0.618371 |
| 180 | average weekly  red wine intake | rs62335889 | T | A | 0.267511 | 0.0109489 | 0.00234838 | 3.10E-06 | 0.000446457 | 0.0251097 | 0.985814 |
| 181 | average weekly  red wine intake | rs62339673 | A | C | 0.6257 | -0.00274641 | 0.00214774 | 0.2 | -0.00581749 | 0.0238199 | 0.807054 |
| 182 | average weekly  red wine intake | rs62439319 | T | C | 0.139952 | -0.00702361 | 0.0029722 | 0.0179999 | 0.0116606 | 0.0338513 | 0.730496 |
| 183 | average weekly  red wine intake | rs62466318 | T | C | 0.20441 | 0.00229924 | 0.00256199 | 0.37 | 0.0169523 | 0.0287966 | 0.556068 |
| 184 | average weekly  red wine intake | rs62573521 | T | C | 0.040468 | -0.0323105 | 0.00524921 | 7.50E-10 | 0.13346 | 0.095121 | 0.160599 |
| 185 | average weekly  red wine intake | rs627685 | C | T | 0.302864 | -0.0125352 | 0.00226097 | 3.00E-08 | 0.0268714 | 0.0257046 | 0.295842 |
| 186 | average weekly  red wine intake | rs6427160 | C | T | 0.420258 | -0.00671033 | 0.00208952 | 0.00129999 | 0.00324104 | 0.0227547 | 0.886737 |
| 187 | average weekly  red wine intake | rs6442994 | T | C | 0.774678 | -0.012162 | 0.0024891 | 1.00E-06 | 0.0202617 | 0.0259564 | 0.435034 |
| 188 | average weekly  red wine intake | rs650558 | T | C | 0.247025 | -0.00647292 | 0.00239268 | 0.00680002 | 0.0142324 | 0.0276215 | 0.606366 |

| 189 | average weekly  red wine intake | rs6531648 | C | T | 0.515631 | -0.00286332 | 0.00206056 | 0.16 | -0.0329961 | 0.023154 | 0.154136 |
| --- | --- | --- | --- | --- | --- | --- | --- | --- | --- | --- | --- |
| 190 | average weekly  red wine intake | rs6534293 | G | A | 0.555581 | -0.005433 | 0.00208437 | 0.00909997 | -0.0116389 | 0.0227104 | 0.608307 |
| 191 | average weekly  red wine intake | rs6700839 | A | G | 0.566022 | -0.00564653 | 0.00208862 | 0.00690001 | 0.0463758 | 0.0229 | 0.0428519 |
| 192 | average weekly  red wine intake | rs6727281 | T | C | 0.185409 | 0.000861838 | 0.00266255 | 0.75 | 0.0404027 | 0.0258477 | 0.118028 |
| 193 | average weekly  red wine intake | rs6744640 | G | A | 0.434834 | -0.00542544 | 0.00208615 | 0.00929994 | -0.0296169 | 0.0247124 | 0.230736 |
| 194 | average weekly  red wine intake | rs67822265 | T | C | 0.379956 | -0.0086548 | 0.00213624 | 5.10E-05 | -0.0592622 | 0.0237159 | 0.0124603 |
| 195 | average weekly  red wine intake | rs68024891 | C | T | 0.233943 | 0.00531589 | 0.00245037 | 0.0299999 | 0.017053 | 0.0269845 | 0.527416 |
| 196 | average weekly  red wine intake | rs6810396 | G | C | 0.196637 | 0.00104425 | 0.0025981 | 0.69 | -0.00378634 | 0.02503 | 0.879761 |
| 197 | average weekly  red wine intake | rs6819372 | G | A | 0.534361 | 0.0111728 | 0.00206588 | 6.40E-08 | -0.0495593 | 0.0227944 | 0.0296907 |
| 198 | average weekly  red wine intake | rs6908328 | A | C | 0.514021 | 0.0127108 | 0.00206267 | 7.20E-10 | 0.0118016 | 0.0236151 | 0.617253 |
| 199 | average weekly  red wine intake | rs693458 | C | A | 0.264106 | -0.00388344 | 0.00233904 | 0.0969996 | 0.0381868 | 0.0293511 | 0.193247 |
| 200 | average weekly  red wine intake | rs6978944 | G | A | 0.202502 | -0.0122005 | 0.00257142 | 2.10E-06 | 0.0239569 | 0.0263869 | 0.363928 |
| 201 | average weekly  red wine intake | rs6992604 | C | T | 0.288768 | -0.00523916 | 0.00228719 | 0.0219999 | -0.00357999 | 0.0244465 | 0.883572 |
| 202 | average weekly  red wine intake | rs6993770 | T | A | 0.288225 | 0.00744556 | 0.00227503 | 0.00109999 | 0.0255879 | 0.0272525 | 0.347772 |
| 203 | average weekly  red wine intake | rs7029768 | T | G | 0.129445 | 0.00739412 | 0.00307581 | 0.016 | 0.0159432 | 0.0349405 | 0.648176 |
| 204 | average weekly  red wine intake | rs7046881 | G | T | 0.424242 | 0.0024692 | 0.00209034 | 0.24 | 0.00255594 | 0.025573 | 0.920387 |
| 205 | average weekly  red wine intake | rs71651683 | T | C | 0.014447 | 0.00173818 | 0.00863666 | 0.84 | -0.252724 | 0.135061 | 0.0613197 |
| 206 | average weekly  red wine intake | rs72641050 | C | A | 0.24604 | -0.0043736 | 0.00240305 | 0.0690001 | -0.00714887 | 0.0240957 | 0.766706 |
| 207 | average weekly  red wine intake | rs72642323 | G | T | 0.007891 | 0.0133833 | 0.0116729 | 0.25 | 0.15774 | 0.26794 | 0.556054 |

| 208 | average weekly  red wine intake | rs7277942 | T | C | 0.172968 | 0.0126232 | 0.00273123 | 3.80E-06 | -0.0245781 | 0.0303094 | 0.417419 |
| --- | --- | --- | --- | --- | --- | --- | --- | --- | --- | --- | --- |
| 209 | average weekly  red wine intake | rs72787062 | A | G | 0.163876 | 0.00729492 | 0.0027869 | 0.0089 | -0.0258147 | 0.0319101 | 0.418527 |
| 210 | average weekly  red wine intake | rs728538 | G | T | 0.167308 | -0.0106407 | 0.00277757 | 0.000129999 | 0.0395208 | 0.028947 | 0.172165 |
| 211 | average weekly  red wine intake | rs7302200 | A | G | 0.341698 | 0.00421652 | 0.00217752 | 0.0530005 | -0.018898 | 0.0247451 | 0.445044 |
| 212 | average weekly  red wine intake | rs73050128 | A | C | 0.165397 | 0.00815488 | 0.00277954 | 0.00329997 | 0.0230786 | 0.026384 | 0.381727 |
| 213 | average weekly  red wine intake | rs7330939 | T | C | 0.721525 | 0.00118772 | 0.00232039 | 0.61 | 0.0176287 | 0.0239395 | 0.461497 |
| 214 | average weekly  red wine intake | rs73543290 | A | G | 0.044505 | 0.000464651 | 0.00504183 | 0.93 | -0.102248 | 0.0754584 | 0.175411 |
| 215 | average weekly  red wine intake | rs7357754 | G | A | 0.498888 | -0.0026457 | 0.00206866 | 0.2 | 0.013318 | 0.0226272 | 0.55614 |
| 216 | average weekly  red wine intake | rs7431028 | G | T | 0.525645 | -0.0099514 | 0.00207493 | 1.60E-06 | -0.0112367 | 0.0227373 | 0.621166 |
| 217 | average weekly  red wine intake | rs7433378 | T | C | 0.232435 | 0.0117215 | 0.00248979 | 2.50E-06 | -0.00413364 | 0.0270996 | 0.878765 |
| 218 | average weekly  red wine intake | rs74424378 | G | T | 0.236182 | 0.00282613 | 0.00243244 | 0.25 | 0.00700843 | 0.0256422 | 0.78461 |
| 219 | average weekly  red wine intake | rs74439139 | G | T | 0.090897 | -0.011889 | 0.00368917 | 0.00129999 | -0.052246 | 0.0510335 | 0.30595 |
| 220 | average weekly  red wine intake | rs74567946 | G | T | 0.028667 | 0.0312151 | 0.00624833 | 5.90E-07 | -0.0807796 | 0.0713049 | 0.257266 |
| 221 | average weekly  red wine intake | rs7460106 | C | T | 0.236839 | 0.0113766 | 0.00245957 | 3.70E-06 | 0.0240849 | 0.0258825 | 0.352087 |
| 222 | average weekly  red wine intake | rs74679146 | C | T | 0.075452 | 0.00242551 | 0.00389654 | 0.53 | -0.0222233 | 0.043923 | 0.612884 |
| 223 | average weekly  red wine intake | rs74808805 | T | G | 0.044015 | 0.000610564 | 0.00508542 | 0.9 | -0.0870283 | 0.070264 | 0.215497 |
| 224 | average weekly  red wine intake | rs7514579 | C | A | 0.231458 | -0.00459592 | 0.00245459 | 0.061 | -0.0516689 | 0.0277706 | 0.0628058 |
| 225 | average weekly  red wine intake | rs7546040 | G | C | 0.732216 | 0.0117688 | 0.00232646 | 4.20E-07 | 0.041383 | 0.0259256 | 0.110439 |
| 226 | average weekly  red wine intake | rs7587791 | C | A | 0.514505 | 0.00331965 | 0.0020643 | 0.11 | -0.0148308 | 0.0227979 | 0.515347 |

| 227 | average weekly  red wine intake | rs76082653 | T | C | 0.053765 | -0.0192432 | 0.00457912 | 2.60E-05 | 0.0764467 | 0.0645011 | 0.235938 |
| --- | --- | --- | --- | --- | --- | --- | --- | --- | --- | --- | --- |
| 228 | average weekly  red wine intake | rs7610856 | A | C | 0.431823 | 0.0120494 | 0.00208986 | 8.10E-09 | 0.000137035 | 0.0232062 | 0.995288 |
| 229 | average weekly  red wine intake | rs7616201 | G | A | 0.349242 | 8.31E-05 | 0.00217024 | 0.97 | 0.0116921 | 0.0237306 | 0.622224 |
| 230 | average weekly  red wine intake | rs767870 | A | G | 0.850879 | 0.00591611 | 0.00289471 | 0.0409996 | -0.026274 | 0.0340551 | 0.440401 |
| 231 | average weekly  red wine intake | rs77169096 | A | T | 0.011358 | -0.0509671 | 0.00998914 | 3.40E-07 | 0.0295034 | 0.0790844 | 0.709102 |
| 232 | average weekly  red wine intake | rs77371894 | G | C | 0.089857 | -0.00230518 | 0.00361651 | 0.52 | 0.0269152 | 0.0528736 | 0.610718 |
| 233 | average weekly  red wine intake | rs780569 | A | T | 0.706871 | -0.0033604 | 0.00228712 | 0.14 | -0.0358402 | 0.0267686 | 0.180607 |
| 234 | average weekly  red wine intake | rs7973103 | T | C | 0.194566 | 0.007242 | 0.00260649 | 0.00549997 | -0.0318631 | 0.0379111 | 0.400646 |
| 235 | average weekly  red wine intake | rs7977455 | G | C | 0.760856 | -0.0116503 | 0.0024228 | 1.50E-06 | -0.0280175 | 0.0258675 | 0.278757 |
| 236 | average weekly  red wine intake | rs79942197 | G | C | 0.064674 | 0.00285808 | 0.00420912 | 0.5 | 0.0226945 | 0.064315 | 0.724189 |
| 237 | average weekly  red wine intake | rs80101850 | C | T | 0.038086 | -0.012264 | 0.00544321 | 0.0239999 | -0.0977829 | 0.0553632 | 0.0773606 |
| 238 | average weekly  red wine intake | rs8015726 | C | T | 0.363304 | 0.00648947 | 0.00215706 | 0.00259998 | -0.00121843 | 0.0254575 | 0.961827 |
| 239 | average weekly  red wine intake | rs80214940 | G | A | 0.17861 | 0.00481316 | 0.0027088 | 0.0759994 | 0.0242296 | 0.0297997 | 0.416172 |
| 240 | average weekly  red wine intake | rs80255756 | A | G | 0.062601 | -0.0135819 | 0.00426555 | 0.0015 | 0.0637283 | 0.0853467 | 0.455245 |
| 241 | average weekly  red wine intake | rs80292319 | C | T | 0.058801 | 0.0112861 | 0.00438293 | 0.01 | -0.00267925 | 0.0413441 | 0.94833 |
| 242 | average weekly  red wine intake | rs8030809 | A | G | 0.551842 | -0.0030236 | 0.00208904 | 0.15 | 0.0451347 | 0.0229857 | 0.049577 |
| 243 | average weekly  red wine intake | rs803223 | A | G | 0.74618 | 0.00350363 | 0.00236796 | 0.14 | -0.0148328 | 0.0228983 | 0.517134 |
| 244 | average weekly  red wine intake | rs8043563 | C | G | 0.735542 | -0.00683084 | 0.00236032 | 0.00379997 | 0.0167797 | 0.0254415 | 0.509549 |
| 245 | average weekly  red wine intake | rs8050545 | G | A | 0.111371 | -0.00783841 | 0.00327798 | 0.017 | 0.0241717 | 0.0281981 | 0.39133 |

| 246 | average weekly  red wine intake | rs8083110 | T | C | 0.420394 | 0.00364928 | 0.00209507 | 0.0819993 | 0.0324019 | 0.0226579 | 0.152703 |
| --- | --- | --- | --- | --- | --- | --- | --- | --- | --- | --- | --- |
| 247 | average weekly  red wine intake | rs838145 | A | G | 0.540505 | -0.00334256 | 0.00208049 | 0.11 | -0.00428127 | 0.0237587 | 0.856997 |
| 248 | average weekly  red wine intake | rs874296 | C | T | 0.384517 | -0.000744834 | 0.00212941 | 0.73 | 0.00297073 | 0.0230431 | 0.897421 |
| 249 | average weekly  red wine intake | rs898751 | T | C | 0.49202 | -0.012363 | 0.00206424 | 2.10E-09 | 0.0109225 | 0.022663 | 0.62984 |
| 250 | average weekly  red wine intake | rs900802 | T | C | 0.696232 | 0.000520684 | 0.00225167 | 0.82 | -0.0341132 | 0.0251307 | 0.174645 |
| 251 | average weekly  red wine intake | rs906580 | T | A | 0.684429 | 0.0102247 | 0.00223068 | 4.60E-06 | 0.0114961 | 0.0238715 | 0.630104 |
| 252 | average weekly  red wine intake | rs911475 | T | C | 0.165978 | -0.00762474 | 0.00279962 | 0.00649995 | -0.0148029 | 0.0363634 | 0.683946 |
| 253 | average weekly  red wine intake | rs927769 | A | C | 0.627128 | 0.0101978 | 0.00213328 | 1.80E-06 | -0.00310665 | 0.0230752 | 0.892903 |
| 254 | average weekly  red wine intake | rs9349379 | G | A | 0.407104 | 0.00388657 | 0.00209838 | 0.064 | -0.0176599 | 0.0226143 | 0.434852 |
| 255 | average weekly  red wine intake | rs9350781 | T | A | 0.350198 | -0.00127417 | 0.00217204 | 0.56 | 0.0221849 | 0.023922 | 0.353726 |
| 256 | average weekly  red wine intake | rs9372625 | A | G | 0.383647 | 0.0122444 | 0.00212712 | 8.60E-09 | -0.0218925 | 0.0242832 | 0.367297 |
| 257 | average weekly  red wine intake | rs9403297 | A | G | 0.371193 | -0.00488124 | 0.00213448 | 0.0219999 | 0.0232451 | 0.0246367 | 0.345419 |
| 258 | average weekly  red wine intake | rs9537938 | A | G | 0.683499 | 0.00648939 | 0.00222058 | 0.00350002 | 0.000802024 | 0.0240428 | 0.973389 |
| 259 | average weekly  red wine intake | rs956752 | G | A | 0.099744 | -0.00906027 | 0.00343936 | 0.00840001 | 0.00621876 | 0.0412838 | 0.880264 |
| 260 | average weekly  red wine intake | rs9611953 | G | A | 0.489888 | -0.00697014 | 0.00206421 | 0.000729995 | -0.0242536 | 0.0233914 | 0.299801 |
| 261 | average weekly  red wine intake | rs9648478 | A | G | 0.508609 | -0.00381271 | 0.00206248 | 0.0649995 | 0.0137287 | 0.0225884 | 0.543337 |
| 262 | average weekly  red wine intake | rs9690021 | T | G | 0.489829 | -0.00242605 | 0.00206125 | 0.24 | 0.0325362 | 0.0226643 | 0.151125 |
| 263 | average weekly  red wine intake | rs9728289 | G | A | 0.410572 | -0.00992252 | 0.00209624 | 2.20E-06 | 0.00906541 | 0.0228161 | 0.691129 |
| 264 | average weekly  red wine intake | rs9899357 | C | T | 0.381526 | -0.00982063 | 0.0021334 | 4.20E-06 | 0.0529102 | 0.0237016 | 0.0255918 |

| 265 | average weekly  red wine intake | rs9902512 | G | C | 0.357758 | 0.00178891 | 0.00215245 | 0.41 | -0.014281 | 0.0241189 | 0.553778 |
| --- | --- | --- | --- | --- | --- | --- | --- | --- | --- | --- | --- |
| 266 | average weekly  red wine intake | rs9906502 | A | G | 0.175544 | 0.000489544 | 0.00270605 | 0.86 | 0.0209678 | 0.028654 | 0.464314 |

Table S7 Instrumental variables used in two-sample MR analysis of ICH to red wine.

|  | **Exposure** | **SNP** | **Effect_Allele** | **Other_Allele** | **EAF** | **beta.ICH** | **se.ICH** | **pval.ICH** | **beta.red_wine** | **se.red_wine** | **pval.red_wine** |
| --- | --- | --- | --- | --- | --- | --- | --- | --- | --- | --- | --- |
| 1 | ICH | rs10774020 | C | T | 0.699429 | 0.114669 | 0.0249591 | 4.34E-06 | 0.00395406 | 0.00218978 | 0.0710003 |
| 2 | ICH | rs151139794 | T | C | 0.0474048 | 0.240171 | 0.0500096 | 1.57E-06 | 0.0023546 | 0.00516001 | 0.649999 |
| 3 | ICH | rs16948815 | A | G | 0.0371304 | 0.255333 | 0.0559257 | 4.98E-06 | 0.0032738 | 0.00978181 | 0.74 |
| 4 | ICH | rs1889499 | C | T | 0.112568 | 0.161604 | 0.0344145 | 2.66E-06 | -0.00714354 | 0.00362349 | 0.0490004 |
| 5 | ICH | rs299256 | G | A | 0.482743 | -0.12014 | 0.0225827 | 1.04E-07 | 0.00109545 | 0.00208627 | 0.6 |
| 6 | ICH | rs56031512 | C | G | 0.163303 | 0.136329 | 0.0297171 | 4.48E-06 | -0.00492427 | 0.00319764 | 0.12 |
| 7 | ICH | rs6990315 | G | A | 0.807075 | -0.133922 | 0.0279012 | 1.59E-06 | -0.00616663 | 0.00293772 | 0.0359998 |
| 8 | ICH | rs7162571 | C | A | 0.795804 | -0.124817 | 0.0273291 | 4.94E-06 | -0.0027996 | 0.00250142 | 0.26 |
| 9 | ICH | rs763193 | G | A | 0.412519 | 0.109409 | 0.0227181 | 1.46E-06 | -0.000968332 | 0.00207944 | 0.64 |
| 10 | ICH | rs7657813 | G | T | 0.866747 | -0.172483 | 0.0321071 | 7.78E-08 | -0.00192265 | 0.00334069 | 0.56 |
| 11 | ICH | rs77659329 | G | T | 0.0295181 | -0.363596 | 0.0743475 | 1.01E-06 | -0.00139729 | 0.00830787 | 0.87 |

Table S8 Instrumental variables used in two-sample MR analysis of red wine to hypertension.

|  | **Exposure** | **SNP** | **Effect**  **Allele** | **Other**  **Allele** | **EAF** | **beta.red_wine** | **se.red_wine** | **pval.red_wine** | **beta.hyperte**  **nsion** | **se.hypertensio**  **n** | **pval.hypertension** |
| --- | --- | --- | --- | --- | --- | --- | --- | --- | --- | --- | --- |
| 1 | average weekly  red wine intake | rs10006551 | T | C | 0.539013 | 0.0105641 | 0.0020678 | 3.20E-07 | -0.0150158 | 0.00560807 | 0.00741652 |
| 2 | average weekly  red wine intake | rs10156047 | T | G | 0.138048 | -0.0140078 | 0.00300956 | 3.20E-06 | -0.0121728 | 0.00971015 | 0.209982 |
| 3 | average weekly  red wine intake | rs10210652 | A | G | 0.350016 | -0.010268 | 0.00216574 | 2.10E-06 | -0.00120402 | 0.00576491 | 0.834563 |
| 4 | average weekly  red wine intake | rs10280836 | G | T | 0.771368 | -0.0123121 | 0.00245691 | 5.40E-07 | 0.00323864 | 0.00693327 | 0.640417 |
| 5 | average weekly  red wine intake | rs10822129 | T | C | 0.406521 | 0.01344 | 0.0021284 | 2.70E-10 | -0.0163605 | 0.00564979 | 0.00378234 |
| 6 | average weekly  red wine intake | rs10865093 | C | T | 0.447573 | -0.00959194 | 0.00207867 | 3.90E-06 | 0.00317897 | 0.00558333 | 0.569106 |
| 7 | average weekly  red wine intake | rs10873070 | G | A | 0.624045 | -0.00999457 | 0.00215359 | 3.50E-06 | 0.00490553 | 0.00596612 | 0.410945 |
| 8 | average weekly  red wine intake | rs10925183 | A | G | 0.607433 | -0.011922 | 0.00211168 | 1.60E-08 | 0.00642474 | 0.00572507 | 0.261772 |
| 9 | average weekly  red wine intake | rs11021354 | G | A | 0.393325 | 0.0112243 | 0.0021204 | 1.20E-07 | -0.0101967 | 0.00627506 | 0.104173 |
| 10 | average weekly  red wine intake | rs11024405 | G | A | 0.185291 | -0.0137073 | 0.00266707 | 2.80E-07 | 0.0129372 | 0.00662999 | 0.0510199 |
| 11 | average weekly  red wine intake | rs11114498 | T | C | 0.673867 | -0.0102206 | 0.00220386 | 3.50E-06 | 0.00697261 | 0.00612657 | 0.255082 |
| 12 | average weekly  red wine intake | rs11126576 | T | C | 0.440017 | 0.010927 | 0.00207899 | 1.50E-07 | -0.00950188 | 0.00554052 | 0.0863495 |
| 13 | average weekly  red wine intake | rs11221890 | G | A | 0.148265 | 0.0158665 | 0.00291925 | 5.50E-08 | -0.00172871 | 0.0080709 | 0.830399 |
| 14 | average weekly  red wine intake | rs114313565 | A | G | 0.027269 | 0.0320306 | 0.00653599 | 9.60E-07 | 0.000205832 | 0.0267435 | 0.993859 |
| 15 | average weekly  red wine intake | rs114795493 | A | G | 0.016059 | 0.0393968 | 0.00819179 | 1.50E-06 | -0.0450464 | 0.0345361 | 0.192122 |
| 16 | average weekly  red wine intake | rs115189110 | T | C | 0.041798 | 0.0246877 | 0.00532761 | 3.60E-06 | -0.0186727 | 0.0111455 | 0.0938642 |
| 17 | average weekly  red wine intake | rs11662234 | G | T | 0.524066 | -0.00945495 | 0.00206748 | 4.80E-06 | -0.00354477 | 0.00562323 | 0.528445 |

| 18 | average weekly  red wine intake | rs11705856 | A | G | 0.642632 | 0.01074 | 0.00215133 | 6.00E-07 | 0.00546534 | 0.00566811 | 0.334933 |
| --- | --- | --- | --- | --- | --- | --- | --- | --- | --- | --- | --- |
| 19 | average weekly  red wine intake | rs11714337 | A | G | 0.43305 | 0.0123918 | 0.00208811 | 2.90E-09 | 4.12E-05 | 0.0056867 | 0.994219 |
| 20 | average weekly  red wine intake | rs11715683 | A | T | 0.344906 | 0.013978 | 0.00217352 | 1.30E-10 | 0.00104932 | 0.00583113 | 0.857191 |
| 21 | average weekly  red wine intake | rs117331323 | T | C | 0.025354 | 0.0300717 | 0.00656731 | 4.70E-06 | -0.00329132 | 0.0315893 | 0.917018 |
| 22 | average weekly  red wine intake | rs11877758 | G | T | 0.310973 | -0.0107631 | 0.00223808 | 1.50E-06 | 0.00930129 | 0.00635788 | 0.14348 |
| 23 | average weekly  red wine intake | rs12030413 | C | G | 0.178642 | 0.012622 | 0.00272179 | 3.50E-06 | 0.00614458 | 0.00753386 | 0.414732 |
| 24 | average weekly  red wine intake | rs12043569 | G | C | 0.220931 | 0.0129125 | 0.00248759 | 2.10E-07 | 0.00891903 | 0.00648155 | 0.168802 |
| 25 | average weekly  red wine intake | rs12373827 | A | G | 0.515869 | 0.00957816 | 0.0020731 | 3.80E-06 | 0.00139535 | 0.00556409 | 0.801986 |
| 26 | average weekly  red wine intake | rs12692596 | T | C | 0.370567 | -0.0116782 | 0.0021345 | 4.50E-08 | -0.00073105 | 0.00583939 | 0.90037 |
| 27 | average weekly  red wine intake | rs13034936 | C | T | 0.111522 | -0.0172726 | 0.00327463 | 1.30E-07 | 0.0368957 | 0.0136109 | 0.00671305 |
| 28 | average weekly  red wine intake | rs1364603 | G | A | 0.624999 | -0.0105902 | 0.00214104 | 7.60E-07 | 0.0100196 | 0.00587895 | 0.0883222 |
| 29 | average weekly  red wine intake | rs1441165 | G | A | 0.534631 | -0.010974 | 0.00207298 | 1.20E-07 | -0.00053108 | 0.00557203 | 0.924067 |
| 30 | average weekly  red wine intake | rs1446577 | G | C | 0.256445 | 0.0151328 | 0.00236106 | 1.50E-10 | -0.00879243 | 0.00604824 | 0.146025 |
| 31 | average weekly  red wine intake | rs146821330 | C | T | 0.014265 | -0.0413513 | 0.00873602 | 2.20E-06 | 0.0221944 | 0.0367883 | 0.546307 |
| 32 | average weekly  red wine intake | rs164328 | T | G | 0.912282 | 0.0184492 | 0.00367585 | 5.20E-07 | -0.00739796 | 0.0119912 | 0.537269 |
| 33 | average weekly  red wine intake | rs16989140 | T | C | 0.088544 | -0.0190561 | 0.00364992 | 1.80E-07 | 0.0144803 | 0.00956828 | 0.130187 |
| 34 | average weekly  red wine intake | rs1896297 | T | C | 0.655541 | -0.0118387 | 0.00218034 | 5.60E-08 | 0.0102572 | 0.00587487 | 0.0808221 |
| 35 | average weekly  red wine intake | rs1916414 | G | A | 0.535336 | 0.0100546 | 0.00207381 | 1.20E-06 | -0.0119659 | 0.0055408 | 0.0308035 |
| 36 | average weekly  red wine intake | rs1966836 | G | A | 0.710792 | 0.0123253 | 0.00227216 | 5.80E-08 | -0.00741699 | 0.00605091 | 0.220288 |

| 37 | average weekly  red wine intake | rs2117137 | G | A | 0.405583 | 0.00983136 | 0.0020973 | 2.80E-06 | -0.0008596 | 0.00556599 | 0.877264 |
| --- | --- | --- | --- | --- | --- | --- | --- | --- | --- | --- | --- |
| 38 | average weekly  red wine intake | rs211799 | G | A | 0.949547 | -0.0220773 | 0.00476767 | 3.60E-06 | 0.00250892 | 0.01261 | 0.842293 |
| 39 | average weekly  red wine intake | rs2162277 | T | C | 0.875179 | 0.0146972 | 0.00311722 | 2.40E-06 | -0.00133866 | 0.00914987 | 0.883682 |
| 40 | average weekly  red wine intake | rs2291007 | C | T | 0.570657 | 0.0098601 | 0.00208449 | 2.20E-06 | 0.00277707 | 0.0057988 | 0.632006 |
| 41 | average weekly  red wine intake | rs2511225 | C | A | 0.353774 | -0.0116801 | 0.00215811 | 6.20E-08 | -0.0127038 | 0.00624419 | 0.0419006 |
| 42 | average weekly  red wine intake | rs261046 | A | G | 0.245007 | 0.0111114 | 0.00241422 | 4.20E-06 | 0.00387949 | 0.00598939 | 0.517161 |
| 43 | average weekly  red wine intake | rs2789517 | A | G | 0.868394 | 0.0146578 | 0.00307273 | 1.80E-06 | -0.0110267 | 0.0123088 | 0.370339 |
| 44 | average weekly  red wine intake | rs28768122 | C | T | 0.75844 | -0.0125919 | 0.00241565 | 1.90E-07 | 0.0184864 | 0.00653537 | 0.00467423 |
| 45 | average weekly  red wine intake | rs34198201 | A | C | 0.230866 | 0.0116136 | 0.00244813 | 2.10E-06 | 0.0133947 | 0.00714557 | 0.0608555 |
| 46 | average weekly  red wine intake | rs34863300 | G | T | 0.30968 | -0.0106628 | 0.00223637 | 1.90E-06 | 0.00503513 | 0.00589761 | 0.393239 |
| 47 | average weekly  red wine intake | rs35698271 | C | A | 0.180339 | -0.0153487 | 0.00269709 | 1.30E-08 | -0.0112468 | 0.00754288 | 0.135947 |
| 48 | average weekly  red wine intake | rs3783297 | C | T | 0.369444 | -0.0109951 | 0.00214288 | 2.90E-07 | 0.0163689 | 0.0057909 | 0.0047037 |
| 49 | average weekly  red wine intake | rs3913960 | C | T | 0.62562 | 0.00979277 | 0.00213039 | 4.30E-06 | -0.0137647 | 0.00558945 | 0.0137924 |
| 50 | average weekly  red wine intake | rs4316974 | G | C | 0.834771 | -0.0128804 | 0.00278984 | 3.90E-06 | -0.00423765 | 0.00858793 | 0.6217 |
| 51 | average weekly  red wine intake | rs4643716 | A | C | 0.583739 | 0.0115308 | 0.00210517 | 4.30E-08 | -0.00028423 | 0.00555999 | 0.959229 |
| 52 | average weekly  red wine intake | rs4757370 | A | G | 0.264074 | -0.0109805 | 0.00234018 | 2.70E-06 | -0.00888818 | 0.00600073 | 0.138558 |
| 53 | average weekly  red wine intake | rs4953122 | A | G | 0.665297 | -0.0113859 | 0.00218371 | 1.80E-07 | 0.0034126 | 0.00570966 | 0.550049 |
| 54 | average weekly  red wine intake | rs55968191 | A | G | 0.24686 | 0.0132033 | 0.00241641 | 4.70E-08 | -0.00337194 | 0.00680832 | 0.620412 |
| 55 | average weekly  red wine intake | rs56006101 | T | C | 0.019424 | 0.0401881 | 0.00769735 | 1.80E-07 | 0.0177996 | 0.0175092 | 0.30935 |

| 56 | average weekly  red wine intake | rs60170726 | A | G | 0.142939 | 0.0139683 | 0.00295929 | 2.40E-06 | 0.00333506 | 0.010783 | 0.757103 |
| --- | --- | --- | --- | --- | --- | --- | --- | --- | --- | --- | --- |
| 57 | average weekly  red wine intake | rs61958175 | G | A | 0.047829 | 0.024198 | 0.00482569 | 5.30E-07 | -0.0111168 | 0.0150391 | 0.459788 |
| 58 | average weekly  red wine intake | rs62335889 | T | A | 0.267511 | 0.0109489 | 0.00234838 | 3.10E-06 | 0.00733878 | 0.00613998 | 0.231991 |
| 59 | average weekly  red wine intake | rs62371224 | A | T | 0.065498 | 0.0198083 | 0.00427573 | 3.60E-06 | -0.0104783 | 0.0113978 | 0.357924 |
| 60 | average weekly  red wine intake | rs62573521 | T | C | 0.040468 | -0.0323105 | 0.00524921 | 7.50E-10 | -0.0126568 | 0.0231901 | 0.585213 |
| 61 | average weekly  red wine intake | rs627685 | C | T | 0.302864 | -0.0125352 | 0.00226097 | 3.00E-08 | -0.0125461 | 0.00630869 | 0.0467337 |
| 62 | average weekly  red wine intake | rs6442994 | T | C | 0.774678 | -0.012162 | 0.0024891 | 1.00E-06 | 0.0054312 | 0.00637581 | 0.3943 |
| 63 | average weekly  red wine intake | rs6552079 | T | G | 0.557983 | 0.0112661 | 0.00207789 | 5.90E-08 | 0.00240468 | 0.00559329 | 0.667252 |
| 64 | average weekly  red wine intake | rs6882046 | G | A | 0.268866 | 0.0162857 | 0.00234886 | 4.10E-12 | -0.00325361 | 0.00723694 | 0.65301 |
| 65 | average weekly  red wine intake | rs6908328 | A | C | 0.514021 | 0.0127108 | 0.00206267 | 7.20E-10 | -0.00980635 | 0.00578149 | 0.0898545 |
| 66 | average weekly  red wine intake | rs6978944 | G | A | 0.202502 | -0.0122005 | 0.00257142 | 2.10E-06 | -0.003919 | 0.00647689 | 0.54513 |
| 67 | average weekly  red wine intake | rs713598 | G | C | 0.4001 | -0.0173682 | 0.00210277 | 1.50E-16 | 0.00257857 | 0.00581724 | 0.657575 |
| 68 | average weekly  red wine intake | rs7192601 | T | C | 0.343078 | -0.0107415 | 0.00220792 | 1.10E-06 | 0.00867168 | 0.00565639 | 0.125257 |
| 69 | average weekly  red wine intake | rs7277942 | T | C | 0.172968 | 0.0126232 | 0.00273123 | 3.80E-06 | -0.0187011 | 0.00745359 | 0.0121071 |
| 70 | average weekly  red wine intake | rs73373942 | T | C | 0.148402 | 0.0139803 | 0.00292021 | 1.70E-06 | -0.00093641 | 0.00665126 | 0.888039 |
| 71 | average weekly  red wine intake | rs7431028 | G | T | 0.525645 | -0.0099514 | 0.00207493 | 1.60E-06 | -0.0027226 | 0.00558068 | 0.625648 |
| 72 | average weekly  red wine intake | rs74567946 | G | T | 0.028667 | 0.0312151 | 0.00624833 | 5.90E-07 | -0.00284212 | 0.0175135 | 0.871084 |
| 73 | average weekly  red wine intake | rs7460106 | C | T | 0.236839 | 0.0113766 | 0.00245957 | 3.70E-06 | 0.00609232 | 0.00636719 | 0.338653 |
| 74 | average weekly  red wine intake | rs7546040 | G | C | 0.732216 | 0.0117688 | 0.00232646 | 4.20E-07 | 0.00137795 | 0.00633616 | 0.827839 |

| 75 | average weekly  red wine intake | rs76448834 | G | C | 0.054343 | 0.0213995 | 0.00462355 | 3.70E-06 | 0.0149652 | 0.010889 | 0.169337 |
| --- | --- | --- | --- | --- | --- | --- | --- | --- | --- | --- | --- |
| 76 | average weekly  red wine intake | rs77061979 | A | G | 0.029752 | 0.0281732 | 0.0061489 | 4.60E-06 | 0.0218884 | 0.0241605 | 0.364958 |
| 77 | average weekly  red wine intake | rs77169096 | A | T | 0.011358 | -0.0509671 | 0.00998914 | 3.40E-07 | 0.0171219 | 0.0195001 | 0.379922 |
| 78 | average weekly  red wine intake | rs77719387 | A | T | 0.017634 | -0.0389537 | 0.00832371 | 2.90E-06 | 0.0452447 | 0.0257006 | 0.0783303 |
| 79 | average weekly  red wine intake | rs7977455 | G | C | 0.760856 | -0.0116503 | 0.0024228 | 1.50E-06 | 0.00581602 | 0.00633516 | 0.35859 |
| 80 | average weekly  red wine intake | rs898751 | T | C | 0.49202 | -0.012363 | 0.00206424 | 2.10E-09 | 0.00153878 | 0.00555862 | 0.781912 |
| 81 | average weekly  red wine intake | rs906580 | T | A | 0.684429 | 0.0102247 | 0.00223068 | 4.60E-06 | -0.00911031 | 0.00585528 | 0.119729 |
| 82 | average weekly  red wine intake | rs927769 | A | C | 0.627128 | 0.0101978 | 0.00213328 | 1.80E-06 | -0.00909372 | 0.00565884 | 0.108056 |
| 83 | average weekly  red wine intake | rs9329343 | G | A | 0.45062 | -0.00952777 | 0.00207725 | 4.50E-06 | 0.00366622 | 0.00558733 | 0.511717 |
| 84 | average weekly  red wine intake | rs9585326 | G | A | 0.53352 | 0.0109987 | 0.00207001 | 1.10E-07 | 0.000927682 | 0.00559026 | 0.868199 |
| 85 | average weekly  red wine intake | rs9728289 | G | A | 0.410572 | -0.00992252 | 0.00209624 | 2.20E-06 | -0.00642672 | 0.00558221 | 0.249615 |
| 86 | average weekly  red wine intake | rs9899357 | C | T | 0.381526 | -0.00982063 | 0.0021334 | 4.20E-06 | 0.00222302 | 0.00581849 | 0.702415 |

Table S9 Instrumental variables used in two-sample MR analysis of hypertension to ICH.

|  | **Exposure** | **SNP** | **Effect**  **Allele** | **Other**  **Allele** | **EAF** | **beta.hypertension** | **se.hypertension** | **pval.hypertension** | **beta.ICH** | **se.ICH** | **pval.ICH** |
| --- | --- | --- | --- | --- | --- | --- | --- | --- | --- | --- | --- |
| 1 | hypertension | rs10047462 | T | G | 0.837596 | -0.0508866 | 0.00750972 | 1.23E-11 | 0.00978431 | 0.0306809 | 0.749799 |
| 2 | hypertension | rs10086575 | A | G | 0.29618 | -0.0388945 | 0.00607868 | 1.57E-10 | -0.00104604 | 0.0246825 | 0.966196 |
| 3 | hypertension | rs10149438 | A | G | 0.793091 | 0.0364185 | 0.00686923 | 1.15E-07 | 0.0595264 | 0.0279151 | 0.0329731 |
| 4 | hypertension | rs10153777 | C | A | 0.373046 | -0.0324626 | 0.00574711 | 1.62E-08 | -0.00152906 | 0.0234021 | 0.947904 |
| 5 | hypertension | rs10197337 | C | G | 0.095155 | -0.0469164 | 0.0094953 | 7.77E-07 | -0.0488082 | 0.0385316 | 0.205261 |
| 6 | hypertension | rs10217559 | T | C | 0.693675 | 0.0436658 | 0.00602847 | 4.38E-13 | 0.012048 | 0.0245338 | 0.62337 |
| 7 | hypertension | rs10219559 | C | T | 0.58957 | -0.0462383 | 0.00563531 | 2.30E-16 | -0.0153018 | 0.0229814 | 0.505517 |
| 8 | hypertension | rs1021956 | T | G | 0.85746 | -0.0489933 | 0.00793909 | 6.78E-10 | -0.00729053 | 0.0325524 | 0.822786 |
| 9 | hypertension | rs10255480 | T | C | 0.25918 | 0.0292911 | 0.00635529 | 4.05E-06 | -0.0270548 | 0.0258832 | 0.295901 |
| 10 | hypertension | rs10265221 | C | T | 0.232398 | 0.047612 | 0.0065805 | 4.65E-13 | 0.0356603 | 0.026914 | 0.185181 |
| 11 | hypertension | rs1030688 | C | A | 0.637935 | -0.0306913 | 0.00578869 | 1.15E-07 | 0.00342268 | 0.0236358 | 0.884862 |
| 12 | hypertension | rs10483863 | G | A | 0.6183 | -0.0281506 | 0.00571059 | 8.24E-07 | 0.010911 | 0.0232731 | 0.639196 |
| 13 | hypertension | rs1048565 | T | C | 0.0751084 | -0.0691215 | 0.0105787 | 6.40E-11 | 0.00863625 | 0.0428078 | 0.840116 |
| 14 | hypertension | rs10776752 | T | G | 0.17067 | 0.110898 | 0.00734458 | 1.64E-51 | 0.073476 | 0.0302335 | 0.0150869 |
| 15 | hypertension | rs10778038 | G | T | 0.456153 | 0.0293964 | 0.00558441 | 1.41E-07 | -0.0176655 | 0.0228188 | 0.438835 |
| 16 | hypertension | rs10786736 | C | G | 0.091454 | -0.0906741 | 0.00966809 | 6.68E-21 | -0.0322895 | 0.0393371 | 0.411736 |
| 17 | hypertension | rs10846167 | T | C | 0.240726 | 0.036081 | 0.00647065 | 2.46E-08 | -0.031004 | 0.0264359 | 0.240877 |
| 18 | hypertension | rs10850524 | C | A | 0.678352 | 0.0515285 | 0.00594918 | 4.66E-18 | 0.0667872 | 0.0243586 | 0.00610984 |
| 19 | hypertension | rs10852545 | T | C | 0.222416 | -0.0328115 | 0.00674535 | 1.15E-06 | -0.0326087 | 0.0274258 | 0.234449 |
| 20 | hypertension | rs10863331 | T | C | 0.263615 | 0.0291227 | 0.0062686 | 3.39E-06 | 0.00876707 | 0.0256026 | 0.732027 |
| 21 | hypertension | rs10922477 | A | C | 0.668932 | -0.031736 | 0.0058853 | 6.95E-08 | 0.0159799 | 0.0240167 | 0.505818 |
| 22 | hypertension | rs10935179 | T | G | 0.024749 | -0.100717 | 0.0180356 | 2.35E-08 | 0.0468579 | 0.0730304 | 0.521119 |
| 23 | hypertension | rs10938397 | G | A | 0.473191 | 0.0275558 | 0.0055647 | 7.35E-07 | 0.0256911 | 0.0227304 | 0.258369 |
| 24 | hypertension | rs10957782 | C | A | 0.282318 | -0.032707 | 0.00617277 | 1.17E-07 | 0.00325778 | 0.0251116 | 0.896778 |
| 25 | hypertension | rs11015994 | T | C | 0.24715 | 0.0334656 | 0.00644669 | 2.09E-07 | 0.0501913 | 0.0263652 | 0.0569495 |
| 26 | hypertension | rs11084946 | C | T | 0.883958 | 0.0445748 | 0.00876056 | 3.62E-07 | 0.0146637 | 0.0354641 | 0.679255 |
| 27 | hypertension | rs11105337 | T | A | 0.0770195 | -0.0984481 | 0.0104647 | 5.07E-21 | -0.0547799 | 0.0422504 | 0.194784 |
| 28 | hypertension | rs11122119 | A | C | 0.250496 | -0.0322158 | 0.00641171 | 5.05E-07 | -0.00811213 | 0.0261725 | 0.756599 |
| 29 | hypertension | rs111283598 | A | G | 0.0981566 | 0.0613563 | 0.00934058 | 5.07E-11 | -0.0477314 | 0.0381165 | 0.210479 |
| 30 | hypertension | rs11155026 | A | G | 0.453646 | 0.0265067 | 0.00557933 | 2.03E-06 | 0.0223131 | 0.0227686 | 0.327091 |
| 31 | hypertension | rs11211570 | C | A | 0.117486 | 0.0514549 | 0.00863379 | 2.53E-09 | 0.00214401 | 0.0351284 | 0.951333 |
| 32 | hypertension | rs112285172 | A | C | 0.198984 | -0.0401232 | 0.00695746 | 8.07E-09 | -0.00138013 | 0.0283509 | 0.961174 |
| 33 | hypertension | rs112557008 | A | C | 0.0321081 | 0.079815 | 0.0158613 | 4.85E-07 | -0.0183928 | 0.0645913 | 0.775831 |
| 34 | hypertension | rs112968778 | T | G | 0.0399735 | 0.07934 | 0.0143392 | 3.15E-08 | 0.101665 | 0.058671 | 0.0831304 |
| 35 | hypertension | rs113216630 | A | G | 0.0903502 | -0.0552941 | 0.00973118 | 1.33E-08 | -0.04155 | 0.0395402 | 0.293336 |

| 36 | hypertension | rs1136165 | T | G | 0.680293 | 0.0415645 | 0.00598296 | 3.73E-12 | 0.018062 | 0.0243086 | 0.457464 |
| --- | --- | --- | --- | --- | --- | --- | --- | --- | --- | --- | --- |
| 37 | hypertension | rs113700836 | T | C | 0.195382 | 0.0472003 | 0.00701597 | 1.73E-11 | 0.00574474 | 0.0286881 | 0.841286 |
| 38 | hypertension | rs113928896 | T | C | 0.217856 | -0.0339206 | 0.00676932 | 5.42E-07 | -0.0319272 | 0.0275561 | 0.246609 |
| 39 | hypertension | rs113958593 | C | T | 0.0613285 | -0.0557247 | 0.0116043 | 1.57E-06 | 0.0530868 | 0.0472327 | 0.261037 |
| 40 | hypertension | rs113993899 | T | G | 0.213063 | 0.03154 | 0.00678558 | 3.35E-06 | 0.0114938 | 0.0277879 | 0.679149 |
| 41 | hypertension | rs114850651 | A | G | 0.0391433 | -0.0866755 | 0.0144489 | 1.99E-09 | 0.0205337 | 0.0588987 | 0.727368 |
| 42 | hypertension | rs115197597 | C | T | 0.0198151 | -0.10003 | 0.0202192 | 7.53E-07 | -0.050962 | 0.0806643 | 0.527531 |
| 43 | hypertension | rs115395863 | T | C | 0.0695948 | -0.052311 | 0.0109956 | 1.96E-06 | 0.0745806 | 0.0445834 | 0.0943604 |
| 44 | hypertension | rs115493740 | A | G | 0.105175 | 0.0594345 | 0.00902954 | 4.63E-11 | 0.0231174 | 0.037032 | 0.532461 |
| 45 | hypertension | rs1159201 | G | A | 0.249395 | -0.0441236 | 0.00641825 | 6.21E-12 | -0.0261822 | 0.0260725 | 0.315277 |
| 46 | hypertension | rs11600607 | G | A | 0.154694 | 0.0484099 | 0.00767205 | 2.79E-10 | 4.77E-05 | 0.0313884 | 0.998788 |
| 47 | hypertension | rs116037790 | A | G | 0.0867534 | 0.0482761 | 0.00994778 | 1.22E-06 | 0.0520653 | 0.0406818 | 0.20061 |
| 48 | hypertension | rs116054732 | A | C | 0.0170545 | -0.124072 | 0.0216914 | 1.07E-08 | -0.111136 | 0.0875753 | 0.20443 |
| 49 | hypertension | rs116099394 | T | G | 0.145922 | -0.0544414 | 0.00786738 | 4.52E-12 | 0.0145454 | 0.0318873 | 0.648281 |
| 50 | hypertension | rs11645355 | A | G | 0.292538 | 0.038118 | 0.00610426 | 4.25E-10 | 0.0103326 | 0.02492 | 0.678413 |
| 51 | hypertension | rs116487283 | T | A | 0.0394647 | 0.0655113 | 0.0142263 | 4.13E-06 | 0.0431311 | 0.0582441 | 0.458982 |
| 52 | hypertension | rs116524218 | T | C | 0.0260743 | -0.088724 | 0.0176178 | 4.75E-07 | -0.0276906 | 0.0712631 | 0.697596 |
| 53 | hypertension | rs11652684 | G | T | 0.210873 | -0.0385297 | 0.00684251 | 1.79E-08 | -0.0391197 | 0.0278169 | 0.159626 |
| 54 | hypertension | rs11667341 | T | C | 0.0332421 | -0.0941196 | 0.0157063 | 2.07E-09 | 0.0354466 | 0.0633467 | 0.575777 |
| 55 | hypertension | rs11695165 | C | T | 0.65078 | 0.0268676 | 0.00582765 | 4.02E-06 | 0.0242651 | 0.0237631 | 0.307195 |
| 56 | hypertension | rs117107123 | G | A | 0.0558582 | -0.058577 | 0.0121424 | 1.41E-06 | 0.00858395 | 0.0492694 | 0.861689 |
| 57 | hypertension | rs117529466 | A | G | 0.0105334 | -0.129668 | 0.0276784 | 2.80E-06 | -0.0507305 | 0.108936 | 0.641437 |
| 58 | hypertension | rs117539635 | G | A | 0.0166788 | -0.117574 | 0.0220849 | 1.02E-07 | -0.0442022 | 0.089084 | 0.619764 |
| 59 | hypertension | rs11760230 | G | C | 0.249469 | -0.0371803 | 0.00643246 | 7.47E-09 | -0.0328243 | 0.026172 | 0.209778 |
| 60 | hypertension | rs117778193 | C | T | 0.101244 | 0.0798916 | 0.00918689 | 3.43E-18 | 0.0389316 | 0.0377803 | 0.302788 |
| 61 | hypertension | rs117791044 | C | G | 0.0696013 | -0.0595958 | 0.0110115 | 6.23E-08 | 0.0834443 | 0.0446255 | 0.0615007 |
| 62 | hypertension | rs11782482 | A | G | 0.314016 | -0.0307649 | 0.00601749 | 3.18E-07 | -0.0234088 | 0.0245238 | 0.339811 |
| 63 | hypertension | rs11785859 | G | A | 0.265975 | -0.0358766 | 0.00627848 | 1.10E-08 | -0.00908243 | 0.0255823 | 0.722568 |
| 64 | hypertension | rs11871955 | T | C | 0.798979 | -0.0435556 | 0.00690826 | 2.88E-10 | 0.0251548 | 0.0282162 | 0.372661 |
| 65 | hypertension | rs11904241 | G | A | 0.207396 | 0.0314531 | 0.00682762 | 4.09E-06 | -0.00242425 | 0.0279487 | 0.930879 |
| 66 | hypertension | rs11973114 | A | G | 0.241683 | -0.0433522 | 0.0064964 | 2.50E-11 | 0.0208436 | 0.026458 | 0.430814 |
| 67 | hypertension | rs12024932 | G | A | 0.0458069 | -0.0641616 | 0.0133859 | 1.64E-06 | 0.035175 | 0.054016 | 0.51492 |
| 68 | hypertension | rs12035921 | C | A | 0.405215 | 0.031536 | 0.0056563 | 2.47E-08 | 0.0034404 | 0.0230798 | 0.881502 |
| 69 | hypertension | rs12142891 | C | T | 0.125323 | -0.0389571 | 0.00836587 | 3.21E-06 | 0.029582 | 0.033982 | 0.384017 |
| 70 | hypertension | rs12262271 | T | C | 0.0894078 | -0.0498202 | 0.00978822 | 3.58E-07 | 0.0166027 | 0.0396557 | 0.675456 |
| 71 | hypertension | rs12375937 | C | G | 0.0889566 | 0.0509693 | 0.00972028 | 1.57E-07 | -0.0179833 | 0.0398994 | 0.652194 |
| 72 | hypertension | rs12456880 | C | T | 0.233977 | 0.0306711 | 0.00654059 | 2.74E-06 | 0.0268851 | 0.0266859 | 0.313712 |
| 73 | hypertension | rs12457876 | G | A | 0.310136 | 0.0312013 | 0.00600595 | 2.05E-07 | 0.0127834 | 0.0245107 | 0.601987 |

| 74 | hypertension | rs1248048 | G | C | 0.665045 | 0.0273778 | 0.00589467 | 3.41E-06 | -0.0181022 | 0.0240457 | 0.451555 |
| --- | --- | --- | --- | --- | --- | --- | --- | --- | --- | --- | --- |
| 75 | hypertension | rs12481717 | A | G | 0.194129 | 0.0486211 | 0.00704323 | 5.08E-12 | 0.00196245 | 0.0287927 | 0.94566 |
| 76 | hypertension | rs1248670 | G | A | 0.612942 | 0.0289457 | 0.0056936 | 3.70E-07 | -0.0575683 | 0.0231695 | 0.0129676 |
| 77 | hypertension | rs12503104 | T | C | 0.44656 | -0.0312802 | 0.00560934 | 2.45E-08 | -0.0087128 | 0.0228253 | 0.702671 |
| 78 | hypertension | rs12535256 | C | T | 0.416253 | 0.025727 | 0.00561454 | 4.60E-06 | 0.0242016 | 0.0229319 | 0.291258 |
| 79 | hypertension | rs12616641 | A | C | 0.212695 | -0.0361241 | 0.0067904 | 1.04E-07 | -0.0248012 | 0.0275634 | 0.368233 |
| 80 | hypertension | rs1265002 | T | A | 0.952634 | -0.0924509 | 0.0130042 | 1.17E-12 | -0.0278008 | 0.053853 | 0.605689 |
| 81 | hypertension | rs12656497 | C | T | 0.585946 | 0.0571764 | 0.00562317 | 2.75E-24 | 0.023184 | 0.0228915 | 0.311168 |
| 82 | hypertension | rs12659998 | T | C | 0.0953799 | -0.0481152 | 0.00946201 | 3.67E-07 | -0.0127062 | 0.0384093 | 0.740788 |
| 83 | hypertension | rs12749040 | A | G | 0.0815262 | 0.0664122 | 0.0101084 | 5.03E-11 | 0.0596195 | 0.0413413 | 0.149266 |
| 84 | hypertension | rs12771120 | C | A | 0.228449 | -0.038429 | 0.00661766 | 6.36E-09 | -0.0239014 | 0.0269465 | 0.375081 |
| 85 | hypertension | rs12828438 | G | A | 0.533871 | -0.0426247 | 0.00558324 | 2.27E-14 | 0.0116382 | 0.0228056 | 0.609827 |
| 86 | hypertension | rs12887521 | A | C | 0.653295 | -0.0290373 | 0.00582706 | 6.25E-07 | -0.0272397 | 0.0237519 | 0.251446 |
| 87 | hypertension | rs13064931 | T | C | 0.654142 | -0.0403062 | 0.00581871 | 4.30E-12 | -0.000857812 | 0.0237088 | 0.971138 |
| 88 | hypertension | rs13070418 | G | T | 0.63312 | 0.0276962 | 0.00574772 | 1.45E-06 | 0.0581276 | 0.0234145 | 0.0130446 |
| 89 | hypertension | rs13084174 | T | C | 0.0418244 | 0.0643465 | 0.0139312 | 3.86E-06 | 0.00518585 | 0.0570311 | 0.927548 |
| 90 | hypertension | rs13109690 | G | T | 0.165585 | -0.0450511 | 0.00748414 | 1.75E-09 | 0.0256313 | 0.0304483 | 0.399901 |
| 91 | hypertension | rs13112725 | C | G | 0.828729 | 0.0545831 | 0.00739814 | 1.61E-13 | 0.0834376 | 0.0306071 | 0.006409 |
| 92 | hypertension | rs13157043 | A | C | 0.363003 | -0.0269225 | 0.00581621 | 3.68E-06 | -0.00565241 | 0.0237091 | 0.811566 |
| 93 | hypertension | rs1317181 | T | G | 0.20937 | 0.0661358 | 0.00680458 | 2.49E-22 | 0.0267913 | 0.0278971 | 0.336874 |
| 94 | hypertension | rs1330298 | G | A | 0.21315 | 0.0345086 | 0.00676781 | 3.42E-07 | 0.0378301 | 0.0276379 | 0.17107 |
| 95 | hypertension | rs1345697 | C | T | 0.452498 | 0.0263513 | 0.00557561 | 2.29E-06 | 0.0262672 | 0.0227477 | 0.248206 |
| 96 | hypertension | rs1350006 | G | A | 0.590595 | -0.0277629 | 0.00563738 | 8.45E-07 | 0.0076466 | 0.0230109 | 0.73966 |
| 97 | hypertension | rs141503193 | C | T | 0.0323427 | 0.0886507 | 0.0157556 | 1.84E-08 | -0.0140909 | 0.0655719 | 0.829851 |
| 98 | hypertension | rs1417922 | G | A | 0.588228 | 0.030261 | 0.00563086 | 7.70E-08 | 0.0373889 | 0.0229224 | 0.102868 |
| 99 | hypertension | rs142391093 | G | A | 0.287331 | -0.0370309 | 0.00616627 | 1.91E-09 | -0.0528448 | 0.0251222 | 0.0354209 |
| 100 | hypertension | rs143011617 | G | T | 0.0359491 | -0.0730559 | 0.015297 | 1.79E-06 | -0.0084141 | 0.0617264 | 0.891574 |
| 101 | hypertension | rs143466522 | A | G | 0.0145781 | 0.172396 | 0.0230063 | 6.71E-14 | 0.117464 | 0.0959563 | 0.2209 |
| 102 | hypertension | rs1436138 | G | A | 0.355854 | -0.0369764 | 0.00580825 | 1.94E-10 | 0.0510901 | 0.0236862 | 0.0310092 |
| 103 | hypertension | rs1454651 | G | A | 0.685523 | 0.0350299 | 0.00600617 | 5.47E-09 | -0.042551 | 0.0244112 | 0.0813167 |
| 104 | hypertension | rs146847723 | C | T | 0.141162 | 0.0450951 | 0.00798309 | 1.62E-08 | -0.0144525 | 0.0326566 | 0.658085 |
| 105 | hypertension | rs1509092 | T | G | 0.402296 | -0.0263484 | 0.00575208 | 4.63E-06 | -0.00243223 | 0.0234136 | 0.917264 |
| 106 | hypertension | rs1515114 | G | A | 0.429036 | 0.0354524 | 0.00562047 | 2.83E-10 | 0.00590843 | 0.022971 | 0.797015 |
| 107 | hypertension | rs1526688 | T | C | 0.646446 | 0.0270963 | 0.00580138 | 3.00E-06 | 0.0623135 | 0.0237286 | 0.00863734 |
| 108 | hypertension | rs1571217 | T | G | 0.627116 | 0.0364801 | 0.005751 | 2.25E-10 | 0.024363 | 0.0234009 | 0.297822 |
| 109 | hypertension | rs164010 | T | C | 0.297144 | -0.0283759 | 0.00607723 | 3.02E-06 | -0.0253656 | 0.0247079 | 0.304601 |
| 110 | hypertension | rs167479 | G | T | 0.576059 | 0.0763138 | 0.00563427 | 8.53E-42 | 0.0295931 | 0.0230076 | 0.198363 |
| 111 | hypertension | rs16849273 | G | A | 0.10487 | -0.0601118 | 0.00907174 | 3.44E-11 | 0.0149586 | 0.0368553 | 0.684837 |

| 112 | hypertension | rs16867335 | T | C | 0.280062 | 0.0417151 | 0.0061568 | 1.24E-11 | -0.0144258 | 0.0252004 | 0.567022 |
| --- | --- | --- | --- | --- | --- | --- | --- | --- | --- | --- | --- |
| 113 | hypertension | rs16971384 | G | A | 0.254079 | -0.0317556 | 0.00641702 | 7.47E-07 | 0.0122355 | 0.0261267 | 0.639558 |
| 114 | hypertension | rs16998073 | T | A | 0.31337 | 0.0908867 | 0.00598253 | 3.99E-52 | 0.0196968 | 0.0244668 | 0.420795 |
| 115 | hypertension | rs17008861 | T | C | 0.0673953 | -0.0515803 | 0.011235 | 4.41E-06 | 0.0276458 | 0.0454199 | 0.542741 |
| 116 | hypertension | rs17010957 | C | T | 0.152678 | 0.0494816 | 0.00771126 | 1.39E-10 | 0.0266261 | 0.0317128 | 0.401132 |
| 117 | hypertension | rs17080102 | C | G | 0.0762934 | -0.106142 | 0.0104944 | 4.78E-24 | -0.0147521 | 0.0424008 | 0.727899 |
| 118 | hypertension | rs17096452 | T | A | 0.18121 | 0.0347322 | 0.00717904 | 1.31E-06 | -0.00827151 | 0.0292797 | 0.77756 |
| 119 | hypertension | rs17174870 | T | C | 0.375509 | -0.0301499 | 0.00573431 | 1.46E-07 | 0.00188163 | 0.0233508 | 0.935775 |
| 120 | hypertension | rs17446306 | C | T | 0.0503453 | 0.0591158 | 0.0127033 | 3.26E-06 | 0.0209402 | 0.0520478 | 0.687443 |
| 121 | hypertension | rs17584100 | T | C | 0.11796 | 0.0399348 | 0.00861001 | 3.51E-06 | 0.0353869 | 0.0353318 | 0.316556 |
| 122 | hypertension | rs17624723 | A | G | 0.117473 | 0.047138 | 0.00859086 | 4.09E-08 | -0.00172876 | 0.0350565 | 0.960669 |
| 123 | hypertension | rs17807723 | A | G | 0.1904 | -0.0349282 | 0.00708453 | 8.21E-07 | -0.00103975 | 0.0287375 | 0.971138 |
| 124 | hypertension | rs1799831 | T | C | 0.19231 | 0.0324216 | 0.00702413 | 3.92E-06 | -0.0153762 | 0.0286889 | 0.591984 |
| 125 | hypertension | rs180957609 | C | A | 0.0343158 | -0.0840343 | 0.0154355 | 5.20E-08 | 0.0265354 | 0.0618803 | 0.668056 |
| 126 | hypertension | rs1819074 | A | G | 0.666179 | -0.03388 | 0.0058744 | 8.05E-09 | -0.0213872 | 0.0239003 | 0.370866 |
| 127 | hypertension | rs184382033 | C | G | 0.0335557 | -0.0813981 | 0.0155395 | 1.62E-07 | 0.0494345 | 0.0627245 | 0.430626 |
| 128 | hypertension | rs1848797 | G | A | 0.358009 | -0.0336035 | 0.00579998 | 6.88E-09 | -0.0238496 | 0.023614 | 0.312506 |
| 129 | hypertension | rs185317789 | T | G | 0.03091 | -0.0773821 | 0.016402 | 2.38E-06 | -0.0295988 | 0.0667244 | 0.657334 |
| 130 | hypertension | rs188155432 | T | C | 0.0238195 | 0.151697 | 0.0181544 | 6.49E-17 | -0.00515311 | 0.0747352 | 0.945028 |
| 131 | hypertension | rs188460025 | T | C | 0.0698814 | -0.0599068 | 0.0109638 | 4.65E-08 | -0.0631942 | 0.0443803 | 0.154469 |
| 132 | hypertension | rs1888693 | A | G | 0.386136 | 0.043097 | 0.00569582 | 3.84E-14 | 0.00242157 | 0.0233068 | 0.917249 |
| 133 | hypertension | rs193686 | T | C | 0.719163 | -0.0343117 | 0.00617661 | 2.77E-08 | -0.0284639 | 0.0252047 | 0.258766 |
| 134 | hypertension | rs1952651 | C | A | 0.390936 | -0.0425696 | 0.00567867 | 6.56E-14 | -0.0214778 | 0.0231317 | 0.353147 |
| 135 | hypertension | rs198833 | A | G | 0.889222 | -0.0691875 | 0.0087913 | 3.55E-15 | -0.0648506 | 0.0361786 | 0.0730516 |
| 136 | hypertension | rs1996097 | C | T | 0.378903 | -0.0330993 | 0.00572078 | 7.22E-09 | 0.0136919 | 0.0232769 | 0.556386 |
| 137 | hypertension | rs2005873 | T | C | 0.552221 | -0.0297583 | 0.0055795 | 9.63E-08 | 0.0229234 | 0.0227431 | 0.31349 |
| 138 | hypertension | rs200779507 | A | T | 0.10131 | -0.0460567 | 0.00931009 | 7.54E-07 | -0.0246549 | 0.0378235 | 0.514504 |
| 139 | hypertension | rs2021807 | C | T | 0.383731 | 0.0301987 | 0.00571246 | 1.25E-07 | 0.0022946 | 0.0233459 | 0.921704 |
| 140 | hypertension | rs2030291 | T | A | 0.333644 | 0.0293645 | 0.00589011 | 6.18E-07 | -0.0139982 | 0.0239792 | 0.559378 |
| 141 | hypertension | rs2119149 | C | T | 0.118125 | -0.0467905 | 0.00867323 | 6.86E-08 | 0.00291134 | 0.035318 | 0.934303 |
| 142 | hypertension | rs2120832 | G | C | 0.960679 | -0.0796916 | 0.0141826 | 1.92E-08 | 0.0324412 | 0.0585828 | 0.579738 |
| 143 | hypertension | rs2206815 | A | C | 0.4375 | -0.0290889 | 0.00562014 | 2.27E-07 | -0.00787528 | 0.0229022 | 0.730947 |
| 144 | hypertension | rs222852 | G | A | 0.395352 | -0.0512171 | 0.00567263 | 1.74E-19 | 0.00132656 | 0.0230766 | 0.954159 |
| 145 | hypertension | rs2230738 | T | C | 0.309959 | 0.0392275 | 0.00601425 | 6.92E-11 | 0.0151979 | 0.0245499 | 0.535877 |
| 146 | hypertension | rs2274224 | C | G | 0.344404 | -0.0481519 | 0.00585595 | 1.99E-16 | -0.0285382 | 0.0238956 | 0.232367 |
| 147 | hypertension | rs2278997 | A | G | 0.217805 | 0.0465073 | 0.00669503 | 3.74E-12 | -0.00570164 | 0.0272797 | 0.834443 |
| 148 | hypertension | rs2286526 | T | C | 0.71288 | 0.0473319 | 0.00613665 | 1.23E-14 | 0.0399494 | 0.0249317 | 0.109078 |
| 149 | hypertension | rs2287696 | A | G | 0.223626 | 0.0417987 | 0.00663644 | 3.01E-10 | 0.0434826 | 0.0271364 | 0.109074 |

| 150 | hypertension | rs2290931 | C | T | 0.492037 | 0.0300945 | 0.00556912 | 6.52E-08 | 0.0156806 | 0.0226796 | 0.489318 |
| --- | --- | --- | --- | --- | --- | --- | --- | --- | --- | --- | --- |
| 151 | hypertension | rs2291434 | T | G | 0.439383 | -0.0328848 | 0.00560829 | 4.53E-09 | -0.0136028 | 0.0228287 | 0.551267 |
| 152 | hypertension | rs2367805 | C | A | 0.0264258 | -0.0891304 | 0.0174839 | 3.44E-07 | 0.011609 | 0.071131 | 0.870356 |
| 153 | hypertension | rs2400650 | T | G | 0.071659 | -0.0522396 | 0.0108841 | 1.59E-06 | -0.00249653 | 0.0438883 | 0.954638 |
| 154 | hypertension | rs2447937 | G | A | 0.47965 | 0.0263655 | 0.00554675 | 2.00E-06 | 0.00277514 | 0.022589 | 0.902223 |
| 155 | hypertension | rs2464859 | A | G | 0.167647 | 0.0365573 | 0.00745306 | 9.34E-07 | 0.0512491 | 0.0303868 | 0.0916875 |
| 156 | hypertension | rs2465275 | A | G | 0.127245 | 0.062151 | 0.00830214 | 7.09E-14 | 0.0407078 | 0.0341281 | 0.232949 |
| 157 | hypertension | rs2483912 | C | T | 0.500339 | -0.0305752 | 0.00559086 | 4.53E-08 | -0.0162993 | 0.0228345 | 0.475351 |
| 158 | hypertension | rs2493292 | T | C | 0.109419 | 0.0556887 | 0.00886678 | 3.37E-10 | 0.0296409 | 0.0362667 | 0.413756 |
| 159 | hypertension | rs2515236 | G | A | 0.391391 | 0.0396605 | 0.00567087 | 2.68E-12 | 0.00551671 | 0.0231281 | 0.811472 |
| 160 | hypertension | rs2515432 | C | T | 0.487468 | 0.0268203 | 0.00556032 | 1.41E-06 | -0.018834 | 0.0226161 | 0.404975 |
| 161 | hypertension | rs2549805 | A | T | 0.807865 | 0.0575964 | 0.00707679 | 3.99E-16 | 0.0117063 | 0.0287201 | 0.683568 |
| 162 | hypertension | rs2569882 | C | T | 0.375753 | -0.03046 | 0.00575028 | 1.18E-07 | -0.0377675 | 0.0234527 | 0.107317 |
| 163 | hypertension | rs2577259 | G | A | 0.0938332 | 0.0440059 | 0.00954867 | 4.05E-06 | 0.0494676 | 0.0390912 | 0.205713 |
| 164 | hypertension | rs2643826 | T | C | 0.404474 | 0.0491146 | 0.00565607 | 3.84E-18 | 0.016939 | 0.0230925 | 0.463237 |
| 165 | hypertension | rs2699803 | C | T | 0.535158 | 0.0379849 | 0.00558246 | 1.02E-11 | 0.00560163 | 0.0227556 | 0.805554 |
| 166 | hypertension | rs270952 | G | T | 0.792203 | 0.0345608 | 0.00683025 | 4.19E-07 | -0.0297698 | 0.0276373 | 0.281408 |
| 167 | hypertension | rs2782980 | C | T | 0.654146 | 0.0612701 | 0.00583387 | 8.41E-26 | 0.0376741 | 0.0237942 | 0.113345 |
| 168 | hypertension | rs2823140 | A | G | 0.383609 | 0.0310756 | 0.00573372 | 5.97E-08 | -0.0116289 | 0.0234146 | 0.619435 |
| 169 | hypertension | rs28361576 | A | G | 0.0350801 | 0.0780615 | 0.0150367 | 2.09E-07 | -0.0339119 | 0.0622132 | 0.58569 |
| 170 | hypertension | rs28455998 | A | T | 0.362762 | 0.0386521 | 0.00578216 | 2.31E-11 | -0.0425975 | 0.0236 | 0.0710788 |
| 171 | hypertension | rs28503652 | A | G | 0.361984 | 0.0273141 | 0.0058688 | 3.25E-06 | -0.0192022 | 0.0239093 | 0.421902 |
| 172 | hypertension | rs2866278 | A | G | 0.263595 | 0.0383704 | 0.0063208 | 1.28E-09 | -0.0220237 | 0.0257982 | 0.393277 |
| 173 | hypertension | rs2869499 | C | G | 0.19723 | -0.0368453 | 0.00698477 | 1.33E-07 | 0.0547953 | 0.0283424 | 0.0531949 |
| 174 | hypertension | rs28708716 | T | G | 0.339818 | 0.0299926 | 0.00585655 | 3.04E-07 | 0.00443194 | 0.0238959 | 0.852861 |
| 175 | hypertension | rs28722142 | A | T | 0.0525183 | 0.0603274 | 0.0124027 | 1.15E-06 | -0.012914 | 0.0507509 | 0.799142 |
| 176 | hypertension | rs2876595 | C | T | 0.695379 | -0.0291501 | 0.00602381 | 1.30E-06 | -0.0147958 | 0.0246043 | 0.547607 |
| 177 | hypertension | rs28795514 | C | T | 0.256483 | 0.0314902 | 0.00634162 | 6.85E-07 | 0.0514897 | 0.0259196 | 0.0469764 |
| 178 | hypertension | rs2884768 | T | C | 0.488376 | 0.026118 | 0.0055617 | 2.65E-06 | -0.00291106 | 0.0227354 | 0.898117 |
| 179 | hypertension | rs28879116 | T | C | 0.224304 | -0.0340122 | 0.00703529 | 1.33E-06 | -0.0197723 | 0.028647 | 0.490065 |
| 180 | hypertension | rs2888854 | A | G | 0.681651 | -0.0396636 | 0.0059742 | 3.16E-11 | -0.0130872 | 0.0244051 | 0.591786 |
| 181 | hypertension | rs289033 | G | A | 0.592826 | -0.0314672 | 0.00564683 | 2.51E-08 | -0.029477 | 0.0230091 | 0.200158 |
| 182 | hypertension | rs2966083 | T | G | 0.58653 | -0.0317757 | 0.00564675 | 1.83E-08 | -0.0477929 | 0.0230077 | 0.0377781 |
| 183 | hypertension | rs297935 | G | A | 0.762684 | 0.0304125 | 0.00653191 | 3.22E-06 | 0.0472416 | 0.0265652 | 0.0753494 |
| 184 | hypertension | rs303762 | C | T | 0.35071 | 0.0348006 | 0.00581202 | 2.13E-09 | 0.0245186 | 0.0236992 | 0.300867 |
| 185 | hypertension | rs303948 | G | A | 0.100261 | 0.0541811 | 0.00921914 | 4.18E-09 | 0.00941597 | 0.0376472 | 0.802502 |
| 186 | hypertension | rs3211166 | G | A | 0.291135 | 0.0290481 | 0.00611612 | 2.04E-06 | 0.00152247 | 0.0249359 | 0.951315 |
| 187 | hypertension | rs34004783 | G | C | 0.593582 | -0.0819357 | 0.00564881 | 1.13E-47 | -0.0543517 | 0.0230877 | 0.0185656 |

| 188 | hypertension | rs34063976 | G | A | 0.315882 | -0.0288764 | 0.00596945 | 1.32E-06 | 0.0229516 | 0.0243206 | 0.345318 |
| --- | --- | --- | --- | --- | --- | --- | --- | --- | --- | --- | --- |
| 189 | hypertension | rs34634092 | C | T | 0.169575 | 0.0345097 | 0.00739925 | 3.10E-06 | 0.0212427 | 0.0301407 | 0.480946 |
| 190 | hypertension | rs34811474 | A | G | 0.22937 | -0.0325274 | 0.00663571 | 9.49E-07 | -0.0353556 | 0.0269238 | 0.189124 |
| 191 | hypertension | rs34869093 | G | A | 0.356567 | 0.0310422 | 0.00579788 | 8.60E-08 | -0.0132907 | 0.0236556 | 0.574225 |
| 192 | hypertension | rs34924059 | T | G | 0.153908 | 0.0430756 | 0.00767237 | 1.97E-08 | -0.010762 | 0.0314489 | 0.732197 |
| 193 | hypertension | rs35198836 | T | C | 0.321464 | 0.0436478 | 0.00593951 | 2.00E-13 | 0.00390412 | 0.0242543 | 0.87212 |
| 194 | hypertension | rs35346340 | C | G | 0.270698 | 0.0718642 | 0.00624686 | 1.26E-30 | -0.0136925 | 0.0255937 | 0.592654 |
| 195 | hypertension | rs35427 | G | T | 0.365322 | -0.0485854 | 0.00581385 | 6.44E-17 | -0.0458422 | 0.0236651 | 0.0527303 |
| 196 | hypertension | rs35783704 | A | G | 0.154979 | -0.0593618 | 0.00770013 | 1.27E-14 | 0.00123348 | 0.0310838 | 0.968347 |
| 197 | hypertension | rs35784342 | T | C | 0.578232 | 0.036368 | 0.0056696 | 1.41E-10 | 0.0758135 | 0.0231247 | 0.00104376 |
| 198 | hypertension | rs35855137 | A | G | 0.0309185 | -0.0825229 | 0.0162381 | 3.73E-07 | -0.0573515 | 0.0657802 | 0.383282 |
| 199 | hypertension | rs3735533 | C | T | 0.904966 | 0.120761 | 0.00959477 | 2.52E-36 | 0.0745243 | 0.0386418 | 0.0537811 |
| 200 | hypertension | rs3740051 | G | A | 0.130118 | 0.0454353 | 0.00821886 | 3.24E-08 | 0.0540499 | 0.0335987 | 0.107683 |
| 201 | hypertension | rs3748618 | A | G | 0.361724 | -0.0274447 | 0.00577813 | 2.04E-06 | 0.0339314 | 0.0235392 | 0.149448 |
| 202 | hypertension | rs3755192 | T | C | 0.0564201 | -0.0601872 | 0.012047 | 5.85E-07 | -0.038003 | 0.049349 | 0.441249 |
| 203 | hypertension | rs376510895 | A | G | 0.022376 | -0.117159 | 0.0190263 | 7.38E-10 | 0.0644868 | 0.0772134 | 0.403618 |
| 204 | hypertension | rs3771612 | A | G | 0.865939 | -0.0520749 | 0.00810627 | 1.33E-10 | 0.0132765 | 0.0331297 | 0.688609 |
| 205 | hypertension | rs3774573 | C | T | 0.240065 | -0.0400705 | 0.00650063 | 7.09E-10 | -0.0310341 | 0.0264674 | 0.240981 |
| 206 | hypertension | rs3781412 | G | A | 0.383147 | 0.0299028 | 0.00569145 | 1.49E-07 | 0.0690675 | 0.0230784 | 0.00276497 |
| 207 | hypertension | rs3802228 | G | A | 0.568847 | -0.0349286 | 0.0055984 | 4.40E-10 | -0.0384507 | 0.0227584 | 0.0911192 |
| 208 | hypertension | rs3821843 | A | G | 0.659992 | 0.0397867 | 0.00591572 | 1.75E-11 | 0.0367261 | 0.0240747 | 0.127133 |
| 209 | hypertension | rs3863166 | A | G | 0.754751 | 0.0366603 | 0.00645707 | 1.37E-08 | 0.000654845 | 0.026269 | 0.980112 |
| 210 | hypertension | rs3918226 | T | C | 0.0699159 | 0.158459 | 0.0107723 | 5.57E-49 | 0.0834738 | 0.0447881 | 0.0623563 |
| 211 | hypertension | rs3975148 | C | A | 0.555446 | 0.0316689 | 0.00559685 | 1.53E-08 | -0.0258345 | 0.0228108 | 0.257401 |
| 212 | hypertension | rs4150828 | G | C | 0.113144 | -0.0440939 | 0.0088071 | 5.54E-07 | -0.0139054 | 0.0356587 | 0.696567 |
| 213 | hypertension | rs4233796 | T | C | 0.765672 | 0.0407396 | 0.00656439 | 5.43E-10 | 0.0264711 | 0.0267491 | 0.322365 |
| 214 | hypertension | rs4298 | T | C | 0.0920623 | -0.0745954 | 0.00967378 | 1.25E-14 | -0.0082485 | 0.0390886 | 0.832871 |
| 215 | hypertension | rs4320727 | A | G | 0.626015 | 0.0401986 | 0.00574467 | 2.60E-12 | -0.0456359 | 0.0233786 | 0.0509331 |
| 216 | hypertension | rs4416119 | A | C | 0.621949 | -0.0274839 | 0.0057855 | 2.03E-06 | -0.0399152 | 0.0235744 | 0.0904253 |
| 217 | hypertension | rs4555841 | A | G | 0.642961 | 0.0341986 | 0.00578742 | 3.44E-09 | 0.020565 | 0.0235928 | 0.383392 |
| 218 | hypertension | rs4599004 | T | C | 0.286131 | -0.0344707 | 0.00616151 | 2.21E-08 | 0.0441559 | 0.0250661 | 0.0781412 |
| 219 | hypertension | rs4617543 | C | T | 0.872693 | -0.0583469 | 0.00829068 | 1.96E-12 | -0.0346711 | 0.0339054 | 0.306505 |
| 220 | hypertension | rs4638151 | T | C | 0.628474 | -0.0366002 | 0.00573515 | 1.75E-10 | -0.0334841 | 0.0234229 | 0.152847 |
| 221 | hypertension | rs4661163 | A | T | 0.23061 | 0.0305329 | 0.00659176 | 3.62E-06 | 0.0657769 | 0.0269602 | 0.0146964 |
| 222 | hypertension | rs4690777 | C | T | 0.642846 | -0.0376983 | 0.00577762 | 6.81E-11 | 0.00375116 | 0.023533 | 0.873354 |
| 223 | hypertension | rs4691670 | T | C | 0.399389 | -0.0312864 | 0.00568084 | 3.64E-08 | 0.000726513 | 0.0231217 | 0.974934 |
| 224 | hypertension | rs4724960 | G | A | 0.440058 | 0.0344985 | 0.00558075 | 6.34E-10 | 0.0276006 | 0.0227189 | 0.224414 |
| 225 | hypertension | rs4743770 | A | G | 0.247416 | -0.0294771 | 0.00644403 | 4.78E-06 | -0.0377042 | 0.0261736 | 0.149714 |

| 226 | hypertension | rs4793089 | A | G | 0.327035 | 0.0286737 | 0.00592203 | 1.29E-06 | -0.0370089 | 0.0241374 | 0.125211 |
| --- | --- | --- | --- | --- | --- | --- | --- | --- | --- | --- | --- |
| 227 | hypertension | rs4822242 | A | G | 0.507659 | 0.0319253 | 0.00556329 | 9.55E-09 | -0.00350404 | 0.0226577 | 0.877096 |
| 228 | hypertension | rs4852898 | A | G | 0.214411 | 0.0334319 | 0.00674277 | 7.12E-07 | 0.0432777 | 0.027558 | 0.116316 |
| 229 | hypertension | rs489574 | A | G | 0.283018 | -0.0414815 | 0.00618197 | 1.95E-11 | -0.022328 | 0.0251711 | 0.375052 |
| 230 | hypertension | rs4921464 | G | A | 0.482985 | -0.0263603 | 0.0055544 | 2.08E-06 | -0.0576772 | 0.0226646 | 0.0109335 |
| 231 | hypertension | rs4930274 | T | A | 0.247211 | -0.0339005 | 0.00645358 | 1.50E-07 | -0.0143045 | 0.0262358 | 0.585599 |
| 232 | hypertension | rs507446 | T | C | 0.579577 | 0.0401159 | 0.00563837 | 1.12E-12 | 0.0240619 | 0.0229554 | 0.294545 |
| 233 | hypertension | rs512943 | T | C | 0.143572 | -0.0479795 | 0.00794952 | 1.58E-09 | 0.00112467 | 0.0322513 | 0.972182 |
| 234 | hypertension | rs536014705 | T | G | 0.038725 | 0.0662487 | 0.0144437 | 4.50E-06 | 0.00284587 | 0.0593629 | 0.961764 |
| 235 | hypertension | rs550135980 | T | C | 0.044823 | 0.0704792 | 0.0136129 | 2.25E-07 | 0.0101755 | 0.0557595 | 0.855199 |
| 236 | hypertension | rs55640925 | A | C | 0.175225 | -0.0547699 | 0.00737969 | 1.16E-13 | -0.0017925 | 0.029974 | 0.952314 |
| 237 | hypertension | rs55823154 | G | A | 0.197308 | -0.0366483 | 0.00702254 | 1.80E-07 | -0.0384515 | 0.0286537 | 0.179615 |
| 238 | hypertension | rs55850843 | T | C | 0.055187 | -0.0742503 | 0.0122508 | 1.35E-09 | -0.0255655 | 0.049631 | 0.606475 |
| 239 | hypertension | rs55962699 | T | A | 0.139779 | -0.0377999 | 0.00802069 | 2.44E-06 | -0.0326171 | 0.0326658 | 0.318034 |
| 240 | hypertension | rs57249154 | A | G | 0.175214 | 0.0339368 | 0.00727694 | 3.11E-06 | 0.0121509 | 0.0297031 | 0.682483 |
| 241 | hypertension | rs57274670 | C | G | 0.264725 | -0.0402382 | 0.00630551 | 1.75E-10 | -0.0123925 | 0.025635 | 0.628798 |
| 242 | hypertension | rs5752822 | G | A | 0.212799 | 0.0529293 | 0.00677213 | 5.46E-15 | -0.00412982 | 0.0276713 | 0.88136 |
| 243 | hypertension | rs57541197 | A | G | 0.102194 | -0.0683784 | 0.00918574 | 9.77E-14 | -0.0554903 | 0.0372537 | 0.136349 |
| 244 | hypertension | rs581136 | T | A | 0.417949 | -0.0301344 | 0.00562316 | 8.37E-08 | 0.0211447 | 0.022935 | 0.356559 |
| 245 | hypertension | rs5994376 | C | A | 0.539204 | 0.0294932 | 0.00556407 | 1.15E-07 | 0.00151129 | 0.022661 | 0.946828 |
| 246 | hypertension | rs6015445 | C | G | 0.162542 | 0.122374 | 0.00748051 | 3.75E-60 | 0.0218474 | 0.0306373 | 0.475784 |
| 247 | hypertension | rs6031400 | T | C | 0.126287 | 0.0393544 | 0.00833844 | 2.36E-06 | 0.0799088 | 0.0340382 | 0.0188934 |
| 248 | hypertension | rs60655814 | C | T | 0.279467 | 0.0312083 | 0.00620448 | 4.91E-07 | 0.026245 | 0.0253071 | 0.299707 |
| 249 | hypertension | rs607398 | C | A | 0.290941 | 0.0319305 | 0.0061281 | 1.88E-07 | -0.00400783 | 0.0249538 | 0.872401 |
| 250 | hypertension | rs60961847 | A | G | 0.189821 | -0.0448255 | 0.00712307 | 3.11E-10 | -0.0334476 | 0.029002 | 0.248793 |
| 251 | hypertension | rs61079605 | T | A | 0.300427 | 0.0277295 | 0.00605045 | 4.58E-06 | 0.0319888 | 0.0247265 | 0.195768 |
| 252 | hypertension | rs612652 | C | T | 0.573807 | 0.0624283 | 0.0056087 | 8.90E-29 | 0.0158164 | 0.0228204 | 0.488258 |
| 253 | hypertension | rs61890121 | A | T | 0.106418 | -0.0441802 | 0.00905646 | 1.07E-06 | 0.0404938 | 0.0368467 | 0.271776 |
| 254 | hypertension | rs61909170 | G | T | 0.270825 | 0.0298433 | 0.0062697 | 1.94E-06 | 0.0109218 | 0.0256026 | 0.669679 |
| 255 | hypertension | rs62052820 | A | G | 0.218506 | 0.041687 | 0.00674831 | 6.52E-10 | -0.0143741 | 0.0276375 | 0.602998 |
| 256 | hypertension | rs62097680 | G | A | 0.0533535 | -0.0686334 | 0.0124837 | 3.84E-08 | 0.0409673 | 0.050562 | 0.417802 |
| 257 | hypertension | rs62185661 | A | G | 0.241974 | 0.0548578 | 0.0064874 | 2.77E-17 | 0.0284766 | 0.0265369 | 0.283229 |
| 258 | hypertension | rs62271876 | C | A | 0.0214764 | -0.0896093 | 0.0193464 | 3.62E-06 | 0.196631 | 0.0774593 | 0.0111324 |
| 259 | hypertension | rs62287891 | A | G | 0.44281 | 0.036906 | 0.00559874 | 4.34E-11 | 0.0578541 | 0.0228448 | 0.0113256 |
| 260 | hypertension | rs62294340 | A | G | 0.278676 | -0.0365337 | 0.00621415 | 4.12E-09 | -0.0411172 | 0.0252699 | 0.103712 |
| 261 | hypertension | rs62363221 | T | C | 0.0918631 | 0.0471373 | 0.00958698 | 8.80E-07 | 0.0332185 | 0.0392148 | 0.396944 |
| 262 | hypertension | rs62426324 | T | C | 0.500236 | 0.0594781 | 0.00555077 | 8.63E-27 | -0.00877634 | 0.0226576 | 0.698499 |
| 263 | hypertension | rs62481856 | A | G | 0.297804 | 0.0521356 | 0.00606233 | 7.98E-18 | -0.00212064 | 0.0247681 | 0.931769 |

| 264 | hypertension | rs62494194 | A | G | 0.449944 | 0.0284751 | 0.00557236 | 3.22E-07 | 0.0200572 | 0.0227418 | 0.377802 |
| --- | --- | --- | --- | --- | --- | --- | --- | --- | --- | --- | --- |
| 265 | hypertension | rs6271 | T | C | 0.067042 | -0.0625106 | 0.0111824 | 2.27E-08 | 0.0367057 | 0.0451969 | 0.416718 |
| 266 | hypertension | rs630901 | A | T | 0.304272 | 0.0331743 | 0.00604342 | 4.03E-08 | 0.0352896 | 0.0246876 | 0.152877 |
| 267 | hypertension | rs6503837 | G | A | 0.518112 | -0.0321793 | 0.00557893 | 8.02E-09 | -0.0114906 | 0.0227307 | 0.613201 |
| 268 | hypertension | rs6538214 | C | A | 0.506875 | -0.0367923 | 0.00555757 | 3.59E-11 | -0.0461455 | 0.0226317 | 0.0414524 |
| 269 | hypertension | rs6591561 | G | A | 0.224357 | -0.0365174 | 0.00668625 | 4.72E-08 | -0.0153972 | 0.0272233 | 0.571673 |
| 270 | hypertension | rs66506004 | T | C | 0.0450498 | 0.0629164 | 0.0133142 | 2.30E-06 | -0.0326698 | 0.0544663 | 0.548628 |
| 271 | hypertension | rs665759 | A | G | 0.250677 | 0.0413022 | 0.00638206 | 9.70E-11 | -0.0251187 | 0.0259975 | 0.333945 |
| 272 | hypertension | rs66794444 | T | C | 0.108257 | 0.0563699 | 0.00891575 | 2.57E-10 | 0.0271573 | 0.0365634 | 0.457636 |
| 273 | hypertension | rs67227401 | T | C | 0.0996756 | -0.0681048 | 0.00931127 | 2.59E-13 | -0.0332386 | 0.0378675 | 0.380074 |
| 274 | hypertension | rs67341842 | C | T | 0.177339 | 0.0369073 | 0.00730542 | 4.37E-07 | -0.0161669 | 0.0298165 | 0.587672 |
| 275 | hypertension | rs6738563 | T | G | 0.504089 | -0.0285849 | 0.00559025 | 3.16E-07 | 0.0112144 | 0.0228006 | 0.622829 |
| 276 | hypertension | rs6743357 | A | G | 0.0994703 | -0.0510415 | 0.00931081 | 4.21E-08 | -0.0422752 | 0.0378726 | 0.264316 |
| 277 | hypertension | rs6756718 | T | C | 0.475433 | 0.027655 | 0.00555304 | 6.35E-07 | 0.0353056 | 0.0226219 | 0.118598 |
| 278 | hypertension | rs6766859 | T | C | 0.631788 | -0.0322447 | 0.00575294 | 2.08E-08 | -0.0350167 | 0.0234825 | 0.135914 |
| 279 | hypertension | rs67878714 | G | T | 0.804976 | 0.0374946 | 0.00700027 | 8.50E-08 | 0.0289891 | 0.0284279 | 0.307852 |
| 280 | hypertension | rs68041310 | T | G | 0.259906 | -0.0362881 | 0.00636644 | 1.20E-08 | -0.0554461 | 0.0259576 | 0.0326776 |
| 281 | hypertension | rs6860901 | T | C | 0.297284 | 0.059561 | 0.00605094 | 7.33E-23 | -0.0355151 | 0.0247236 | 0.150864 |
| 282 | hypertension | rs6885997 | T | C | 0.408568 | -0.030279 | 0.00564691 | 8.23E-08 | -0.0212992 | 0.0230184 | 0.354804 |
| 283 | hypertension | rs6901866 | C | T | 0.284292 | 0.0516837 | 0.00612009 | 3.04E-17 | 0.0407096 | 0.0250345 | 0.10392 |
| 284 | hypertension | rs6905288 | A | G | 0.566703 | 0.0325823 | 0.00561773 | 6.63E-09 | 0.0395504 | 0.022881 | 0.0838938 |
| 285 | hypertension | rs6918791 | G | C | 0.728541 | 0.0432675 | 0.00625094 | 4.46E-12 | 0.0100634 | 0.0254392 | 0.692409 |
| 286 | hypertension | rs6927317 | C | T | 0.399742 | 0.0350738 | 0.00567239 | 6.28E-10 | -0.0101869 | 0.0231293 | 0.659623 |
| 287 | hypertension | rs6961048 | G | C | 0.135898 | 0.0695971 | 0.00807773 | 6.94E-18 | 0.0104586 | 0.0330958 | 0.751995 |
| 288 | hypertension | rs6984496 | T | G | 0.457749 | -0.0443921 | 0.00561129 | 2.55E-15 | -0.0368176 | 0.0228796 | 0.107574 |
| 289 | hypertension | rs7070847 | A | G | 0.234826 | -0.0369762 | 0.00655155 | 1.66E-08 | -0.0179693 | 0.0267061 | 0.50104 |
| 290 | hypertension | rs7107356 | G | A | 0.400245 | 0.0354732 | 0.00566802 | 3.89E-10 | 0.0191533 | 0.0231098 | 0.407222 |
| 291 | hypertension | rs7139122 | A | G | 0.0200539 | 0.179197 | 0.0197305 | 1.06E-19 | -0.13266 | 0.0819533 | 0.105506 |
| 292 | hypertension | rs7174250 | T | C | 0.405516 | 0.0485348 | 0.00565258 | 8.98E-18 | 0.0137596 | 0.0230268 | 0.550144 |
| 293 | hypertension | rs7188268 | T | G | 0.0699373 | 0.0610143 | 0.0108519 | 1.88E-08 | -0.0503068 | 0.0440504 | 0.253442 |
| 294 | hypertension | rs7218899 | T | C | 0.601652 | -0.0270756 | 0.00565882 | 1.71E-06 | 0.00505051 | 0.0231189 | 0.827072 |
| 295 | hypertension | rs72640287 | T | C | 0.0771018 | -0.109011 | 0.010542 | 4.61E-25 | -0.0782934 | 0.042462 | 0.0652049 |
| 296 | hypertension | rs72662001 | C | T | 0.224007 | 0.0353305 | 0.0066607 | 1.13E-07 | 0.0323402 | 0.0272777 | 0.235783 |
| 297 | hypertension | rs72689147 | T | G | 0.217712 | -0.0623595 | 0.00672073 | 1.72E-20 | -0.0325165 | 0.0273659 | 0.234751 |
| 298 | hypertension | rs72702577 | C | T | 0.098378 | -0.056297 | 0.00937268 | 1.90E-09 | 0.0726277 | 0.0377976 | 0.0546701 |
| 299 | hypertension | rs72727487 | A | G | 0.178594 | -0.0393276 | 0.00726554 | 6.20E-08 | 0.00418721 | 0.0295111 | 0.88717 |
| 300 | hypertension | rs72759211 | G | A | 0.0399378 | 0.0709444 | 0.0141189 | 5.04E-07 | -0.0332138 | 0.0578917 | 0.566156 |
| 301 | hypertension | rs72779268 | A | C | 0.0642391 | -0.116634 | 0.0114188 | 1.71E-24 | -0.0128925 | 0.0459789 | 0.77917 |

| 302 | hypertension | rs72851658 | A | T | 0.196523 | 0.0523541 | 0.00700827 | 8.00E-14 | -0.0212744 | 0.0285692 | 0.456475 |
| --- | --- | --- | --- | --- | --- | --- | --- | --- | --- | --- | --- |
| 303 | hypertension | rs72981530 | T | C | 0.188083 | 0.0341936 | 0.00708574 | 1.40E-06 | 0.0559247 | 0.0288879 | 0.0528774 |
| 304 | hypertension | rs72995085 | C | T | 0.213677 | -0.0416616 | 0.00676134 | 7.19E-10 | 0.0198322 | 0.0274245 | 0.469583 |
| 305 | hypertension | rs73030267 | C | A | 0.0674858 | -0.0626514 | 0.011124 | 1.78E-08 | -0.0290574 | 0.0452938 | 0.521178 |
| 306 | hypertension | rs73033340 | G | A | 0.086982 | -0.0556014 | 0.00993266 | 2.17E-08 | -0.000804688 | 0.0404396 | 0.984124 |
| 307 | hypertension | rs73052033 | C | T | 0.16164 | -0.0356522 | 0.00753738 | 2.24E-06 | 0.00525392 | 0.0307483 | 0.864327 |
| 308 | hypertension | rs73073676 | T | A | 0.21958 | -0.0480506 | 0.00672853 | 9.24E-13 | 0.00842646 | 0.0272962 | 0.757546 |
| 309 | hypertension | rs7310615 | G | C | 0.58664 | -0.0658121 | 0.00563309 | 1.55E-31 | -0.0244849 | 0.0230451 | 0.28802 |
| 310 | hypertension | rs73143875 | G | A | 0.188855 | -0.0353196 | 0.00712999 | 7.28E-07 | 0.000886944 | 0.0289724 | 0.975578 |
| 311 | hypertension | rs73226528 | C | T | 0.282505 | 0.0323553 | 0.00616577 | 1.54E-07 | -0.00510679 | 0.0251833 | 0.839303 |
| 312 | hypertension | rs7336692 | C | T | 0.236659 | 0.0387518 | 0.00651591 | 2.73E-09 | 0.0428222 | 0.0266235 | 0.10774 |
| 313 | hypertension | rs74453624 | T | C | 0.140933 | 0.03862 | 0.00799035 | 1.34E-06 | 0.0375435 | 0.0327712 | 0.251951 |
| 314 | hypertension | rs74593044 | G | C | 0.0796423 | 0.0559978 | 0.0102648 | 4.89E-08 | 0.0305758 | 0.0415648 | 0.461964 |
| 315 | hypertension | rs751984 | C | T | 0.170905 | -0.0670123 | 0.00740938 | 1.51E-19 | -0.0255267 | 0.0300264 | 0.395246 |
| 316 | hypertension | rs754133 | A | G | 0.407722 | -0.0618261 | 0.00564484 | 6.45E-28 | -0.0323734 | 0.0230412 | 0.160015 |
| 317 | hypertension | rs75535096 | T | C | 0.0462312 | -0.0849014 | 0.0132941 | 1.70E-10 | -0.0353249 | 0.0534057 | 0.508328 |
| 318 | hypertension | rs75602510 | A | G | 0.0376957 | 0.0777445 | 0.0150201 | 2.27E-07 | -0.0977908 | 0.0617288 | 0.113148 |
| 319 | hypertension | rs75672964 | T | C | 0.0789102 | 0.0742783 | 0.0103435 | 6.91E-13 | 0.0406885 | 0.0424366 | 0.337656 |
| 320 | hypertension | rs756874 | G | A | 0.299679 | 0.0317877 | 0.00605425 | 1.52E-07 | 0.00141703 | 0.0246809 | 0.954215 |
| 321 | hypertension | rs75804753 | T | G | 0.133773 | -0.0377399 | 0.00818858 | 4.05E-06 | -0.0172167 | 0.0332929 | 0.605066 |
| 322 | hypertension | rs76181358 | T | A | 0.109308 | -0.0469335 | 0.00914115 | 2.83E-07 | -0.0440349 | 0.0371662 | 0.236092 |
| 323 | hypertension | rs76279233 | C | G | 0.0542105 | 0.0570984 | 0.012206 | 2.90E-06 | -0.00686599 | 0.0500605 | 0.890909 |
| 324 | hypertension | rs76357261 | G | T | 0.196519 | 0.0345708 | 0.00697446 | 7.17E-07 | 0.00979912 | 0.0285771 | 0.731673 |
| 325 | hypertension | rs76386877 | C | T | 0.468611 | 0.0319473 | 0.00556419 | 9.38E-09 | 0.0590889 | 0.0225931 | 0.00891354 |
| 326 | hypertension | rs76503758 | T | C | 0.0625259 | -0.0639132 | 0.0116267 | 3.86E-08 | 0.028862 | 0.047265 | 0.541436 |
| 327 | hypertension | rs7651161 | C | T | 0.470563 | -0.0258452 | 0.00560449 | 4.00E-06 | -0.00167903 | 0.0228231 | 0.941355 |
| 328 | hypertension | rs77098064 | A | G | 0.114804 | -0.0438442 | 0.00874624 | 5.36E-07 | 0.0214879 | 0.0355115 | 0.545116 |
| 329 | hypertension | rs7731095 | T | C | 0.129564 | 0.0490064 | 0.00825088 | 2.86E-09 | -0.0563756 | 0.0336765 | 0.0941239 |
| 330 | hypertension | rs7775474 | C | T | 0.431332 | -0.0263813 | 0.00558775 | 2.34E-06 | -0.0110504 | 0.0227683 | 0.627435 |
| 331 | hypertension | rs7788271 | G | A | 0.261803 | 0.030443 | 0.00629586 | 1.33E-06 | 0.0102177 | 0.0256599 | 0.690485 |
| 332 | hypertension | rs77924615 | A | G | 0.22305 | -0.037294 | 0.00671277 | 2.77E-08 | 0.0163421 | 0.0273662 | 0.550398 |
| 333 | hypertension | rs78058190 | A | G | 0.0828117 | 0.0586099 | 0.0100818 | 6.12E-09 | 0.0148727 | 0.0412165 | 0.718218 |
| 334 | hypertension | rs7813760 | T | G | 0.0283123 | -0.0929464 | 0.0168723 | 3.61E-08 | 0.121499 | 0.0679624 | 0.0738193 |
| 335 | hypertension | rs78204022 | T | C | 0.0529179 | 0.0628385 | 0.0123619 | 3.71E-07 | -0.0741054 | 0.0509091 | 0.145492 |
| 336 | hypertension | rs78307470 | G | A | 0.0639623 | -0.0772115 | 0.0114005 | 1.26E-11 | -0.0776798 | 0.046175 | 0.0925124 |
| 337 | hypertension | rs7864837 | A | G | 0.307081 | -0.030347 | 0.00602694 | 4.77E-07 | 0.0110023 | 0.0245702 | 0.654305 |
| 338 | hypertension | rs78660772 | T | A | 0.171536 | 0.0376857 | 0.00734592 | 2.90E-07 | -0.0425667 | 0.0299959 | 0.155875 |
| 339 | hypertension | rs7874497 | G | A | 0.627582 | 0.0433846 | 0.00577134 | 5.59E-14 | 0.0349072 | 0.0234988 | 0.137416 |

| 340 | hypertension | rs78745308 | G | C | 0.0476507 | 0.0766176 | 0.0130149 | 3.93E-09 | 0.000462369 | 0.0538544 | 0.99315 |
| --- | --- | --- | --- | --- | --- | --- | --- | --- | --- | --- | --- |
| 341 | hypertension | rs7874990 | T | C | 0.479192 | 0.0261109 | 0.00555849 | 2.63E-06 | 0.0241998 | 0.0226955 | 0.286296 |
| 342 | hypertension | rs78757334 | A | G | 0.071168 | 0.0511357 | 0.0108155 | 2.27E-06 | -0.0152797 | 0.044164 | 0.72936 |
| 343 | hypertension | rs7898224 | A | G | 0.384235 | 0.0387579 | 0.00571877 | 1.22E-11 | 0.0264822 | 0.0233716 | 0.257174 |
| 344 | hypertension | rs79386071 | C | T | 0.195558 | 0.0330674 | 0.00702426 | 2.51E-06 | 0.0744204 | 0.028227 | 0.00837664 |
| 345 | hypertension | rs7943119 | G | A | 0.259006 | 0.0418823 | 0.00633743 | 3.88E-11 | 0.0107639 | 0.0259508 | 0.678303 |
| 346 | hypertension | rs800980 | C | T | 0.877996 | -0.0443524 | 0.00852043 | 1.94E-07 | -0.00872089 | 0.034794 | 0.80209 |
| 347 | hypertension | rs8013475 | G | A | 0.166871 | 0.0374378 | 0.007435 | 4.77E-07 | 0.00299361 | 0.0304436 | 0.921668 |
| 348 | hypertension | rs804068 | T | C | 0.515695 | -0.0257384 | 0.00554708 | 3.48E-06 | -0.00244249 | 0.0226282 | 0.914043 |
| 349 | hypertension | rs8045875 | C | T | 0.594073 | 0.0400878 | 0.00565419 | 1.34E-12 | 0.0413115 | 0.0230466 | 0.07305 |
| 350 | hypertension | rs8079271 | T | G | 0.645531 | -0.0269956 | 0.00580443 | 3.31E-06 | 0.000664554 | 0.0236468 | 0.97758 |
| 351 | hypertension | rs8099311 | G | A | 0.40307 | -0.0265537 | 0.00565375 | 2.64E-06 | 0.0129355 | 0.0230071 | 0.573953 |
| 352 | hypertension | rs8126001 | T | C | 0.522454 | -0.0350677 | 0.00558385 | 3.38E-10 | -0.00270275 | 0.0227413 | 0.905396 |
| 353 | hypertension | rs880315 | C | T | 0.413507 | 0.0761292 | 0.0056349 | 1.36E-41 | 0.0424503 | 0.0230224 | 0.0652019 |
| 354 | hypertension | rs893065 | T | G | 0.194628 | -0.0469609 | 0.00707379 | 3.16E-11 | -0.00366921 | 0.0287995 | 0.89862 |
| 355 | hypertension | rs9265981 | C | A | 0.259164 | 0.0483824 | 0.00631767 | 1.88E-14 | 0.0325564 | 0.0258348 | 0.207605 |
| 356 | hypertension | rs9297164 | T | C | 0.619885 | -0.0316908 | 0.00574123 | 3.39E-08 | -0.0328009 | 0.023422 | 0.161384 |
| 357 | hypertension | rs9359360 | T | C | 0.409612 | 0.0451694 | 0.00563515 | 1.10E-15 | 0.0375516 | 0.023029 | 0.10297 |
| 358 | hypertension | rs9369275 | A | G | 0.394431 | -0.02902 | 0.00569734 | 3.51E-07 | -0.0231907 | 0.0232201 | 0.317924 |
| 359 | hypertension | rs941208 | T | C | 0.675736 | -0.0332762 | 0.00593489 | 2.06E-08 | -0.0119762 | 0.024242 | 0.621289 |
| 360 | hypertension | rs9508490 | T | C | 0.641733 | -0.0367507 | 0.00579069 | 2.20E-10 | -0.0271775 | 0.0236312 | 0.250115 |
| 361 | hypertension | rs9529924 | T | C | 0.599572 | -0.033095 | 0.00569823 | 6.32E-09 | 0.008907 | 0.0232262 | 0.701357 |
| 362 | hypertension | rs9532679 | C | A | 0.176075 | 0.0347605 | 0.00730055 | 1.92E-06 | -0.025382 | 0.0297619 | 0.39375 |
| 363 | hypertension | rs9649559 | G | A | 0.546175 | 0.0287256 | 0.00558556 | 2.71E-07 | -0.00324463 | 0.0227008 | 0.886346 |
| 364 | hypertension | rs9679855 | G | A | 0.0335001 | 0.111518 | 0.0153346 | 3.53E-13 | -0.0207471 | 0.0628245 | 0.74122 |
| 365 | hypertension | rs9692268 | T | C | 0.677476 | -0.0343696 | 0.00594187 | 7.28E-09 | -0.0109739 | 0.0242616 | 0.651042 |
| 366 | hypertension | rs9808753 | G | A | 0.185394 | 0.0392048 | 0.00712216 | 3.70E-08 | 0.0135806 | 0.0290351 | 0.639978 |
| 367 | hypertension | rs997295 | T | G | 0.644566 | 0.0400458 | 0.0057949 | 4.83E-12 | 0.016127 | 0.0235859 | 0.49413 |

Table S10 Instrumental variables used in MVMR analysis of red wine to hypertension.

|  | **Exposure** | **SNP** | **effect_allele** | **other_allele** | **eaf** | **beta** | **se** | **pval** |
| --- | --- | --- | --- | --- | --- | --- | --- | --- |
| 1 | average weekly red  wine intake | rs10063055 | T | C | 0.253339 | 0.000914786 | 0.00237101 | 0.699999936 |
| 2 | average weekly red  wine ingake | rs10063744 | G | C | 0.284531 | -0.00591272 | 0.00229651 | 0.01 |
| 3 | average weekly red  wine incake | rs10160769 | C | G | 0.217273 | -0.00128276 | 0.00252929 | 0.610000232 |
| 4 | average weekly red  wine intake | rs10172483 | T | A | 0.235526 | -0.00505974 | 0.00243021 | 0.036999853 |
| 5 | average weekly red  wine ingake | rs10182416 | G | A | 0.512647 | -0.00483718 | 0.00206224 | 0.018999842 |
| 6 | average weekly red  wine intake | rs10184537 | T | C | 0.338784 | 0.00356212 | 0.00218192 | 0.1 |
| 7 | average weekly red  wine intake | rs1019240 | T | A | 0.643138 | 0.000841897 | 0.00216119 | 0.699999936 |
| 8 | average weekly red  wine ingake | rs10280836 | G | T | 0.771368 | -0.0123121 | 0.00245691 | 5.39995325071583e-07 |
| 9 | average weekly red  wine incake | rs10402950 | C | T | 0.288967 | -0.0037405 | 0.00227756 | 0.1 |
| 10 | average weekly red  wine inaake | rs10423928 | A | T | 0.194206 | -0.00277932 | 0.00260217 | 0.290000001 |
| 11 | average weekly red  wine ingake | rs1048637 | G | T | 0.45207 | -0.00418332 | 0.00207079 | 0.043000153 |
| 12 | average weekly red  wine incake | rs10505836 | C | A | 0.859923 | -0.00302929 | 0.00299206 | 0.310000219 |
| 13 | average weekly red  wine intake | rs10510025 | T | C | 0.245928 | -0.00583298 | 0.00239985 | 0.014999957 |
| 14 | average weekly red  wine inaake | rs1064213 | A | G | 0.477109 | -0.00250134 | 0.00206318 | 0.230000087 |
| 15 | average weekly red  wine incake | rs10742752 | C | T | 0.612024 | 0.00425335 | 0.00211682 | 0.04499974 |
| 16 | average weekly red  wine intake | rs10760277 | T | C | 0.384679 | 0.000708897 | 0.00212458 | 0.740000478 |
| 17 | average weekly red  wine intake | rs10771041 | T | C | 0.115474 | -0.0079435 | 0.00322948 | 0.014000063 |
| 18 | average weekly red  wine inaake | rs10780248 | A | G | 0.559871 | 0.00294516 | 0.00207797 | 0.160000006 |

| 19 | average weekly red  wine intake | rs1078141 | T | C | 0.383015 | -0.0110116 | 0.00214723 | 2.90001336905407e-07 |
| --- | --- | --- | --- | --- | --- | --- | --- | --- |
| 20 | average weekly red  wine ingake | rs10799778 | G | T | 0.833855 | 0.00461663 | 0.00277208 | 0.095999727 |
| 21 | average weekly red  wine intake | rs10824211 | T | C | 0.139614 | -0.0019071 | 0.0029873 | 0.519999589 |
| 22 | average weekly red  wine intake | rs10827380 | T | C | 0.313711 | 0.00103804 | 0.00222622 | 0.640000038 |
| 23 | average weekly red  wine ingake | rs10832778 | G | C | 0.622133 | -0.00376829 | 0.00212583 | 0.075999371 |
| 24 | average weekly red  wine incake | rs10887578 | C | G | 0.497126 | -0.00175462 | 0.00207557 | 0.400000008 |
| 25 | average weekly red  wine inaake | rs10894670 | A | C | 0.551051 | 0.00354355 | 0.00207757 | 0.087999459 |
| 26 | average weekly red  wine inaake | rs10903791 | A | G | 0.604379 | -0.00112693 | 0.0021151 | 0.589999984 |
| 27 | average weekly red  wine ingake | rs10905688 | G | A | 0.902496 | 0.00747963 | 0.00348146 | 0.032000002 |
| 28 | average weekly red  wine incake | rs10915821 | C | T | 0.777253 | -6.07E-05 | 0.00247206 | 0.98000001 |
| 29 | average weekly red  wine inaake | rs10925183 | A | G | 0.607433 | -0.011922 | 0.00211168 | 1.60000006389793e-08 |
| 30 | average weekly red  wine inaake | rs10954277 | A | G | 0.291154 | 0.00141717 | 0.00227327 | 0.530000159 |
| 31 | average weekly red  wine intake | rs10960293 | T | G | 0.33365 | 0.00200455 | 0.00219352 | 0.359999585 |
| 32 | average weekly red  wine incake | rs10982030 | C | T | 0.055001 | -0.000887571 | 0.00453371 | 0.840000027 |
| 33 | average weekly red  wine inaake | rs10989067 | A | G | 0.314317 | -0.00211038 | 0.0022191 | 0.340000065 |
| 34 | average weekly red  wine intake | rs11001963 | T | C | 0.581522 | -0.00160144 | 0.00210513 | 0.450000504 |
| 35 | average weekly red  wine incake | rs11004108 | C | T | 0.262173 | 0.000456823 | 0.00234677 | 0.84999995 |
| 36 | average weekly red  wine intake | rs11009685 | T | C | 0.243716 | -0.00426282 | 0.00240651 | 0.075999371 |
| 37 | average weekly red  wine ingake | rs11012732 | G | A | 0.330529 | -0.00669083 | 0.00219318 | 0.002300011 |

| 38 | average weekly red  wine ingake | rs11021354 | G | A | 0.393325 | 0.0112243 | 0.0021204 | 1.19999655704812e-07 |
| --- | --- | --- | --- | --- | --- | --- | --- | --- |
| 39 | average weekly red  wine ingake | rs11057072 | G | A | 0.233185 | -0.00247467 | 0.00244082 | 0.310000219 |
| 40 | average weekly red  wine incake | rs11066651 | C | A | 0.293466 | -0.000451691 | 0.00226334 | 0.840000027 |
| 41 | average weekly red  wine intake | rs11079849 | T | C | 0.329998 | 0.00173099 | 0.00219771 | 0.429999549 |
| 42 | average weekly red  wine inaake | rs11097236 | A | G | 0.200042 | -0.00402679 | 0.00257628 | 0.119999932 |
| 43 | average weekly red  wine intake | rs11099020 | T | C | 0.641422 | -0.000166539 | 0.00215257 | 0.9400001 |
| 44 | average weekly red  wine inaake | rs11115160 | A | G | 0.237894 | 0.00425209 | 0.00243727 | 0.081000929 |
| 45 | average weekly red  wine ingake | rs11122450 | G | T | 0.612102 | 0.000972327 | 0.00211564 | 0.649999466 |
| 46 | average weekly red  wine ingake | rs11134679 | G | A | 0.68452 | -0.00194902 | 0.00222624 | 0.380000353 |
| 47 | average weekly red  wine incake | rs11136566 | C | A | 0.387536 | -0.000597722 | 0.00213289 | 0.780000714 |
| 48 | average weekly red  wine inaake | rs11150462 | A | T | 0.632779 | -0.00437234 | 0.00214497 | 0.042000069 |
| 49 | average weekly red  wine ingake | rs11150745 | G | A | 0.318276 | -0.00485188 | 0.00221731 | 0.029000134 |
| 50 | average weekly red  wine intake | rs111598585 | T | C | 0.208674 | 0.00295063 | 0.00254536 | 0.249999995 |
| 51 | average weekly red  wine incake | rs11161044 | C | G | 0.807238 | -0.00130924 | 0.00261767 | 0.620000443 |
| 52 | average weekly red  wine intake | rs11165643 | T | C | 0.590481 | 0.00410141 | 0.00209647 | 0.05 |
| 53 | average weekly red  wine incake | rs111689389 | C | G | 0.28288 | 8.61905e-05 | 0.00228931 | 0.969999923 |
| 54 | average weekly red  wine inaake | rs11218510 | A | G | 0.400743 | -0.00374291 | 0.00210867 | 0.075999371 |
| 55 | average weekly red  wine incake | rs11250094 | C | G | 0.548329 | -0.00316343 | 0.00207653 | 0.129999895 |
| 56 | average weekly red  wine incake | rs1126930 | C | G | 0.035193 | -0.00460911 | 0.0056 | 0.410000135 |

| 57 | average weekly red  wine intake | rs112765062 | T | C | 0.09301 | -0.00835253 | 0.00356213 | 0.018999842 |
| --- | --- | --- | --- | --- | --- | --- | --- | --- |
| 58 | average weekly red  wine intake | rs113079574 | T | C | 0.19225 | 0.00267587 | 0.00262999 | 0.310000219 |
| 59 | average weekly red  wine intake | rs113603865 | T | C | 0.211314 | -0.00331886 | 0.00254091 | 0.190000175 |
| 60 | average weekly red  wine inaake | rs113624107 | A | G | 0.225734 | -0.00023765 | 0.00246919 | 0.919999942 |
| 61 | average weekly red  wine incake | rs113936755 | C | T | 0.090609 | -0.00159067 | 0.00386189 | 0.680000137 |
| 62 | average weekly red  wine inaake | rs114167666 | A | G | 0.024846 | 0.000974832 | 0.00663421 | 0.880000056 |
| 63 | average weekly red  wine inaake | rs114313565 | A | G | 0.027269 | 0.0320306 | 0.00653599 | 9.59997274392481e-07 |
| 64 | average weekly red  wine inaake | rs114875897 | A | C | 0.019201 | -0.00120468 | 0.00750092 | 0.870000095 |
| 65 | average weekly red  wine incake | rs11525873 | C | T | 0.097902 | -0.000453935 | 0.00347448 | 0.89999998 |
| 66 | average weekly red  wine incake | rs11607476 | C | A | 0.485956 | 0.00180744 | 0.00207826 | 0.380000353 |
| 67 | average weekly red  wine ingake | rs11608710 | G | T | 0.061497 | -0.00777182 | 0.00435044 | 0.073999707 |
| 68 | average weekly red  wine inaake | rs11610621 | A | T | 0.14807 | 0.0033213 | 0.00290117 | 0.249999995 |
| 69 | average weekly red  wine ingake | rs11634851 | G | C | 0.46477 | -2.43E-05 | 0.00206558 | 0.98999999 |
| 70 | average weekly red  wine inaake | rs116374395 | A | G | 0.035042 | -0.00403686 | 0.00562359 | 0.470000154 |
| 71 | average weekly red  wine incake | rs11642090 | C | T | 0.373302 | -0.00200326 | 0.00214782 | 0.349999964 |
| 72 | average weekly red  wine inaake | rs11656076 | A | G | 0.224583 | -0.00186332 | 0.00246982 | 0.450000504 |
| 73 | average weekly red  wine inaake | rs1167311 | A | G | 0.681291 | 0.00322153 | 0.0022286 | 0.149999911 |
| 74 | average weekly red  wine inaake | rs116806934 | A | G | 0.023295 | 0.0052231 | 0.00689693 | 0.450000504 |
| 75 | average weekly red  wine inaake | rs11691869 | A | C | 0.362755 | 0.00775915 | 0.00215233 | 0.000309999 |

| 76 | average weekly red  wine incake | rs116952199 | C | A | 0.037777 | 0.00292371 | 0.00559737 | 0.599999654 |
| --- | --- | --- | --- | --- | --- | --- | --- | --- |
| 77 | average weekly red  wine inaake | rs11699828 | A | G | 0.036181 | 0.0212496 | 0.00603556 | 0.000430002 |
| 78 | average weekly red  wine ingake | rs11709402 | G | A | 0.277586 | -0.00679177 | 0.00231025 | 0.00329997 |
| 79 | average weekly red  wine incake | rs117118217 | C | G | 0.017729 | 0.00234831 | 0.00820495 | 0.770000487 |
| 80 | average weekly red  wine inaake | rs117435593 | A | G | 0.045424 | 0.00210524 | 0.00500755 | 0.670000304 |
| 81 | average weekly red  wine incake | rs11757278 | C | T | 0.304236 | 0.000736643 | 0.00224579 | 0.740000478 |
| 82 | average weekly red  wine ingake | rs11778219 | G | A | 0.163001 | -0.00528101 | 0.00280387 | 0.059999827 |
| 83 | average weekly red  wine incake | rs118019496 | C | G | 0.071676 | 0.00149016 | 0.0041096 | 0.719999177 |
| 84 | average weekly red  wine intake | rs118136827 | T | G | 0.281306 | 0.00109286 | 0.00229277 | 0.630000654 |
| 85 | average weekly red  wine inaake | rs1191600 | A | C | 0.593922 | 0.00975306 | 0.0021112 | 3.79996853036855e-06 |
| 86 | average weekly red  wine intake | rs11919665 | T | A | 0.680041 | 0.00460147 | 0.00220848 | 0.036999853 |
| 87 | average weekly red  wine ingake | rs12043569 | G | C | 0.220931 | 0.0129125 | 0.00248759 | 2.10000341026661e-07 |
| 88 | average weekly red  wine ingake | rs12072739 | G | A | 0.224206 | -0.00481929 | 0.00247695 | 0.0519996 |
| 89 | average weekly red  wine inaake | rs12089815 | A | G | 0.549035 | 0.00769688 | 0.00207848 | 0.00021 |
| 90 | average weekly red  wine ingake | rs12101393 | G | C | 0.218047 | 0.00262665 | 0.0025096 | 0.299999824 |
| 91 | average weekly red  wine intake | rs12140153 | T | G | 0.094941 | 0.010103 | 0.00360723 | 0.005099998 |
| 92 | average weekly red  wine inaake | rs12149660 | A | G | 0.115121 | 0.00132175 | 0.00324402 | 0.680000137 |
| 93 | average weekly red  wine incake | rs12151636 | C | T | 0.030483 | -0.00529332 | 0.00599735 | 0.380000353 |
| 94 | average weekly red  wine inaake | rs12259464 | A | G | 0.483935 | -0.00544395 | 0.00207144 | 0.008600031 |

| 95 | average weekly red  wine intake | rs12273545 | T | C | 0.056191 | -0.0113952 | 0.0044807 | 0.010999932 |
| --- | --- | --- | --- | --- | --- | --- | --- | --- |
| 96 | average weekly red  wine incake | rs1229984 | C | T | 0.976512 | 0.0609318 | 0.00673472 | 1.50003019045595e-19 |
| 97 | average weekly red  wine intake | rs12340969 | T | C | 0.440885 | 0.000751405 | 0.00208089 | 0.719999177 |
| 98 | average weekly red  wine ingake | rs12364470 | G | T | 0.164916 | -0.0014832 | 0.00277768 | 0.589999984 |
| 99 | average weekly red  wine ingake | rs12414412 | G | C | 0.085019 | -0.000353784 | 0.00370755 | 0.919999942 |
| 100 | average weekly red  wine intake | rs12440603 | T | C | 0.432885 | 0.00500284 | 0.00208286 | 0.016000001 |
| 101 | average weekly red  wine inaake | rs12462975 | A | G | 0.328972 | -0.0009738 | 0.00220916 | 0.660000098 |
| 102 | average weekly red  wine incake | rs12478299 | C | T | 0.252562 | 0.00431212 | 0.00237974 | 0.070000316 |
| 103 | average weekly red  wine intake | rs12507026 | T | A | 0.433782 | 0.00126424 | 0.00208302 | 0.540000299 |
| 104 | average weekly red  wine incake | rs12541408 | C | T | 0.317926 | 0.00590966 | 0.00221787 | 0.007699987 |
| 105 | average weekly red  wine intake | rs12633841 | T | G | 0.178046 | -0.00313584 | 0.00271552 | 0.249999995 |
| 106 | average weekly red  wine ingake | rs1263629 | G | A | 0.14377 | -0.00341637 | 0.00295452 | 0.249999995 |
| 107 | average weekly red  wine ingake | rs1266874 | G | A | 0.349864 | 0.00230028 | 0.00216241 | 0.290000001 |
| 108 | average weekly red  wine inaake | rs12681792 | A | C | 0.192918 | 0.00370279 | 0.00262328 | 0.160000006 |
| 109 | average weekly red  wine intake | rs12692596 | T | C | 0.370567 | -0.0116782 | 0.0021345 | 4.49997395325803e-08 |
| 110 | average weekly red  wine ingake | rs12696039 | G | A | 0.14964 | 0.00504723 | 0.00289109 | 0.081000929 |
| 111 | average weekly red  wine inaake | rs12712767 | A | G | 0.370893 | -0.00615554 | 0.00214057 | 0.004 |
| 112 | average weekly red  wine inaake | rs12776809 | A | G | 0.034184 | -0.00682613 | 0.00574931 | 0.239999866 |
| 113 | average weekly red  wine incake | rs1285245 | C | G | 0.374457 | -0.00271759 | 0.00213285 | 0.200000002 |

| 114 | average weekly red  wine inaake | rs1286058 | A | T | 0.702798 | -0.000979836 | 0.0022573 | 0.660000098 |
| --- | --- | --- | --- | --- | --- | --- | --- | --- |
| 115 | average weekly red  wine ingake | rs12881629 | G | A | 0.081979 | 0.00291768 | 0.00375134 | 0.440000328 |
| 116 | average weekly red  wine ingake | rs12921986 | G | A | 0.077838 | -0.0103841 | 0.00386157 | 0.007199959 |
| 117 | average weekly red  wine intake | rs12937411 | T | C | 0.408306 | 0.000535341 | 0.00209806 | 0.800000024 |
| 118 | average weekly red  wine incake | rs1296328 | C | A | 0.558893 | -0.00286358 | 0.0020884 | 0.170000031 |
| 119 | average weekly red  wine intake | rs12974458 | T | C | 0.54321 | -0.00214514 | 0.00207897 | 0.299999824 |
| 120 | average weekly red  wine intake | rs12987009 | T | A | 0.439949 | 0.00277162 | 0.00208429 | 0.180000205 |
| 121 | average weekly red  wine inaake | rs13012070 | A | G | 0.228591 | 0.000609342 | 0.00245733 | 0.800000024 |
| 122 | average weekly red  wine inaake | rs13033310 | A | G | 0.252135 | -0.00695987 | 0.00238985 | 0.003599979 |
| 123 | average weekly red  wine incake | rs13034936 | C | T | 0.111522 | -0.0172726 | 0.00327463 | 1.29998996537546e-07 |
| 124 | average weekly red  wine ingake | rs13041173 | G | A | 0.341966 | 0.00532142 | 0.00217748 | 0.014999957 |
| 125 | average weekly red  wine inaake | rs13097918 | A | T | 0.21215 | -0.00537118 | 0.00252488 | 0.032999701 |
| 126 | average weekly red  wine intake | rs13107325 | T | C | 0.073981 | -0.0123455 | 0.0039441 | 0.001700004 |
| 127 | average weekly red  wine inaake | rs13163306 | A | G | 0.465599 | 0.00279072 | 0.00206974 | 0.180000205 |
| 128 | average weekly red  wine incake | rs13176429 | C | T | 0.68717 | -0.00286968 | 0.00222497 | 0.200000002 |
| 129 | average weekly red  wine intake | rs1320251 | T | C | 0.454662 | 0.00259612 | 0.00207712 | 0.209999857 |
| 130 | average weekly red  wine ingake | rs13218383 | G | C | 0.334821 | 0.00217421 | 0.00218671 | 0.320000016 |
| 131 | average weekly red  wine ingake | rs1322842 | G | A | 0.608334 | -0.000297273 | 0.00212441 | 0.889999986 |
| 132 | average weekly red  wine incake | rs13248187 | C | T | 0.268417 | 0.00122478 | 0.00233788 | 0.599999654 |

| 133 | average weekly red  wine ingake | rs1327259 | G | A | 0.386693 | -0.000498514 | 0.00212495 | 0.809999965 |
| --- | --- | --- | --- | --- | --- | --- | --- | --- |
| 134 | average weekly red  wine incake | rs13290403 | C | T | 0.295752 | 0.00361017 | 0.00226367 | 0.11000008 |
| 135 | average weekly red  wine inaake | rs13291723 | A | G | 0.57205 | 0.00201221 | 0.00208529 | 0.330000046 |
| 136 | average weekly red  wine ingake | rs13427822 | G | A | 0.271242 | 0.00180021 | 0.00234476 | 0.440000328 |
| 137 | average weekly red  wine inaake | rs1346841 | A | G | 0.405268 | 0.00394996 | 0.00210666 | 0.061000023 |
| 138 | average weekly red  wine intake | rs1360201 | T | C | 0.48113 | -0.00513382 | 0.00206238 | 0.0129999 |
| 139 | average weekly red  wine ingake | rs1377825 | G | C | 0.436886 | 0.000382321 | 0.00208789 | 0.84999995 |
| 140 | average weekly red  wine inaake | rs1384131 | A | T | 0.420584 | 0.000123861 | 0.00210285 | 0.949999988 |
| 141 | average weekly red  wine intake | rs140159717 | T | C | 0.082483 | 0.00495388 | 0.0038594 | 0.200000002 |
| 142 | average weekly red  wine incake | rs1411432 | C | A | 0.185736 | -0.00692947 | 0.00266309 | 0.009299937 |
| 143 | average weekly red  wine incake | rs143121872 | C | T | 0.022386 | 0.00325252 | 0.0075089 | 0.660000098 |
| 144 | average weekly red  wine inaake | rs1438945 | A | T | 0.715174 | -0.00155386 | 0.00229374 | 0.499999995 |
| 145 | average weekly red  wine inaake | rs1441264 | A | G | 0.592719 | -0.000795205 | 0.00214242 | 0.70999943 |
| 146 | average weekly red  wine ingake | rs1446577 | G | C | 0.256445 | 0.0151328 | 0.00236106 | 1.49999565138204e-10 |
| 147 | average weekly red  wine intake | rs1451963 | T | G | 0.082187 | -0.00399079 | 0.00375688 | 0.290000001 |
| 148 | average weekly red  wine ingake | rs1454687 | G | C | 0.515563 | -0.00287493 | 0.00206186 | 0.160000006 |
| 149 | average weekly red  wine intake | rs1458156 | T | C | 0.489064 | 0.00718058 | 0.00206426 | 0.0005 |
| 150 | average weekly red  wine ingake | rs145981104 | G | A | 0.063511 | -0.00760489 | 0.00422321 | 0.071999586 |
| 151 | average weekly red  wine inaake | rs146569428 | A | G | 0.201347 | 0.00569593 | 0.00259045 | 0.028000127 |

| 152 | average weekly red  wine inaake | rs1471093 | A | G | 0.616446 | -0.00208432 | 0.00212961 | 0.330000046 |
| --- | --- | --- | --- | --- | --- | --- | --- | --- |
| 153 | average weekly red  wine incake | rs1471740 | C | T | 0.7408 | -0.00650142 | 0.00235716 | 0.005800027 |
| 154 | average weekly red  wine incake | rs147568678 | C | T | 0.238289 | -0.000469158 | 0.00242775 | 0.84999995 |
| 155 | average weekly red  wine incake | rs1477290 | C | T | 0.137325 | 0.0100495 | 0.00302333 | 0.00089 |
| 156 | average weekly red  wine intake | rs147730268 | T | G | 0.087184 | -0.00694102 | 0.00373984 | 0.06299992 |
| 157 | average weekly red  wine inaake | rs150215901 | A | T | 0.043004 | 0.00387837 | 0.00515878 | 0.450000504 |
| 158 | average weekly red  wine incake | rs1503526 | C | T | 0.479531 | -0.00339792 | 0.00206383 | 0.1 |
| 159 | average weekly red  wine intake | rs150684652 | T | C | 0.018655 | 0.0045372 | 0.00848021 | 0.589999984 |
| 160 | average weekly red  wine inaake | rs1523751 | A | G | 0.502961 | 0.00364709 | 0.00206724 | 0.077999173 |
| 161 | average weekly red  wine incake | rs156201 | C | G | 0.752919 | -0.00445872 | 0.00238832 | 0.061999759 |
| 162 | average weekly red  wine inaake | rs1582931 | A | G | 0.472977 | -0.00604936 | 0.00208274 | 0.003699985 |
| 163 | average weekly red  wine intake | rs1608113 | T | A | 0.365411 | 0.00469763 | 0.00214123 | 0.028000127 |
| 164 | average weekly red  wine ingake | rs1609010 | G | A | 0.565788 | -0.000728526 | 0.00208199 | 0.730000235 |
| 165 | average weekly red  wine intake | rs164328 | T | G | 0.912282 | 0.0184492 | 0.00367585 | 5.19995996533516e-07 |
| 166 | average weekly red  wine ingake | rs16965658 | G | A | 0.060358 | 0.00860471 | 0.00434893 | 0.047999863 |
| 167 | average weekly red  wine incake | rs17005677 | C | T | 0.305606 | -0.00412201 | 0.00224769 | 0.066999259 |
| 168 | average weekly red  wine incake | rs17056301 | C | T | 0.255556 | -0.00135141 | 0.00237307 | 0.570000189 |
| 169 | average weekly red  wine incake | rs17132130 | C | G | 0.222079 | 0.00430182 | 0.00248566 | 0.084000138 |
| 170 | average weekly red  wine incake | rs17141778 | C | G | 0.418211 | 0.00568168 | 0.00209018 | 0.00659994 |

| 171 | average weekly red  wine ingake | rs17289010 | G | A | 0.328838 | 0.00321973 | 0.00219529 | 0.139999988 |
| --- | --- | --- | --- | --- | --- | --- | --- | --- |
| 172 | average weekly red  wine ingake | rs17399739 | G | A | 0.069122 | 0.0074251 | 0.00407227 | 0.06800017 |
| 173 | average weekly red  wine ingake | rs17446299 | G | C | 0.165659 | 0.00553978 | 0.00277226 | 0.04600023 |
| 174 | average weekly red  wine incake | rs17544384 | C | T | 0.210839 | -0.00270642 | 0.00252733 | 0.27999998 |
| 175 | average weekly red  wine ingake | rs17668356 | G | C | 0.146472 | -0.00131896 | 0.00291638 | 0.649999466 |
| 176 | average weekly red  wine ingake | rs17724992 | G | A | 0.268688 | 0.000566052 | 0.00233156 | 0.809999965 |
| 177 | average weekly red  wine incake | rs17741830 | C | T | 0.08021 | -0.00179114 | 0.00382039 | 0.640000038 |
| 178 | average weekly red  wine intake | rs17770336 | T | C | 0.322164 | -0.00322066 | 0.00220435 | 0.139999988 |
| 179 | average weekly red  wine inaake | rs1778830 | A | G | 0.361979 | -0.00297352 | 0.00215103 | 0.170000031 |
| 180 | average weekly red  wine incake | rs1793636 | C | G | 0.309164 | 0.000489892 | 0.0022327 | 0.830000015 |
| 181 | average weekly red  wine intake | rs1818917 | T | C | 0.51299 | 0.00362822 | 0.00206785 | 0.079000529 |
| 182 | average weekly red  wine inaake | rs1834144 | A | C | 0.374654 | 0.00287872 | 0.00213597 | 0.180000205 |
| 183 | average weekly red  wine inaake | rs1860750 | A | T | 0.510331 | -0.00173894 | 0.00206449 | 0.400000008 |
| 184 | average weekly red  wine intake | rs1861410 | T | C | 0.555946 | 0.00300444 | 0.00208091 | 0.149999911 |
| 185 | average weekly red  wine ingake | rs1884897 | G | A | 0.627829 | 0.00278229 | 0.00214094 | 0.190000175 |
| 186 | average weekly red  wine intake | rs189252432 | T | G | 0.012497 | 0.0077729 | 0.00928661 | 0.400000008 |
| 187 | average weekly red  wine incake | rs1919243 | C | T | 0.486633 | -0.00434074 | 0.00208975 | 0.037999685 |
| 188 | average weekly red  wine inaake | rs1928706 | A | G | 0.471699 | 0.000284594 | 0.00207288 | 0.889999986 |
| 189 | average weekly red  wine inaake | rs1934102 | A | G | 0.347113 | 0.00282989 | 0.00219332 | 0.200000002 |

| 190 | average weekly red  wine inaake | rs1967772 | A | G | 0.285536 | -0.00314831 | 0.00229443 | 0.170000031 |
| --- | --- | --- | --- | --- | --- | --- | --- | --- |
| 191 | average weekly red  wine incake | rs1990662 | C | A | 0.193004 | 0.00411007 | 0.00262256 | 0.119999932 |
| 192 | average weekly red  wine intake | rs2035936 | T | G | 0.055306 | -0.00706923 | 0.00457367 | 0.119999932 |
| 193 | average weekly red  wine incake | rs2051559 | C | T | 0.13125 | -0.00404275 | 0.00306313 | 0.190000175 |
| 194 | average weekly red  wine ingake | rs2102278 | G | A | 0.321712 | -0.00525137 | 0.00220901 | 0.017000042 |
| 195 | average weekly red  wine intake | rs2133561 | T | A | 0.6122 | -0.000843252 | 0.00214062 | 0.689999856 |
| 196 | average weekly red  wine incake | rs213518 | C | T | 0.146328 | 0.00217951 | 0.00291917 | 0.460000178 |
| 197 | average weekly red  wine ingake | rs2153740 | G | A | 0.479023 | -0.00429165 | 0.00207542 | 0.038999586 |
| 198 | average weekly red  wine ingake | rs215634 | G | A | 0.613067 | 0.00374804 | 0.002125 | 0.077999173 |
| 199 | average weekly red  wine incake | rs2172131 | C | T | 0.580669 | 0.00446667 | 0.00209103 | 0.032999701 |
| 200 | average weekly red  wine incake | rs217672 | C | A | 0.271135 | -0.00827456 | 0.00232703 | 0.000379997 |
| 201 | average weekly red  wine ingake | rs2181350 | G | A | 0.713861 | 0.000137878 | 0.00230161 | 0.949999988 |
| 202 | average weekly red  wine intake | rs2190887 | T | C | 0.560796 | 0.000430983 | 0.00207786 | 0.840000027 |
| 203 | average weekly red  wine ingake | rs2192158 | G | A | 0.555409 | 0.00323049 | 0.00207389 | 0.119999932 |
| 204 | average weekly red  wine ingake | rs2193101 | G | C | 0.812933 | 0.00105799 | 0.00267567 | 0.689999856 |
| 205 | average weekly red  wine inaake | rs2216931 | A | C | 0.661061 | -0.00308178 | 0.00218185 | 0.160000006 |
| 206 | average weekly red  wine intake | rs2234458 | T | C | 0.64004 | 0.00578513 | 0.00214569 | 0.007000032 |
| 207 | average weekly red  wine incake | rs2249825 | C | G | 0.266805 | 0.00148538 | 0.00233177 | 0.519999589 |
| 208 | average weekly red  wine ingake | rs2253310 | G | C | 0.6267 | 0.00502578 | 0.00213168 | 0.017999896 |

| 209 | average weekly red  wine incake | rs2270494 | C | G | 0.549516 | 0.0110561 | 0.00208552 | 1.09999319893519e-07 |
| --- | --- | --- | --- | --- | --- | --- | --- | --- |
| 210 | average weekly red  wine inaake | rs2271189 | A | G | 0.404546 | 0.0031213 | 0.00210509 | 0.139999988 |
| 211 | average weekly red  wine ingake | rs2275003 | G | A | 0.521085 | -0.000849431 | 0.00206188 | 0.680000137 |
| 212 | average weekly red  wine inaake | rs2275444 | A | G | 0.725775 | 0.00354227 | 0.00230926 | 0.129999895 |
| 213 | average weekly red  wine incake | rs227638 | C | T | 0.855591 | -0.000550304 | 0.00294138 | 0.84999995 |
| 214 | average weekly red  wine intake | rs2283093 | T | C | 0.202882 | -0.00104703 | 0.00256094 | 0.680000137 |
| 215 | average weekly red  wine intake | rs2289379 | T | C | 0.397107 | 0.00512727 | 0.00211581 | 0.014999957 |
| 216 | average weekly red  wine incake | rs2307111 | C | T | 0.395274 | -0.000490735 | 0.00211007 | 0.82000009 |
| 217 | average weekly red  wine ingake | rs2342892 | G | T | 0.516098 | -0.00235527 | 0.0020621 | 0.249999995 |
| 218 | average weekly red  wine inaake | rs2376481 | A | G | 0.469028 | -0.00523774 | 0.0020659 | 0.010999932 |
| 219 | average weekly red  wine incake | rs2381404 | C | T | 0.243805 | -0.00254226 | 0.00240582 | 0.290000001 |
| 220 | average weekly red  wine inaake | rs2396625 | A | T | 0.422088 | 0.00171809 | 0.00209866 | 0.410000135 |
| 221 | average weekly red  wine ingake | rs2398861 | G | A | 0.257692 | -0.0012647 | 0.00236892 | 0.589999984 |
| 222 | average weekly red  wine incake | rs2416927 | C | G | 0.437989 | 0.00108556 | 0.00207699 | 0.599999654 |
| 223 | average weekly red  wine intake | rs242556 | T | A | 0.765394 | -0.00247862 | 0.00249137 | 0.320000016 |
| 224 | average weekly red  wine inaake | rs2425816 | A | G | 0.414703 | 0.0001487 | 0.00209413 | 0.9400001 |
| 225 | average weekly red  wine inaake | rs2433733 | A | G | 0.678047 | 0.00300343 | 0.00220814 | 0.170000031 |
| 226 | average weekly red  wine ingake | rs2439823 | G | A | 0.544552 | -0.00680674 | 0.00207509 | 0.001 |
| 227 | average weekly red  wine incake | rs2482356 | C | T | 0.428824 | -0.000516273 | 0.00208078 | 0.800000024 |

| 228 | average weekly red  wine incake | rs2512892 | C | T | 0.566078 | -0.00800775 | 0.00208414 | 0.00012 |
| --- | --- | --- | --- | --- | --- | --- | --- | --- |
| 229 | average weekly red  wine intake | rs252761 | T | G | 0.588199 | -0.000235331 | 0.00210641 | 0.910000016 |
| 230 | average weekly red  wine intake | rs2555502 | T | C | 0.376201 | -0.000161585 | 0.00212738 | 0.9400001 |
| 231 | average weekly red  wine inaake | rs2568958 | A | G | 0.602142 | 0.00723841 | 0.00210376 | 0.000580003 |
| 232 | average weekly red  wine inaake | rs2591131 | A | G | 0.186294 | 0.00176245 | 0.00264826 | 0.509999793 |
| 233 | average weekly red  wine incake | rs2606228 | C | A | 0.64604 | 0.00113131 | 0.00217665 | 0.599999654 |
| 234 | average weekly red  wine inaake | rs2616143 | A | G | 0.320796 | -0.000998784 | 0.00221501 | 0.649999466 |
| 235 | average weekly red  wine intake | rs2618039 | T | A | 0.380923 | -0.00267218 | 0.00212577 | 0.209999857 |
| 236 | average weekly red  wine incake | rs2646355 | C | T | 0.449099 | 0.000386692 | 0.00207891 | 0.84999995 |
| 237 | average weekly red  wine incake | rs2660971 | C | G | 0.653812 | 0.00349566 | 0.00216895 | 0.11000008 |
| 238 | average weekly red  wine ingake | rs2678204 | G | T | 0.339707 | -0.00694541 | 0.00217785 | 0.001400006 |
| 239 | average weekly red  wine ingake | rs2699189 | G | A | 0.457763 | -0.000112366 | 0.00208915 | 0.959999927 |
| 240 | average weekly red  wine ingake | rs2725371 | G | A | 0.697449 | 0.00602815 | 0.00225327 | 0.007499978 |
| 241 | average weekly red  wine intake | rs2781668 | T | C | 0.165389 | -0.00273569 | 0.00277856 | 0.320000016 |
| 242 | average weekly red  wine intake | rs2791643 | T | C | 0.761593 | 0.00067096 | 0.00241932 | 0.780000714 |
| 243 | average weekly red  wine intake | rs2833971 | T | C | 0.101767 | 0.00887439 | 0.00342034 | 0.009499921 |
| 244 | average weekly red  wine ingake | rs28350 | G | A | 0.82123 | -0.00172876 | 0.00270088 | 0.519999589 |
| 245 | average weekly red  wine incake | rs28366156 | C | T | 0.130767 | -0.00177055 | 0.00305644 | 0.559999965 |
| 246 | average weekly red  wine incake | rs2837996 | C | T | 0.650071 | -0.0050113 | 0.00216115 | 0.02 |

| 247 | average weekly red  wine intake | rs28404639 | T | C | 0.366021 | -0.00685351 | 0.00214559 | 0.001400006 |
| --- | --- | --- | --- | --- | --- | --- | --- | --- |
| 248 | average weekly red  wine ingake | rs284532 | G | T | 0.523344 | 0.00101145 | 0.00206683 | 0.620000443 |
| 249 | average weekly red  wine ingake | rs28454448 | G | A | 0.719062 | -0.00250755 | 0.00229488 | 0.270000147 |
| 250 | average weekly red  wine inaake | rs28489620 | A | G | 0.291629 | 0.001486 | 0.00228704 | 0.519999589 |
| 251 | average weekly red  wine incake | rs2861685 | C | T | 0.412195 | -0.00275306 | 0.00209104 | 0.190000175 |
| 252 | average weekly red  wine incake | rs28670671 | C | T | 0.285474 | 0.000571752 | 0.00236289 | 0.809999965 |
| 253 | average weekly red  wine ingake | rs28678108 | G | A | 0.448326 | -0.000346475 | 0.00209733 | 0.870000095 |
| 254 | average weekly red  wine intake | rs2870111 | T | C | 0.412273 | 0.00122529 | 0.00210134 | 0.559999965 |
| 255 | average weekly red  wine incake | rs2875762 | C | G | 0.241968 | 0.00255664 | 0.00241827 | 0.290000001 |
| 256 | average weekly red  wine intake | rs2899644 | T | C | 0.230046 | -0.00155988 | 0.00245757 | 0.530000159 |
| 257 | average weekly red  wine intake | rs2919389 | T | C | 0.401154 | 0.00326487 | 0.00211531 | 0.119999932 |
| 258 | average weekly red  wine intake | rs2920503 | T | C | 0.286479 | 0.00241088 | 0.00229392 | 0.290000001 |
| 259 | average weekly red  wine ingake | rs2941452 | G | A | 0.618266 | -0.00376563 | 0.00211974 | 0.075999371 |
| 260 | average weekly red  wine intake | rs2962334 | T | G | 0.020045 | 0.00799897 | 0.00733576 | 0.27999998 |
| 261 | average weekly red  wine ingake | rs2973564 | G | A | 0.300812 | 0.000674352 | 0.00224939 | 0.760000713 |
| 262 | average weekly red  wine inaake | rs303753 | A | G | 0.344672 | -0.0183206 | 0.0021899 | 6.00067353864114e-17 |
| 263 | average weekly red  wine ingake | rs303948 | G | A | 0.091742 | 0.00305962 | 0.00358535 | 0.390000353 |
| 264 | average weekly red  wine intake | rs308632 | T | C | 0.253255 | -0.0010263 | 0.00238776 | 0.670000304 |
| 265 | average weekly red  wine inaake | rs317656 | A | T | 0.72501 | -0.00110735 | 0.00231077 | 0.630000654 |

| 266 | average weekly red  wine ingake | rs3212038 | G | A | 0.328484 | 0.000322816 | 0.00220161 | 0.880000056 |
| --- | --- | --- | --- | --- | --- | --- | --- | --- |
| 267 | average weekly red  wine inaake | rs3213943 | A | C | 0.130022 | 0.000966843 | 0.00303365 | 0.749999545 |
| 268 | average weekly red  wine intake | rs32421 | T | A | 0.224119 | 0.00114165 | 0.00248349 | 0.649999466 |
| 269 | average weekly red  wine intake | rs329118 | T | C | 0.420173 | 0.00418516 | 0.0020927 | 0.04600023 |
| 270 | average weekly red  wine intake | rs329651 | T | G | 0.803333 | -0.00397383 | 0.00260575 | 0.129999895 |
| 271 | average weekly red  wine intake | rs34045288 | T | C | 0.334753 | -0.000805413 | 0.00218499 | 0.70999943 |
| 272 | average weekly red  wine incake | rs34153025 | C | T | 0.022264 | -0.0028899 | 0.00704828 | 0.680000137 |
| 273 | average weekly red  wine inaake | rs34173062 | A | G | 0.072542 | 0.00444948 | 0.00420636 | 0.290000001 |
| 274 | average weekly red  wine inaake | rs34481751 | A | C | 0.165557 | -0.000717159 | 0.00281552 | 0.800000024 |
| 275 | average weekly red  wine inaake | rs34517439 | A | C | 0.121219 | -0.00762439 | 0.00319695 | 0.017000042 |
| 276 | average weekly red  wine incake | rs34696181 | C | T | 0.475818 | 0.00302324 | 0.00206929 | 0.139999988 |
| 277 | average weekly red  wine ingake | rs347551 | G | C | 0.471524 | -0.000219202 | 0.00209939 | 0.919999942 |
| 278 | average weekly red  wine incake | rs34774377 | C | T | 0.119072 | 0.000789905 | 0.00318528 | 0.800000024 |
| 279 | average weekly red  wine inaake | rs34811474 | A | G | 0.232314 | 0.00762082 | 0.00244149 | 0.00179999 |
| 280 | average weekly red  wine inaake | rs349071 | A | G | 0.500328 | -0.00170572 | 0.00206836 | 0.410000135 |
| 281 | average weekly red  wine ingake | rs35154326 | G | A | 0.274362 | 0.00856285 | 0.00232953 | 0.000239999 |
| 282 | average weekly red  wine intake | rs35364449 | T | C | 0.109977 | 0.00406811 | 0.00331047 | 0.220000162 |
| 283 | average weekly red  wine inaake | rs35408866 | A | G | 0.136063 | 0.000868563 | 0.00301771 | 0.770000487 |
| 284 | average weekly red  wine incake | rs355777 | C | G | 0.407019 | 0.00385754 | 0.00210415 | 0.066999259 |

| 285 | average weekly red  wine inaake | rs35697587 | A | G | 0.508655 | -0.00439755 | 0.00206461 | 0.032999701 |
| --- | --- | --- | --- | --- | --- | --- | --- | --- |
| 286 | average weekly red  wine ingake | rs35697691 | G | C | 0.089275 | 0.00215133 | 0.00366914 | 0.559999965 |
| 287 | average weekly red  wine ingake | rs35746264 | G | A | 0.454806 | 0.00531183 | 0.0020711 | 0.01 |
| 288 | average weekly red  wine inaake | rs35809007 | A | G | 0.363994 | 0.000885144 | 0.0021486 | 0.680000137 |
| 289 | average weekly red  wine intake | rs35957544 | T | G | 0.575046 | -0.00256415 | 0.00208959 | 0.220000162 |
| 290 | average weekly red  wine inaake | rs36007635 | A | G | 0.138319 | 0.00839471 | 0.00298803 | 0.005 |
| 291 | average weekly red  wine incake | rs36029912 | C | T | 0.051504 | -0.00347153 | 0.00468836 | 0.460000178 |
| 292 | average weekly red  wine intake | rs36061954 | T | C | 0.397819 | -0.004999 | 0.00210812 | 0.017999896 |
| 293 | average weekly red  wine inaake | rs3730071 | A | C | 0.03012 | -0.00508334 | 0.0060289 | 0.400000008 |
| 294 | average weekly red  wine ingake | rs3764625 | G | T | 0.587426 | 0.000691619 | 0.00209758 | 0.740000478 |
| 295 | average weekly red  wine inaake | rs3766823 | A | G | 0.171596 | -0.00950739 | 0.00273656 | 0.00051 |
| 296 | average weekly red  wine incake | rs3784710 | C | T | 0.226259 | -0.000605611 | 0.00246125 | 0.809999965 |
| 297 | average weekly red  wine ingake | rs3803286 | G | A | 0.666363 | 0.0019875 | 0.00218675 | 0.359999585 |
| 298 | average weekly red  wine intake | rs3807566 | T | G | 0.43772 | 9.93681e-05 | 0.00208112 | 0.959999927 |
| 299 | average weekly red  wine intake | rs3814883 | T | C | 0.481277 | -0.00207388 | 0.00207042 | 0.320000016 |
| 300 | average weekly red  wine intake | rs3847199 | T | C | 0.629642 | 0.00279893 | 0.00217247 | 0.200000002 |
| 301 | average weekly red  wine incake | rs3848453 | C | T | 0.584205 | -0.00119521 | 0.00209648 | 0.570000189 |
| 302 | average weekly red  wine ingake | rs3851998 | G | C | 0.743661 | 0.000278272 | 0.00237275 | 0.910000016 |
| 303 | average weekly red  wine inaake | rs3866805 | A | C | 0.355171 | -0.00395597 | 0.00216103 | 0.066999259 |

| 304 | average weekly red  wine intake | rs3897102 | T | C | 0.410418 | -0.0065767 | 0.00211626 | 0.001899984 |
| --- | --- | --- | --- | --- | --- | --- | --- | --- |
| 305 | average weekly red  wine inaake | rs3901286 | A | C | 0.152015 | -0.00746828 | 0.00287764 | 0.009499921 |
| 306 | average weekly red  wine inaake | rs3935190 | A | G | 0.53644 | -0.000763335 | 0.00207944 | 0.70999943 |
| 307 | average weekly red  wine incake | rs394608 | C | T | 0.537331 | -0.00309079 | 0.00207378 | 0.139999988 |
| 308 | average weekly red  wine ingake | rs396755 | G | C | 0.572004 | 0.000515247 | 0.00208794 | 0.809999965 |
| 309 | average weekly red  wine incake | rs40071 | C | T | 0.17979 | -0.00470301 | 0.00269156 | 0.081000929 |
| 310 | average weekly red  wine intake | rs4017425 | T | C | 0.469775 | 0.00819153 | 0.00206845 | 7.49997818203003e-05 |
| 311 | average weekly red  wine intake | rs4055791 | T | C | 0.417762 | -0.000304176 | 0.00208989 | 0.880000056 |
| 312 | average weekly red  wine intake | rs40631 | T | G | 0.609513 | -0.000732064 | 0.00223669 | 0.740000478 |
| 313 | average weekly red  wine ingake | rs406388 | G | C | 0.177336 | 0.00051497 | 0.00270477 | 0.84999995 |
| 314 | average weekly red  wine ingake | rs41279738 | G | T | 0.025871 | -0.00549874 | 0.00652284 | 0.400000008 |
| 315 | average weekly red  wine incake | rs41315816 | C | T | 0.058225 | -0.00709261 | 0.00440386 | 0.11000008 |
| 316 | average weekly red  wine ingake | rs4148155 | G | A | 0.113329 | -0.00371447 | 0.00324471 | 0.249999995 |
| 317 | average weekly red  wine inaake | rs4235838 | A | G | 0.326027 | 0.000147188 | 0.00221233 | 0.949999988 |
| 318 | average weekly red  wine ingake | rs4261944 | G | T | 0.363959 | 0.00169021 | 0.00215001 | 0.429999549 |
| 319 | average weekly red  wine incake | rs4284600 | C | T | 0.466936 | -0.00116267 | 0.00207589 | 0.580000009 |
| 320 | average weekly red  wine ingake | rs429343 | G | A | 0.577538 | 0.00480166 | 0.00208969 | 0.021999864 |
| 321 | average weekly red  wine incake | rs429358 | C | T | 0.154643 | 0.0004977 | 0.00285472 | 0.860000097 |
| 322 | average weekly red  wine ingake | rs4307239 | G | A | 0.458295 | -0.00011449 | 0.00207279 | 0.959999927 |

| 323 | average weekly red  wine incake | rs4397962 | C | T | 0.220258 | 0.000377315 | 0.00249796 | 0.880000056 |
| --- | --- | --- | --- | --- | --- | --- | --- | --- |
| 324 | average weekly red  wine intake | rs4419475 | T | A | 0.406962 | -0.00360984 | 0.00209747 | 0.08500021 |
| 325 | average weekly red  wine incake | rs4430672 | C | T | 0.801744 | 0.00142883 | 0.00259091 | 0.580000009 |
| 326 | average weekly red  wine intake | rs4456769 | T | C | 0.333869 | -0.00134511 | 0.0021857 | 0.540000299 |
| 327 | average weekly red  wine intake | rs4477562 | T | C | 0.128437 | 0.00364708 | 0.00310677 | 0.239999866 |
| 328 | average weekly red  wine inaake | rs4482463 | A | C | 0.923106 | -0.000487292 | 0.00388213 | 0.89999998 |
| 329 | average weekly red  wine inaake | rs4524456 | A | G | 0.525814 | -0.0003107 | 0.00206572 | 0.880000056 |
| 330 | average weekly red  wine inaake | rs45486197 | A | G | 0.065861 | 0.00555794 | 0.00419483 | 0.190000175 |
| 331 | average weekly red  wine incake | rs4605363 | C | A | 0.340003 | -0.0045107 | 0.00217748 | 0.037999685 |
| 332 | average weekly red  wine intake | rs4625888 | T | C | 0.556266 | -0.00195907 | 0.00207451 | 0.340000065 |
| 333 | average weekly red  wine inaake | rs4643716 | A | C | 0.583739 | 0.0115308 | 0.00210517 | 4.3000152915127e-08 |
| 334 | average weekly red  wine inaake | rs4648450 | A | C | 0.467135 | 0.00207579 | 0.00208019 | 0.320000016 |
| 335 | average weekly red  wine intake | rs4658403 | T | C | 0.834186 | 0.00818272 | 0.00277579 | 0.0032 |
| 336 | average weekly red  wine intake | rs4672338 | T | C | 0.335716 | -0.00221108 | 0.00218448 | 0.310000219 |
| 337 | average weekly red  wine intake | rs4700780 | T | C | 0.298348 | -0.00407064 | 0.00225685 | 0.07100027 |
| 338 | average weekly red  wine intake | rs4722398 | T | C | 0.136055 | 0.00610417 | 0.00299956 | 0.042000069 |
| 339 | average weekly red  wine ingake | rs4734122 | G | A | 0.577345 | 0.00571421 | 0.00208978 | 0.006299992 |
| 340 | average weekly red  wine intake | rs4737188 | T | A | 0.474832 | 0.00210292 | 0.00206932 | 0.310000219 |
| 341 | average weekly red  wine ingake | rs4764949 | G | A | 0.324681 | -0.0031966 | 0.00220364 | 0.149999911 |

| 342 | average weekly red  wine inaake | rs4790292 | A | C | 0.154213 | 0.00204026 | 0.00286683 | 0.479999738 |
| --- | --- | --- | --- | --- | --- | --- | --- | --- |
| 343 | average weekly red  wine inaake | rs4800756 | A | G | 0.647135 | -0.000761877 | 0.00215762 | 0.719999177 |
| 344 | average weekly red  wine ingake | rs4820410 | G | A | 0.3453 | -0.00571398 | 0.00217063 | 0.008500021 |
| 345 | average weekly red  wine intake | rs4832298 | T | C | 0.687215 | 0.0015504 | 0.00222396 | 0.48999991 |
| 346 | average weekly red  wine incake | rs4858940 | C | T | 0.885006 | -0.00619676 | 0.00323431 | 0.054999659 |
| 347 | average weekly red  wine ingake | rs4876611 | G | A | 0.719472 | -0.00102084 | 0.0022979 | 0.660000098 |
| 348 | average weekly red  wine ingake | rs4884559 | G | T | 0.45339 | -0.000612441 | 0.00207154 | 0.770000487 |
| 349 | average weekly red  wine intake | rs4908672 | T | C | 0.392357 | 0.00329329 | 0.002115 | 0.119999932 |
| 350 | average weekly red  wine incake | rs4929923 | C | T | 0.644618 | -0.00045123 | 0.00215066 | 0.830000015 |
| 351 | average weekly red  wine inaake | rs4944769 | A | G | 0.281973 | 0.0023601 | 0.00231191 | 0.310000219 |
| 352 | average weekly red  wine incake | rs4947461 | C | T | 0.53896 | 0.00402095 | 0.00206754 | 0.0519996 |
| 353 | average weekly red  wine ingake | rs4976553 | G | A | 0.194168 | 0.00174829 | 0.0026347 | 0.509999793 |
| 354 | average weekly red  wine ingake | rs5011579 | G | C | 0.714103 | -0.00366031 | 0.00228473 | 0.11000008 |
| 355 | average weekly red  wine incake | rs512121 | C | T | 0.192046 | 0.00375313 | 0.00262754 | 0.149999911 |
| 356 | average weekly red  wine ingake | rs529200 | G | A | 0.526832 | -0.00524097 | 0.00206712 | 0.010999932 |
| 357 | average weekly red  wine incake | rs539515 | C | A | 0.204314 | -0.00370319 | 0.0025572 | 0.149999911 |
| 358 | average weekly red  wine ingake | rs55707359 | G | T | 0.01531 | -0.00936644 | 0.00850508 | 0.270000147 |
| 359 | average weekly red  wine incake | rs55714539 | C | A | 0.34387 | 0.00114157 | 0.00218556 | 0.599999654 |
| 360 | average weekly red  wine inaake | rs55726687 | A | G | 0.209817 | -0.000531419 | 0.0025294 | 0.830000015 |

| 361 | average weekly red  wine intake | rs55736343 | T | A | 0.197919 | 0.00184103 | 0.00259371 | 0.479999738 |
| --- | --- | --- | --- | --- | --- | --- | --- | --- |
| 362 | average weekly red  wine inaake | rs55769038 | A | G | 0.590605 | 0.00381195 | 0.00209599 | 0.069000144 |
| 363 | average weekly red  wine ingake | rs557951 | G | T | 0.312534 | 0.00225061 | 0.00222974 | 0.310000219 |
| 364 | average weekly red  wine incake | rs558882 | C | A | 0.216102 | -0.00271704 | 0.002506 | 0.27999998 |
| 365 | average weekly red  wine ingake | rs558887 | G | A | 0.307305 | 0.00166543 | 0.00224148 | 0.460000178 |
| 366 | average weekly red  wine intake | rs559231 | T | G | 0.392295 | 0.00292536 | 0.00212065 | 0.170000031 |
| 367 | average weekly red  wine inaake | rs56038322 | A | G | 0.311284 | 0.00151216 | 0.00224305 | 0.499999995 |
| 368 | average weekly red  wine ingake | rs56133507 | G | T | 0.196416 | 0.00226856 | 0.00259046 | 0.380000353 |
| 369 | average weekly red  wine intake | rs56143236 | T | C | 0.256449 | -0.00111844 | 0.00236743 | 0.640000038 |
| 370 | average weekly red  wine intake | rs56161855 | T | A | 0.132469 | -0.00107707 | 0.00304175 | 0.719999177 |
| 371 | average weekly red  wine incake | rs56203622 | C | T | 0.144884 | -0.00314414 | 0.00292816 | 0.27999998 |
| 372 | average weekly red  wine inaake | rs56211164 | A | G | 0.239657 | -5.21E-05 | 0.00241475 | 0.98000001 |
| 373 | average weekly red  wine incake | rs56352336 | C | T | 0.154712 | 0.00556733 | 0.00286486 | 0.0519996 |
| 374 | average weekly red  wine intake | rs56399737 | T | C | 0.449599 | 0.00122075 | 0.0020802 | 0.559999965 |
| 375 | average weekly red  wine inaake | rs56858768 | A | G | 0.297683 | -0.00429848 | 0.00226307 | 0.058000268 |
| 376 | average weekly red  wine ingake | rs56893062 | G | T | 0.304303 | -0.0016708 | 0.00224397 | 0.460000178 |
| 377 | average weekly red  wine intake | rs56930105 | T | C | 0.138923 | -0.00513576 | 0.00299985 | 0.08700015 |
| 378 | average weekly red  wine ingake | rs57263785 | G | A | 0.246021 | -0.0029618 | 0.00239577 | 0.220000162 |
| 379 | average weekly red  wine incake | rs57636386 | C | T | 0.084128 | 0.00356939 | 0.00372444 | 0.340000065 |

| 380 | average weekly red  wine inaake | rs57803 | A | G | 0.824072 | 0.00487643 | 0.00271547 | 0.072999519 |
| --- | --- | --- | --- | --- | --- | --- | --- | --- |
| 381 | average weekly red  wine incake | rs57989773 | C | T | 0.244561 | 0.00305031 | 0.00247041 | 0.220000162 |
| 382 | average weekly red  wine intake | rs587271 | T | C | 0.687595 | 0.00103432 | 0.00232217 | 0.660000098 |
| 383 | average weekly red  wine intake | rs58862095 | T | C | 0.420122 | 0.00441433 | 0.00209391 | 0.035000158 |
| 384 | average weekly red  wine intake | rs59068084 | T | G | 0.40978 | -0.00129578 | 0.00209851 | 0.540000299 |
| 385 | average weekly red  wine inaake | rs59086897 | A | T | 0.487384 | -0.000316377 | 0.00206098 | 0.880000056 |
| 386 | average weekly red  wine ingake | rs59227842 | G | A | 0.310798 | -0.0052368 | 0.00224739 | 0.02 |
| 387 | average weekly red  wine incake | rs594024 | C | T | 0.554431 | 0.000604124 | 0.00207739 | 0.770000487 |
| 388 | average weekly red  wine inaake | rs6000329 | A | G | 0.561597 | -0.000297142 | 0.00212245 | 0.889999986 |
| 389 | average weekly red  wine ingake | rs6023655 | G | A | 0.765515 | -0.00134202 | 0.00244508 | 0.580000009 |
| 390 | average weekly red  wine intake | rs6074432 | T | C | 0.545909 | 0.00280209 | 0.00207576 | 0.180000205 |
| 391 | average weekly red  wine intake | rs60764613 | T | G | 0.14529 | 0.0049668 | 0.0029391 | 0.090999708 |
| 392 | average weekly red  wine ingake | rs6092194 | G | T | 0.556555 | 0.00336763 | 0.00208107 | 0.11000008 |
| 393 | average weekly red  wine intake | rs6134916 | T | C | 0.493236 | 0.00309226 | 0.002068 | 0.129999895 |
| 394 | average weekly red  wine inaake | rs61740466 | A | G | 0.23811 | 0.00477628 | 0.00242069 | 0.047999863 |
| 395 | average weekly red  wine intake | rs61813324 | T | C | 0.13536 | -0.00675232 | 0.00305499 | 0.026999766 |
| 396 | average weekly red  wine inaake | rs61828641 | A | G | 0.109694 | 0.00342406 | 0.00329737 | 0.299999824 |
| 397 | average weekly red  wine intake | rs61871615 | T | C | 0.092068 | 0.00686823 | 0.00373492 | 0.065999402 |
| 398 | average weekly red  wine ingake | rs61903695 | G | A | 0.254559 | -0.00454469 | 0.00237011 | 0.054999659 |

| 399 | average weekly red  wine intake | rs61955525 | T | G | 0.196085 | 0.000160277 | 0.00263265 | 0.949999988 |
| --- | --- | --- | --- | --- | --- | --- | --- | --- |
| 400 | average weekly red  wine ingake | rs61992671 | G | A | 0.492536 | -0.00453203 | 0.00215591 | 0.035999793 |
| 401 | average weekly red  wine inaake | rs62007782 | A | G | 0.265788 | 0.00405426 | 0.00233373 | 0.081999273 |
| 402 | average weekly red  wine inaake | rs62020775 | A | T | 0.142388 | 0.00127875 | 0.00297434 | 0.670000304 |
| 403 | average weekly red  wine incake | rs62058023 | C | T | 0.134142 | 0.00360123 | 0.00303336 | 0.239999866 |
| 404 | average weekly red  wine ingake | rs62062168 | G | C | 0.199819 | 0.00119905 | 0.00257702 | 0.640000038 |
| 405 | average weekly red  wine incake | rs62072006 | C | A | 0.144721 | -0.000257376 | 0.00293778 | 0.929999896 |
| 406 | average weekly red  wine incake | rs62107261 | C | T | 0.048057 | -0.0058458 | 0.0048352 | 0.230000087 |
| 407 | average weekly red  wine incake | rs62190049 | C | G | 0.391059 | 0.00173187 | 0.00212944 | 0.419999719 |
| 408 | average weekly red  wine ingake | rs62240473 | G | A | 0.039001 | 0.000204166 | 0.00533359 | 0.969999923 |
| 409 | average weekly red  wine ingake | rs62241847 | G | A | 0.315839 | -0.00194876 | 0.00222075 | 0.380000353 |
| 410 | average weekly red  wine ingake | rs62379271 | G | T | 0.578511 | -0.00179648 | 0.00209375 | 0.390000353 |
| 411 | average weekly red  wine intake | rs62516785 | T | C | 0.064891 | 0.00215207 | 0.0041864 | 0.610000232 |
| 412 | average weekly red  wine intake | rs6265 | T | C | 0.188485 | -0.00243428 | 0.00263301 | 0.359999585 |
| 413 | average weekly red  wine intake | rs633284 | T | A | 0.516174 | 0.000206018 | 0.0020814 | 0.919999942 |
| 414 | average weekly red  wine inaake | rs6430068 | A | G | 0.107846 | -0.0063993 | 0.00334195 | 0.056000254 |
| 415 | average weekly red  wine inaake | rs6444950 | A | G | 0.237445 | -0.00285542 | 0.00242301 | 0.239999866 |
| 416 | average weekly red  wine incake | rs6445258 | C | T | 0.79231 | 0.00232168 | 0.00254059 | 0.359999585 |
| 417 | average weekly red  wine intake | rs6474856 | T | C | 0.641005 | 0.00280172 | 0.00215891 | 0.190000175 |

| 418 | average weekly red  wine inaake | rs6531639 | A | G | 0.248893 | 0.00369621 | 0.00243772 | 0.129999895 |
| --- | --- | --- | --- | --- | --- | --- | --- | --- |
| 419 | average weekly red  wine inaake | rs6545714 | A | G | 0.601813 | 0.00689157 | 0.00211021 | 0.001099993 |
| 420 | average weekly red  wine incake | rs6560906 | C | T | 0.691916 | -0.00558605 | 0.00223347 | 0.011999966 |
| 421 | average weekly red  wine inaake | rs6561937 | A | T | 0.753737 | 0.00861945 | 0.00239988 | 0.000329997 |
| 422 | average weekly red  wine incake | rs6567160 | C | T | 0.231288 | 0.00167577 | 0.00244505 | 0.48999991 |
| 423 | average weekly red  wine inaake | rs6575340 | A | G | 0.635298 | 4.39493e-05 | 0.00214505 | 0.98000001 |
| 424 | average weekly red  wine ingake | rs6597975 | G | C | 0.543998 | -0.00293715 | 0.00208033 | 0.160000006 |
| 425 | average weekly red  wine ingake | rs6606580 | G | A | 0.588913 | 0.00142916 | 0.00209246 | 0.48999991 |
| 426 | average weekly red  wine intake | rs66679256 | T | C | 0.445658 | 0.00367923 | 0.00207655 | 0.075999371 |
| 427 | average weekly red  wine intake | rs6669189 | T | C | 0.402015 | -0.00164375 | 0.00210974 | 0.440000328 |
| 428 | average weekly red  wine incake | rs6682438 | C | T | 0.673937 | 0.00564768 | 0.00219818 | 0.01 |
| 429 | average weekly red  wine incake | rs6696828 | C | G | 0.304469 | -4.78E-05 | 0.00223773 | 0.98000001 |
| 430 | average weekly red  wine incake | rs6705567 | C | T | 0.376627 | -0.00148885 | 0.00214315 | 0.48999991 |
| 431 | average weekly red  wine ingake | rs6707827 | G | A | 0.702758 | -0.00522563 | 0.00227138 | 0.021000034 |
| 432 | average weekly red  wine ingake | rs6710091 | G | C | 0.349258 | 0.0018587 | 0.00216019 | 0.390000353 |
| 433 | average weekly red  wine incake | rs6713781 | C | G | 0.401578 | 0.00340807 | 0.00212121 | 0.11000008 |
| 434 | average weekly red  wine intake | rs6725931 | T | C | 0.847054 | -0.00564633 | 0.00286827 | 0.049000442 |
| 435 | average weekly red  wine ingake | rs6744646 | G | A | 0.827926 | 0.00289487 | 0.00273018 | 0.290000001 |
| 436 | average weekly red  wine inaake | rs6752979 | A | G | 0.317006 | -0.00367724 | 0.00221673 | 0.096999613 |

| 437 | average weekly red  wine incake | rs67609008 | C | T | 0.283857 | -0.00346049 | 0.00229467 | 0.129999895 |
| --- | --- | --- | --- | --- | --- | --- | --- | --- |
| 438 | average weekly red  wine inaake | rs6774894 | A | T | 0.357631 | -0.00132528 | 0.00215132 | 0.540000299 |
| 439 | average weekly red  wine incake | rs6791296 | C | T | 0.88165 | 0.00362766 | 0.00320309 | 0.259999792 |
| 440 | average weekly red  wine incake | rs6805758 | C | A | 0.331343 | 0.00181163 | 0.00219194 | 0.410000135 |
| 441 | average weekly red  wine inaake | rs6824271 | A | T | 0.270745 | -6.23E-05 | 0.00236498 | 0.98000001 |
| 442 | average weekly red  wine inaake | rs6831088 | A | G | 0.639427 | -0.00435842 | 0.00215058 | 0.043000153 |
| 443 | average weekly red  wine ingake | rs6834120 | G | C | 0.137703 | 0.00284952 | 0.00302977 | 0.349999964 |
| 444 | average weekly red  wine intake | rs6843852 | T | C | 0.507182 | 0.00105132 | 0.00206121 | 0.610000232 |
| 445 | average weekly red  wine intake | rs6909685 | T | C | 0.326822 | 0.0031004 | 0.00220758 | 0.160000006 |
| 446 | average weekly red  wine ingake | rs6922607 | G | A | 0.189441 | -0.00258619 | 0.00262749 | 0.320000016 |
| 447 | average weekly red  wine incake | rs6938973 | C | T | 0.599963 | -0.0121708 | 0.00210844 | 7.79991733536343e-09 |
| 448 | average weekly red  wine inaake | rs6950388 | A | G | 0.795106 | -0.000874327 | 0.00255512 | 0.730000235 |
| 449 | average weekly red  wine incake | rs6962980 | C | A | 0.557463 | 0.00396885 | 0.00207335 | 0.056000254 |
| 450 | average weekly red  wine ingake | rs698147 | G | A | 0.54427 | -0.00231362 | 0.00207095 | 0.259999792 |
| 451 | average weekly red  wine ingake | rs6998660 | G | A | 0.451286 | -0.00381997 | 0.0020745 | 0.065999402 |
| 452 | average weekly red  wine ingake | rs7024334 | G | T | 0.779576 | -0.00169343 | 0.00248814 | 0.499999995 |
| 453 | average weekly red  wine intake | rs7027304 | T | C | 0.651747 | 0.000586801 | 0.00217489 | 0.789999834 |
| 454 | average weekly red  wine ingake | rs7034554 | G | A | 0.37425 | 0.00064226 | 0.00212914 | 0.760000713 |
| 455 | average weekly red  wine incake | rs7038943 | C | T | 0.338682 | -0.000719317 | 0.00217525 | 0.740000478 |

| 456 | average weekly red  wine incake | rs704061 | C | T | 0.454341 | 0.000317643 | 0.00206908 | 0.880000056 |
| --- | --- | --- | --- | --- | --- | --- | --- | --- |
| 457 | average weekly red  wine inaake | rs705145 | A | C | 0.344664 | 0.00239374 | 0.00216956 | 0.270000147 |
| 458 | average weekly red  wine intake | rs7070670 | T | C | 0.328294 | 0.00180911 | 0.00220904 | 0.410000135 |
| 459 | average weekly red  wine incake | rs7081254 | C | T | 0.206411 | 0.00450316 | 0.00255445 | 0.077999173 |
| 460 | average weekly red  wine ingake | rs7109581 | G | T | 0.419945 | -0.000973709 | 0.00209201 | 0.640000038 |
| 461 | average weekly red  wine inaake | rs7124681 | A | C | 0.406754 | -0.00551806 | 0.00209312 | 0.008400014 |
| 462 | average weekly red  wine inaake | rs7132908 | A | G | 0.383524 | 0.00110396 | 0.00212183 | 0.599999654 |
| 463 | average weekly red  wine ingake | rs713598 | G | C | 0.4001 | -0.0173682 | 0.00210277 | 1.50003019045595e-16 |
| 464 | average weekly red  wine inaake | rs71495038 | A | G | 0.076625 | 0.00299581 | 0.0038789 | 0.440000328 |
| 465 | average weekly red  wine intake | rs71511072 | T | G | 0.176909 | -0.000743231 | 0.00271585 | 0.780000714 |
| 466 | average weekly red  wine inaake | rs7201895 | A | G | 0.353885 | -0.00287521 | 0.00216993 | 0.190000175 |
| 467 | average weekly red  wine ingake | rs7206608 | G | C | 0.321358 | -0.00478857 | 0.00221081 | 0.029999913 |
| 468 | average weekly red  wine incake | rs7218014 | C | T | 0.19643 | -0.00369651 | 0.00260231 | 0.160000006 |
| 469 | average weekly red  wine intake | rs7232171 | T | G | 0.582314 | -0.00134931 | 0.00209152 | 0.519999589 |
| 470 | average weekly red  wine intake | rs723672 | T | C | 0.430844 | -0.00358962 | 0.00209359 | 0.086000307 |
| 471 | average weekly red  wine incake | rs7259070 | C | T | 0.595344 | 0.00143242 | 0.00211764 | 0.499999995 |
| 472 | average weekly red  wine inaake | rs72634826 | A | G | 0.259826 | 0.00789284 | 0.00238373 | 0.000929994 |
| 473 | average weekly red  wine incake | rs72649373 | C | T | 0.142904 | -0.00116385 | 0.00300961 | 0.699999936 |
| 474 | average weekly red  wine ingake | rs72673947 | G | A | 0.106718 | 0.000476649 | 0.0033553 | 0.889999986 |

| 475 | average weekly red  wine ingake | rs72744924 | G | A | 0.089326 | 0.00751701 | 0.00364028 | 0.038999586 |
| --- | --- | --- | --- | --- | --- | --- | --- | --- |
| 476 | average weekly red  wine inaake | rs72813172 | A | G | 0.040361 | 0.000198588 | 0.00533789 | 0.969999923 |
| 477 | average weekly red  wine ingake | rs7283057 | G | A | 0.746707 | 0.00128588 | 0.00238535 | 0.589999984 |
| 478 | average weekly red  wine intake | rs72866851 | T | A | 0.101613 | 0.00483822 | 0.00340946 | 0.160000006 |
| 479 | average weekly red  wine intake | rs72892910 | T | G | 0.171123 | -0.00673007 | 0.00274419 | 0.014000063 |
| 480 | average weekly red  wine inaake | rs72915955 | A | G | 0.161106 | 0.00790667 | 0.00282238 | 0.005099998 |
| 481 | average weekly red  wine inaake | rs72925179 | A | G | 0.181709 | 0.00217157 | 0.00267566 | 0.419999719 |
| 482 | average weekly red  wine inaake | rs72976986 | A | G | 0.190794 | 0.00142357 | 0.00264772 | 0.589999984 |
| 483 | average weekly red  wine inaake | rs73026725 | A | C | 0.153862 | -0.00481103 | 0.00285941 | 0.09200046 |
| 484 | average weekly red  wine incake | rs73034216 | C | T | 0.173111 | 0.00123068 | 0.0027322 | 0.649999466 |
| 485 | average weekly red  wine incake | rs73052033 | C | T | 0.185131 | -0.00107939 | 0.00265918 | 0.680000137 |
| 486 | average weekly red  wine inaake | rs7306534 | A | G | 0.62156 | 0.00405462 | 0.00214432 | 0.058999727 |
| 487 | average weekly red  wine incake | rs7306544 | C | T | 0.105088 | 0.00427281 | 0.00336748 | 0.200000002 |
| 488 | average weekly red  wine incake | rs73124396 | C | T | 0.204876 | -0.00354129 | 0.00256084 | 0.170000031 |
| 489 | average weekly red  wine intake | rs73142879 | T | C | 0.192667 | 0.0030712 | 0.00262459 | 0.239999866 |
| 490 | average weekly red  wine ingake | rs73193736 | G | A | 0.244598 | 0.00210379 | 0.00241696 | 0.380000353 |
| 491 | average weekly red  wine intake | rs73213484 | T | A | 0.140948 | 0.00169412 | 0.00296453 | 0.570000189 |
| 492 | average weekly red  wine inaake | rs7331420 | A | G | 0.286444 | 0.00435909 | 0.00229065 | 0.056999363 |
| 493 | average weekly red  wine intake | rs73529119 | T | C | 0.113635 | -0.00111754 | 0.00326237 | 0.730000235 |

| 494 | average weekly red  wine ingake | rs7357754 | G | A | 0.498888 | -0.0026457 | 0.00206866 | 0.200000002 |
| --- | --- | --- | --- | --- | --- | --- | --- | --- |
| 495 | average weekly red  wine intake | rs73601548 | T | C | 0.114163 | 0.00571491 | 0.00325504 | 0.079000529 |
| 496 | average weekly red  wine incake | rs73985439 | C | A | 0.306919 | -0.0068004 | 0.00224026 | 0.002399993 |
| 497 | average weekly red  wine intake | rs74252325 | T | C | 0.211873 | -0.00229078 | 0.00246917 | 0.349999964 |
| 498 | average weekly red  wine ingake | rs7442885 | G | C | 0.214909 | 0.0145178 | 0.00251755 | 8.1000929031245e-09 |
| 499 | average weekly red  wine intake | rs745249 | T | C | 0.281896 | -0.00198208 | 0.00229815 | 0.390000353 |
| 500 | average weekly red  wine incake | rs74750282 | C | T | 0.086982 | -0.00186265 | 0.00367288 | 0.610000232 |
| 501 | average weekly red  wine inaake | rs74887628 | A | G | 0.035222 | -0.011833 | 0.00575633 | 0.040000001 |
| 502 | average weekly red  wine ingake | rs7498665 | G | A | 0.397661 | -0.009207 | 0.00210949 | 1.29998996537546e-05 |
| 503 | average weekly red  wine inaake | rs7519259 | A | G | 0.529133 | -0.000708764 | 0.00207352 | 0.730000235 |
| 504 | average weekly red  wine ingake | rs7546040 | G | C | 0.732216 | 0.0117688 | 0.00232646 | 4.20000686246631e-07 |
| 505 | average weekly red  wine ingake | rs754635 | G | C | 0.886263 | 0.00224557 | 0.00324594 | 0.48999991 |
| 506 | average weekly red  wine intake | rs75499503 | T | C | 0.220681 | 0.0121257 | 0.00252419 | 1.60000006389793e-06 |
| 507 | average weekly red  wine incake | rs7568228 | C | G | 0.527243 | 0.00369037 | 0.00206377 | 0.073999707 |
| 508 | average weekly red  wine ingake | rs7571496 | G | A | 0.260369 | 0.00149068 | 0.00236052 | 0.530000159 |
| 509 | average weekly red  wine inaake | rs760644 | A | G | 0.820294 | 0.00272449 | 0.00268319 | 0.310000219 |
| 510 | average weekly red  wine incake | rs76183894 | C | T | 0.080779 | 0.00324897 | 0.00380919 | 0.390000353 |
| 511 | average weekly red  wine inaake | rs7619139 | A | T | 0.589098 | 0.00338083 | 0.00210212 | 0.11000008 |
| 512 | average weekly red  wine intake | rs7621422 | T | C | 0.498825 | 0.000926785 | 0.00206868 | 0.649999466 |

| 513 | average weekly red  wine intake | rs76267866 | T | A | 0.205521 | 0.00297721 | 0.00255255 | 0.239999866 |
| --- | --- | --- | --- | --- | --- | --- | --- | --- |
| 514 | average weekly red  wine intake | rs76469486 | T | A | 0.079737 | 0.00351743 | 0.00380754 | 0.359999585 |
| 515 | average weekly red  wine inaake | rs765874 | A | T | 0.489449 | 0.000237588 | 0.00206266 | 0.910000016 |
| 516 | average weekly red  wine ingake | rs76702514 | G | C | 0.210818 | 0.00129929 | 0.00254534 | 0.610000232 |
| 517 | average weekly red  wine inaake | rs7683836 | A | G | 0.557344 | 0.00518366 | 0.00208126 | 0.0129999 |
| 518 | average weekly red  wine ingake | rs7704382 | G | C | 0.433226 | -0.0033953 | 0.00208392 | 0.1 |
| 519 | average weekly red  wine ingake | rs7708584 | G | A | 0.572511 | -0.000466727 | 0.00208073 | 0.82000009 |
| 520 | average weekly red  wine ingake | rs7742698 | G | T | 0.366063 | 0.00174606 | 0.00214334 | 0.419999719 |
| 521 | average weekly red  wine inaake | rs7761673 | A | T | 0.220581 | 0.00141022 | 0.00249152 | 0.570000189 |
| 522 | average weekly red  wine ingake | rs7762794 | G | A | 0.284509 | -0.00155942 | 0.00228344 | 0.48999991 |
| 523 | average weekly red  wine inaake | rs7774 | A | C | 0.310041 | -0.00389224 | 0.00224102 | 0.081999273 |
| 524 | average weekly red  wine inaake | rs7776021 | A | G | 0.286933 | -0.00319937 | 0.00228227 | 0.160000006 |
| 525 | average weekly red  wine ingake | rs78012460 | G | A | 0.022664 | 0.0126405 | 0.00694209 | 0.069000144 |
| 526 | average weekly red  wine intake | rs7805441 | T | C | 0.501386 | -0.00407204 | 0.00207434 | 0.05 |
| 527 | average weekly red  wine inaake | rs7819514 | A | G | 0.337403 | 0.00341593 | 0.00218581 | 0.119999932 |
| 528 | average weekly red  wine intake | rs7828631 | T | C | 0.109692 | -0.00100925 | 0.00330653 | 0.760000713 |
| 529 | average weekly red  wine inaake | rs7833023 | A | C | 0.725358 | 0.00118208 | 0.00231967 | 0.610000232 |
| 530 | average weekly red  wine incake | rs784257 | C | T | 0.811412 | -0.00824283 | 0.0026472 | 0.00179999 |
| 531 | average weekly red  wine incake | rs78605811 | C | A | 0.053602 | -0.00765239 | 0.00466416 | 0.1 |

| 532 | average weekly red  wine ingake | rs78886584 | G | A | 0.489454 | -0.00174962 | 0.002087 | 0.400000008 |
| --- | --- | --- | --- | --- | --- | --- | --- | --- |
| 533 | average weekly red  wine intake | rs7893571 | T | G | 0.66599 | 0.00136564 | 0.0021915 | 0.530000159 |
| 534 | average weekly red  wine incake | rs7900590 | C | T | 0.942999 | -0.0090235 | 0.00458439 | 0.049000442 |
| 535 | average weekly red  wine incake | rs79027764 | C | T | 0.021023 | 0.00029203 | 0.00717488 | 0.969999923 |
| 536 | average weekly red  wine intake | rs7924036 | T | G | 0.503959 | 0.0116464 | 0.00206248 | 1.60000006389793e-08 |
| 537 | average weekly red  wine inaake | rs7925100 | A | G | 0.395935 | 0.000265281 | 0.00210958 | 0.89999998 |
| 538 | average weekly red  wine incake | rs7942037 | C | G | 0.360362 | 0.00231828 | 0.00214742 | 0.27999998 |
| 539 | average weekly red  wine ingake | rs7944782 | G | T | 0.510125 | -0.00087272 | 0.0020737 | 0.670000304 |
| 540 | average weekly red  wine inaake | rs7947143 | A | G | 0.163737 | -0.00388997 | 0.0027909 | 0.160000006 |
| 541 | average weekly red  wine ingake | rs7975187 | G | A | 0.213357 | -0.00159371 | 0.00251723 | 0.530000159 |
| 542 | average weekly red  wine intake | rs79780963 | T | C | 0.077225 | -0.000860168 | 0.0038573 | 0.82000009 |
| 543 | average weekly red  wine intake | rs79906980 | T | C | 0.159908 | -0.00160798 | 0.00282096 | 0.570000189 |
| 544 | average weekly red  wine incake | rs79966207 | C | T | 0.176386 | 0.0027567 | 0.00270555 | 0.310000219 |
| 545 | average weekly red  wine inaake | rs7996639 | A | G | 0.448554 | 0.000893535 | 0.00208674 | 0.670000304 |
| 546 | average weekly red  wine intake | rs80135274 | T | A | 0.06997 | 0.00201915 | 0.00405361 | 0.620000443 |
| 547 | average weekly red  wine inaake | rs8015400 | A | C | 0.677481 | 0.00123955 | 0.0022058 | 0.570000189 |
| 548 | average weekly red  wine inaake | rs8020365 | A | T | 0.220137 | -0.00721644 | 0.0024977 | 0.003899959 |
| 549 | average weekly red  wine intake | rs8024137 | T | A | 0.84775 | -0.00143957 | 0.00287699 | 0.620000443 |
| 550 | average weekly red  wine ingake | rs8025516 | G | T | 0.645886 | 0.0027993 | 0.00216012 | 0.200000002 |

| 551 | average weekly red  wine incake | rs8033510 | C | T | 0.636771 | -0.003317 | 0.00215107 | 0.119999932 |
| --- | --- | --- | --- | --- | --- | --- | --- | --- |
| 552 | average weekly red  wine intake | rs8063946 | T | C | 0.054642 | -0.0118089 | 0.00454861 | 0.009400046 |
| 553 | average weekly red  wine incake | rs8076669 | C | T | 0.559958 | -0.000559675 | 0.00207914 | 0.789999834 |
| 554 | average weekly red  wine inaake | rs8089514 | A | T | 0.368115 | 0.00483274 | 0.00216137 | 0.025 |
| 555 | average weekly red  wine ingake | rs8112818 | G | A | 0.400741 | 0.0021555 | 0.00210968 | 0.310000219 |
| 556 | average weekly red  wine incake | rs8124896 | C | T | 0.100656 | -0.00348132 | 0.00343446 | 0.310000219 |
| 557 | average weekly red  wine ingake | rs8137518 | G | C | 0.0573 | -0.00804326 | 0.00452909 | 0.075999371 |
| 558 | average weekly red  wine incake | rs815163 | C | T | 0.562637 | 0.00214213 | 0.00207572 | 0.299999824 |
| 559 | average weekly red  wine incake | rs8176166 | C | T | 0.148216 | 0.0062578 | 0.00291316 | 0.032000002 |
| 560 | average weekly red  wine ingake | rs852042 | G | A | 0.758789 | -0.00195705 | 0.00240782 | 0.419999719 |
| 561 | average weekly red  wine ingake | rs861578 | G | C | 0.554182 | -0.00367266 | 0.00208962 | 0.079000529 |
| 562 | average weekly red  wine intake | rs862320 | T | C | 0.410784 | 0.00322154 | 0.00209932 | 0.119999932 |
| 563 | average weekly red  wine intake | rs878627 | T | C | 0.379144 | 0.00107736 | 0.0021478 | 0.620000443 |
| 564 | average weekly red  wine inaake | rs909892 | A | G | 0.13531 | 0.00814225 | 0.00302794 | 0.007199959 |
| 565 | average weekly red  wine ingake | rs923994 | G | A | 0.783564 | 0.00554574 | 0.00251142 | 0.026999766 |
| 566 | average weekly red  wine inaake | rs9294260 | A | G | 0.476612 | -0.000981806 | 0.00207764 | 0.640000038 |
| 567 | average weekly red  wine inaake | rs9299525 | A | G | 0.409823 | -0.00326956 | 0.00209459 | 0.119999932 |
| 568 | average weekly red  wine intake | rs9349235 | T | C | 0.410823 | -0.00103599 | 0.00209851 | 0.620000443 |
| 569 | average weekly red  wine inaake | rs935166 | A | G | 0.506805 | 0.00177102 | 0.00206433 | 0.390000353 |

| 570 | average weekly red  wine incake | rs9366863 | C | T | 0.672606 | -0.000141542 | 0.00219299 | 0.949999988 |
| --- | --- | --- | --- | --- | --- | --- | --- | --- |
| 571 | average weekly red  wine intake | rs9375702 | T | C | 0.691948 | -0.00231351 | 0.0022345 | 0.299999824 |
| 572 | average weekly red  wine inaake | rs9388446 | A | T | 0.515387 | -0.000166489 | 0.00206769 | 0.9400001 |
| 573 | average weekly red  wine intake | rs9395885 | T | C | 0.083192 | 0.000577457 | 0.00372954 | 0.880000056 |
| 574 | average weekly red  wine intake | rs9461887 | T | C | 0.277394 | 0.00101515 | 0.00229898 | 0.660000098 |
| 575 | average weekly red  wine intake | rs9463175 | T | C | 0.337692 | 0.000201868 | 0.00219577 | 0.929999896 |
| 576 | average weekly red  wine incake | rs9478496 | C | T | 0.16373 | -0.00699172 | 0.00279838 | 0.011999966 |
| 577 | average weekly red  wine ingake | rs9515446 | G | A | 0.448008 | -0.003285 | 0.0020741 | 0.11000008 |
| 578 | average weekly red  wine intake | rs9522180 | T | C | 0.553561 | 0.00220354 | 0.00207595 | 0.290000001 |
| 579 | average weekly red  wine inaake | rs9571687 | A | C | 0.329289 | -0.000181449 | 0.00219781 | 0.929999896 |
| 580 | average weekly red  wine ingake | rs9585326 | G | A | 0.53352 | 0.0109987 | 0.00207001 | 1.09999319893519e-07 |
| 581 | average weekly red  wine incake | rs961498 | C | G | 0.502982 | 0.0029497 | 0.0020805 | 0.160000006 |
| 582 | average weekly red  wine ingake | rs9638713 | G | A | 0.974955 | 0.00689621 | 0.00664774 | 0.299999824 |
| 583 | average weekly red  wine ingake | rs9673839 | G | A | 0.491035 | -0.00746083 | 0.00207361 | 0.00032 |
| 584 | average weekly red  wine ingake | rs9674487 | G | C | 0.001329 | 0.0278121 | 0.0298698 | 0.349999964 |
| 585 | average weekly red  wine inaake | rs979724 | A | G | 0.300872 | -0.000413848 | 0.00224653 | 0.84999995 |
| 586 | average weekly red  wine inaake | rs9830592 | A | C | 0.58189 | -0.00381454 | 0.00208875 | 0.06800017 |
| 587 | average weekly red  wine inaake | rs9839081 | A | G | 0.325851 | -0.00101927 | 0.00223525 | 0.649999466 |
| 588 | average weekly red  wine incake | rs9843653 | C | T | 0.510964 | -0.00704833 | 0.00206431 | 0.00064 |

| 589 | average weekly red  wine intake | rs9876664 | T | G | 0.374986 | 0.00330361 | 0.00213359 | 0.119999932 |
| --- | --- | --- | --- | --- | --- | --- | --- | --- |
| 590 | average weekly red  wine intake | rs9888533 | T | C | 0.538262 | -0.00353354 | 0.00210297 | 0.092999369 |
| 591 | average weekly red  wine incake | rs9926784 | C | T | 0.183289 | -0.00411712 | 0.00266363 | 0.119999932 |
| 592 | average weekly red  wine ingake | rs9931586 | G | C | 0.270109 | -0.00206694 | 0.0023378 | 0.380000353 |
| 593 | average weekly red  wine ingake | rs9951619 | G | T | 0.767937 | -0.000567605 | 0.00245682 | 0.82000009 |
| 594 | average weekly red  wine incake | rs995460 | C | A | 0.459618 | -0.00264651 | 0.0020696 | 0.200000002 |
| 595 | average weekly red  wine incake | rs9954755 | C | G | 0.538678 | -0.00285328 | 0.00207646 | 0.170000031 |
| 596 | average weekly red  wine incake | rs9992189 | C | G | 0.599903 | -0.0036756 | 0.00210497 | 0.081000929 |

| **Table S11 Instrumental variables used in MVMR analysis of BMI to hypertension.** | | | | | | | | |
| --- | --- | --- | --- | --- | --- | --- | --- | --- |
|  | **Exposure** | **SNP** | **effect_allele** | **other_allele** | **eaf** | **beta** | **se** | **pval** |
| 1 | BMI | rs10063055 | T | C | 0.253365 | 0.0136775 | 0.00226991 | 1.70000422215636e-09 |
| 2 | BMI | rs10063744 | G | C | 0.284106 | -0.0116209 | 0.00220039 | 1.29998996537546e-07 |
| 3 | BMI | rs10160769 | C | G | 0.21751 | -0.0155862 | 0.00242199 | 1.19999655704811e-10 |
| 4 | BMI | rs10172483 | T | A | 0.234856 | 0.0127394 | 0.00232602 | 4.3000152915127e-08 |
| 5 | BMI | rs10182416 | G | A | 0.512223 | 0.0130378 | 0.00197134 | 3.69998531172859e-11 |
| 6 | BMI | rs10184537 | T | C | 0.339341 | -0.016303 | 0.00208355 | 5.10035023959446e-15 |
| 7 | BMI | rs1019240 | T | A | 0.643117 | 0.0107588 | 0.0020717 | 2.10000341026661e-07 |
| 8 | BMI | rs10280836 | G | T | 0.771381 | 0.00316176 | 0.00235708 | 0.180000205 |
| 9 | BMI | rs10402950 | C | T | 0.289347 | 0.0135545 | 0.00218692 | 5.69993627094142e-10 |
| 10 | BMI | rs10423928 | A | T | 0.194358 | -0.0340136 | 0.00249869 | 3.40016505804072e-42 |
| 11 | BMI | rs1048637 | G | T | 0.452062 | 0.0103286 | 0.00198219 | 1.89998424621473e-07 |
| 12 | BMI | rs10505836 | C | A | 0.860012 | 0.0184851 | 0.00287086 | 1.19999655704811e-10 |
| 13 | BMI | rs10510025 | T | C | 0.247013 | 0.0175643 | 0.00229886 | 2.19988510904925e-14 |
| 14 | BMI | rs1064213 | A | G | 0.478377 | 0.0149301 | 0.00197196 | 3.69998531172859e-14 |
| 15 | BMI | rs10742752 | C | T | 0.612239 | 0.0117979 | 0.00202961 | 6.10000231740111e-09 |
| 16 | BMI | rs10760277 | T | C | 0.385049 | 0.0138734 | 0.00203825 | 1e-11 |
| 17 | BMI | rs10771041 | T | C | 0.116102 | 0.0213233 | 0.0030884 | 5.00034534976978e-12 |
| 18 | BMI | rs10780248 | A | G | 0.559454 | -0.0121232 | 0.00199415 | 1.19999655704811e-09 |
| 19 | BMI | rs1078141 | T | C | 0.383867 | 0.0142239 | 0.00205862 | 4.90004422793237e-12 |
| 20 | BMI | rs10799778 | G | T | 0.833718 | -0.0182237 | 0.00264876 | 6.00067353864116e-12 |
| 21 | BMI | rs10824211 | T | C | 0.139491 | 0.0207688 | 0.00286631 | 4.30031233630544e-13 |
| 22 | BMI | rs10827380 | T | C | 0.31429 | 0.0108257 | 0.00213337 | 3.89995862874433e-07 |
| 23 | BMI | rs10832778 | G | C | 0.623038 | 0.0115628 | 0.00203985 | 1.40000633223953e-08 |
| 24 | BMI | rs10887578 | C | G | 0.497499 | 0.0133212 | 0.00198909 | 2.09990670376243e-11 |
| 25 | BMI | rs10894670 | A | C | 0.550568 | -0.0101587 | 0.00199063 | 3.29997006285229e-07 |
| 26 | BMI | rs10903791 | A | G | 0.603774 | 0.0106031 | 0.00202628 | 1.70000422215637e-07 |
| 27 | BMI | rs10905688 | G | A | 0.902113 | -0.0174369 | 0.00333065 | 1.60000006389793e-07 |
| 28 | BMI | rs10915821 | C | T | 0.777232 | -0.0124982 | 0.00236506 | 1.29998996537546e-07 |
| 29 | BMI | rs10925183 | A | G | 0.607078 | 0.000665135 | 0.00201706 | 0.740000478 |
| 30 | BMI | rs10954277 | A | G | 0.291557 | 0.0116359 | 0.00217846 | 9.20004602515511e-08 |
| 31 | BMI | rs10960293 | T | G | 0.333719 | -0.0140984 | 0.00210242 | 1.99986186963274e-11 |
| 32 | BMI | rs10982030 | C | T | 0.054911 | -0.0231068 | 0.00435006 | 1.09999319893519e-07 |
| 33 | BMI | rs10989067 | A | G | 0.31593 | 0.0169514 | 0.00212349 | 1.39990962656834e-15 |
| 34 | BMI | rs11001963 | T | C | 0.581338 | 0.0116794 | 0.00201921 | 7.299951924762e-09 |
| 35 | BMI | rs11004108 | C | T | 0.262171 | 0.0111431 | 0.00225122 | 7.39997069733888e-07 |
| 36 | BMI | rs11009685 | T | C | 0.244096 | -0.0130834 | 0.00230766 | 1.40000633223953e-08 |

| 37 | BMI | rs11012732 | G | A | 0.331683 | 0.0216425 | 0.00210126 | 7.10068096254531e-25 |
| --- | --- | --- | --- | --- | --- | --- | --- | --- |
| 38 | BMI | rs11021354 | G | A | 0.393572 | -0.00163426 | 0.00203261 | 0.419999719 |
| 39 | BMI | rs11057072 | G | A | 0.233706 | 0.0128422 | 0.00233829 | 4.00000007987242e-08 |
| 40 | BMI | rs11066651 | C | A | 0.293807 | 0.0110672 | 0.00216832 | 3.29997006285229e-07 |
| 41 | BMI | rs11079849 | T | C | 0.328531 | -0.020093 | 0.00211163 | 1.800113960418e-21 |
| 42 | BMI | rs11097236 | A | G | 0.201635 | 0.0127561 | 0.00245889 | 2.10000341026661e-07 |
| 43 | BMI | rs11099020 | T | C | 0.640609 | -0.0142038 | 0.00206202 | 5.60015438761557e-12 |
| 44 | BMI | rs11115160 | A | G | 0.237797 | -0.0130768 | 0.00233641 | 2.19998641983516e-08 |
| 45 | BMI | rs11122450 | G | T | 0.611739 | -0.0116316 | 0.00202425 | 9.09997082601283e-09 |
| 46 | BMI | rs11134679 | G | A | 0.684753 | 0.0182459 | 0.00213286 | 1.20005182020427e-17 |
| 47 | BMI | rs11136566 | C | A | 0.38661 | -0.0104732 | 0.00204705 | 3.09998790941119e-07 |
| 48 | BMI | rs11150462 | A | T | 0.632134 | -0.0110541 | 0.00205424 | 7.39997069733888e-08 |
| 49 | BMI | rs11150745 | G | A | 0.317711 | -0.0211611 | 0.00212961 | 2.90001336905406e-23 |
| 50 | BMI | rs111598585 | T | C | 0.208671 | -0.0142245 | 0.00243798 | 5.39995325071584e-09 |
| 51 | BMI | rs11161044 | C | G | 0.807458 | 0.0169247 | 0.00251308 | 1.5999263828686e-11 |
| 52 | BMI | rs11165643 | T | C | 0.590103 | 0.0193319 | 0.00200337 | 4.90004422793237e-22 |
| 53 | BMI | rs111689389 | C | G | 0.282614 | -0.0136706 | 0.00219522 | 4.70002318179801e-10 |
| 54 | BMI | rs11218510 | A | G | 0.40047 | -0.0144616 | 0.00202121 | 8.4004006526377e-13 |
| 55 | BMI | rs11250094 | C | G | 0.548052 | -0.0202907 | 0.00199209 | 2.2998525866862e-24 |
| 56 | BMI | rs1126930 | C | G | 0.035271 | 0.0322695 | 0.00535892 | 1.70000422215636e-09 |
| 57 | BMI | rs112765062 | T | C | 0.093205 | -0.0171796 | 0.00341759 | 4.99999995007974e-07 |
| 58 | BMI | rs113079574 | T | C | 0.19279 | -0.0155896 | 0.00251598 | 5.8000267960159e-10 |
| 59 | BMI | rs113603865 | T | C | 0.21205 | 0.0186013 | 0.00242689 | 1.80011396041801e-14 |
| 60 | BMI | rs113624107 | A | G | 0.22586 | 0.0150461 | 0.00236876 | 2.10000341026661e-10 |
| 61 | BMI | rs113936755 | C | T | 0.090839 | 0.0199402 | 0.00370152 | 7.1999586127519e-08 |
| 62 | BMI | rs114167666 | A | G | 0.024969 | 0.0336994 | 0.00634251 | 1.09999319893519e-07 |
| 63 | BMI | rs114313565 | A | G | 0.027039 | -0.0167324 | 0.00627003 | 0.007599937 |
| 64 | BMI | rs114875897 | A | C | 0.019331 | 0.0366242 | 0.0071458 | 2.99999133271615e-07 |
| 65 | BMI | rs11525873 | C | T | 0.097701 | -0.0239781 | 0.00333603 | 6.59933234263564e-13 |
| 66 | BMI | rs11607476 | C | A | 0.486574 | 0.015712 | 0.00199179 | 3.10027344199819e-15 |
| 67 | BMI | rs11608710 | G | T | 0.062119 | 0.0248489 | 0.00415281 | 2.19998641983517e-09 |
| 68 | BMI | rs11610621 | A | T | 0.148174 | 0.0164869 | 0.00278318 | 3.09998790941119e-09 |
| 69 | BMI | rs11634851 | G | C | 0.465442 | 0.0118671 | 0.00198456 | 2.19998641983517e-09 |
| 70 | BMI | rs116374395 | A | G | 0.035444 | 0.0318817 | 0.00536236 | 2.80001269243454e-09 |
| 71 | BMI | rs11642090 | C | T | 0.373536 | 0.0114184 | 0.00205925 | 2.90001336905407e-08 |
| 72 | BMI | rs11656076 | A | G | 0.224774 | -0.0154285 | 0.00237054 | 7.59976214361039e-11 |
| 73 | BMI | rs1167311 | A | G | 0.681309 | -0.0192649 | 0.00213109 | 1.5999263828686e-19 |
| 74 | BMI | rs116806934 | A | G | 0.023319 | -0.0328753 | 0.00658826 | 5.99998272533644e-07 |

| 75 | BMI | rs11691869 | A | C | 0.362038 | -0.0193158 | 0.00205562 | 5.60015438761557e-21 |
| --- | --- | --- | --- | --- | --- | --- | --- | --- |
| 76 | BMI | rs116952199 | C | A | 0.037526 | -0.0286789 | 0.00538943 | 1e-07 |
| 77 | BMI | rs11699828 | A | G | 0.035832 | -0.0335418 | 0.00582677 | 8.60003066888856e-09 |
| 78 | BMI | rs11709402 | G | A | 0.278732 | 0.0228192 | 0.00220796 | 4.90004422793237e-25 |
| 79 | BMI | rs117118217 | C | G | 0.017711 | 0.0448186 | 0.00788004 | 1.29998996537546e-08 |
| 80 | BMI | rs117435593 | A | G | 0.045356 | -0.0252924 | 0.00481316 | 1.49999565138204e-07 |
| 81 | BMI | rs11757278 | C | T | 0.303879 | -0.0146269 | 0.00214834 | 9.89919775080583e-12 |
| 82 | BMI | rs11778219 | G | A | 0.163123 | 0.015796 | 0.0026874 | 4.20000686246632e-09 |
| 83 | BMI | rs118019496 | C | G | 0.072011 | 0.0208509 | 0.00393905 | 1.19999655704812e-07 |
| 84 | BMI | rs118136827 | T | G | 0.281086 | -0.0132582 | 0.0022019 | 1.70000422215636e-09 |
| 85 | BMI | rs1191600 | A | C | 0.593587 | -0.0122197 | 0.00202687 | 1.70000422215636e-09 |
| 86 | BMI | rs11919665 | T | A | 0.679775 | -0.0127854 | 0.00211292 | 1.40000633223953e-09 |
| 87 | BMI | rs12043569 | G | C | 0.220535 | -0.00311667 | 0.00238302 | 0.190000175 |
| 88 | BMI | rs12072739 | G | A | 0.224478 | 0.0157008 | 0.00236691 | 3.29989407910834e-11 |
| 89 | BMI | rs12089815 | A | G | 0.54861 | -0.0123121 | 0.00198606 | 5.69993627094142e-10 |
| 90 | BMI | rs12101393 | G | C | 0.21723 | -0.0160905 | 0.00241172 | 2.49976970217851e-11 |
| 91 | BMI | rs12140153 | T | G | 0.094252 | -0.033075 | 0.0034589 | 1.20005182020427e-21 |
| 92 | BMI | rs12149660 | A | G | 0.114956 | -0.0227338 | 0.00311586 | 2.99985318119079e-13 |
| 93 | BMI | rs12151636 | C | T | 0.030364 | -0.0286555 | 0.00573776 | 5.89997267139774e-07 |
| 94 | BMI | rs12259464 | A | G | 0.48447 | 0.0130895 | 0.00198686 | 4.49987033872151e-11 |
| 95 | BMI | rs12273545 | T | C | 0.056505 | 0.0248393 | 0.00428551 | 6.8000170244089e-09 |
| 96 | BMI | rs1229984 | C | T | 0.972775 | 0.037357 | 0.00599385 | 4.60002296665069e-10 |
| 97 | BMI | rs12340969 | T | C | 0.441399 | -0.0208868 | 0.0019941 | 1.10001852750622e-25 |
| 98 | BMI | rs12364470 | G | T | 0.164556 | 0.0192705 | 0.0026658 | 4.90004422793237e-13 |
| 99 | BMI | rs12414412 | G | C | 0.085559 | 0.0178692 | 0.00354626 | 4.70002318179801e-07 |
| 100 | BMI | rs12440603 | T | C | 0.433693 | 0.0139285 | 0.0020011 | 3.40016505804071e-12 |
| 101 | BMI | rs12462975 | A | G | 0.329692 | 0.0195822 | 0.00212071 | 2.60015956316527e-20 |
| 102 | BMI | rs12478299 | C | T | 0.252002 | -0.0118213 | 0.00227588 | 2.10000341026661e-07 |
| 103 | BMI | rs12507026 | T | A | 0.43475 | 0.0289896 | 0.0019931 | 6.29941180548562e-48 |
| 104 | BMI | rs12541408 | C | T | 0.31742 | -0.0143261 | 0.00212723 | 1.5999263828686e-11 |
| 105 | BMI | rs12633841 | T | G | 0.178084 | 0.0137453 | 0.00260024 | 1.19999655704812e-07 |
| 106 | BMI | rs1263629 | G | A | 0.143824 | 0.0176064 | 0.00282352 | 4.49997395325803e-10 |
| 107 | BMI | rs1266874 | G | A | 0.349619 | 0.0140966 | 0.0020712 | 1e-11 |
| 108 | BMI | rs12681792 | A | C | 0.1926 | 0.0148693 | 0.00251693 | 3.50001576071014e-09 |
| 109 | BMI | rs12692596 | T | C | 0.37185 | 0.0130872 | 0.00203738 | 1.29998996537545e-10 |
| 110 | BMI | rs12696039 | G | A | 0.149405 | -0.0152802 | 0.0027705 | 3.50001576071014e-08 |
| 111 | BMI | rs12712767 | A | G | 0.370925 | 0.0114221 | 0.00204487 | 2.3000114603619e-08 |
| 112 | BMI | rs12776809 | A | G | 0.0345 | 0.0269838 | 0.00549192 | 8.99994799637202e-07 |

| 113 | BMI | rs1285245 | C | G | 0.374354 | -0.0110029 | 0.00204848 | 7.79991733536343e-08 |
| --- | --- | --- | --- | --- | --- | --- | --- | --- |
| 114 | BMI | rs1286058 | A | T | 0.703747 | 0.0149126 | 0.00216922 | 6.20011864208725e-12 |
| 115 | BMI | rs12881629 | G | A | 0.08265 | 0.0220748 | 0.00358929 | 7.69998714277137e-10 |
| 116 | BMI | rs12921986 | G | A | 0.077984 | 0.0203327 | 0.0036983 | 3.79996853036855e-08 |
| 117 | BMI | rs12937411 | T | C | 0.408167 | -0.017187 | 0.00201383 | 1.39990962656834e-17 |
| 118 | BMI | rs1296328 | C | A | 0.559032 | -0.018862 | 0.001999 | 3.90031784475066e-21 |
| 119 | BMI | rs12974458 | T | C | 0.543175 | 0.0152466 | 0.00199717 | 2.29985258668621e-14 |
| 120 | BMI | rs12987009 | T | A | 0.440106 | 0.0106312 | 0.00198844 | 8.99994799637202e-08 |
| 121 | BMI | rs13012070 | A | G | 0.228388 | -0.0136501 | 0.00234795 | 6.10000231740111e-09 |
| 122 | BMI | rs13033310 | A | G | 0.25276 | 0.012611 | 0.0022826 | 3.29997006285229e-08 |
| 123 | BMI | rs13034936 | C | T | 0.111789 | -0.00138206 | 0.00312867 | 0.660000098 |
| 124 | BMI | rs13041173 | G | A | 0.341592 | 0.0103878 | 0.00209138 | 6.80001702440891e-07 |
| 125 | BMI | rs13097918 | A | T | 0.212315 | 0.0145561 | 0.0024161 | 1.70000422215636e-09 |
| 126 | BMI | rs13107325 | T | C | 0.07492 | 0.0475799 | 0.00375479 | 8.49962959449622e-37 |
| 127 | BMI | rs13163306 | A | G | 0.466035 | -0.0104 | 0.00198176 | 1.49999565138204e-07 |
| 128 | BMI | rs13176429 | C | T | 0.687609 | 0.0141555 | 0.00213122 | 3.1002734419982e-11 |
| 129 | BMI | rs1320251 | T | C | 0.454791 | -0.0180321 | 0.00199407 | 1.50003019045595e-19 |
| 130 | BMI | rs13218383 | G | C | 0.335132 | -0.0144025 | 0.00209236 | 5.79962615819643e-12 |
| 131 | BMI | rs1322842 | G | A | 0.609274 | -0.0131292 | 0.00203539 | 1.09999319893519e-10 |
| 132 | BMI | rs13248187 | C | T | 0.268557 | 0.015764 | 0.00224155 | 1.99986186963274e-12 |
| 133 | BMI | rs1327259 | G | A | 0.387722 | -0.0148532 | 0.00203363 | 2.80027059501064e-13 |
| 134 | BMI | rs13290403 | C | T | 0.295157 | -0.0108073 | 0.00217093 | 6.40000038338762e-07 |
| 135 | BMI | rs13291723 | A | G | 0.570683 | 0.0110318 | 0.00199876 | 3.4000084782586e-08 |
| 136 | BMI | rs13427822 | G | A | 0.271198 | -0.0181515 | 0.00224142 | 5.60015438761557e-16 |
| 137 | BMI | rs1346841 | A | G | 0.405041 | -0.013056 | 0.0020172 | 9.70063133783958e-11 |
| 138 | BMI | rs1360201 | T | C | 0.481546 | 0.0130075 | 0.00197747 | 4.79954424931945e-11 |
| 139 | BMI | rs1377825 | G | C | 0.436808 | 0.00981636 | 0.00199879 | 9.09997082601283e-07 |
| 140 | BMI | rs1384131 | A | T | 0.420163 | -0.0101331 | 0.00201652 | 4.99999995007974e-07 |
| 141 | BMI | rs140159717 | T | C | 0.082292 | -0.0246882 | 0.00370967 | 2.80027059501064e-11 |
| 142 | BMI | rs1411432 | C | A | 0.186225 | 0.0214668 | 0.0025491 | 3.6999853117286e-17 |
| 143 | BMI | rs143121872 | C | T | 0.022529 | -0.0368683 | 0.00719077 | 2.90001336905407e-07 |
| 144 | BMI | rs1438945 | A | T | 0.715119 | -0.0133824 | 0.00219791 | 1.09999319893519e-09 |
| 145 | BMI | rs1441264 | A | G | 0.593681 | 0.0179033 | 0.00205854 | 3.40016505804072e-18 |
| 146 | BMI | rs1446577 | G | C | 0.255643 | -0.0147709 | 0.00225682 | 5.90065196995665e-11 |
| 147 | BMI | rs1451963 | T | G | 0.082266 | 0.022201 | 0.00360585 | 7.39997069733888e-10 |
| 148 | BMI | rs1454687 | G | C | 0.515371 | -0.0207916 | 0.00197396 | 6.09958095872238e-26 |
| 149 | BMI | rs1458156 | T | C | 0.488431 | 0.014075 | 0.00197951 | 1.20005182020427e-12 |
| 150 | BMI | rs145981104 | G | A | 0.063711 | 0.022711 | 0.00404552 | 2.0000000199681e-08 |

| 151 | BMI | rs146569428 | A | G | 0.200749 | 0.0139533 | 0.00248571 | 2.0000000199681e-08 |
| --- | --- | --- | --- | --- | --- | --- | --- | --- |
| 152 | BMI | rs1471093 | A | G | 0.616663 | 0.0134619 | 0.00203996 | 4.10015240506613e-11 |
| 153 | BMI | rs1471740 | C | T | 0.7405 | 0.0193576 | 0.00225404 | 8.90020479625527e-18 |
| 154 | BMI | rs147568678 | C | T | 0.23807 | -0.0133327 | 0.00233034 | 1.09999319893519e-08 |
| 155 | BMI | rs1477290 | C | T | 0.136947 | 0.0337772 | 0.00289815 | 2.19988510904926e-31 |
| 156 | BMI | rs147730268 | T | G | 0.087243 | -0.0350799 | 0.00358357 | 1.29987023738861e-22 |
| 157 | BMI | rs150215901 | A | T | 0.043065 | -0.0264027 | 0.0049414 | 9.09997082601283e-08 |
| 158 | BMI | rs1503526 | C | T | 0.480077 | 0.0154308 | 0.00197681 | 5.90065196995665e-15 |
| 159 | BMI | rs150684652 | T | C | 0.018788 | 0.0404522 | 0.0081056 | 5.99998272533644e-07 |
| 160 | BMI | rs1523751 | A | G | 0.50339 | -0.0104319 | 0.00197889 | 1.40000633223953e-07 |
| 161 | BMI | rs156201 | C | G | 0.753455 | 0.0131899 | 0.00228932 | 8.3000364575513e-09 |
| 162 | BMI | rs1582931 | A | G | 0.473242 | -0.0133419 | 0.0019956 | 2.29985258668621e-11 |
| 163 | BMI | rs1608113 | T | A | 0.364998 | -0.0117766 | 0.00205086 | 9.2999368597584e-09 |
| 164 | BMI | rs1609010 | G | A | 0.565728 | 0.0209773 | 0.0019963 | 7.89950721335818e-26 |
| 165 | BMI | rs164328 | T | G | 0.912558 | -0.00507172 | 0.00352893 | 0.149999911 |
| 166 | BMI | rs16965658 | G | A | 0.060091 | -0.0243736 | 0.00418404 | 5.69993627094142e-09 |
| 167 | BMI | rs17005677 | C | T | 0.305252 | 0.011166 | 0.0021523 | 2.10000341026661e-07 |
| 168 | BMI | rs17056301 | C | T | 0.256425 | 0.0135831 | 0.0022697 | 2.19998641983517e-09 |
| 169 | BMI | rs17132130 | C | G | 0.221467 | -0.0178418 | 0.00238564 | 7.50066898736068e-14 |
| 170 | BMI | rs17141778 | C | G | 0.417833 | 0.0109027 | 0.00200564 | 5.39995325071583e-08 |
| 171 | BMI | rs17289010 | G | A | 0.32787 | -0.0134661 | 0.00210438 | 1.60000006389793e-10 |
| 172 | BMI | rs17399739 | G | A | 0.068893 | 0.0270713 | 0.00391006 | 4.40047947835983e-12 |
| 173 | BMI | rs17446299 | G | C | 0.165544 | 0.0153235 | 0.00266515 | 8.89999986382574e-09 |
| 174 | BMI | rs17544384 | C | T | 0.210864 | 0.0140931 | 0.00241434 | 5.30005040639665e-09 |
| 175 | BMI | rs17668356 | G | C | 0.146029 | -0.0230543 | 0.00279318 | 1.50003019045595e-16 |
| 176 | BMI | rs17724992 | G | A | 0.26777 | -0.017199 | 0.00224218 | 1.69980851320349e-14 |
| 177 | BMI | rs17741830 | C | T | 0.080179 | 0.0188535 | 0.00366863 | 2.80001269243454e-07 |
| 178 | BMI | rs17770336 | T | C | 0.322437 | 0.0242931 | 0.00211161 | 1.29987023738861e-30 |
| 179 | BMI | rs1778830 | A | G | 0.362165 | 0.0140775 | 0.00205596 | 7.50066898736068e-12 |
| 180 | BMI | rs1793636 | C | G | 0.309144 | -0.0133343 | 0.0021408 | 4.70002318179801e-10 |
| 181 | BMI | rs1818917 | T | C | 0.512835 | -0.010138 | 0.0019854 | 3.29997006285229e-07 |
| 182 | BMI | rs1834144 | A | C | 0.373192 | -0.0140112 | 0.00205213 | 8.60003066888856e-12 |
| 183 | BMI | rs1860750 | A | T | 0.510958 | 0.0117038 | 0.00198192 | 3.50001576071014e-09 |
| 184 | BMI | rs1861410 | T | C | 0.555423 | -0.0212558 | 0.00198918 | 1.20005182020427e-26 |
| 185 | BMI | rs1884897 | G | A | 0.627378 | 0.020001 | 0.00205634 | 2.2998525866862e-22 |
| 186 | BMI | rs189252432 | T | G | 0.012383 | -0.0452179 | 0.0089427 | 4.3000152915127e-07 |
| 187 | BMI | rs1919243 | C | T | 0.487406 | 0.0116759 | 0.00200175 | 5.49996593976403e-09 |
| 188 | BMI | rs1928706 | A | G | 0.471082 | -0.0119847 | 0.00198846 | 1.70000422215636e-09 |

| 189 | BMI | rs1934102 | A | G | 0.347583 | -0.0136682 | 0.00210374 | 8.19973845457475e-11 |
| --- | --- | --- | --- | --- | --- | --- | --- | --- |
| 190 | BMI | rs1967772 | A | G | 0.285117 | -0.0170434 | 0.00220377 | 1e-14 |
| 191 | BMI | rs1990662 | C | A | 0.192721 | 0.0144775 | 0.00251798 | 8.89999986382574e-09 |
| 192 | BMI | rs2035936 | T | G | 0.055892 | 0.0370863 | 0.00435778 | 1.69980851320349e-17 |
| 193 | BMI | rs2051559 | C | T | 0.13254 | 0.0204078 | 0.00291963 | 2.80027059501064e-12 |
| 194 | BMI | rs2102278 | G | A | 0.322486 | 0.0118583 | 0.0021139 | 2.0000000199681e-08 |
| 195 | BMI | rs2133561 | T | A | 0.611084 | -0.014097 | 0.00204744 | 5.79962615819643e-12 |
| 196 | BMI | rs213518 | C | T | 0.145608 | 0.0157894 | 0.00280491 | 1.79998961724559e-08 |
| 197 | BMI | rs2153740 | G | A | 0.479914 | -0.0112551 | 0.00199313 | 1.60000006389793e-08 |
| 198 | BMI | rs215634 | G | A | 0.611879 | -0.0155223 | 0.00203492 | 2.39993787680988e-14 |
| 199 | BMI | rs2172131 | C | T | 0.578723 | -0.0149382 | 0.00200386 | 8.99911910870052e-14 |
| 200 | BMI | rs217672 | C | A | 0.271741 | 0.0170155 | 0.00223046 | 2.39993787680988e-14 |
| 201 | BMI | rs2181350 | G | A | 0.713555 | 0.0110405 | 0.00220538 | 5.60002544078001e-07 |
| 202 | BMI | rs2190887 | T | C | 0.560766 | -0.0108576 | 0.0019937 | 5.19995996533516e-08 |
| 203 | BMI | rs2192158 | G | A | 0.553308 | -0.015012 | 0.00198302 | 3.69998531172859e-14 |
| 204 | BMI | rs2193101 | G | C | 0.812878 | -0.0142847 | 0.00256649 | 2.59997995670925e-08 |
| 205 | BMI | rs2216931 | A | C | 0.661966 | 0.0169109 | 0.00208587 | 5.19995996533515e-16 |
| 206 | BMI | rs2234458 | T | C | 0.639519 | -0.0203841 | 0.00205579 | 3.59997927043357e-23 |
| 207 | BMI | rs2249825 | C | G | 0.266743 | -0.0146183 | 0.00223793 | 6.49980009683026e-11 |
| 208 | BMI | rs2253310 | G | C | 0.626099 | 0.0173213 | 0.00204115 | 2.09990670376242e-17 |
| 209 | BMI | rs2270494 | C | G | 0.549272 | -0.00169816 | 0.00199844 | 0.400000008 |
| 210 | BMI | rs2271189 | A | G | 0.40272 | -0.0163086 | 0.00201869 | 6.49980009683026e-16 |
| 211 | BMI | rs2275003 | G | A | 0.520932 | -0.0119236 | 0.00197803 | 1.70000422215636e-09 |
| 212 | BMI | rs2275444 | A | G | 0.725748 | -0.0117967 | 0.00220827 | 9.20004602515511e-08 |
| 213 | BMI | rs227638 | C | T | 0.855893 | 0.0145294 | 0.00282979 | 2.80001269243454e-07 |
| 214 | BMI | rs2283093 | T | C | 0.202897 | 0.0124862 | 0.00245748 | 3.79996853036855e-07 |
| 215 | BMI | rs2289379 | T | C | 0.395648 | -0.0152765 | 0.00202952 | 5.19995996533515e-14 |
| 216 | BMI | rs2307111 | C | T | 0.395025 | -0.0280042 | 0.00202203 | 1.29987023738861e-43 |
| 217 | BMI | rs2342892 | G | T | 0.516212 | -0.0126972 | 0.00197715 | 1.29998996537545e-10 |
| 218 | BMI | rs2376481 | A | G | 0.469013 | 0.010039 | 0.00198509 | 4.3000152915127e-07 |
| 219 | BMI | rs2381404 | C | T | 0.243837 | 0.0139664 | 0.00229974 | 1.29998996537545e-09 |
| 220 | BMI | rs2396625 | A | T | 0.421331 | -0.018919 | 0.00201335 | 5.60015438761557e-21 |
| 221 | BMI | rs2398861 | G | A | 0.259169 | 0.0179932 | 0.00226733 | 2.09990670376243e-15 |
| 222 | BMI | rs2416927 | C | G | 0.437901 | 0.0107584 | 0.00199231 | 6.69992590760657e-08 |
| 223 | BMI | rs242556 | T | A | 0.766365 | 0.0122492 | 0.00239472 | 3.09998790941119e-07 |
| 224 | BMI | rs2425816 | A | G | 0.415111 | 0.0122284 | 0.00201106 | 1.19999655704811e-09 |
| 225 | BMI | rs2433733 | A | G | 0.677659 | -0.0171805 | 0.00210907 | 3.80014352941586e-16 |
| 226 | BMI | rs2439823 | G | A | 0.545612 | 0.0191998 | 0.00199108 | 5.30029448835795e-22 |

| 227 | BMI | rs2482356 | C | T | 0.429018 | -0.0113511 | 0.00199506 | 1.29998996537546e-08 |
| --- | --- | --- | --- | --- | --- | --- | --- | --- |
| 228 | BMI | rs2512892 | C | T | 0.566085 | 0.0129468 | 0.00199819 | 9.20025786648235e-11 |
| 229 | BMI | rs252761 | T | G | 0.587966 | -0.0115046 | 0.00201769 | 1.19999655704812e-08 |
| 230 | BMI | rs2555502 | T | C | 0.375396 | 0.0107332 | 0.00203879 | 1.40000633223953e-07 |
| 231 | BMI | rs2568958 | A | G | 0.603656 | 0.0222928 | 0.00201204 | 1.5999263828686e-28 |
| 232 | BMI | rs2591131 | A | G | 0.18606 | 0.0129122 | 0.00253333 | 3.50001576071014e-07 |
| 233 | BMI | rs2606228 | C | A | 0.646368 | -0.0138791 | 0.00208379 | 2.70022528688824e-11 |
| 234 | BMI | rs2616143 | A | G | 0.319862 | -0.0138738 | 0.00212547 | 6.70038873807757e-11 |
| 235 | BMI | rs2618039 | T | A | 0.381481 | 0.0143963 | 0.0020317 | 1.39990962656834e-12 |
| 236 | BMI | rs2646355 | C | T | 0.44952 | 0.0100921 | 0.0019903 | 4.00000007987242e-07 |
| 237 | BMI | rs2660971 | C | G | 0.653608 | 0.0110522 | 0.00207699 | 1e-07 |
| 238 | BMI | rs2678204 | G | T | 0.340162 | 0.024161 | 0.00208158 | 3.80014352941585e-31 |
| 239 | BMI | rs2699189 | G | A | 0.457933 | 0.0100676 | 0.00199626 | 4.60002296665068e-07 |
| 240 | BMI | rs2725371 | G | A | 0.696115 | -0.0160301 | 0.00215845 | 1.10001852750622e-13 |
| 241 | BMI | rs2781668 | T | C | 0.165587 | 0.0148965 | 0.00265988 | 2.10000341026661e-08 |
| 242 | BMI | rs2791643 | T | C | 0.761794 | -0.0133837 | 0.00231332 | 7.19995861275188e-09 |
| 243 | BMI | rs2833971 | T | C | 0.101527 | -0.0174088 | 0.00329097 | 1.19999655704812e-07 |
| 244 | BMI | rs28350 | G | A | 0.820684 | -0.0180335 | 0.00258234 | 2.90001336905407e-12 |
| 245 | BMI | rs28366156 | C | T | 0.130595 | -0.0264826 | 0.00292978 | 1.5999263828686e-19 |
| 246 | BMI | rs2837996 | C | T | 0.651408 | 0.0126647 | 0.00207859 | 1.09999319893519e-09 |
| 247 | BMI | rs28404639 | T | C | 0.366041 | -0.0117129 | 0.00205533 | 1.19999655704812e-08 |
| 248 | BMI | rs284532 | G | T | 0.522907 | -0.0103859 | 0.00197456 | 1.40000633223953e-07 |
| 249 | BMI | rs28454448 | G | A | 0.718026 | -0.0108647 | 0.00219591 | 7.49997818203003e-07 |
| 250 | BMI | rs28489620 | A | G | 0.290349 | -0.0153737 | 0.00220025 | 2.80027059501064e-12 |
| 251 | BMI | rs2861685 | C | T | 0.411984 | -0.0171274 | 0.00199763 | 1e-17 |
| 252 | BMI | rs28670671 | C | T | 0.286024 | -0.0124638 | 0.00226426 | 3.6999853117286e-08 |
| 253 | BMI | rs28678108 | G | A | 0.447933 | -0.0105721 | 0.00200988 | 1.40000633223953e-07 |
| 254 | BMI | rs2870111 | T | C | 0.412066 | -0.0157151 | 0.00201912 | 7.10068096254533e-15 |
| 255 | BMI | rs2875762 | C | G | 0.242953 | 0.015339 | 0.0023121 | 3.29989407910834e-11 |
| 256 | BMI | rs2899644 | T | C | 0.229995 | 0.0149659 | 0.0023609 | 2.3000114603619e-10 |
| 257 | BMI | rs2919389 | T | C | 0.401102 | 0.0106626 | 0.00202845 | 1.49999565138204e-07 |
| 258 | BMI | rs2920503 | T | C | 0.285413 | -0.0140307 | 0.00219666 | 1.70000422215636e-10 |
| 259 | BMI | rs2941452 | G | A | 0.617751 | 0.0111622 | 0.00203237 | 4.00000007987242e-08 |
| 260 | BMI | rs2962334 | T | G | 0.020065 | 0.0432552 | 0.00703547 | 7.79991733536343e-10 |
| 261 | BMI | rs2973564 | G | A | 0.300096 | -0.0108878 | 0.00215596 | 4.39997288359989e-07 |
| 262 | BMI | rs303753 | A | G | 0.346357 | 0.0183835 | 0.00209802 | 1.90020300257723e-18 |
| 263 | BMI | rs303948 | G | A | 0.092303 | 0.0173902 | 0.00343535 | 4.09996359041765e-07 |
| 264 | BMI | rs308632 | T | C | 0.253977 | -0.0120344 | 0.00229082 | 1.49999565138204e-07 |

| 265 | BMI | rs317656 | A | T | 0.724452 | -0.0144763 | 0.00221303 | 6.09958095872238e-11 |
| --- | --- | --- | --- | --- | --- | --- | --- | --- |
| 266 | BMI | rs3212038 | G | A | 0.328484 | 0.0149201 | 0.0021124 | 1.5999263828686e-12 |
| 267 | BMI | rs3213943 | A | C | 0.131525 | -0.0179406 | 0.00288163 | 4.7999863240391e-10 |
| 268 | BMI | rs32421 | T | A | 0.224315 | 0.0132315 | 0.00237757 | 2.59997995670925e-08 |
| 269 | BMI | rs329118 | T | C | 0.419409 | -0.0165734 | 0.0020038 | 1.2998702373886e-16 |
| 270 | BMI | rs329651 | T | G | 0.803977 | 0.0157218 | 0.00250202 | 3.29997006285228e-10 |
| 271 | BMI | rs34045288 | T | C | 0.334435 | 0.0234784 | 0.00209371 | 3.50025754158434e-29 |
| 272 | BMI | rs34153025 | C | T | 0.022158 | -0.0389151 | 0.00678155 | 9.59997274392481e-09 |
| 273 | BMI | rs34173062 | A | G | 0.072487 | -0.0197835 | 0.00403679 | 9.49992113622595e-07 |
| 274 | BMI | rs34481751 | A | C | 0.165329 | -0.0185029 | 0.00270404 | 7.80009693716499e-12 |
| 275 | BMI | rs34517439 | A | C | 0.121787 | 0.038848 | 0.00304983 | 3.59997927043357e-37 |
| 276 | BMI | rs34696181 | C | T | 0.476084 | 0.0114345 | 0.00198283 | 8.1000929031245e-09 |
| 277 | BMI | rs347551 | G | C | 0.472268 | 0.013865 | 0.00201085 | 5.40007759066593e-12 |
| 278 | BMI | rs34774377 | C | T | 0.119083 | -0.0162216 | 0.00304947 | 1e-07 |
| 279 | BMI | rs34811474 | A | G | 0.230756 | -0.0285293 | 0.00234269 | 4.10015240506613e-34 |
| 280 | BMI | rs349071 | A | G | 0.500388 | -0.0133062 | 0.00198231 | 1.90020300257723e-11 |
| 281 | BMI | rs35154326 | G | A | 0.274044 | -0.0130427 | 0.00223442 | 5.30005040639665e-09 |
| 282 | BMI | rs35364449 | T | C | 0.109739 | 0.0217069 | 0.00318335 | 9.20025786648235e-12 |
| 283 | BMI | rs35408866 | A | G | 0.136155 | 0.0143322 | 0.00289027 | 7.1000269957767e-07 |
| 284 | BMI | rs355777 | C | G | 0.407537 | 0.0152715 | 0.00201314 | 3.29989407910835e-14 |
| 285 | BMI | rs35697587 | A | G | 0.508121 | -0.0164677 | 0.00198067 | 9.20025786648231e-17 |
| 286 | BMI | rs35697691 | G | C | 0.089346 | 0.0230305 | 0.00352223 | 6.20011864208726e-11 |
| 287 | BMI | rs35746264 | G | A | 0.4545 | -0.0104463 | 0.00198671 | 1.49999565138204e-07 |
| 288 | BMI | rs35809007 | A | G | 0.363205 | -0.0171009 | 0.00205627 | 9.09913272632251e-17 |
| 289 | BMI | rs35957544 | T | G | 0.574316 | -0.0196429 | 0.00200438 | 1.10001852750622e-22 |
| 290 | BMI | rs36007635 | A | G | 0.137698 | -0.021045 | 0.00286788 | 2.19988510904925e-13 |
| 291 | BMI | rs36029912 | C | T | 0.051467 | 0.0236515 | 0.00449695 | 1.40000633223953e-07 |
| 292 | BMI | rs36061954 | T | C | 0.398747 | 0.0128445 | 0.00201928 | 2.00000001996811e-10 |
| 293 | BMI | rs3730071 | A | C | 0.030224 | -0.0287785 | 0.0057765 | 6.29999202947419e-07 |
| 294 | BMI | rs3764625 | G | T | 0.587594 | -0.0117714 | 0.00201523 | 5.19995996533515e-09 |
| 295 | BMI | rs3766823 | A | G | 0.17196 | 0.016056 | 0.0026141 | 8.1000929031245e-10 |
| 296 | BMI | rs3784710 | C | T | 0.226602 | -0.0297067 | 0.00236042 | 2.49976970217851e-36 |
| 297 | BMI | rs3803286 | G | A | 0.666815 | -0.0186417 | 0.00209828 | 6.40029512108372e-19 |
| 298 | BMI | rs3807566 | T | G | 0.43832 | -0.0120727 | 0.00199583 | 1.49999565138204e-09 |
| 299 | BMI | rs3814883 | T | C | 0.482402 | 0.0240107 | 0.00198424 | 1e-33 |
| 300 | BMI | rs3847199 | T | C | 0.629109 | -0.0104341 | 0.00208528 | 5.60002544078001e-07 |
| 301 | BMI | rs3848453 | C | T | 0.583691 | -0.0103353 | 0.00201282 | 2.80001269243454e-07 |
| 302 | BMI | rs3851998 | G | C | 0.743064 | -0.0136071 | 0.00226874 | 2.00000001996811e-09 |

| 303 | BMI | rs3866805 | A | C | 0.355683 | 0.0117835 | 0.00206546 | 1.19999655704812e-08 |
| --- | --- | --- | --- | --- | --- | --- | --- | --- |
| 304 | BMI | rs3897102 | T | C | 0.411157 | 0.0120838 | 0.00202716 | 2.49999995007974e-09 |
| 305 | BMI | rs3901286 | A | C | 0.15244 | -0.0225553 | 0.00275602 | 2.70022528688824e-16 |
| 306 | BMI | rs3935190 | A | G | 0.536784 | -0.0144864 | 0.00199701 | 4.00036851046125e-13 |
| 307 | BMI | rs394608 | C | T | 0.537697 | 0.0186353 | 0.00199484 | 9.49948365876343e-21 |
| 308 | BMI | rs396755 | G | C | 0.571164 | -0.0123639 | 0.00199937 | 6.29999202947421e-10 |
| 309 | BMI | rs40071 | C | T | 0.179536 | -0.0261666 | 0.00258163 | 3.80014352941585e-24 |
| 310 | BMI | rs4017425 | T | C | 0.470208 | -0.0125817 | 0.00198035 | 2.10000341026661e-10 |
| 311 | BMI | rs4055791 | T | C | 0.416804 | -0.0178082 | 0.00200801 | 7.39945954312723e-19 |
| 312 | BMI | rs40631 | T | G | 0.60984 | 0.0106931 | 0.00214239 | 5.99998272533644e-07 |
| 313 | BMI | rs406388 | G | C | 0.177266 | 0.015973 | 0.00260181 | 8.3000364575513e-10 |
| 314 | BMI | rs41279738 | G | T | 0.025988 | 0.0684263 | 0.0062225 | 4.00036851046125e-28 |
| 315 | BMI | rs41315816 | C | T | 0.058989 | 0.0254409 | 0.00419129 | 1.29998996537545e-09 |
| 316 | BMI | rs4148155 | G | A | 0.113268 | -0.0229681 | 0.00310659 | 1.39990962656834e-13 |
| 317 | BMI | rs4235838 | A | G | 0.325427 | -0.010385 | 0.00211862 | 9.49992113622595e-07 |
| 318 | BMI | rs4261944 | G | T | 0.364935 | 0.0138553 | 0.00205652 | 1.5999263828686e-11 |
| 319 | BMI | rs4284600 | C | T | 0.467122 | 0.0119673 | 0.00199506 | 2.00000001996811e-09 |
| 320 | BMI | rs429343 | G | A | 0.576573 | -0.0173795 | 0.00199729 | 3.29989407910835e-18 |
| 321 | BMI | rs429358 | C | T | 0.154166 | -0.0266723 | 0.00274373 | 2.39993787680987e-22 |
| 322 | BMI | rs4307239 | G | A | 0.458941 | 0.0121413 | 0.00198816 | 1e-09 |
| 323 | BMI | rs4397962 | C | T | 0.220937 | 0.0124083 | 0.00239471 | 2.19998641983516e-07 |
| 324 | BMI | rs4419475 | T | A | 0.407327 | 0.011487 | 0.0020106 | 1.09999319893519e-08 |
| 325 | BMI | rs4430672 | C | T | 0.800968 | -0.0131059 | 0.00248286 | 1.29998996537546e-07 |
| 326 | BMI | rs4456769 | T | C | 0.333444 | 0.0146111 | 0.00210138 | 3.59997927043357e-12 |
| 327 | BMI | rs4477562 | T | C | 0.128644 | 0.0296118 | 0.00298011 | 2.90001336905406e-23 |
| 328 | BMI | rs4482463 | A | C | 0.923002 | -0.0313175 | 0.00370692 | 2.99985318119079e-17 |
| 329 | BMI | rs4524456 | A | G | 0.524995 | -0.0103094 | 0.00197662 | 1.79998961724559e-07 |
| 330 | BMI | rs45486197 | A | G | 0.065685 | 0.025766 | 0.00403737 | 1.70000422215636e-10 |
| 331 | BMI | rs4605363 | C | A | 0.341595 | 0.0163638 | 0.00207733 | 3.29989407910835e-15 |
| 332 | BMI | rs4625888 | T | C | 0.557644 | 0.0117507 | 0.00198303 | 3.09998790941119e-09 |
| 333 | BMI | rs4643716 | A | C | 0.583213 | 0.0017969 | 0.00201543 | 0.370000235 |
| 334 | BMI | rs4648450 | A | C | 0.466824 | -0.014837 | 0.00198775 | 8.4004006526377e-14 |
| 335 | BMI | rs4658403 | T | C | 0.833659 | -0.0188877 | 0.00264997 | 1e-12 |
| 336 | BMI | rs4672338 | T | C | 0.336228 | 0.0135646 | 0.00208669 | 8.000184448551e-11 |
| 337 | BMI | rs4700780 | T | C | 0.298382 | 0.0109388 | 0.00216197 | 4.20000686246631e-07 |
| 338 | BMI | rs4722398 | T | C | 0.136137 | 0.0186993 | 0.00287545 | 7.89950721335818e-11 |
| 339 | BMI | rs4734122 | G | A | 0.576826 | -0.0105889 | 0.00200486 | 1.29998996537546e-07 |
| 340 | BMI | rs4737188 | T | A | 0.473864 | -0.0124447 | 0.00198274 | 3.50001576071014e-10 |

| 341 | BMI | rs4764949 | G | A | 0.325892 | -0.0183924 | 0.00211197 | 3.10027344199818e-18 |
| --- | --- | --- | --- | --- | --- | --- | --- | --- |
| 342 | BMI | rs4790292 | A | C | 0.153693 | -0.0254509 | 0.00275603 | 2.60015956316527e-20 |
| 343 | BMI | rs4800756 | A | G | 0.647047 | -0.0109567 | 0.00207269 | 1.19999655704812e-07 |
| 344 | BMI | rs4820410 | G | A | 0.345317 | -0.0177457 | 0.00208527 | 1.69980851320349e-17 |
| 345 | BMI | rs4832298 | T | C | 0.686142 | -0.015965 | 0.00212268 | 5.40007759066593e-14 |
| 346 | BMI | rs4858940 | C | T | 0.885569 | 0.0229281 | 0.00309976 | 1.39990962656834e-13 |
| 347 | BMI | rs4876611 | G | A | 0.720243 | 0.0197549 | 0.00220502 | 3.29989407910834e-19 |
| 348 | BMI | rs4884559 | G | T | 0.453724 | 0.00991374 | 0.00198819 | 6.19997588072326e-07 |
| 349 | BMI | rs4908672 | T | C | 0.39308 | 0.0102031 | 0.00202102 | 4.49997395325803e-07 |
| 350 | BMI | rs4929923 | C | T | 0.645204 | 0.0189424 | 0.00206436 | 4.49987033872149e-20 |
| 351 | BMI | rs4944769 | A | G | 0.282444 | 0.0108758 | 0.00221425 | 8.99994799637202e-07 |
| 352 | BMI | rs4947461 | C | T | 0.538506 | -0.0100302 | 0.00198235 | 4.20000686246631e-07 |
| 353 | BMI | rs4976553 | G | A | 0.19316 | -0.0134891 | 0.00252743 | 9.40004645744658e-08 |
| 354 | BMI | rs5011579 | G | C | 0.715152 | 0.0140268 | 0.00219322 | 1.60000006389793e-10 |
| 355 | BMI | rs512121 | C | T | 0.192054 | -0.0159359 | 0.00252176 | 2.59997995670925e-10 |
| 356 | BMI | rs529200 | G | A | 0.527829 | 0.0168965 | 0.0019785 | 1.29987023738861e-17 |
| 357 | BMI | rs539515 | C | A | 0.204942 | 0.0495291 | 0.0024426 | 1.99986186963275e-91 |
| 358 | BMI | rs55707359 | G | T | 0.015432 | 0.0531292 | 0.00812381 | 6.20011864208726e-11 |
| 359 | BMI | rs55714539 | C | A | 0.343587 | 0.0175719 | 0.00210043 | 6.00067353864114e-17 |
| 360 | BMI | rs55726687 | A | G | 0.209709 | 0.02483 | 0.00242628 | 1.39990962656834e-24 |
| 361 | BMI | rs55736343 | T | A | 0.198635 | 0.0124515 | 0.00247575 | 4.90004422793236e-07 |
| 362 | BMI | rs55769038 | A | G | 0.590384 | 0.0160961 | 0.00201096 | 1.20005182020427e-15 |
| 363 | BMI | rs557951 | G | T | 0.312937 | 0.0140534 | 0.00213387 | 4.49987033872151e-11 |
| 364 | BMI | rs558882 | C | A | 0.216607 | 0.0126811 | 0.00239887 | 1.19999655704812e-07 |
| 365 | BMI | rs558887 | G | A | 0.307507 | -0.0129976 | 0.00214881 | 1.49999565138204e-09 |
| 366 | BMI | rs559231 | T | G | 0.393053 | 0.0134907 | 0.00203562 | 3.40016505804071e-11 |
| 367 | BMI | rs56038322 | A | G | 0.31062 | 0.0139276 | 0.00214833 | 8.99911910870052e-11 |
| 368 | BMI | rs56133507 | G | T | 0.196875 | 0.0137622 | 0.00247385 | 2.69997659840121e-08 |
| 369 | BMI | rs56143236 | T | C | 0.256388 | 0.0126667 | 0.00226533 | 2.3000114603619e-08 |
| 370 | BMI | rs56161855 | T | A | 0.132847 | 0.0224575 | 0.00291784 | 1.39990962656834e-14 |
| 371 | BMI | rs56203622 | C | T | 0.145529 | 0.0179769 | 0.00280183 | 1.40000633223953e-10 |
| 372 | BMI | rs56211164 | A | G | 0.239602 | -0.012468 | 0.0023151 | 7.1999586127519e-08 |
| 373 | BMI | rs56352336 | C | T | 0.154894 | -0.016327 | 0.00275021 | 2.90001336905407e-09 |
| 374 | BMI | rs56399737 | T | C | 0.449124 | -0.0161325 | 0.00199646 | 6.40029512108372e-16 |
| 375 | BMI | rs56858768 | A | G | 0.296861 | 0.0159188 | 0.00217378 | 2.39993787680988e-13 |
| 376 | BMI | rs56893062 | G | T | 0.303342 | 0.0125141 | 0.00215383 | 6.19997588072324e-09 |
| 377 | BMI | rs56930105 | T | C | 0.139295 | 0.0157557 | 0.00286187 | 3.6999853117286e-08 |
| 378 | BMI | rs57263785 | G | A | 0.246397 | -0.011461 | 0.00229759 | 6.10000231740111e-07 |

| 379 | BMI | rs57636386 | C | T | 0.08383 | -0.0412553 | 0.00358326 | 1.10001852750622e-30 |
| --- | --- | --- | --- | --- | --- | --- | --- | --- |
| 380 | BMI | rs57803 | A | G | 0.82412 | -0.0128212 | 0.00260178 | 8.30003645755132e-07 |
| 381 | BMI | rs57989773 | C | T | 0.244995 | 0.0133488 | 0.00236315 | 1.60000006389793e-08 |
| 382 | BMI | rs587271 | T | C | 0.686873 | 0.0118147 | 0.00221758 | 9.9001095426017e-08 |
| 383 | BMI | rs58862095 | T | C | 0.419266 | -0.0229877 | 0.00200786 | 2.39993787680987e-30 |
| 384 | BMI | rs59068084 | T | G | 0.410232 | 0.0110552 | 0.00201048 | 3.79996853036855e-08 |
| 385 | BMI | rs59086897 | A | T | 0.487541 | 0.033385 | 0.00196986 | 1.99986186963275e-64 |
| 386 | BMI | rs59227842 | G | A | 0.31149 | 0.0229675 | 0.00215313 | 1.50003019045595e-26 |
| 387 | BMI | rs594024 | C | T | 0.554162 | -0.0146771 | 0.00199081 | 1.69980851320349e-13 |
| 388 | BMI | rs6000329 | A | G | 0.560302 | -0.0108389 | 0.00204125 | 1.09999319893519e-07 |
| 389 | BMI | rs6023655 | G | A | 0.765741 | -0.0147346 | 0.00234997 | 3.59997927043357e-10 |
| 390 | BMI | rs6074432 | T | C | 0.545198 | -0.0105337 | 0.00199253 | 1.19999655704812e-07 |
| 391 | BMI | rs60764613 | T | G | 0.144883 | 0.02099 | 0.00282688 | 1.10001852750622e-13 |
| 392 | BMI | rs6092194 | G | T | 0.556172 | 0.0104832 | 0.00199753 | 1.49999565138204e-07 |
| 393 | BMI | rs6134916 | T | C | 0.492722 | -0.0105412 | 0.00198529 | 1.09999319893519e-07 |
| 394 | BMI | rs61740466 | A | G | 0.237203 | -0.0135153 | 0.00231885 | 5.60002544078e-09 |
| 395 | BMI | rs61813324 | T | C | 0.135728 | 0.0290259 | 0.00292038 | 2.80027059501063e-23 |
| 396 | BMI | rs61828641 | A | G | 0.10925 | 0.0224585 | 0.00315913 | 1.20005182020427e-12 |
| 397 | BMI | rs61871615 | T | C | 0.091551 | -0.0267159 | 0.00359269 | 1e-13 |
| 398 | BMI | rs61903695 | G | A | 0.254964 | 0.0166235 | 0.00227115 | 2.49976970217851e-13 |
| 399 | BMI | rs61955525 | T | G | 0.195563 | -0.0124094 | 0.00252769 | 9.09997082601283e-07 |
| 400 | BMI | rs61992671 | G | A | 0.49195 | -0.0161925 | 0.00206924 | 5.10035023959446e-15 |
| 401 | BMI | rs62007782 | A | G | 0.265091 | -0.0167007 | 0.00224215 | 9.39939714766782e-14 |
| 402 | BMI | rs62020775 | A | T | 0.14168 | -0.0165665 | 0.0028635 | 7.19995861275188e-09 |
| 403 | BMI | rs62058023 | C | T | 0.134392 | 0.0151713 | 0.00290991 | 1.89998424621473e-07 |
| 404 | BMI | rs62062168 | G | C | 0.200125 | 0.0124853 | 0.00247393 | 4.49997395325803e-07 |
| 405 | BMI | rs62072006 | C | A | 0.144541 | 0.0156492 | 0.00282374 | 2.99999133271615e-08 |
| 406 | BMI | rs62107261 | C | T | 0.048327 | -0.0911559 | 0.00460882 | 4.60044666393483e-87 |
| 407 | BMI | rs62190049 | C | G | 0.390446 | -0.0111798 | 0.00203614 | 4.00000007987242e-08 |
| 408 | BMI | rs62240473 | G | A | 0.038948 | -0.0261724 | 0.00513132 | 3.40000847825859e-07 |
| 409 | BMI | rs62241847 | G | A | 0.314443 | -0.0124263 | 0.00212897 | 5.30005040639665e-09 |
| 410 | BMI | rs62379271 | G | T | 0.578511 | 0.0117237 | 0.00200557 | 4.99999995007973e-09 |
| 411 | BMI | rs62516785 | T | C | 0.064773 | -0.0203742 | 0.00401688 | 3.89995862874433e-07 |
| 412 | BMI | rs6265 | T | C | 0.188472 | -0.0399185 | 0.00252719 | 3.29989407910837e-56 |
| 413 | BMI | rs633284 | T | A | 0.515594 | -0.0104184 | 0.00200049 | 1.89998424621473e-07 |
| 414 | BMI | rs6430068 | A | G | 0.108559 | 0.0186066 | 0.0031882 | 5.30005040639665e-09 |
| 415 | BMI | rs6444950 | A | G | 0.237474 | 0.0158525 | 0.00232007 | 8.30041869715702e-12 |
| 416 | BMI | rs6445258 | C | T | 0.792336 | -0.0127333 | 0.00243071 | 1.60000006389793e-07 |

| 417 | BMI | rs6474856 | T | C | 0.64022 | -0.0122041 | 0.00207048 | 3.79996853036855e-09 |
| --- | --- | --- | --- | --- | --- | --- | --- | --- |
| 418 | BMI | rs6531639 | A | G | 0.247548 | -0.0136291 | 0.00233909 | 5.69993627094142e-09 |
| 419 | BMI | rs6545714 | A | G | 0.601448 | -0.0205219 | 0.00201573 | 2.39993787680987e-24 |
| 420 | BMI | rs6560906 | C | T | 0.691874 | -0.012198 | 0.00214136 | 1.19999655704812e-08 |
| 421 | BMI | rs6561937 | A | T | 0.753587 | -0.015934 | 0.00230346 | 4.60044666393479e-12 |
| 422 | BMI | rs6567160 | C | T | 0.232714 | 0.0541723 | 0.00234213 | 2.30144181740848e-118 |
| 423 | BMI | rs6575340 | A | G | 0.636038 | 0.0207333 | 0.00206189 | 8.69961433065266e-24 |
| 424 | BMI | rs6597975 | G | C | 0.54389 | 0.013681 | 0.00199532 | 7.10068096254533e-12 |
| 425 | BMI | rs6606580 | G | A | 0.589256 | -0.0107126 | 0.00200826 | 9.59997274392481e-08 |
| 426 | BMI | rs66679256 | T | C | 0.445826 | 0.0148864 | 0.00198759 | 6.89922009844049e-14 |
| 427 | BMI | rs6669189 | T | C | 0.401588 | 0.0171368 | 0.00201682 | 1.90020300257723e-17 |
| 428 | BMI | rs6682438 | C | T | 0.673081 | 0.0131586 | 0.00210047 | 3.69998531172859e-10 |
| 429 | BMI | rs6696828 | C | G | 0.303943 | 0.0137805 | 0.00214061 | 1.19999655704811e-10 |
| 430 | BMI | rs6705567 | C | T | 0.37596 | -0.0146168 | 0.00204919 | 9.79941161095592e-13 |
| 431 | BMI | rs6707827 | G | A | 0.703627 | 0.0119437 | 0.00217332 | 3.89995862874433e-08 |
| 432 | BMI | rs6710091 | G | C | 0.348174 | -0.0116835 | 0.00206707 | 1.60000006389793e-08 |
| 433 | BMI | rs6713781 | C | G | 0.401882 | -0.0135736 | 0.00202799 | 2.19988510904925e-11 |
| 434 | BMI | rs6725931 | T | C | 0.847658 | 0.0191046 | 0.00274403 | 3.29989407910835e-12 |
| 435 | BMI | rs6744646 | G | A | 0.828299 | 0.0554684 | 0.00261213 | 4.49987033872149e-100 |
| 436 | BMI | rs6752979 | A | G | 0.31688 | 0.0125235 | 0.00211721 | 3.29997006285228e-09 |
| 437 | BMI | rs67609008 | C | T | 0.283614 | 0.0170863 | 0.0022021 | 8.60003066888856e-15 |
| 438 | BMI | rs6774894 | A | T | 0.358148 | 0.0133088 | 0.00205674 | 9.70063133783958e-11 |
| 439 | BMI | rs6791296 | C | T | 0.881898 | 0.0151398 | 0.00306868 | 8.09990639374006e-07 |
| 440 | BMI | rs6805758 | C | A | 0.331093 | 0.0103377 | 0.00210062 | 8.60003066888856e-07 |
| 441 | BMI | rs6824271 | A | T | 0.272079 | 0.0111673 | 0.00226075 | 7.79991733536343e-07 |
| 442 | BMI | rs6831088 | A | G | 0.640139 | -0.0115222 | 0.0020591 | 2.19998641983516e-08 |
| 443 | BMI | rs6834120 | G | C | 0.138038 | -0.0145813 | 0.0028963 | 4.79998632403909e-07 |
| 444 | BMI | rs6843852 | T | C | 0.507861 | 0.0130897 | 0.00197607 | 3.50025754158434e-11 |
| 445 | BMI | rs6909685 | T | C | 0.326809 | -0.014612 | 0.00211352 | 4.70002318179801e-12 |
| 446 | BMI | rs6922607 | G | A | 0.189847 | 0.0148848 | 0.00251479 | 3.20000015974484e-09 |
| 447 | BMI | rs6938973 | C | T | 0.601456 | 0.0182223 | 0.00201872 | 1.800113960418e-19 |
| 448 | BMI | rs6950388 | A | G | 0.795086 | 0.0155219 | 0.00244806 | 2.3000114603619e-10 |
| 449 | BMI | rs6962980 | C | A | 0.556019 | -0.0159568 | 0.00198814 | 1e-15 |
| 450 | BMI | rs698147 | G | A | 0.543596 | -0.0128512 | 0.00198514 | 9.60063590945348e-11 |
| 451 | BMI | rs6998660 | G | A | 0.452592 | 0.0125456 | 0.00198902 | 2.80001269243454e-10 |
| 452 | BMI | rs7024334 | G | T | 0.77914 | -0.0138177 | 0.00238338 | 6.69992590760657e-09 |
| 453 | BMI | rs7027304 | T | C | 0.652661 | 0.0145492 | 0.00208703 | 3.10027344199819e-12 |
| 454 | BMI | rs7034554 | G | A | 0.373825 | -0.0129826 | 0.00204252 | 2.10000341026661e-10 |

| 455 | BMI | rs7038943 | C | T | 0.33879 | -0.0140202 | 0.00208641 | 1.80011396041801e-11 |
| --- | --- | --- | --- | --- | --- | --- | --- | --- |
| 456 | BMI | rs704061 | C | T | 0.45505 | 0.0146374 | 0.00198552 | 1.69980851320349e-13 |
| 457 | BMI | rs705145 | A | C | 0.34518 | 0.0139021 | 0.0020798 | 2.29985258668621e-11 |
| 458 | BMI | rs7070670 | T | C | 0.327923 | -0.0123274 | 0.00211928 | 5.99998272533644e-09 |
| 459 | BMI | rs7081254 | C | T | 0.205739 | -0.0142516 | 0.00245216 | 6.19997588072324e-09 |
| 460 | BMI | rs7109581 | G | T | 0.42015 | 0.0104979 | 0.00200472 | 1.60000006389793e-07 |
| 461 | BMI | rs7124681 | A | C | 0.408353 | 0.0256977 | 0.0020063 | 1.50003019045594e-37 |
| 462 | BMI | rs7132908 | A | G | 0.384458 | 0.0297904 | 0.00203363 | 1.39990962656833e-48 |
| 463 | BMI | rs713598 | G | C | 0.402092 | 0.00129273 | 0.00201402 | 0.519999589 |
| 464 | BMI | rs71495038 | A | G | 0.076936 | 0.0277998 | 0.00370969 | 6.70038873807757e-14 |
| 465 | BMI | rs71511072 | T | G | 0.176696 | -0.0127765 | 0.00260627 | 9.49992113622595e-07 |
| 466 | BMI | rs7201895 | A | G | 0.354291 | -0.0149781 | 0.00207999 | 6.00067353864116e-13 |
| 467 | BMI | rs7206608 | G | C | 0.32162 | 0.0135122 | 0.00211797 | 1.79998961724559e-10 |
| 468 | BMI | rs7218014 | C | T | 0.197305 | 0.0189524 | 0.00249216 | 2.90001336905407e-14 |
| 469 | BMI | rs7232171 | T | G | 0.58263 | 0.0123404 | 0.00200821 | 8.00000023961727e-10 |
| 470 | BMI | rs723672 | T | C | 0.43151 | 0.0111273 | 0.00200698 | 2.99999133271615e-08 |
| 471 | BMI | rs7259070 | C | T | 0.596062 | 0.021869 | 0.00203628 | 6.59933234263567e-27 |
| 472 | BMI | rs72634826 | A | G | 0.259858 | -0.021277 | 0.00228001 | 1e-20 |
| 473 | BMI | rs72649373 | C | T | 0.143188 | 0.0178062 | 0.00287805 | 6.10000231740111e-10 |
| 474 | BMI | rs72673947 | G | A | 0.107031 | 0.0218864 | 0.00321496 | 9.89919775080583e-12 |
| 475 | BMI | rs72744924 | G | A | 0.089309 | 0.0177733 | 0.00350016 | 3.79996853036855e-07 |
| 476 | BMI | rs72813172 | A | G | 0.040281 | -0.0259199 | 0.00512141 | 4.20000686246631e-07 |
| 477 | BMI | rs7283057 | G | A | 0.746283 | -0.0114364 | 0.00229354 | 6.19997588072326e-07 |
| 478 | BMI | rs72866851 | T | A | 0.102263 | 0.0172545 | 0.00325254 | 1.09999319893519e-07 |
| 479 | BMI | rs72892910 | T | G | 0.172232 | 0.0387798 | 0.00262053 | 1.50003019045594e-49 |
| 480 | BMI | rs72915955 | A | G | 0.161058 | -0.0143794 | 0.00270606 | 1.09999319893519e-07 |
| 481 | BMI | rs72925179 | A | G | 0.181677 | -0.0131229 | 0.00256072 | 2.99999133271615e-07 |
| 482 | BMI | rs72976986 | A | G | 0.190123 | -0.0232257 | 0.00254712 | 7.59976214361039e-20 |
| 483 | BMI | rs73026725 | A | C | 0.153534 | -0.022318 | 0.00275052 | 4.90004422793237e-16 |
| 484 | BMI | rs73034216 | C | T | 0.172517 | -0.0137126 | 0.00262688 | 1.79998961724559e-07 |
| 485 | BMI | rs73052033 | C | T | 0.18493 | -0.0303929 | 0.00254634 | 7.70016444356869e-33 |
| 486 | BMI | rs7306534 | A | G | 0.621712 | -0.0112754 | 0.00205437 | 4.09996359041765e-08 |
| 487 | BMI | rs7306544 | C | T | 0.105534 | 0.0162564 | 0.00322284 | 4.60002296665068e-07 |
| 488 | BMI | rs73124396 | C | T | 0.205144 | -0.0154472 | 0.0024548 | 3.09998790941119e-10 |
| 489 | BMI | rs73142879 | T | C | 0.192297 | -0.0266936 | 0.00252257 | 3.59997927043357e-26 |
| 490 | BMI | rs73193736 | G | A | 0.243934 | -0.0177165 | 0.00232002 | 2.19988510904925e-14 |
| 491 | BMI | rs73213484 | T | A | 0.141236 | -0.0225766 | 0.0028364 | 1.69980851320349e-15 |
| 492 | BMI | rs7331420 | A | G | 0.285276 | -0.0143702 | 0.00220081 | 6.59933234263564e-11 |

| 493 | BMI | rs73529119 | T | C | 0.113761 | 0.0158465 | 0.00312557 | 4.00000007987242e-07 |
| --- | --- | --- | --- | --- | --- | --- | --- | --- |
| 494 | BMI | rs7357754 | G | A | 0.500139 | 0.0141146 | 0.00198362 | 1.10001852750622e-12 |
| 495 | BMI | rs73601548 | T | C | 0.114527 | 0.0177354 | 0.00311639 | 1.29998996537546e-08 |
| 496 | BMI | rs73985439 | C | A | 0.307299 | 0.0136616 | 0.00214059 | 1.70000422215636e-10 |
| 497 | BMI | rs74252325 | T | C | 0.212418 | 0.0116298 | 0.00236977 | 9.20004602515511e-07 |
| 498 | BMI | rs7442885 | G | C | 0.214049 | -0.0228163 | 0.00241251 | 3.19963176731834e-21 |
| 499 | BMI | rs745249 | T | C | 0.281945 | 0.0176118 | 0.00219724 | 1.10001852750622e-15 |
| 500 | BMI | rs74750282 | C | T | 0.086623 | -0.0196382 | 0.00352561 | 2.49999995007974e-08 |
| 501 | BMI | rs74887628 | A | G | 0.035119 | 0.0291468 | 0.00551198 | 1.19999655704812e-07 |
| 502 | BMI | rs7498665 | G | A | 0.399659 | 0.0268646 | 0.00201997 | 2.2998525866862e-40 |
| 503 | BMI | rs7519259 | A | G | 0.52836 | 0.0140028 | 0.00198381 | 1.69980851320349e-12 |
| 504 | BMI | rs7546040 | G | C | 0.731068 | -0.00299329 | 0.00222154 | 0.180000205 |
| 505 | BMI | rs754635 | G | C | 0.886582 | 0.0219925 | 0.0031093 | 1.50003019045595e-12 |
| 506 | BMI | rs75499503 | T | C | 0.220101 | -0.0180331 | 0.00241845 | 8.90020479625527e-14 |
| 507 | BMI | rs7568228 | C | G | 0.52717 | -0.0119625 | 0.00197288 | 1.29998996537545e-09 |
| 508 | BMI | rs7571496 | G | A | 0.260545 | -0.0158916 | 0.00225454 | 1.80011396041801e-12 |
| 509 | BMI | rs760644 | A | G | 0.81982 | -0.0138904 | 0.00256345 | 5.99998272533644e-08 |
| 510 | BMI | rs76183894 | C | T | 0.080747 | -0.0219584 | 0.00364437 | 1.70000422215636e-09 |
| 511 | BMI | rs7619139 | A | T | 0.588616 | 0.0134537 | 0.00201061 | 2.19988510904925e-11 |
| 512 | BMI | rs7621422 | T | C | 0.498136 | -0.0101242 | 0.00198083 | 3.20000015974484e-07 |
| 513 | BMI | rs76267866 | T | A | 0.206473 | 0.0141438 | 0.00243965 | 6.69992590760657e-09 |
| 514 | BMI | rs76469486 | T | A | 0.079993 | 0.0178738 | 0.00363611 | 8.79994585505891e-07 |
| 515 | BMI | rs765874 | A | T | 0.489362 | -0.0120592 | 0.00197569 | 1e-09 |
| 516 | BMI | rs76702514 | G | C | 0.210623 | -0.0164862 | 0.00243278 | 1.20005182020427e-11 |
| 517 | BMI | rs7683836 | A | G | 0.557215 | -0.0122469 | 0.00199427 | 8.19992726270379e-10 |
| 518 | BMI | rs7704382 | G | C | 0.433528 | 0.0120126 | 0.00199631 | 1.79998961724559e-09 |
| 519 | BMI | rs7708584 | G | A | 0.572344 | -0.0159321 | 0.0019949 | 1.39990962656834e-15 |
| 520 | BMI | rs7742698 | G | T | 0.366664 | 0.0104163 | 0.00205209 | 3.89995862874433e-07 |
| 521 | BMI | rs7761673 | A | T | 0.21988 | -0.0135915 | 0.0023901 | 1.29998996537546e-08 |
| 522 | BMI | rs7762794 | G | A | 0.285408 | 0.0149077 | 0.00218606 | 9.09913272632251e-12 |
| 523 | BMI | rs7774 | A | C | 0.310467 | 0.014988 | 0.00215169 | 3.29989407910835e-12 |
| 524 | BMI | rs7776021 | A | G | 0.287599 | 0.0123701 | 0.00218251 | 1.40000633223953e-08 |
| 525 | BMI | rs78012460 | G | A | 0.022673 | -0.0370828 | 0.00666929 | 2.69997659840121e-08 |
| 526 | BMI | rs7805441 | T | C | 0.502261 | 0.0133747 | 0.00198884 | 1.80011396041801e-11 |
| 527 | BMI | rs7819514 | A | G | 0.336901 | -0.011289 | 0.00210031 | 7.69998714277137e-08 |
| 528 | BMI | rs7828631 | T | C | 0.109989 | 0.0170372 | 0.00316553 | 7.39997069733888e-08 |
| 529 | BMI | rs7833023 | A | C | 0.725555 | 0.0116858 | 0.00222312 | 1.49999565138204e-07 |
| 530 | BMI | rs784257 | C | T | 0.812541 | 0.0179315 | 0.00254968 | 1.99986186963274e-12 |

| 531 | BMI | rs78605811 | C | A | 0.05405 | -0.032737 | 0.00444673 | 1.80011396041801e-13 |
| --- | --- | --- | --- | --- | --- | --- | --- | --- |
| 532 | BMI | rs78886584 | G | A | 0.491021 | 0.0116661 | 0.0019948 | 4.99999995007973e-09 |
| 533 | BMI | rs7893571 | T | G | 0.665897 | 0.0140553 | 0.00210152 | 2.29985258668621e-11 |
| 534 | BMI | rs7900590 | C | T | 0.943063 | 0.0227457 | 0.00439339 | 2.3000114603619e-07 |
| 535 | BMI | rs79027764 | C | T | 0.0208 | -0.0347438 | 0.00691196 | 4.99999995007974e-07 |
| 536 | BMI | rs7924036 | T | G | 0.503265 | -0.0142836 | 0.0019778 | 5.10035023959446e-13 |
| 537 | BMI | rs7925100 | A | G | 0.396117 | 0.014725 | 0.0020221 | 3.29989407910835e-13 |
| 538 | BMI | rs7942037 | C | G | 0.360333 | -0.0161952 | 0.00205793 | 3.59997927043357e-15 |
| 539 | BMI | rs7944782 | G | T | 0.509784 | 0.0157606 | 0.00198748 | 2.19988510904925e-15 |
| 540 | BMI | rs7947143 | A | G | 0.163483 | -0.0182782 | 0.00267522 | 8.30041869715702e-12 |
| 541 | BMI | rs7975187 | G | A | 0.213905 | 0.0151361 | 0.00241251 | 3.50001576071014e-10 |
| 542 | BMI | rs79780963 | T | C | 0.077428 | 0.0236803 | 0.00369591 | 1.49999565138204e-10 |
| 543 | BMI | rs79906980 | T | C | 0.159775 | 0.0139322 | 0.00269613 | 2.39999313805789e-07 |
| 544 | BMI | rs79966207 | C | T | 0.175809 | 0.0133328 | 0.00260526 | 3.09998790941119e-07 |
| 545 | BMI | rs7996639 | A | G | 0.449354 | 0.0145559 | 0.00200245 | 3.59997927043357e-13 |
| 546 | BMI | rs80135274 | T | A | 0.070324 | 0.021339 | 0.00388113 | 3.79996853036855e-08 |
| 547 | BMI | rs8015400 | A | C | 0.677097 | 0.0213422 | 0.00211709 | 6.70038873807757e-24 |
| 548 | BMI | rs8020365 | A | T | 0.220464 | 0.0251068 | 0.00239517 | 1e-25 |
| 549 | BMI | rs8024137 | T | A | 0.848207 | 0.0156329 | 0.00276778 | 1.60000006389793e-08 |
| 550 | BMI | rs8025516 | G | T | 0.645928 | -0.0146558 | 0.00207578 | 1.69980851320349e-12 |
| 551 | BMI | rs8033510 | C | T | 0.635692 | -0.010822 | 0.00206373 | 1.60000006389793e-07 |
| 552 | BMI | rs8063946 | T | C | 0.05448 | -0.0251967 | 0.00436586 | 7.90005291083221e-09 |
| 553 | BMI | rs8076669 | C | T | 0.561561 | 0.0140681 | 0.00199601 | 1.80011396041801e-12 |
| 554 | BMI | rs8089514 | A | T | 0.36868 | 0.012988 | 0.00207588 | 3.89995862874433e-10 |
| 555 | BMI | rs8112818 | G | A | 0.400329 | -0.020696 | 0.00202712 | 1.800113960418e-24 |
| 556 | BMI | rs8124896 | C | T | 0.100956 | 0.0176068 | 0.00329514 | 9.09997082601283e-08 |
| 557 | BMI | rs8137518 | G | C | 0.057355 | 0.0222316 | 0.00435241 | 3.29997006285229e-07 |
| 558 | BMI | rs815163 | C | T | 0.563148 | -0.016485 | 0.00198582 | 1e-16 |
| 559 | BMI | rs8176166 | C | T | 0.148627 | -0.0138993 | 0.00279375 | 6.49994976198143e-07 |
| 560 | BMI | rs852042 | G | A | 0.758575 | -0.0131207 | 0.00231309 | 1.40000633223953e-08 |
| 561 | BMI | rs861578 | G | C | 0.5541 | 0.0106328 | 0.00199877 | 1e-07 |
| 562 | BMI | rs862320 | T | C | 0.40965 | -0.0231703 | 0.00201345 | 1.20005182020427e-30 |
| 563 | BMI | rs878627 | T | C | 0.379167 | -0.0102569 | 0.00206186 | 6.49994976198143e-07 |
| 564 | BMI | rs909892 | A | G | 0.134817 | -0.0184105 | 0.00291255 | 2.59997995670925e-10 |
| 565 | BMI | rs923994 | G | A | 0.783195 | -0.0145761 | 0.00240219 | 1.29998996537545e-09 |
| 566 | BMI | rs9294260 | A | G | 0.476559 | 0.014782 | 0.00198818 | 1e-13 |
| 567 | BMI | rs9299525 | A | G | 0.411515 | 0.0107189 | 0.00200786 | 9.40004645744658e-08 |
| 568 | BMI | rs9349235 | T | C | 0.410635 | 0.0111809 | 0.00200924 | 2.59997995670925e-08 |

| 569 | BMI | rs935166 | A | G | 0.506805 | -0.0161066 | 0.00197292 | 3.19963176731835e-16 |
| --- | --- | --- | --- | --- | --- | --- | --- | --- |
| 570 | BMI | rs9366863 | C | T | 0.671833 | -0.0285083 | 0.00210043 | 5.79962615819639e-42 |
| 571 | BMI | rs9375702 | T | C | 0.691158 | -0.0111999 | 0.0021384 | 1.60000006389793e-07 |
| 572 | BMI | rs9388446 | A | T | 0.515653 | 0.0111013 | 0.00197919 | 2.0000000199681e-08 |
| 573 | BMI | rs9395885 | T | C | 0.083575 | 0.0180094 | 0.00356556 | 4.39997288359989e-07 |
| 574 | BMI | rs9461887 | T | C | 0.277069 | 0.0148892 | 0.00220206 | 1.39990962656834e-11 |
| 575 | BMI | rs9463175 | T | C | 0.338958 | -0.0115 | 0.00210115 | 4.39997288359989e-08 |
| 576 | BMI | rs9478496 | C | T | 0.164225 | 0.0181836 | 0.00267493 | 1.10001852750622e-11 |
| 577 | BMI | rs9515446 | G | A | 0.447709 | 0.0151051 | 0.00199052 | 3.19963176731835e-14 |
| 578 | BMI | rs9522180 | T | C | 0.553392 | -0.0141095 | 0.00199325 | 1.50003019045595e-12 |
| 579 | BMI | rs9571687 | A | C | 0.329363 | -0.0135388 | 0.0021094 | 1.40000633223953e-10 |
| 580 | BMI | rs9585326 | G | A | 0.53318 | -0.00182722 | 0.00198659 | 0.359999585 |
| 581 | BMI | rs961498 | C | G | 0.503131 | 0.011981 | 0.00199336 | 1.79998961724559e-09 |
| 582 | BMI | rs9638713 | G | A | 0.97477 | -0.0361735 | 0.00635399 | 1.19999655704812e-08 |
| 583 | BMI | rs9673839 | G | A | 0.490967 | 0.0130337 | 0.00198783 | 5.50047252867633e-11 |
| 584 | BMI | rs9674487 | G | C | 0.001338 | 0.158445 | 0.0286171 | 3.09998790941119e-08 |
| 585 | BMI | rs979724 | A | G | 0.301023 | -0.0116167 | 0.0021525 | 6.80001702440891e-08 |
| 586 | BMI | rs9830592 | A | C | 0.582421 | 0.0154849 | 0.00200164 | 1e-14 |
| 587 | BMI | rs9839081 | A | G | 0.325232 | -0.011701 | 0.00214045 | 4.60002296665068e-08 |
| 588 | BMI | rs9843653 | C | T | 0.511652 | 0.0294509 | 0.00197538 | 2.90001336905409e-50 |
| 589 | BMI | rs9876664 | T | G | 0.375389 | -0.0180478 | 0.00204174 | 9.60063590945344e-19 |
| 590 | BMI | rs9888533 | T | C | 0.538079 | 0.0120038 | 0.0020182 | 2.69997659840121e-09 |
| 591 | BMI | rs9926784 | C | T | 0.184576 | -0.023818 | 0.00254677 | 8.60003066888856e-21 |
| 592 | BMI | rs9931586 | G | C | 0.270097 | -0.0122407 | 0.00224111 | 4.70002318179801e-08 |
| 593 | BMI | rs9951619 | G | T | 0.767369 | 0.0144231 | 0.00235777 | 9.49992113622595e-10 |
| 594 | BMI | rs995460 | C | A | 0.459664 | 0.00972167 | 0.00198645 | 9.9001095426017e-07 |
| 595 | BMI | rs9954755 | C | G | 0.538505 | 0.0107007 | 0.0019943 | 8.09990639374006e-08 |
| 596 | BMI | rs9992189 | C | G | 0.599503 | -0.0101033 | 0.00201587 | 5.39995325071583e-07 |
