# Supplementary material for: Red Wine May Mitigate the Risk of Intracerebral Hemorrhage by Preventing Hypertension—A Mendelian Randomization Study Combining CHARLS
Source: Food Sci Nutr. 2025 Dec 12;13(12):e71329. doi: 10.1002/fsn3.71329 (PMC12701324; doi:10.1002/fsn3.71329)
Supplement: Supplementary file 1 — Table S1: Detailed information for the GWAS datasets used in the study. Table S2: Instrumental variables used in two‐sample MR analysis of alcohol to ICH. Table S3: Instrumental variables used in two‐sample MR analysis of red wine to ICH. Table S4: Instrumental variables used in two‐sample MR analysis of white wine to ICH. Table S5: Instrumental variables used in multivariable MR analysis of alcohol to ICH. Table S6: Instrumental variables used in multivariable MR analysis of red wine to ICH. Table S7: Instrumental variables used in two‐sample MR analysis of ICH to red wine. Table S8: Instrumental variables used in two‐sample MR analysis of red wine to hypertension. Table S9: Instrumental variables used in two‐sample MR analysis of hypertension to ICH. Table S10: Instrumental variables used in MVMR analysis of red wine to hypertension. Table S11: Instrumental variables used in MVMR analysis of BMI to hypertension. Table S12: Two‐sample MR analysis result of alcohol to ICH. Table S13: Two‐sample MR analysis result of red wine to ICH. Table S14: Two‐sample MR analysis result of white wine to ICH. Table S15: Multivariable MR analysis result of alcohol and red wine to ICH. Multivariable inverse‐variance weighted method. Table S16: Two‐sample MR analysis result of ICH to red wine. Table S17: Two‐sample MR analysis result of red wine to hypertension. Table S18: Two‐sample MR analysis result of hypertension to ICH. Table S19: Multivariable MR analysis result of BMI and red wine to hypertension. Table S20: STROBE‐MR checklist of recommended items to address in reports of Mendelian randomization studies. [file FSN3-13-e71329-s001.zip › fsn371329-sup-0004-TableS12-S19@supplementary_data3_MR_results.docx]

**Table S12 Two-sample MR analysis result of alcohol to ICH.**

|  | **id.exposure** | **id.outcome** | **outcome** | **exposure** | **method** | **nsnp** | **b** | **se** | **lo_ci** | **up_ci** | **or** | **r_lci9** | **or_uci95** | **pval** |
| --- | --- | --- | --- | --- | --- | --- | --- | --- | --- | --- | --- | --- | --- | --- |
| 1 | ukb-b-5779 | finngen_R10_I9_ICH | ICH | alcohol | MR Egger | 237 | 0.699 | 0.284 | 0.142 | 1.26 | 2.013 | 1.15 | 3.514 | 0.0146 |
| 2 | ukb-b-5779 | finngen_R10_I9_ICH | ICH | alcohol | Weighted median | 237 | 0.26 | 0.134 | -0.002 | 0.52 | 1.296 | 1 | 1.6854 | 0.0525 |
| 3 | ukb-b-5779 | finngen_R10_I9_ICH | ICH | alcohol | Inverse variance weighted | 237 | 0.191 | 0.091 | 0.0134 | 0.37 | 1.211 | 1.01 | 1.4471 | 0.0351 |
| 4 | ukb-b-5779 | finngen_R10_I9_ICH | ICH | alcohol | Simple mode | 237 | 0.337 | 0.385 | -0.418 | 1.09 | 1.401 | 0.67 | 2.9229 | 0.3702 |
| 5 | ukb-b-5779 | finngen_R10_I9_ICH | ICH | alcohol | Weighted mode | 237 | 0.379 | 0.259 | -0.129 | 0.89 | 1.461 | 0.84 | 2.5314 | 0.1776 |

|  | **id.exposure** | **id.outcome** | **outcome** | **exposure** | **method** | **Q** | **Q_df** | **Q_pval** |
| --- | --- | --- | --- | --- | --- | --- | --- | --- |
| 1 | ukb-b-5779 | finngen_R10_I9_ICH | ICH | alcohol | MR Egger | 257.16 | 235 | 0.153 |
| 2 | ukb-b-5779 | finngen_R10_I9_ICH | ICH | alcohol | Inverse variance weighted | 261.05 | 236 | 0.126 |

|  | **id.exposure** | **id.outcome** | **outcome** | **exposure** | **egger_intercept** | **se** | **pval** |
| --- | --- | --- | --- | --- | --- | --- | --- |
| 1 | ukb-b-5779 | finngen_R10_I9_ICH | ICH | alcohol | -0.011132194 | 0.0059 | 0.061 |

**Table S13 Two-sample MR analysis result of red wine to ICH.**

|  | **id.exposure** | **id.outcome** | **outcome** | **exposure** | **method** | **nsnp** | **b** | **se** | **lo_ci** | **up_ci** | **or** | **or_lci95** | **or_uci95** | **pval** |
| --- | --- | --- | --- | --- | --- | --- | --- | --- | --- | --- | --- | --- | --- | --- |
| 1 | ukb-b-5239 | finngen_R10_I9_ICH | ICH | red wine | MR Egger | 95 | -1.21 | 0.8725 | -2.92 | 0.5006 | 0.2983 | 0.0539 | 1.6497 | 0.169 |
| 2 | ukb-b-5239 | finngen_R10_I9_ICH | ICH | red wine | Weighted median | 95 | -0.629 | 0.3283 | -1.272 | 0.0148 | 0.5332 | 0.2802 | 1.0149 | 0.0555 |
| 3 | ukb-b-5239 | finngen_R10_I9_ICH | ICH | red wine | Inverse variance weighted | 95 | -0.495 | 0.241 | -0.968 | -0.023 | 0.6095 | 0.38 | 0.9776 | 0.04 |
| 4 | ukb-b-5239 | finngen_R10_I9_ICH | ICH | red wine | Simple mode | 95 | -0.862 | 0.8391 | -2.506 | 0.7827 | 0.4224 | 0.0816 | 2.1874 | 0.307 |
| 5 | ukb-b-5239 | finngen_R10_I9_ICH | ICH | red wine | Weighted mode | 95 | -1.007 | 0.7708 | -2.518 | 0.5036 | 0.3653 | 0.0806 | 1.6546 | 0.1945 |

|  | **id.exposure** | **id.outcome** | **outcome** | **exposure** | **method** | **Q** | **Q_df** | **Q_pval** |
| --- | --- | --- | --- | --- | --- | --- | --- | --- |
| 1 | ukb-b-5239 | finngen_R10_I9_ICH | ICH | red wine | MR Egger | 109.45 | 93 | 0.1171 |
| 2 | ukb-b-5239 | finngen_R10_I9_ICH | ICH | red wine | Inverse variance weighted | 110.3 | 94 | 0.1201 |

|  | **id.exposure** | **id.outcome** | **outcome** | **exposure** | **egger_intercept** | **se** | **pval** |
| --- | --- | --- | --- | --- | --- | --- | --- |
| 1 | ukb-b-5239 | finngen_R10_I9_ICH | ICH | red wine | 0.009406287 | 0.011 | 0.3964 |

**Table S14 Two-sample MR analysis result of white wine to ICH.**

|  | **id.exposure** | **id.outcome** | **outcome** | **exposure** | **method** | **nsnp** | **b** | **se** | **lo_ci** | **up_ci** | **or** | **or_lci95** | **or_uci95** | **pval** |
| --- | --- | --- | --- | --- | --- | --- | --- | --- | --- | --- | --- | --- | --- | --- |
| 1 | ukb-b-5716 | finngen_R10_I9_ICH | ICH | white wine | MR Egger | 46 | -1.091 | 0.9887 | -3.029 | 0.8472 | 0.336 | 0.0484 | 2.333 | 0.276 |
| 2 | ukb-b-5716 | finngen_R10_I9_ICH | ICH | white wine | Weighted median | 46 | -0.417 | 0.4655 | -1.33 | 0.4951 | 0.6588 | 0.2646 | 1.6407 | 0.37 |
| 3 | ukb-b-5716 | finngen_R10_I9_ICH | ICH | white wine | Inverse variance weighted | 46 | -0.215 | 0.3281 | -0.858 | 0.4284 | 0.8068 | 0.4241 | 1.5348 | 0.5129 |
| 4 | ukb-b-5716 | finngen_R10_I9_ICH | ICH | white wine | Simple mode | 46 | -1.251 | 1.0911 | -3.39 | 0.8874 | 0.2862 | 0.0337 | 2.4289 | 0.2576 |
| 5 | ukb-b-5716 | finngen_R10_I9_ICH | ICH | white wine | Weighted mode | 46 | -1.139 | 1.018 | -3.134 | 0.8565 | 0.3202 | 0.0435 | 2.355 | 0.2693 |

|  | **id.exposure** | **id.outcome** | **outcome** | **exposure** | **method** | **Q** | **Q_df** | **Q_pval** |
| --- | --- | --- | --- | --- | --- | --- | --- | --- |
| 1 | ukb-b-5716 | finngen_R10_I9_ICH | ICH | white wine | MR Egger | 46.19 | 44 | 0.3819 |
| 2 | ukb-b-5716 | finngen_R10_I9_ICH | ICH | white wine | Inverse variance weighted | 47.1164 | 45 | 0.386 |

|  | **id.exposure** | **id.outcome** | **outcome** | **exposure** | **egger_intercept** | **se** | **pval** |
| --- | --- | --- | --- | --- | --- | --- | --- |
| 1 | ukb-b-5716 | finngen_R10_I9_ICH | ICH | white wine | 0.011820362 | 0.01258 | 0.3527 |

# Table S15 Multivariable MR analysis result of alcohol and red wine to ICH.

## Multivariable inverse-variance weighted method

(variants uncorrelated, random-effect model)

Number of Variants : 266

| **exposure** | **IVW_beta** | **IVW_se** | **IVW_CILower** | **IVW_CIUpper** | **IVW_pvalue** |
| --- | --- | --- | --- | --- | --- |
| alcohol | 0.051723343 | 0.122850115 | -0.189058458 | 0.292505145 | 0.673734608 |
| red wine | -0.597669954 | 0.297058804 | -1.179894511 | -0.015445396 | 0.044224323 |

Residual standard error = 1.052

Heterogeneity test statistic = 292.3995 on 264 degrees of freedom, (p-value = 0.1107)

## Multivariable MR-Egger method

(variants uncorrelated, random-effect model)

Orientated to exposure : 1

Number of Variants : 266

| **exposure** | **EGGER_beta** | **EGGER_se** | **EGGER_CILower** | **EGGER_CIUpper** | **EGGER_p** |
| --- | --- | --- | --- | --- | --- |
| alcohol | 0.232292468 | 0.234188591 | -0.226708736 | 0.691293672 | 0.321244633 |
| red wine | -0.733393102 | 0.332801338 | -1.385671738 | -0.081114467 | 0.027545704 |
| (intercept) | -0.004449156 | 0.004911999 | -0.0140765 | 0.005178186 | 0.3650561 |

Residual standard error = 1.053

Heterogeneity test statistic = 291.4902 on 263 degrees of freedom, (p-value = 0.1096)

## Multivariable MR-Lasso method

Orientated to exposure : 1

Number of variants : 266

Number of valid instruments : 259 Tuning parameter : 0.1336534

| **exposure** | **LASSO_beta** | **LASSO_se** | **LASSO_CILower** | **LASSO_CIUpper** | **LASSO_p** |
| --- | --- | --- | --- | --- | --- |
| alcohol | 0.102065454 | 0.117983182 | -0.129177334 | 0.333308242 | 0.386992264 |
| red wine | -0.606026028 | 0.285869687 | -1.166320318 | -0.045731737 | 0.03401127 |

## Multivariable median method

Number of variants : 266

| **exposure** | **ME_beta** | **ME_se** | **ME_CILower** | **ME_CIUpper** | **ME_p** |
| --- | --- | --- | --- | --- | --- |
| alcohol | 0.046616686 | 0.172223071 | -0.29093433 | 0.384167701 | 0.786640086 |
| red wine | -0.786751044 | 0.40621274 | -1.582913384 | 0.009411296 | 0.052770335 |

Q-Statistic for instrument validity: 291.5611 on 263 DF , p-value: 0.1090273

**Table S16 Two-sample MR analysis result of ICH to red wine.**

|  | **id.exposure** | **id.outcome** | **outcome** | **exposure** | **method** | **nsnp** | **b** | **se** | **lo_ci** | **up_ci** | **or** | **or_lci95** | **or_uci95** | **pval** |
| --- | --- | --- | --- | --- | --- | --- | --- | --- | --- | --- | --- | --- | --- | --- |
| 1 | finngen_R10_I9_ICH | ukb-b-5239 | red wine | ICH | MR Egger | 11 | -0.001 | 0.0312 | -0.063 | 0.0598 | 0.9987 | 0.9394 | 1.0617 | 0.9667 |
| 2 | finngen_R10_I9_ICH | ukb-b-5239 | red wine | ICH | Weighted median | 11 | 0.0076 | 0.0091 | -0.01 | 0.0256 | 1.0077 | 0.9898 | 1.0259 | 0.4043 |
| 3 | finngen_R10_I9_ICH | ukb-b-5239 | red wine | ICH | verse variance weighte | 11 | 0.0043 | 0.008 | -0.011 | 0.02 | 1.0043 | 0.9886 | 1.0202 | 0.5964 |
| 4 | finngen_R10_I9_ICH | ukb-b-5239 | red wine | ICH | Simple mode | 11 | 0.0089 | 0.016 | -0.022 | 0.0403 | 1.009 | 0.9778 | 1.0411 | 0.5895 |
| 5 | finngen_R10_I9_ICH | ukb-b-5239 | red wine | ICH | Weighted mode | 11 | 0.0057 | 0.0147 | -0.023 | 0.0346 | 1.0057 | 0.9771 | 1.0352 | 0.7061 |

|  | **id.exposure** | **id.outcome** | **outcome** | **exposure** | **method** | **Q** | **Q_df** | **Q_pval** |
| --- | --- | --- | --- | --- | --- | --- | --- | --- |
| 1 | finngen_R10_I9_ICH | ukb-b-5239 | red wine | ICH | MR Egger | 15.8427 | 9 | 0.0702 |
| 2 | finngen_R10_I9_ICH | ukb-b-5239 | red wine | ICH | verse variance weighte | 15.9037 | 10 | 0.1024 |

|  | **id.exposure** | **id.outcome** | **outcome** | **exposure** | **egger_intercept** | **se** | **pval** |
| --- | --- | --- | --- | --- | --- | --- | --- |
| 1 | finngen_R10_I9_ICH | ukb-b-5239 | red wine | ICH | 0.000808974 | 0.00434 | 0.8564 |

**Table S17 Two-sample MR analysis result of red wine to hypertension.**

|  | **id.exposure** | **id.outcome** | **outcome** | **exposure** | **method** | **nsnp** | **b** | **se** | **lo_ci** | **up_ci** | **or** | **or_lci95** | **or_uci95** | **pval** |
| --- | --- | --- | --- | --- | --- | --- | --- | --- | --- | --- | --- | --- | --- | --- |
| 1 | ukb-b-5239 | finngen_R10_I9_HYPTENS | hypertension | red wine | MR Egger | 86 | -0.188 | 0.2561 | -0.69 | 0.3144 | 0.8289 | 0.5017 | 1.3694 | 0.4658 |
| 2 | ukb-b-5239 | finngen_R10_I9_HYPTENS | hypertension | red wine | Weighted median | 86 | -0.152 | 0.0911 | -0.33 | 0.0267 | 0.8591 | 0.7187 | 1.027 | 0.0955 |
| 3 | ukb-b-5239 | finngen_R10_I9_HYPTENS | hypertension | red wine | nverse variance weighted | 86 | -0.235 | 0.0699 | -0.372 | -0.098 | 0.7908 | 0.6895 | 0.907 | 0.0008 |
| 4 | ukb-b-5239 | finngen_R10_I9_HYPTENS | hypertension | red wine | Simple mode | 86 | -0.111 | 0.219 | -0.54 | 0.318 | 0.8948 | 0.5825 | 1.3744 | 0.6129 |
| 5 | ukb-b-5239 | finngen_R10_I9_HYPTENS | hypertension | red wine | Weighted mode | 86 | -0.13 | 0.2012 | -0.524 | 0.2644 | 0.8781 | 0.5919 | 1.3027 | 0.5201 |

|  | **id.exposure** | **id.outcome** | **outcome** | **exposure** | **method** | **Q** | **Q_df** | **Q_pval** |
| --- | --- | --- | --- | --- | --- | --- | --- | --- |
| 1 | ukb-b-5239 | finngen_R10_I9_HYPTENS | hypertension | red wine | MR Egger | 123.55 | 84 | 0.0032 |
| 2 | ukb-b-5239 | finngen_R10_I9_HYPTENS | hypertension | red wine | nverse variance weighted | 123.61 | 85 | 0.004 |

|  | **id.exposure** | **id.outcome** | **outcome** | **exposure** | **egger_intercept** | **se** | **pval** |
| --- | --- | --- | --- | --- | --- | --- | --- |
| 1 | ukb-b-5239 | finngen_R10_I9_HYPTENS | hypertension | red wine | -0.000616286 | 0.0032 | 0.8491 |

**Table S18 Two-sample MR analysis result of hypertension to ICH.**

|  | **id.exposure** | **id.outcome** | **outcome** | **exposure** | **method** | **nsnp** | **b** | **se** | **lo_ci** | **up_ci** | **or** | **or_lci95** | **or_uci95** | **pval** |
| --- | --- | --- | --- | --- | --- | --- | --- | --- | --- | --- | --- | --- | --- | --- |
| 1 | finngen_R10_I9_HY  PTENS | finngen_R10_I9_I  CH | ICH | hypertension | MR Egger | 367 | 0.1621 | 0.0909 | -0.016 | 0.3402 | 1.176 | 0.9841 | 1.4052 | 0.07534 |
| 2 | finngen_R10_I9_HY  PTENS | finngen_R10_I9_I  CH | ICH | hypertension | Weighted median | 367 | 0.3308 | 0.0519 | 0.2291 | 0.4325 | 1.3921 | 1.2575 | 1.5412 | 1.81E-10 |
| 3 | finngen_R10_I9_HY  PTENS | finngen_R10_I9_I  CH | ICH | hypertension | IVW | 367 | 0.2838 | 0.0334 | 0.2183 | 0.3492 | 1.3281 | 1.2439 | 1.418 | 1.98E-17 |
| 4 | finngen_R10_I9_HY  PTENS | finngen_R10_I9_I  CH | ICH | hypertension | Simple mode | 367 | 0.3171 | 0.1538 | 0.0157 | 0.6186 | 1.3732 | 1.0158 | 1.8563 | 0.0399 |
| 5 | finngen_R10_I9_HY  PTENS | finngen_R10_I9_I  CH | ICH | hypertension | Weighted mode | 367 | 0.4275 | 0.1226 | 0.1873 | 0.6678 | 1.5335 | 1.2059 | 1.9499 | 0.00055 |

|  | **id.exposure** | **id.outcome** | **outcome** | **exposure** | **method** | **Q** | **Q_df** | **Q_pval** |
| --- | --- | --- | --- | --- | --- | --- | --- | --- |
| 1 | finngen_R10_I9_HY  PTENS | finngen_R10_I9_I  CH | ICH | hypertension | MR Egger | 369.5 | 365 | 0.4245 |
| 2 | finngen_R10_I9_HY  PTENS | finngen_R10_I9_I  CH | ICH | hypertension | IVW | 371.59 | 366 | 0.4089 |

|  | **id.exposure** | **id.outcome** | **outcome** | **exposure** | **egger_intercept** | **se** | **pval** |
| --- | --- | --- | --- | --- | --- | --- | --- |
| 1 | finngen_R10_I9_HY  PTENS | finngen_R10_I9_I  CH | ICH | hypertension | 0.005783459 | 0.004 | 0.1509 |

# Table S19 Multivariable MR analysis result of BMI and red wine to hypertension.

## Multivariable inverse-variance weighted method

(variants uncorrelated, random-effect model)

Number of Variants : 596

| **exposure** | **IVW_beta** | **IVW_se** | **IVW_CILower** | **IVW_CIUpper** | **IVW_pvalue** |
| --- | --- | --- | --- | --- | --- |
| BMI | 0.471369959 | 0.028558141 | 0.415397032 | 0.527342886 | 3.34254186623853e-61 |
| red wine | -0.303321401 | 0.111050536 | -0.520976452 | -0.085666351 | 0.006306938 |

Residual standard error = 1.789

Heterogeneity test statistic = 1900.5919 on 594 degrees of freedom, (p-value = 0.0000)

## Multivariable MR-Egger method

(variants uncorrelated, random-effect model)

Orientated to exposure : 1

Number of Variants : 596

| **exposure** | **EGGER_beta** | **EGGER_se** | **EGGER_CILower** | **EGGER_CIUpper** | **EGGER_p** |
| --- | --- | --- | --- | --- | --- |
| BMI | 0.472661619 | 0.028844069 | 0.416128284 | 0.529194955 | 2.37678601208353e-60 |
| red wine | -0.341535958 | 0.160142703 | -0.655409889 | -0.027662027 | 0.032949541 |
| (intercept) | 0.000 | 0.001 | -0.001 | 0.002 | 0.740 |

Residual standard error = 1.790

Heterogeneity test statistic = 1900.2400 on 593 degrees of freedom, (p-value = 0.0000)

## Multivariable MR-Lasso method

Orientated to exposure : 1

Number of variants : 596

Number of valid instruments : 469

Tuning parameter : 0.08082065

| **exposure** | **LASSO_beta** | **LASSO_se** | **LASSO_CILower** | **LASSO_CIUpper** | **LASSO_p** |
| --- | --- | --- | --- | --- | --- |
| BMI | 0.546405495 | 0.019406601 | 0.508369256 | 0.584441734 | 2.04347364451509e-174 |
| red wine | -0.15409727 | 0.072755525 | -0.296695479 | -0.011499061 | 0.034173825 |

## Multivariable median method

Number of variants : 596

| **exposure** | **ME_beta** | **ME_se** | **ME_CILower** | **ME_CIUpper** | **ME_p** |
| --- | --- | --- | --- | --- | --- |
| BMI | 0.551422918 | 0.029224831 | 0.494143301 | 0.608702534 | 2.07874206160817e-79 |
| red wine | -0.110580865 | 0.104546265 | -0.315487779 | 0.094326049 | 0.290182286 |
